# Synthesis of BN-Polyarenes by a Mild Borylative Cyclization Cascade

**DOI:** 10.1021/acs.orglett.2c02477

**Published:** 2022-08-01

**Authors:** Ester Sans-Panadés, Juan J. Vaquero, Manuel A. Fernández-Rodríguez, Patricia García-García

**Affiliations:** Universidad de Alcalá (IRYCIS). Departamento de Química Orgánica y Química Inorgánica, Instituto de Investigación Química “Andrés M. del Río” (IQAR). Campus Científico-Tecnológico, Facultad de Farmacia. Autovía A-II, Km 33.1, 28805-Alcalá de Henares, Madrid, Spain

## Abstract

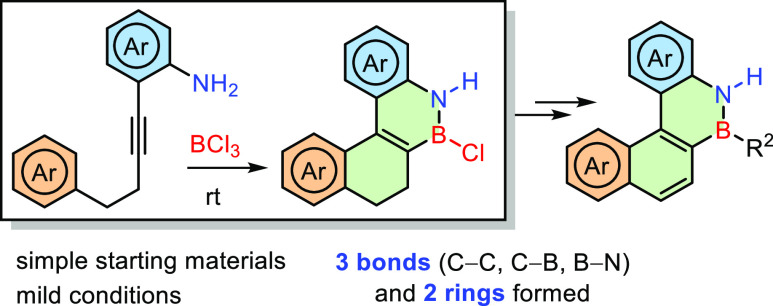

Reaction of BCl_3_ with suitably substituted *o*-alkynylanilines promotes a cascade reaction in which BN-polycyclic compounds are obtained via the formation of two new cycles and three new bonds in a single operational step. The reaction is highly efficient and takes place at room temperature, providing a very mild and straightforward strategy for the preparation of BN-aromatic compounds, which can be further transformed into a variety of BN-PAHs with different polycyclic cores and substituents.

BN-arenes have attracted great interest in recent years due to their unique properties, which have prompted applications in different fields.^1^ The formal substitution of a C=C double bond in an aromatic compound by a B–N bond results in a polarization that significantly affects the electronic properties, although the planar structure and aromaticity are maintained.^2^ Whereas this C=C/B–N isosterism has been exploited in medicinal chemistry^3^ and in the design of ligands for catalysis,^4^ the major interest lies in the field of materials science. Thus, BN-polycyclic aromatic hydrocarbons (BN-PAHs) have been applied in the design of novel optoelectronic materials such as OFETs or OLEDs.^5^ Further progress in the promising area of BN-arenes requires synthetic methods that allow the efficient production of these compounds on a large scale. To date, the most widely used strategy for the preparation of polycyclic BN-arenes is the classical electrophilic borylation, which requires high temperatures.^6,7^ As such, the development of mild and efficient approaches for the synthesis of polycyclic BN-aromatic compounds is highly desirable.^8^

Borylative cyclizations of alkynes are useful tools for the straightforward construction of borylated carbo- and heterocycles.^9^ Although such transformations have traditionally required metal catalysts, in the past few years metal-free borylative cyclizations, in which the boron compound itself acts as activator of the triple bond, have been developed. Thus, Ingleson reported a borylative cyclization of alkynes, with an aromatic ring acting as an internal nucleophile, to yield dihydronaphthalenes containing a C(sp^2^)–B bond (Scheme 1a).^10^ This method has also been used to synthesize boron-doped PAHs via the intramolecular reaction of a suitably located aromatic ring with the B–Cl bond present in the cyclized intermediate, upon solvent exchange and AlCl_3_/2,6-dichloropyridine addition.^11,12^

**Scheme 1:**
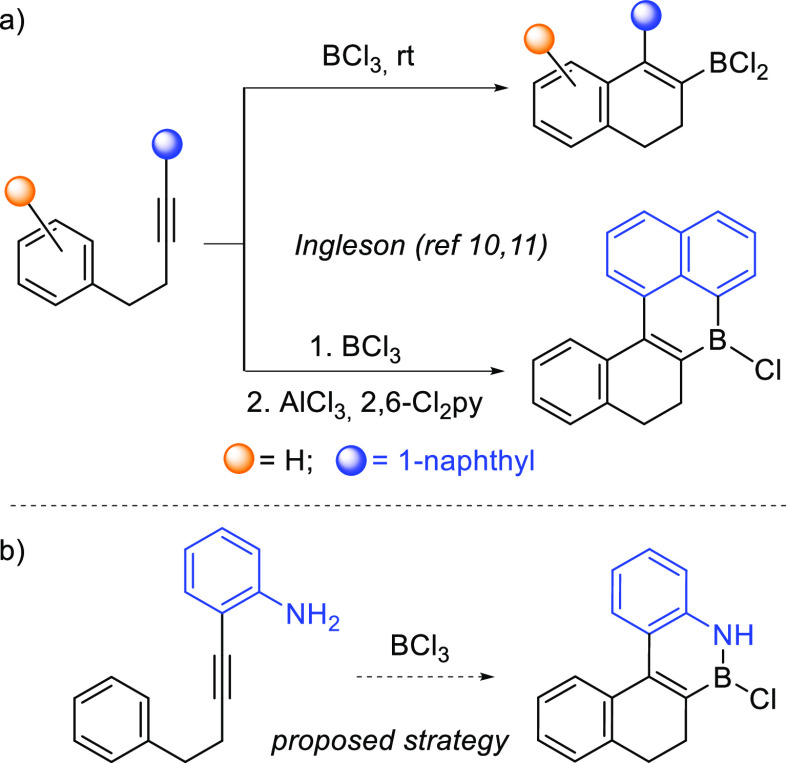
Borylative Carbocyclizations of Alkynes

Due to our interest in the synthesis of polycyclic BN-arenes^13^ and our experience in metal-free borylative cyclizations,^14^ and based on the seminal work by Ingleson, we envisioned that the location of an amino group in a suitable position could favor the concomitant formation of a B–N bond and therefore provide a straightforward access to polycyclic BN-arenes (Scheme 1b).

The required starting materials would be appropriately functionalized *o*-alkynylanilines. It is worth noting that simple *o*-alkynylanilines have been reported to react in the presence of BCl_3_ via different pathways. Thus, haloborylation of the triple bond occurs for *N,N*-dimethyl-2-(phenylethynyl) aniline,^15^ while borylated indoles are obtained via an aminoborylation when the amino moiety is a sulfonamide that acts as a nucleophile.^16^ BN-naphthalenes have also been prepared by treating *o*-alkynylanilines not substituted at the nitrogen with PhBCl_2_.^17^ In this approach, a chlorine atom is incorporated as a nucleophile in the cyclization event. Although this strategy has found useful applications,^18^ it is somewhat limited because of the very high temperatures required. Despite these precedents, we considered that the presence of a suitably located aromatic ring as a potential internal nucleophile would facilitate the proposed route by inhibiting the other possible reported reaction pathways.

To test our hypothesis, we selected 2-(4-phenylbut-1-yn-1-yl)aniline (**1a**) as a model substrate, which is easily obtained in one step from commercially available materials, and tested its reaction with 1 equiv of BCl_3_ in dichloromethane at room temperature (Scheme 2). Under these conditions, only coordination of BCl_3_ to the amine was observed, yielding compound **2a** in which the triple bond remained unreacted. The ^11^B NMR shift (6.5 ppm) clearly indicates that a dative bond has been formed, and no loss of HCl to form a covalent N–B bond occurs under these mild conditions, as also corroborated by the presence of a NH_2_ group in the ^1^H NMR spectrum. However, when 2 equiv of BCl_3_ were used, the reaction afforded exclusively dihydro BN-benzo[*c*]phenanthrene **3a** as a result of the planned borylative cyclization. No products resulting from any of the previously reported reactions of *o*-alkynylanilines with BCl_3_ were observed. The transformation of starting material **1a** into **3a** implies the formation of three new bonds (C–C, C–B, and B–N) and two new rings in a single step, which occurs under very mild conditions and without the need for any external additive.

**Scheme 2:**
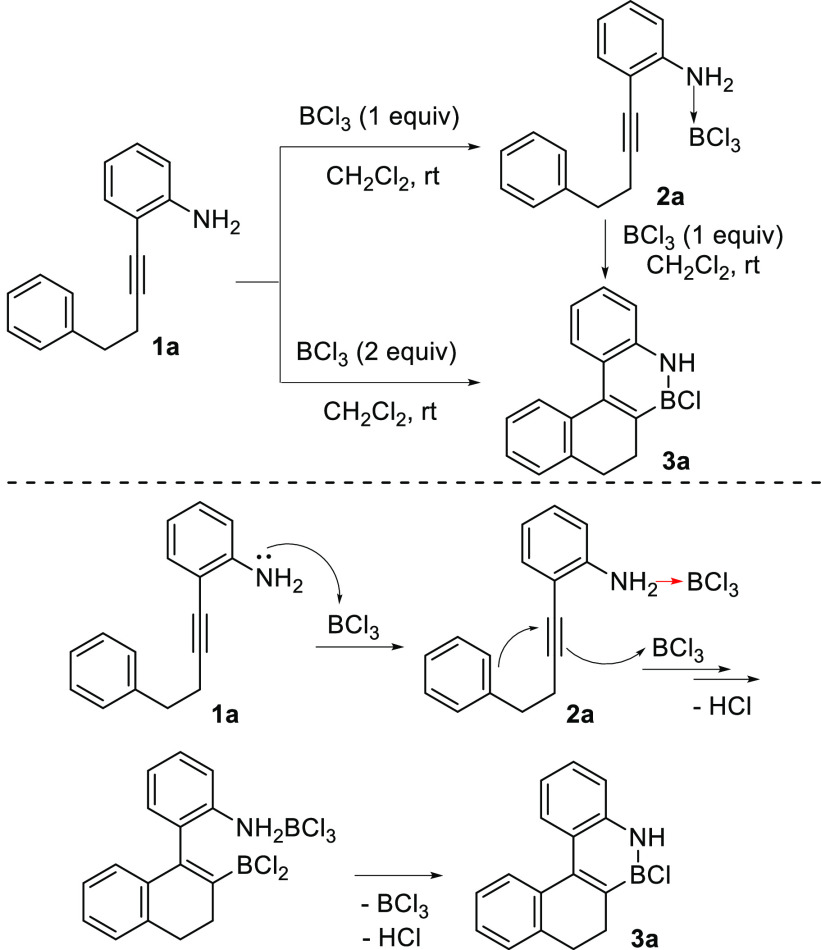
Preliminary Results and Proposed Mechanism

Moreover, we observed that compound **2a** can be transformed into dihydro BN-benzo[*c*]phenanthrene **3a** by addition of 1 equiv of BCl_3_. Based on all these observations, we propose the tentative mechanism shown in Scheme 2 for the conversion of *o*-alkynylaniline **1a** into polycyclic BN-arene **3a**. Thus, coordination of BCl_3_ to the amine moiety occurs initially, and then a second molecule of BCl_3_ would activate the triple bond, triggering the nucleophilic addition of the aromatic ring.^10^ Finally, the BCl_3_ initially coordinated to the amino group would be released, and an intramolecular B–N bond would be formed leading, after loss of HCl, to the final product **3a**. Intramolecular activation of the alkyne by the boron atom initially coordinated to the amino group, after BCl_3_ facilitated chloride abstraction,^19^ cannot be completely ruled out. However, the formation of the required borenium cation would presumably require a significantly high energy,^7^ and the fact that cyclization occurs at room temperature without any external additive makes the proposed mechanism more feasible.^20^

Noteworthily, clean NMR spectra of polycyclic BN-arene **3a** were obtained after evaporation of the solvent from the reaction mixture under an inert atmosphere, with no further purification. As expected, **3a** is not stable toward air and humidity due to the presence of a B–Cl bond. Nevertheless, this bond can be easily functionalized^21^ to yield stable polycyclic BN-arene **3b**, which can be isolated in high yield, highlighting the remarkable efficiency of the reported cascade process (Scheme 3). Furthermore, addition of a Grignard reagent after the borylative cyclization step of **1a** yields dihydro BN-benzo[*c*]phenanthrenes **3c**–**f**, which can be isolated in moderate to good yields (Scheme 3). Both aromatic and aliphatic organomagnesium compounds are suitable reagents for this transformation, with aromatic compounds providing slightly higher yields. The scope of the novel cascade borylative cyclization was explored using this one-pot cyclization–Grignard addition strategy.

**Scheme 3:**
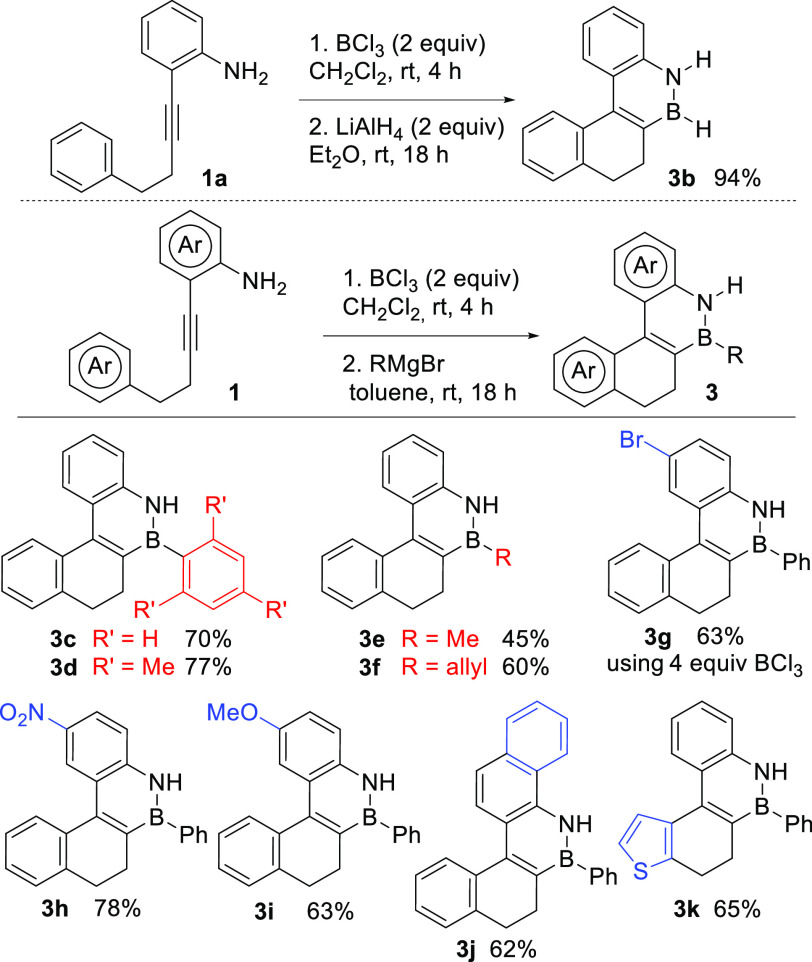
Scope of the Borylative Cyclization Cascade

*o*-Alkynylanilines with both electron-withdrawing (**1g**,**h**) and electron-donating groups (**1i**) were efficiently cyclized to yield the corresponding BN-arenes **3**. Interestingly, compound **3g** contains a C–Br bond as a suitable handle for further functionalization. BN-PAH **3j**, with extended conjugation, was also synthesized in good yield by borylative cyclization of the corresponding *o*-alkynylnaphthylamine. Thiophene-containing polycyclic BN-arenes can also be prepared using this methodology, as illustrated in the synthesis of **3k**. These borylative cyclization cascade reactions were typically performed at 0.25–0.5 mmol scale, but a similar yield was obtained when the cyclization of **1a** was performed at 1 mmol scale (65% for **3c**). Interestingly, no haloborylation or indole formation was observed for any of the substrates examined. To further demonstrate the usefulness of the developed methodology for the synthesis of complex BN-aromatic compounds from relatively simple starting materials in a single step, we envisioned that heptacyclic BN-PAHs containing two B–N bonds could be prepared if a double borylative cascade cyclization could be achieved using suitable bis(*o*-alkynylanilines) as starting materials. To this end, we synthesized **4a** and **4b** and tested their reaction with BCl_3_. After some optimization, we were able to obtain **5** in high yield through cascade borylative cyclization of **4a** (Scheme 4). A higher temperature was required for this process due to the low solubility of **4a** in the reaction medium at room temperature. The high yield obtained in this reaction is remarkable considering that it accounts for two consecutive reactions, with the overall formation of two C–C bonds, two B–N bonds, four C–B bonds, and four new cycles. However, addition of BCl_3_ to **4b**, which bears the two amino groups in relative *ortho*-positions, resulted in the formation of BN-indole **6** in excellent yield,^22^ which can be attributed to the favorable chelation of the boron atom with the two amine units preventing the coordination of the alkyne necessary for the cascade reaction to proceed (Scheme 4).

**Scheme 4:**
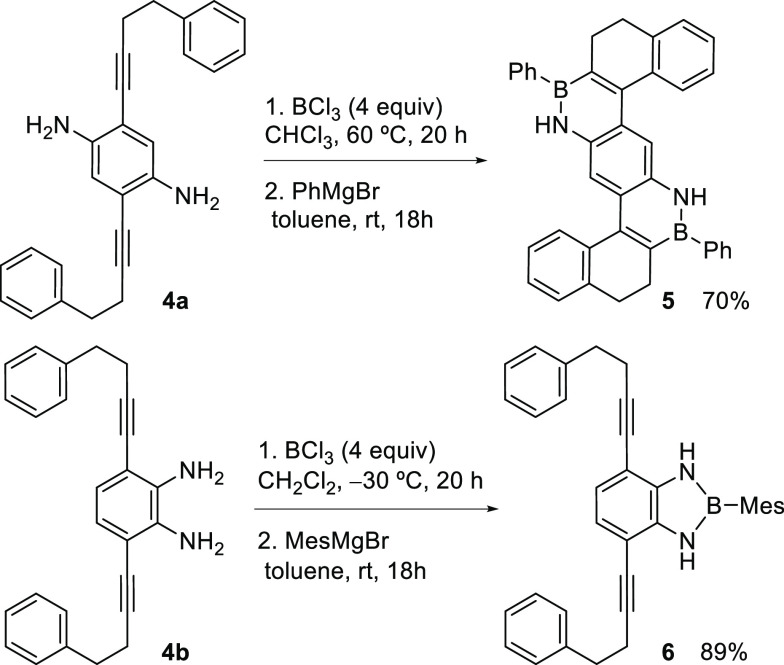
Synthesis of Bis-BN-PAH **5** and BN-Indole **6**

The polycyclic compounds obtained by the reported borylative cyclization cascade can be oxidized to obtain fully aromatic systems, as illustrated for selected representative examples in Scheme 5. After some experimentation, we found that [Ph_3_C][BF_4_] is the most efficient reagent for this transformation. Under the optimized conditions, BN-benzo[*c*]phenanthrenes **7c**(23) and **7g**, benzo[*c*]chrysene **7j**, and phenanthro[3,4-*b*]thiophene **7k** were obtained in moderate to good yields. Notably, **7j** and **7k** represent previously unknown BN-PAH structures.

**Scheme 5:**
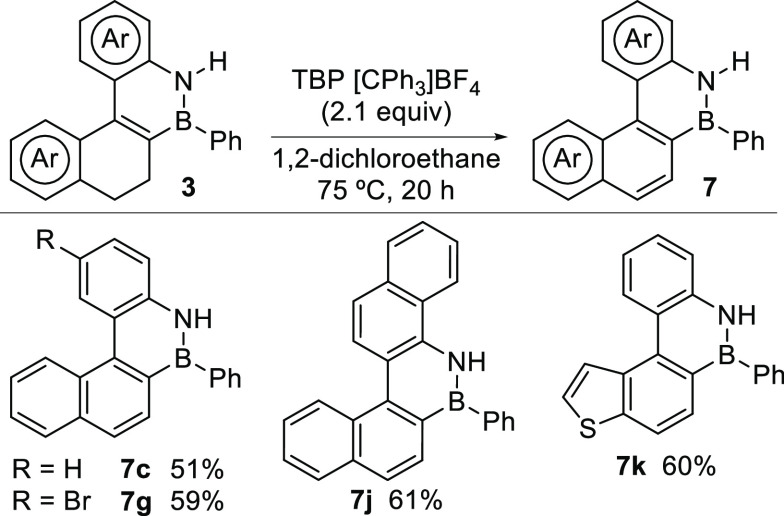
Oxidation of **3** to Fully Aromatic Compounds **7** TBP = 2,4,6-tri-*tert*-butylpyridine.

Once the scope of the borylative cyclization cascade had been established, and the oxidation to obtain fully aromatic compounds achieved, we turned our attention to the possibility of postfunctionalization of BN-benzo[*c*]phenanthrene **7c**.^24^ Thus, when **7c** was treated with an excess of bromine, dibrominated compound **7l** was obtained in good yield (Scheme 6). The crystal structure of **7l** was determined by X-ray diffraction analysis, which confirmed the positions in which the bromine atoms had been incorporated. The B–N bond length is in the range of previously reported BN-aromatic compounds, and the molecule shows a twisted conformation, with an angle between the external rings of 37.8°, which is significantly higher than that reported for benzo[*c*]phenanthrene (26.7°),^25^ but slightly lower than that reported for a BN-benzo[*c*]phenanthrene with B and N in bridgehead positions (38.9°).^13b^

**Scheme 6:**
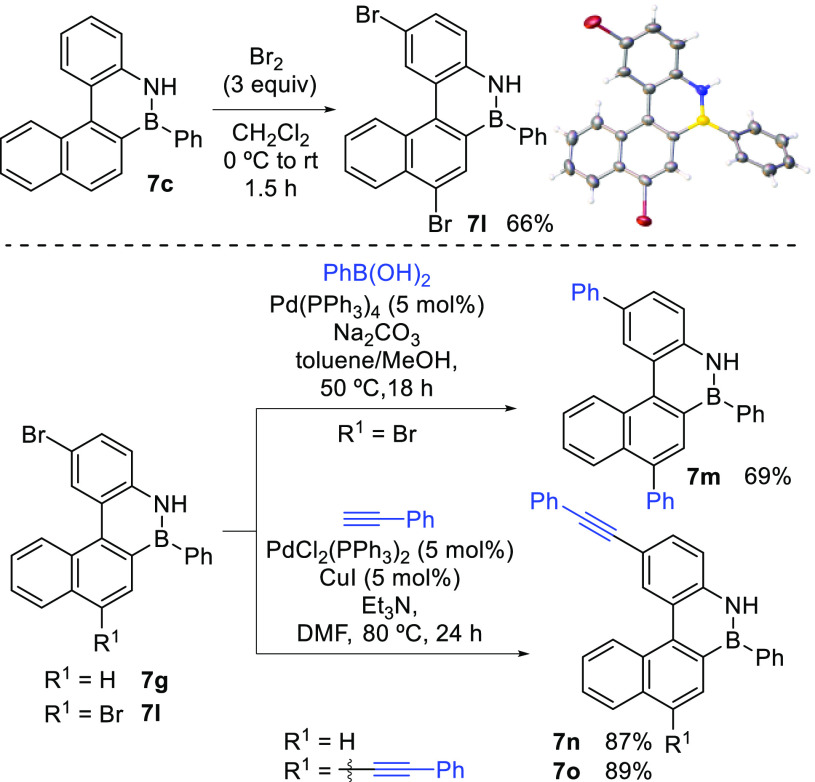
Functionalization of BN-Benzo[*c*]phenanthrenes X-ray structure ellipsoids are drawn at 50% probability level.

The presence of C(sp^2^)–Br bonds offers the possibility of easy further functionalization via cross-coupling reactions, and thus modulation of the optical properties of the system. Thus, aryl- and alkynyl-difunctionalized BN-PAHs **7m** and **7o** were easily obtained by Suzuki and Sonogashira couplings of dibrominated compound **7l**, respectively (Scheme 6). Monoalkynyl substituted BN-benzo[*c*]phenanthrene **7n** was also synthesized using monobrominated compound **7g**, directly obtained by cyclization of Br-substituted substrate **1g** and subsequent oxidation.

Moreover, preliminary results demonstrate that an analogous cascade cyclization can enable an easy access to polycyclic BN-heteroarenes (Scheme 7). The strategy is similar to the one proposed above, but the nucleophilic attack on the boron-activated triple bond is effected in this case by a suitable located heteroatomic nucleophile,^26^ instead of an aryl ring, thus leading to the formation of an heterocyclic ring. Consequently, *o*-alkynylaniline **8** was successfully cyclized in the presence of BCl_3_ upon heating to 50 °C, leading to thiophene containing BN-PAH **9** upon boron functionalization with MeMgBr. The structure of **9** was confirmed by X-ray diffraction experiments.

**Scheme 7:**
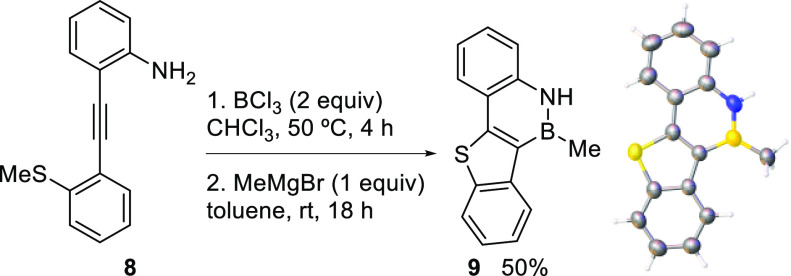
Cascade Heterocyclization/B-Functionalization X-ray structure ellipsoids are drawn at 40% probability level.

Finally, the photophysical properties of selected compounds synthesized using the reported methodology and subsequent elaborations described in this manuscript were also analyzed. The absorption and emission data in cyclohexane are summarized in Table 1. BN-benzo[*c*]phenanthrene **7c** (entry 1) shows a significantly higher quantum yield than previously reported BN-benzo[*c*]phenanthrenes with B and N atoms at the bridgehead positions.^13b^ Not surprisingly, introduction of a bromine substituent (**7g**, entry 2) results in a low quantum yield. However, the presence of phenyl or alkynyl groups (**7m,n**) leads to an increase in the quantum yield together with a bathochromic shift in the emission, when compared to **7c**. The same effect is observed for compound **7j**, which exhibits extended conjugation. The influence of the introduction of a thiophene ring in the polycyclic backbone highly depends on the position of this heterocyclic moiety. Thus, **7k** shows a low quantum yield whereas **9** is notably more fluorescent. Interestingly, derivative **5**, which contains two BN units, shows a high quantum yield^27^ for which a significant bathochromic shift in the emission is also observed.

**Table 1:** UV/vis and Fluorescence Data for Selected BN-PAHs^a^

compd	ε (M^–1^ cm^–1^)	λ_abs max_ (nm)	λ_ex_ (nm)	λ_em_ (nm)	ϕ_f_
**7c**	6068	324	324	386	0.32
**7g**	7074	367	367	390	<0.03
**7m**	2600	330	330	411	0.58
**7n**	14 967	354	352	411	0.51
**7j**	1273	305	384	411	0.47
**7k**	1705	319	319	351	0.05
**9**	4178	316	346	392	0.56
**5**	1686	352	352	414	0.91

a All experiments were performed in cyclohexane solution.

In conclusion, a mild and versatile method for the synthesis of polycyclic BN-arenes based on a borylative cyclization cascade, which allows the formation of a C–C (or a C–X), C–B, and B–N bond, and the construction of two new rings, in a single process has been described. Thus, addition of BCl_3_ to a solution of the corresponding *o*-alkynylaniline used as starting material in CH_2_Cl_2_ at room temperature provides the B–Cl derivative, which can be further functionalized, with high efficiency. Moreover, oxidation of the initially formed partially unsaturated compounds to fully aromatic ones has been achieved, as has their functionalization via bromination and subsequent cross-coupling reactions. Overall, the reported methodology provides a useful method for the synthesis of BN-aromatics, complementary to those already available, and has allowed the preparation of several previously unknown derivatives, some of which show interesting photophysical properties.

## Supporting Information Available

Experimental details, NMR spectra for all new compounds, and X-ray crystallographic data for **7l** and **9** (PDF)
