# Supplementary material for: Synthesis of BN-Polyarenes by a Mild Borylative Cyclization Cascade
Source: Org Lett. 2022 Aug 1;24(31):5860–5. doi: 10.1021/acs.orglett.2c02477 (PMC9384698; doi:10.1021/acs.orglett.2c02477)
Supplement: Supplementary file 1 — ol2c02477_si_001.pdf [file ol2c02477_si_001.pdf]

Synthesis of BN-Polyarenes by a Mild Borylative Cyclization Cascade

Ester Sans-Panadés, Juan J. Vaquero, Manuel A. Fernández-Rodríguez,* and Patricia García-García*

Universidad de Alcalá (IRYCIS). Departamento de Química Orgánica y Química Inorgánica, Instituto de Investigación Química "Andrés M. del Río" (IQAR). Campus Científico-Tecnológico, Facultad de Farmacia. Autovía A-II, Km 33.1, 28805-Alcalá de Henares, Madrid, Spain.

Electronic Supplementary Information

Table of Contents

Experimental Procedures and Data -- S2

X-ray crystallographic data for **7l** --S20

X-ray crystallographic data for **9** ---S31

Photophysical data -- S37

Copies of ^{1}H, ^{13}C, ^{10}B and ^{11}B-NMR spectra for novel compounds and selected gCOSY, TOCSY, NOESY, gHSQC and gHMBC spectra --- S41

EXPERIMENTAL PROCEDURES AND DATA

General experimental details

Reagents were acquired from commercial sources and used without further purification. Dry solvents, where necessary, were dried by a MBRAUN MB-SPS-800 apparatus. For reactions that require heating, a sand bath was used. Reactions were monitored by thin-layer chromatography (TLC) carried out on 0.25 mm E. Merck silica gel plates (60FS-254) using UV light for visualization. Column chromatography was performed using silica gel (60 F254, 70–200 mm) as the stationary phase. All melting points were determined in open capillary tubes using a Stuart Scientific SMP3 melting point apparatus (uncorrected). ^{1}H, ^{13}C, ^{11}B and ^{10}B NMR spectra were recorded on either a Varian Mercury VX-300, Varian Unity 300, Bruker Avance Neo 400 or Varian Unity 500 MHz spectrometer at room temperature. Chemical shifts are given in ppm (δ) downfield from TMS. Coupling constants (J) are in hertz (Hz) and signals are described as follows: s, singlet; d, doublet; dd, doublet of doublets; ddd, doublet of doublet of doublets; ddt, doublet of doublets of triplets; t, triplet; td, triplet of doublets, q, quadruplet; m, multiplet; b, broad. High-resolution analysis (HRMS) was performed on Agilent 6210 time of-flight LC/MS and Agilent 6545 Q-TOF.

Synthesis of starting materials

1a was synthesized according to a reported procedure.[1]

Synthesis of 5-bromo-2-(4-phenylbut-1-yn-1-yl)aniline 1g

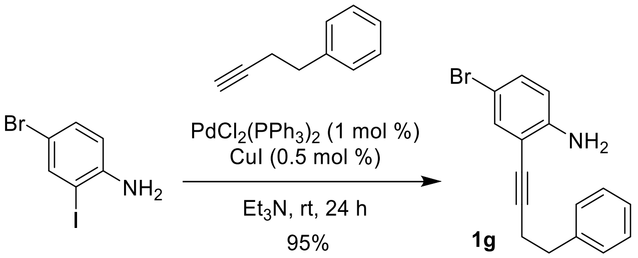

To an oven-dried Biotage microwave vial equipped with a stir bar were added $PdCl_2(PPh_3)_2$ (1.20 mg, 0.0017 mmol, 1 mol %), CuI (1.63 mg, 0.0084 mmol, 0.5 mol %) and 5-bromo-2-iodoaniline[2] (50 mg, 0.168 mmol, 1 equiv.). The vial was sealed with a cap lined with a disposable Teflon septum, evacuated under vacuum, and purged with argon five times. Triethylamine (0.32 mL) was added, and the resulting suspension was stirred at room temperature for 30 min. Then, 4-phenyl-1-butyne (36.5 µL, 0.252 mmol, 1.5 equiv.) was injected, and the mixture was stirred at room temperature for 24 h. The mixture was extracted with DCM and the combined organic layers were dried over anhydrous Na_2SO_4, filtered and concentrated under reduced pressure. The resulting residue was purified by column chromatography on silica gel (5% ethyl acetate in hexane) to give **1g** (47.9 mg, 0.16 mmol, 95%) as a yellow-orange oil.

^{1}H RMN (300 MHz, $CDCl_3$): δ (ppm) 7.39–7.19 (m, 6H), 7.17–7.10 (m, 1H), 6.53–6.46 (m, 1H), 3.97 (bs, 2H), 2.99–2.90 (m, 2H), 2.84–2.75 (m, 2H).

^{13}C RMN (75 MHz, $CDCl_3$): δ (ppm) 147.0 (C), 140.6 (C), 134.2 (CH), 131.9 (CH), 128.8 (2xCH), 128.7 (2xCH), 126.7 (CH), 115.7 (CH + C), 110.7 (C), 108.9 (C), 96.2 (C), 35.3 (CH_2), 22.0 (CH_2).

HRMS (ESI^+) calculated for $C_{16}H_{14}BrN$ $[M+H]^+$: 300.0382; found $[M+H]^+$: 300.0386.

---

[1] T. Saito, H. Nihei, T. Otani, T. Suyama, N. Furukawa, M. Saito, *Chem. Commun.*, 2008, 172-174

[2] H. Li, H. Yang, J. Petersen, K. Wang, *J. Org. Chem.* **2004**, *69*, 4500–4508.

Synthesis of 4-nitro-2-(4-phenylbut-1-yn-1-yl)aniline (1h)

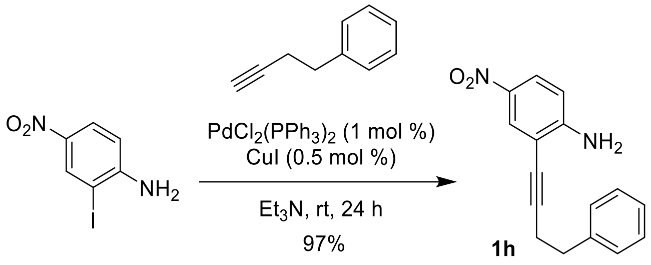

In a round bottom flask equipped with a stir bar were added $PdCl_2(PPh_3)_2$ (26.6 mg, 0.04 mmol, 1 mol %), CuI (36.1 mg, 0.19 mmol, 5 mol%) and 2-iodo-4-nitroaniline (1 g, 3.79 mmol, 1 equiv.). The flask was evacuated under vacuum and purged with argon. Triethylamine (7.0 mL) was added, and the resulting suspension was stirred at room temperature for 30 min. Then, 4-phenyl-1-butyne (0.80 mL, 5.68 mmol, 1.5 equiv.) was injected, and the mixture was stirred for 24 h at room temperature. The mixture was concentrated under reduced pressure. The remaining residue was purified by flash column chromatography (10% ethyl acetate in hexane) to provide the corresponding product **1h** (0.86 g, 3.7 mmol, 97%) as a yellow solid. M. p.: 103-104 ºC.

^{1}H-NMR (500 MHz, $CDCl_3$) δ (ppm) 8.13–8.07 (m, 1H), 7.99–7.87 (m, 1H), 7.42–7.20 (m, 5H), 6.62–6.51 (m, 1H), 4.87 (bs, 2H), 2.95 (t, *J* = 6.8 Hz, 2H), 2.82 (t, *J* = 6.8 Hz, 2H).

^{13}C-NMR (125 MHz, $CDCl_3$) δ (ppm) 153.8 (C), 140.5 (C), 138.0 (C), 128.8 (4XCH), 128.6 (CH), 126.8 (CH), 125.7 (CH), 112.8 (CH), 107.8 (C), 97.0 (C), 76.3 (C), 35.0 (CH_2), 21.9 (CH_2).

HRMS (ESI$^+$) calculated for $C_{16}H_{15}N_2O_2$ $[M+H]^+$: 267.1128; found $[M+H]^+$: 267.1137

Synthesis of 4-methoxy-2-(4-phenylbut-1-yn-1-yl)aniline (1i)

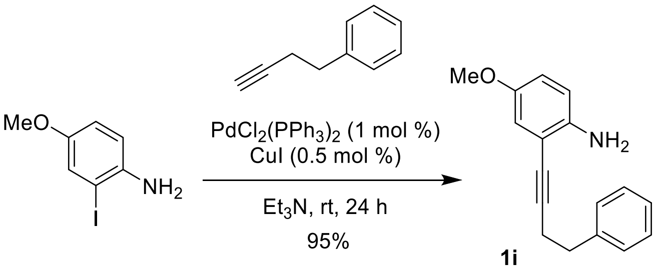

In a round bottom flask equipped with a stir bar were added $PdCl_2(PPh_3)_2$ (140 mg, 0.20 mmol, 0.5 mol %), CuI (76.0 mg, 0.4 mmol, 1 mol %) and 2-iodo-4-methoxyaniline (1 g, 4.0 mmol, 1 equiv.). The flask was evacuated under vacuum and purged with argon. Triethylamine (7.0 mL) was added, and the resulting suspension was stirred at room temperature for 30 min. Then, 4-phenyl-1-butyne (0.68 mL, 4.8 mmol, 1.2 equiv.) was injected, and the mixture was stirred for 20 h at room temperature. The mixture was concentrated under reduced pressure. The remaining residue was purified by flash column chromatography (10% ethyl acetate in hexane) to provide the corresponding product **1i** (0.96 g, 3.8 mmol, 95%) as a red oil.

^{1}H-NMR (500 MHz, $CDCl_3$) δ (ppm) 7.42 – 7.24 (m, 5H), 6.87 (d, *J* = 2.9 Hz, 1H), 6.77 (dd, *J* = 8.7, 2.9 Hz, 1H), 6.63 (d, *J* = 8.7 Hz, 1H), 3.79 (bs, 2H), 3.76 (s, 3H), 2.99 (t, *J* = 7.0 Hz, 2H), 2.85 (t, *J* = 7.0 Hz, 2H).

^{13}C-NMR (125 MHz, $CDCl_3$) δ (ppm) 152.0 (C), 142.3 (C), 140.8 (C), 128.9 (2XCH), 128.7 (2xCH), 126.7 (CH), 116.6 (CH), 116.3 (CH), 116.0 (CH), 109.6 (C), 95.1 (C), 78.6 (C), 56.1 (CH_3), 35.5 (CH_2), 22.2 (CH_2).

HRMS (ESI$^+$) calculated for $C_{17}H_{18}NO$ $[M+H]^+$: 252.1383; found $[M+H]^+$: 252.1389

Synthesis of 2-(4-phenylbut-1-yn-1-yl)naphthalen-1-amine (1j)

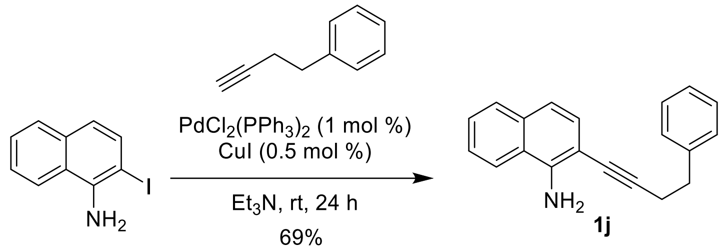

In a round bottom flask equipped with a stir bar were added $PdCl_2(PPh_3)_2$ (9.4 mg, 0.013 mmol, 1 mol %), CuI (5.1 mg, 0.027 mmol, 0.5 mol %) and 2-iodonaphthalen-1-amine (72 mg, 0.27 mmol, 1 equiv.). The flask was evacuated under vacuum and purged with argon. Triethylamine (1.6 mL) was added, and the resulting suspension was stirred at room temperature for 30 min. Then, 4-phenyl-1-butyne (57 µL, 0.41 mmol, 1.5 equiv.) was injected, and the mixture was stirred for 20 h at room temperature. The mixture was concentrated under reduced pressure. The remaining residue was purified by flash column chromatography (5% ethyl acetate in hexane) to provide the corresponding product **1j** (50.6 mg, 0.19 mmol, 69%) as a purple oil.

^{1}H-NMR (500 MHz, $CDCl_3$) δ (ppm) 7.81–7.72 (m, 2H), 7.52–7.44 (m, 2H), 7.43–7.28 (m, 6H), 7.22 (d, J = 8.5, 1H), 4.61 (bs, 2H), 3.02 (t, J = 6.8 Hz, 2H), 2.91 (t, J = 6.8 Hz, 2H).

^{13}C-NMR (125 MHz, $CDCl_3$) δ (ppm) 144.5 (C), 140.7 (C), 133.7 (C), 128.7 (2xCH), 128.60 (CH), 128.57 (CH), 128.5 (2xCH), 126.5 (CH), 126.3 (CH), 125.2 (CH), 122.5 (C), 120.9 (CH), 117.9 (CH), 102.7 (C), 95.4 (C), 79.0 (C), 35.2 (CH_2), 21.9 (CH_2).

HRMS (ESI$^+$) calculated for $C_{20}H_{18}N$ $[M+H]^+$: 272.1434; found $[M+H]^+$: 272.1443.

Synthesis of 2-(4-(thiophen-2-yl)but-1-yn-1-yl)aniline (1k)

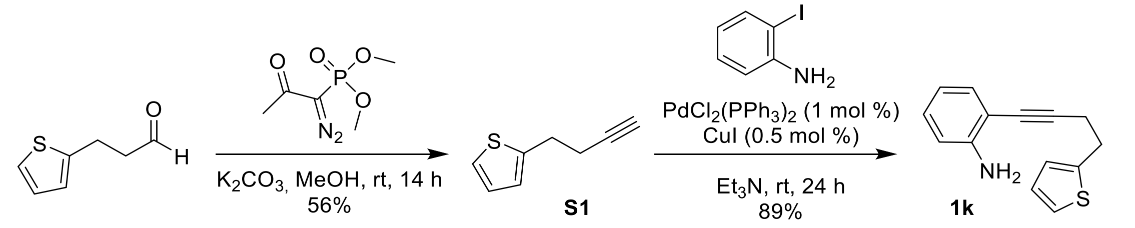

Synthesis of 2-(but-3-yn-1-yl)thiophene S1: a mixture of 3-(thiophen-2-yl)propanal[3] (236 mg, 1.7 mmol, 1 equiv.), Ohira-Bestmann reagent (391.9 mg, 2.0 mmol, 1.2 equiv.) and K_2CO_3 (467.0 mg, 3.4 mmol, 2 equiv.) in MeOH (5 mL) was stirred at room temperature for 12 h. After removal of the solvent, the residue was extracted with EtOAc. The organic layer was washed with brine, dried over Na_2SO_4, filtered and concentrated under reduced pressure. The crude was purified by column chromatography on silica gel (10% ethyl acetate in hexane) to give acetylene **S1** (129.7 mg, 0.95 mmol, 56%) as a yellow oil.

[3] C. Romano, D. Fiorito, F. Mazet, *J. Am. Chem. Soc.* **2019**, *141*, 16983–16990.

^{1}H NMR (300 MHz, CDCl$_3$) δ (ppm) 7.21–7.13 (m, 1H), 6.99–6.93 (m, 1H), 6.92–6.86 (m, 1H), 3.09 (t, *J* = 7.4 Hz, 2H), 2.56 (td, *J* = 7.4, 2.6 Hz, 2H), 2.04 (t, *J* = 2.6 Hz, 1H).

^{13}C-NMR (125 MHz, CDCl$_3$) δ (ppm) 142.9 (C), 126.8 (CH), 124.8 (CH), 123.6 (CH), 83.3 (C), 69.4 (C), 29.2 (CH$_2$), 21.1 (CH$_2$).

HRMS (APCI) calculated for C_8H_9S [M+H]$^+$: 137.0419; found [M+H]$^+$: 137.0420.

Synthesis of 2-(4-(thiophen-2-yl)but-1-yn-1-yl)aniline (1k): in a round bottom flask equipped with a stir bar were added PdCl$_2$(PPh$_3$)$_2$ (2.2 mg, 0.032 mmol), CuI (1.2 mg, 0.0063 mmol) and 2-iodoaniline (139.0 mg, 0.63 mmol). The flask was evacuated under vacuum and purged with argon five times. Triethylamine (1.6 mL) was added, and the resulting suspension was stirred at room temperature for 30 min. Then, 2-(but-3-yn-1-yl)thiophene (129.7 mg, 0.95 mmol) was injected, and the mixture was stirred for 20 h at room temperature. At the end of the reaction, the mixture was concentrated under reduced pressure. The remaining residue was purified by flash column chromatography (1% diethyl ether in pentane) to provide the corresponding product **1k** (122.7 mg, 0.56 mmol, 89%) as orange oil.

^{1}H-NMR (300 MHz, CDCl$_3$) δ (ppm) δ 7.26–7.18 (m, 1H), 7.14 (dd, *J* = 4.1, 2.4 Hz, 1H), 7.09–7.03 (t, *J* = 8.0 Hz 1H), 6.96–6.89 (m, 2H), 6.68–6.60 (m, 2H), 4.04 (bs, 2H), 3.27–2.99 (t, *J* = 7.2 Hz, 2H), 2.83 (t, *J* = 7.2 Hz, 2H).

^{13}C-NMR (125 MHz, CDCl$_3$) 147.9 (C), 143.3 (C), 132.1 (CH), 129.2 (CH), 126.9 (CH), 125.1 (CH), 123.8 (CH), 117.9 (CH), 114.3 (CH), 108.6 (C), 94.3 (C), 78.6 (C), 29.9 (CH$_2$), 22.7 (CH$_2$).

HRMS (ESI$^+$) calculated for $C_{14}H_{13}NS$ [M+H]$^+$: 227.0769 found [M+H]$^+$: 227.0777.

Synthesis of 2,5-bis(4-phenylbut-1-yn-1-yl)benzene-1,4-diamine (4a)

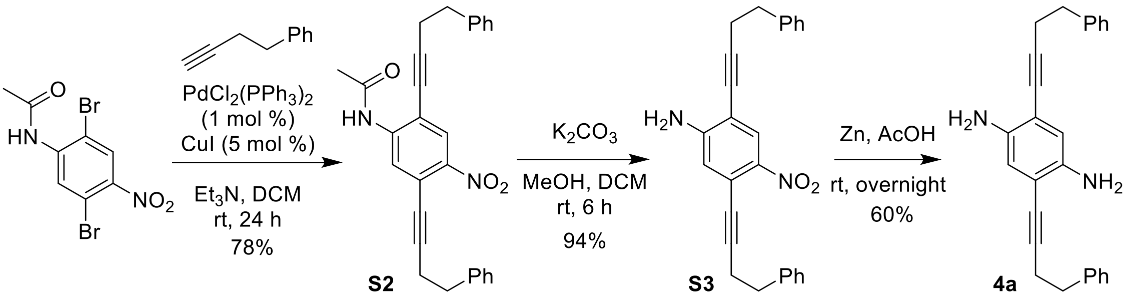

Synthesis of N-(4-nitro-2,5-bis(4-phenylbut-1-yn-1-yl)phenyl)acetamide S2: A round bottom flask equipped with a stir bar were added PdCl$_2$(PPh$_3$)$_2$ (1.0 mg, 0.074 mmol), CuI (0.6 mg, 0.03 mmol) and N-(2,5-dibromo-4-nitrophenyl)acetamide[4] (5 g, 14.8 mmol). The flask was evacuated under vacuum and purged with argon five times. Triethylamine (20 mL) and DCM (20 mL) were added, and the resulting suspension was stirred at room temperature for 30 min. Then, but-3-yn-1-ylbenzene (5 mL, 35.5 mmol) was injected, and the mixture was stirred for 20 h at room temperature. At the end of the reaction, the mixture was concentrated under reduced pressure. The remaining residue was purified by flash column chromatography (20% ethyl acetate in hexane) to provide the product **S2** (5.0 g, 11.5 mmol, 78%) as yellow solid. M. p.: 147−148 ºC.

^{1}H NMR (500 MHz, CDCl$_3$) δ (ppm) 8.64 (s, 1H), 8.03 (s, 1H), 7.73 (bs, 1H), 7.42–7.14 (m, 10H), 3.06–2.65 (m, 8H), 2.02 (s, 3H).

---

[4] Nakamura, T., Furukawa, S., Nakamura, E. *Chem. Asian J.* **2016**, *11*, 2016-2020.

^{13}C-NMR (125 MHz, $CDCl_3$) δ (ppm) 168.5 (CO), 143.9 (C), 142.1 (C), 140.4 (C), 139.8 (C), 128.7 (2xCH), 128.53 (2xCH), 128.50 (2xCH), 128.49 (2xCH), 128.2 (CH), 126.8 (CH), 126.4 (CH), 123.7 (CH), 120.2 (C), 111.6 (C), 100.2 (C), 100.0 (C), 77.3 (C), 74.9 (C), 34.6 (CH_2), 34.4 (CH_2), 24.9 (CH_3), 22.3 (CH_2), 21.5 (CH_2).

HRMS (ESI$^+$) calculated for $C_{28}H_{24}N_2NaO_3$ $[M+Na]^+$: 459.1679; found $[M+Na]^+$: 459.1688.

Synthesis of 4-nitro-2,5-bis(4-phenylbut-1-yn-1-yl)aniline S3: in a round bottom flask, N-(4-nitro-2,5-bis(4-phenylbut-1-yn-1-yl)phenyl)acetamide **S2** (5.0 g, 11.5 mmol) was dissolved in MeOH (15 mL) and DCM (10 mL). K_2CO_3 (2.4 g, 17.3 mmol) was added and the mixture was stirred for 8 h at room temperature. The mixture was washed with 10% HCl solution and the combined organic layers were washed with brine, dried over anhydrous Na_2SO_4, filtered and concentrated under reduced pressure. The resulting residue was purified by column chromatography on silica gel (20% ethyl acetate in hexane) to give compound **S3** (4.3 g, 10.8 mmol, 94%) as a yellow solid. M. p.: 146−147 ºC.

^{1}H NMR (500 MHz, $CDCl_3$) δ (ppm) 8.04 (s, 1H), 7.42–7.17 (m, 10H), 6.61 (s, 1H), 4.49 (bs, 2H), 3.02–2.89 (m, 4H), 2.86–2.71 (m, 4H).

^{13}C-NMR (125 MHz, $CDCl_3$) δ (ppm) 151.4 (C), 140.9 (C), 140.4 (C), 139.5 (C), 129.9 (CH), 128.8 (4xCH), 128.7 (2xCH), 128.6 (2xCH), 126.8 (CH), 126.5 (CH), 120.9 (C), 118.2 (CH), 107.6 (C), 98.4 (C), 97.8 (C), 78.1 (C), 76.2 (C), 35.1 (CH_2), 35.0 (CH_2), 22.5 (CH_2), 21.9 (CH_2).

HRMS (ESI$^+$) calculated for $C_{26}H_{23}N_2O_2$ $[M+H]^+$: 395.1754; found $[M+H]^+$: 395.1753.

Synthesis of 2,5-bis(4-phenylbut-1-yn-1-yl)benzene-1,4-diamine (4a): in a round bottom flask, 4-nitro-2,5-bis(4-phenylbut-1-yn-1-yl)aniline **S3** (4.3 g, 10.8 mmol, 1 equiv.) was dissolved in AcOH (30 mL). Then, zinc powder (3.5 g, 54.0 mmol, 5 equiv.) was added in portions and the resulting suspension was stirred at room temperature overnight. The reaction was quenched by addition of a saturated $NaHCO_3$ aqueous solution. The mixture was extracted with Et_2O and the combined organic layers were washed with brine, dried over anhydrous Na_2SO_4, filtered and concentrated under reduced pressure. The resulting residue was purified by column chromatography on silica gel (20% ethyl acetate in hexane) to give **4a** (2.4 g, 6.5 mmol, 60%) as a yellow solid. M. p.: 130−132 ºC.

^{1}H-NMR (500 MHz, $CDCl_3$) δ (ppm) 7.36–7.30 (m, 4H), 7.29–7.20 (m, 6H), 6.59 (s, 2H), 3.57 (bs, 4H), 2.93 (t, J = 7.3 Hz, 4H), 2.78 (t, J = 7.3 Hz, 4H).

^{13}C-NMR (125 MHz, $CDCl_3$) δ (ppm) 140.6 (2xC), 139.7 (2xC), 128.6 (4xCH), 128.5 (4xCH), 126.4 (2xCH), 117.6 (2xCH), 110.4 (2xC), 95.4 (2xC), 78.0 (2xC), 35.1 (2xCH_2), 21.8 (2xCH_2).

HRMS (ESI$^+$) calculated for $C_{26}H_{25}N_2$ $[M+H]^+$: 365.2012; found $[M+H]^+$: 365.2010.

Synthesis 3,6-bis(4-phenylbut-1-yn-1-yl)benzene-1,2-diamine (4b)

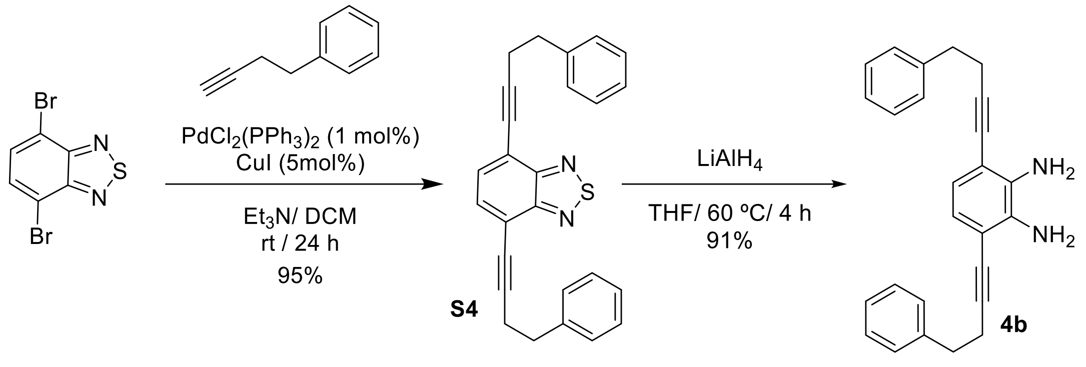

Synthesis of 4,7-bis(4-phenylbut-1-yn-1-yl)benzo[c][1,2,5]thiadiazole S4: In a round bottom flask equipped with a stir bar were added $PdCl_2(PPh_3)_2$ (30 mg, 0.034 mmol, 2 mol%), CuI (1 mg, 0.017 mmol, 1 mol%), PPh_3 (10 mg, 0.034 mmol, 2 mol%) and 4,7-dibromobenzo[c][1,2,5]thiadiazole (0.5 g, 1.7 mmol, 1 equiv). The flask was evacuated under vacuum and purged with argon five times. Triethylamine (20 mL) was added, and the resulting suspension was stirred at room temperature for 30 min. Then, but-3-yn-1-ylbenzene (0.72 mL, 5.1 mol, 3 equiv.) was injected, and the mixture was stirred for 20 h at room temperature. At the end of the reaction, the mixture was concentrated under reduced pressure. The remaining residue was purified by flash column chromatography (20% ethyl acetate in hexane) to provide the product (0.63 g, 1.6 mmol, 95%) as yellow solid. M. p.: 150-152 ºC.

^{1}H-NMR (500 MHz, $CDCl_3$) δ (ppm) 7.55 (s, 2H), 7.43–7.16 (m, 10H), 3.05 (t, *J* = 7.5 Hz, 4H), 2.90 (t, *J* = 7.3 Hz, 4H).

^{13}C-NMR (125 MHz, $CDCl_3$) δ (ppm) 154.6 (2xC), 140.5 (2xC), 132.4 (2xCH), 128.6 (4xCH), 128.5 (4xCH), 126.5 (2xCH), 117.2 (2xC), 98.0 (2xC), 77.5 (2xC), 35.0 (2xCH_2), 22.3 (2xCH_2).

HRMS (ESI$^+$) calculated for $C_{26}H_{20}N_2S$ $[M+H]^+$: 392.1347; found $[M+H]^+$: 392.1349.

Synthesis 3,6-bis(4-phenylbut-1-yn-1-yl)benzene-1,2-diamine 4b: 4,7-bis(4-phenylbut-1-yn-1-yl)benzo[c][1,2,5]thiadiazole (50mg, 0.13 mmol, 1.0 equiv.) was dissolved in anhydrous THF (1 mL) in round bottom flask equipped with a stir bar, and the resulting solution was cooled to 0 ºC. Cold lithium aluminium hydride solution 1.0 M in Et_2O (0.25 mL, 0.25 mmol, 2 equiv.) was added dropwise via syringe, and the reaction mixture was allowed to warm to room temperature and stirred for 18 hours at 60 ºC. Then, a hydrogen chloride solution 2.0 M in Et_2O (0.25 mL, 0.5 mmol 2 equiv.) was added, and the resulting mixture was filtered through a silica gel plug. The filtrate was concentrated under reduced pressure. At the end of the reaction, the mixture was concentrated under reduced pressure. The remaining residue was purified by flash column chromatography (20% ethyl acetate in hexane) to provide the product (41 mg, 0.11 mmol, 91%) as orange solid. M.p.: 132-134 ºC.

^{1}H-NMR (500 MHz, $CDCl_3$) δ (ppm) 7.42–7.16 (m, 10H), 6.72 (s, 2H), 3.64 (bs, 4H), 2.96 (t, *J* = 6.9 Hz, 4H), 2.82 (t, *J* = 7.1 Hz, 4H).

^{13}C-NMR (125 MHz, $CDCl_3$) δ (ppm) 140.6, (2xC), 136.0 (2xC), 128.6 (4xCH), 128.5 (4xCH), 126.4 (2xCH), 121.8 (2xCH), 109.7 (2xC), 95.3 (2xC), 78.3 (2xC), 35.1 (2xCH_2), 21.8 (2xCH_2).

HRMS (ESI$^+$) calculated for $C_{26}H_{25}N_2$ $[M+H]^+$: 365.2012; found $[M+H]^+$: 365.2009.

Synthesis of 2-((2-(methylthio)phenyl)ethynyl)aniline (8)

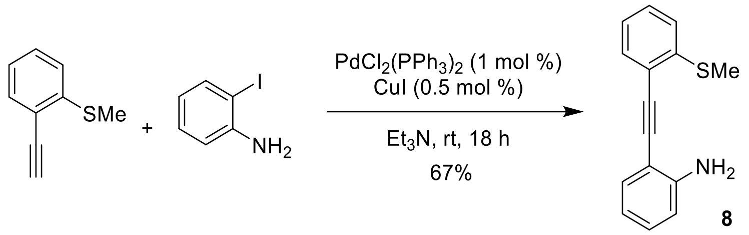

In a round bottom flask equipped with a stir bar were added $PdCl_2(PPh_3)_2$ (135.5 mg, 0.193mmol, 1 mol %), CuI (18.4 mg, 0.097 mmol, 5 mol%) and 2-iodoaniline (422.0 mg, 1.93 mmol, 1 equiv.). The flask was evacuated under vacuum and purged with argon. Triethylamine (3.5 mL) was added, and the resulting suspension was stirred at room temperature for 30 min. Then, (2-ethynylphenyl)(methyl)sulfane[5] (0.573, 2.89mmol, 1.5 equiv.) was added, and the mixture was stirred for 18 h at room temperature. The mixture was concentrated under reduced pressure. The remaining residue was purified by flash column chromatography (5% ethyl acetate in hexane) to provide the corresponding product **8** (303.0 mg, 3.7 mmol, 67%) as a light yellow solid. M. p.: 91-92ºC.

^{1}H-NMR (500 MHz, $CDCl_3$) δ (ppm) 7.56–7.47 (m, 1H), 7.45–7.36 (m, 1H), 7.35–7.24 (m, 1H), 7.23–7.09 (m, 2H), 6.79–6.68 (m, 2H), 4.49 (bs, 2H), 2.54 (s, 3H).

^{13}C-NMR (125 MHz, $CDCl_3$) δ (ppm) 148.4 (C), 140.8 (C), 132.10 (CH), 132.08 (CH), 130.1 (CH), 128.8 (CH), 124.77 (CH), 124.72 (CH), 122.0 (C), 117.9 (CH), 114.5 (CH), 107.9 (C), 93.1 (C), 92.5 (C), 15.8 (CH_3).

HRMS (ESI$^+$) calculated for $C_{15}H_{14}NS$ $[M+H]^+$: 240.0841; found $[M+H]^+$: 240.0850

Reaction of o-alkynylanilines 1 with BCl_3 and subsequent functionalization of the B-Cl bond

Reaction of 1a with 1 equivalent of BCl_3 (formation of 2a)

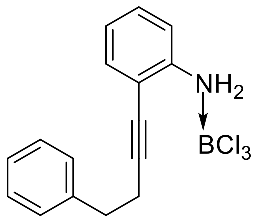

A Schlenk flask was evacuated under vacuum and purged with argon five times. The *o*-alkynylaniline **1a** (55.3 mg, 0.25 mmol, 1 equiv.) was dissolved in DCM (1 mL) in the Schlenk flask. Cold (≈5 ºC) boron trichloride 1M solution in hexanes (0.25 mL, 0.25 mmol, 1.0 equiv.) was added dropwise via syringe to the vigorously stirring solution of *o*-alkynylaniline in DCM. The reaction was stirred for 4 hours at room temperature. The resulting mixture was concentrated under reduced pressure to afford **2a** as an air- and moisture-sensitive withe foam.

^{1}H-NMR (500 MHz, $CDCl_3$) δ (ppm) 7.76–7.66 (m, 1H), 7.54–7.45 (m, 1H), 7.44–7.21 (m, 7H), 6.80 (bs, 2H), 3.00 (t, *J* = 7.3 Hz, 2H), 2.83 (t, *J* = 7.2 Hz, 2H).

^{13}C-NMR (125 MHz, $CDCl_3$) δ (ppm) 140.3 (C), 134.4 (C), 132.4 (CH), 129.0 (CH), 128.8 (2xCH), 128.7 (2xCH), 126.8 (2xCH), 123.3 (CH), 119.9 (C), 100.2 (C), 75.8 (C), 34.9 (CH_2), 22.0 (CH_2).

^{11}B-NMR (128 MHz, $CDCl_3$) δ (ppm) 6.52.

[5] Faizi, D. J.; Davis, A. J.; Meany, F. B.; Blum, S. A. *Angew. Chem., Int. Ed.* **2016**, *55*, 14286−14290.

Synthesis of 6-cloro-5,6,7,8-tetrahydro-5-aza-6-borabenzo[*c*]phenanthrene (3a)

A Schlenk flask was evacuated under vacuum and purged with argon five times. The *o*-alkynylaniline **1a** (55.3 mg, 0.25 mmol, 1 equiv.) was dissolved in DCM (1 mL) in the Schlenk flask. Cold (≈5 ºC) boron trichloride 1M solution in hexanes (0.5 mmol, 0.5 mmol, 2.0 equiv.) was added dropwise via syringe to the vigorously stirring solution of *o*-alkynylaniline in DCM. The reaction was stirred for 4 hours at room temperature. The resulting mixture was concentrated under reduced pressure to afford the corresponding B-Cl intermediate **3a** as an air- and moisture-sensitive solid, which could be characterized and used without further purification for subsequent functionalization reactions of the B-Cl bond.

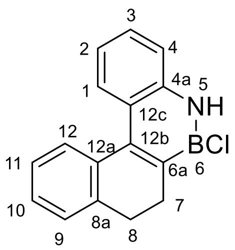

^{1}H-NMR (500 MHz, CDCl$_3$) δ (ppm) 8.27 (d, *J* = 8.2 Hz, 1H, H-1), 7.84–7.73 (m, 2H, H-12, N-H), 7.47–7.39 (m, 2H, H-3, H-9), 7.38–7.33 (m, 2H, H-10, H-11), 7,28–7.20 (2, 2H, H-2, H-4), 2.75 (s, 4H, H-7, H-8).

^{13}C-NMR (125 MHz, CDCl$_3$) δ (ppm) 147.7 (C, C-4a), 141.1 (C, C-8a), 139.8 (C, C-12b), 136.4 (C, C-6a)*, 133.2 (C, C-12a), 128.3 (CH, C-12), 128.2 (CH, C-1), 128.1 (CH, C-9), 128.0 (CH, C-10), 127.9 (CH, C-3), 125.9 (CH, C-11), 122.5 (C, C-12c), 121.3 (CH, C-2), 118.5 (CH, C-4), 29.0 (CH$_2$, C-8), 26.1 (CH$_2$, C-7). *Carbon not observed in ^{13}C-NMR, assigned by gHMBC.

^{11}B-NMR (128 MHz, CDCl$_3$) δ (ppm) 33.18.

Synthesis of 5,6,7,8-tetrahydro-5-aza-6-borabenzo[*c*]phenanthrene (3b)

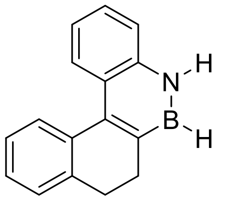

The corresponding B-Cl intermediate **3a**, from the reaction of 55.3 mg (0.25 mmol) of **1a,** was dissolved in anhydrous Et$_2$O (2 mL) in a Schlenk flask under argon atmosphere, and the resulting solution was cooled to 0 ºC. Cold (≈5 ºC) lithium aluminium hydride solution 1.0 M in Et$_2$O (0.5 mL, 0.5 mmol) was added dropwise via syringe, and the reaction mixture was allowed to warm to room temperature and stirred for 18 hours. Then, a hydrogen chloride solution (2.0 M in Et$_2$O, 2.2 equiv.) was added, and the resulting mixture was filtered through a silica gel plug. The filtrate was concentrated under reduced pressure to afford the corresponding product **3b** (54.3 mg, 0.24 mmol, 94%) as a white solid. M. p.: 95−97 ºC.

^{1}H-NMR (300 MHz, CDCl$_3$) δ (ppm) 8.35 (d, *J* = 8.2 Hz, 1H), 8.11 (bs, 1H), 7.85 (d, *J* = 7.8 Hz, 1H), 7.54–7.30 (m, 5H), 7.29–7.19 (m, 1H), 5.08 (bs, 1H), 2.93–2.63 (m, 4H).

^{13}C-NMR (125 MHz, CDCl$_3$) δ (ppm) 144.7 (C), 141.0 (C), 140.2 (C), 133.1 (C), 128.0 (CH), 127.6 (CH), 127.4 (2xCH), 126.8 (CH), 125.6 (CH), 122.9 (C), 120.8 (CH), 118.8 (CH), 29.3 (CH$_2$), 29.1 (CH$_2$). The carbon atoms bonded to the boron atom are not observed.

^{11}B-NMR (128 MHz, CDCl$_3$) δ (ppm) 31.67.

HRMS: The compound decomposes during the measurement.

<u>General procedure for the synthesis of 3 and 5 by borylative cyclization/Grignard addition</u>

In a Schlenk flask, evacuated under vacuum and purged with argon five times, the corresponding *o*-alkynylaniline **1** (1 equiv.) was dissolved in anhydrous DCM (0.25 M). Cold (≈5 ºC) boron trichloride solution (1.0 M in hexanes, 2.0 equiv.) was added dropwise via syringe to the

vigorously stirring solution of *o*-alkynylaniline in DCM and the reaction was stirred at room temperature until completion. The resulting mixture was concentrated under reduced pressure to afford the corresponding B-Cl intermediate, which was redissolved in toluene. The resulting solution was treated with the corresponding organomagnesium compound (1.0 M in THF, 1 equiv.) and the reaction mixture was stirred for 18 h. The remaining Grignard was quenched with a 2-propanol:toluene solution (2:8) and after 10 min the resulting mixture was filtered through a Celite plug. The filtrate was concentrated under reduced pressure and the crude product was further purified via silica gel column chromatography (hexane:ethyl acetate 95:5).

Synthesis of 6-phenyl-5,6,7,8-tetrahydro-5-aza-6-borabenzo[*c*]phenanthrene (3c)

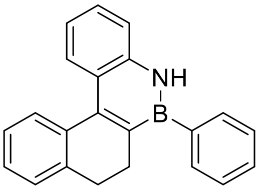

Following the general procedure to produce **3a** using anyline **1a** (221.1 mg, 1.0 mmol, 1 equiv.) and BCl_3 (2.0 mL, 2.0 mmol, 2.0 equiv.). The B-Cl intermediate, obtained after 4 h, was then treated with phenylmagnesium bromide solution (1.0 mL, 1.0 mmol, 1 equiv.) to give **3c** (200.9 mg, 0.65 mmol, 65%) as a white solid. M. p.: 112−113 ºC.

^{1}H NMR (500 MHz, $CDCl_3$) δ (ppm) 8.34 (d, *J* = 8.2 Hz, 1H), 7.95 (bs, 1H), 7.86 (dd, *J* = 7.3, 1.5 Hz, 1H), 7.78–7.72 (m, 2H), 7.55–7.45 (m, 3H), 7.44–7.34 (m, 4H), 7.24 (ddd, *J* = 8.3, 7.0, 1.3 Hz, 1H), 2.89–2.81 (m, 2H), 2.75–2.70 (m, 2H).

^{13}C-NMR (125 MHz, $CDCl_3$) δ (ppm) 146.4 (C), 141.1 (C), 140.5 (C), 133.8 (C), 133.0 (2xCH), 128.7 (CH), 128.4 (CH), 128.1 (2xCH), 127.9 (CH), 127.74 (CH), 127.70 (CH), 127.4 (CH), 125.8 (CH), 123.0 (C), 120.9 (CH), 118.9 (CH), 29.4 (CH_2), 28.0 (CH_2). The carbon atoms bonded to the boron atom are not observed.

^{11}B-NMR (128 MHz, $CDCl_3$) δ (ppm) 34.28.

HRMS (APCI) calculated for $C_{22}H_{19}BN$ $[M+H]^+$: 308.1609; found $[M+H]^+$: 308.1608.

Synthesis of 6-mesityl-5,6,7,8-tetrahydro-5-aza-6-borabenzo[*c*]phenanthrene (3d)

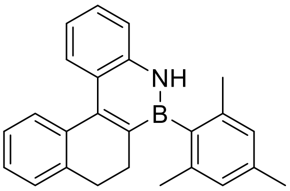

Following the general procedure to produce **3a** using aniline **1a** (132.8 mg, 0.50 mmol, 1 equiv.) and BCl_3 (1.0 mL, 1.0 mmol, 2.0 equiv.). The B-Cl intermediate, obtained after 4 h, was then treated with mesitylmagnesium bromide solution (1.0 mL, 1.0 mmol, 2 equiv.) to give **3d** (134,5 mg, 0,39 mmol, 77%) as an orange oil.

^{1}H NMR (500 MHz, $CDCl_3$) δ (ppm) 8.34 (d, *J* = 8.2 Hz, 1H), 7.90–7.86 (m, 1H), 7.79 (bs, 1H), 7.43 (ddd, *J* = 8.3, 7.1, 1.4 Hz, 1H), 7.39–7.27 (m, 4H), 7.22 (ddd, *J* = 8.3, 6.9, 1.3 Hz, 1H), 6.92 (s, 2H), 2.69–2.64 (m, 2H), 2.46–2.40 (m, 2H), 2.36 (s, 3H), 2.17 (s, 6H).

^{13}C-NMR (125 MHz, $CDCl_3$) δ (ppm) 145.1 (C), 141.1 (C), 140.5 (C), 140.0 (2xC), 137.4 (C), 133.6 (C), 128.3 (CH), 127.7 (CH), 127.6 (CH), 127.5 (CH), 127.1 (2xCH), 127.0 (CH), 125.6 (CH), 122.9 (C), 120.7 (CH), 118.8 (CH), 29.2 (CH_2), 27.4 (CH_2), 22.6 ($2xCH_3$), 21.2 (CH_3). The carbon atoms bonded to the boron atom are not observed.

^{11}B-NMR (128 MHz, $CDCl_3$) δ (ppm) 36.00.

HRMS (APCI) calculated for $C_{25}H_{25}BN$ $[M+H]^+$: 350.2079; found $[M+H]^+$: 350.2074.

Synthesis of 6-methyl-5,6,7,8-tetrahydro-5-aza-6-borabenzo[*c*]phenanthrene (3e)

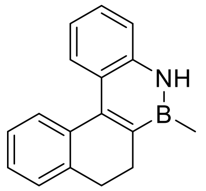

Following the general procedure to produce **3a** using anyline **1a** (66.4 mg, 0.25 mmol, 1 equiv.) and BCl_3 (0.5 mL, 0.5 mmol, 2.0 equiv.). The B-Cl intermediate, obtained after 4 h, was treated with methylmagnesium bromide solution (0.50 mL, 0.50 mmol, 2 equiv.) to give **3e** (27.6mg, 0.11 mmol, 45%) as a white oil.

^{1}H NMR (500 MHz, CDCl$_3$) δ (ppm) 8.25 (d, *J* = 8.2Hz, 1H), 7.81 (dd, *J* = 7.5, 1.6 Hz, 1H), 7.74 (bs, 1H), 7.41–7.29 (m, 4H), 7.26 (dd, *J* = 8.0, 1.3 Hz, 1H), 7.16 (ddd, *J* = 8.3, 7.1, 1.3 Hz, 1H), 2.77–2.71 (m, 2H), 2.70–2.63 (m, 2H), 0.81 (s, 3H).

^{13}C-NMR (125 MHz, CDCl$_3$) δ (ppm) 143.8 (C), 140.8 (C), 140.4 (C), 133.7 (C), 128.1 (CH), 127.7 (CH), 127.6 (CH), 127.3 (CH), 126.9 (CH), 125.6 (CH), 122.6 (C), 120.2 (CH), 118.3 (CH), 29.1 (CH_2), 27.0 (CH_2). The carbon atoms bonded to the boron atom are not observed.

^{11}B-NMR (128 MHz, CDCl$_3$) δ (ppm) 37.08.

HRMS (ESI$^+$) calculated for $C_{17}H_{16}BN$ $[M+H]^+$: 246.1449; found $[M+H]^+$: 246.1450.

Synthesis of 6-allyl-5,6,7,8-tetrahydro-5-aza-6-borabenzo[*c*]phenanthrene (3f)

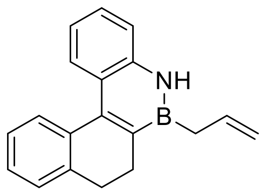

Following the general procedure to produce **3a** using anyline **1a** (66.4 mg, 0.25 mmol, 1 equiv.) and BCl_3 (0.5 mL, 0.5 mmol, 2.0 equiv.). The B-Cl intermediate, obtained after 4 h, was treated with allylmagnesium bromide solution (0.50 mL, 0.50 mmol, 2 equiv.) to give **3f** (40.7 mg, 0,15 mmol, 60%) as a yellow oil.

^{1}H NMR (300 MHz, CDCl$_3$) δ (ppm) 8.25 (d, *J* = 8.2 Hz, 1H), 7.84–7.75 (m, 1H), 7.72 (bs, 1H), 7.45–7.25 (m, 5H), 7.21–7.11 (m, 1H), 6.11 (ddt, *J* = 17.6, 10.2, 7.7 Hz, 1H), 5.21–5.05 (m, 2H), 2.78-2.69 (m, 2H), 2.68-2.58 (m, 2H), 2.33 (d, *J* = 7.6 Hz, 2H).

^{13}C-NMR (125 MHz, CDCl$_3$) δ (ppm) 144.6 (C), 140.9 (C), 140.4 (C), 137.1 (CH), 133.7 (C), 128.3 (CH), 127.8 (CH), 127.7 (CH), 127.6 (CH), 127.1 (CH), 125.8 (CH), 122.9 (C), 120.6 (CH), 118.7 (CH), 115.2 (CH_2), 29.4 (CH_2), 26.8 (CH_2). The carbon atoms bonded to the boron atom are not observed.

^{11}B-NMR (128 MHz, CDCl$_3$) δ (ppm) 35.49.

HRMS (APCI) calculated for $C_{19}H_{19}BN$ $[M+H]^+$: 272.1609; found $[M+H]^+$: 272.1601.

Synthesis of 2-bromo-6-phenyl-5,6,7,8-tetrahydro-5-aza-6-borabenzo[*c*]phenanthrene (3g)

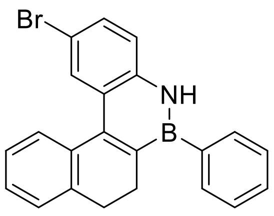

Compound **1h** (171.2 mg, 0.50 mmol, 1 equiv) was mixed with cold (≈5 ºC) boron trichloride solution (2.0 mL, 2.0 mmol, 4.0 equiv.) and the reaction was stirred for 18 hours at room temperature. The B-Cl intermediate formed was treated with phenylmagnesium bromide solution (1.0 mL, 1.0 mmol, 2 equiv.) to give **3g** (122.2 mg, 0,32 mmol, 63%) as a white solid. M.p.: 111–113 ºC.

^{1}H-NMR (500 MHz, CDCl$_3$) δ (ppm) 8.43 (d, *J* = 2.2 Hz, 1H), 7.90 (bs, 1H), 7.77 (d, *J* = 7.5 Hz, 1H), 7.72–7.67 (m, 2H), 7.50–7.33 (m, 7H), 7.23 (dd, *J* = 8.6 Hz, 1H), 2.86–2.77 (m, 2H), 2.72–2.65 (m, 2H).

13C-NMR (125 MHz, CDCl3) δ (ppm) 145.4 (C), 140.9 (C), 139.1 (C), 133.1 (C), 132.8 (2xCH), 130.1 (CH), 130.0 (CH), 128.8 (CH), 128.1 (2xCH), 127.93 (CH), 127.90 (CH), 127.7 (CH), 126.0 (CH), 124.4 (C), 120.3 (CH), 113.6 (C), 29.1 (CH_2), 27.9 (CH_2). The carbon atoms bonded to the boron atom are not observed.

11B-NMR (128 MHz, CDCl3) δ (ppm) 34.44.

HRMS (APCI) calculated for $C_{22}H_{18}BBrN$ $[M+H]^+$: 386.0714; found $[M+H]^+$: 386.0716.

Synthesis of 2-nitro -6-phenyl-5,6,7,8-tetrahydro-5-aza-6-borabenzo[*c*]phenanthrene (3h)

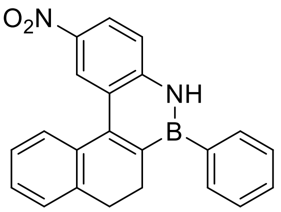

Following the general procedure using compound **1h** (66.6 mg, 0.25 mmol, 1 equiv) and cold (≈5 ºC) boron trichloride solution (0.5 mL, 0.5 mmol, 2.0 equiv.) and the reaction was stirred for 4 hours at room temperature. The B-Cl intermediate formed was treated with phenylmagnesium bromide solution (0.25 mL, 0.25 mmol, 1 equiv.) to give **3h** (68.7 mg, 0,20 mmol, 78%) as a white solid. M.p.: 123−124 ºC.

1H-NMR (500 MHz, CDCl3) δ (ppm) 8.96 (d, *J* = 2.5 Hz, 1H), 8.01 (dd, *J* = 8.9, 2.5 Hz, 1H), 7.89 (bs, 1H), 7.48–7.41 (m, 3H), 7.25–7.20 (m, 3H), 7.19–7.09 (m, 4H), 2.57 (t, *J* = 6.9 Hz, 2H), 2.44 (t, *J* = 6.9 Hz, 2H).

13C-NMR (125 MHz, CDCl3) δ (ppm) 146.7 (C), 144.6 (C), 141.8 (C), 140.8 (C), 132.8 (2XCH), 132.6(C), 129.3 (CH), 128.5 (CH), 128.2 (2XCH), 127.93 (CH), 127.92 (CH), 126.4 (CH), 124.6 (CH), 122.3 (C), 122.1 (CH), 119.3 (CH), 28.9 (CH_2), 27.7 (CH_2). The carbon atoms bonded to the boron atom are not observed.

11B-NMR (128 MHz, CDCl3) δ (ppm) 35.55.

HRMS (APCI) calculated for $C_{22}H_{17}BN_2O_2$ $[M+H]^+$: 352.1383; found $[M+H]^+$: 352.1387.

Synthesis of 2-methoxy -6-phenyl-5,6,7,8-tetrahydro-5-aza-6-borabenzo[*c*]phenanthrene (3i)

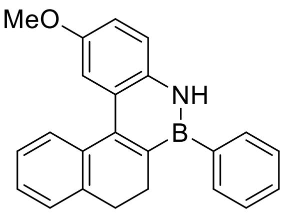

Following the general procedure using compound **1i** (62.83 mg, 0.25 mmol, 1 equiv) and cold (≈5 ºC) boron trichloride solution (0.5 mL, 0.5 mmol, 2.0 equiv.) and the reaction was stirred for 4 hours at room temperature. The B-Cl intermediate formed was treated with phenylmagnesium bromide solution (0.25 mL, 0.25 mmol, 1 equiv.) to give **3i** (53.1 mg, 0,16 mmol, 63%) as a white solid. M.p.: 120−121 ºC.

1H-NMR (500 MHz, CDCl3) δ (ppm) 7.89 (dd, *J* = 7.4, 1.5 Hz, 1H), 7.86 (bs, 1H), 7.82 (d, *J* = 2.7 Hz, 1H), 7.70 (dd, *J* = 7.8, 1.7 Hz, 2H), 7.50-7.42 (m, 3H), 7.41–7.31 (m, 3H), 7.29 (d, *J* = 8.8 Hz, 1H), 7.10 (dd, *J* = 8.8, 2.7 Hz, 1H), 3.86 (s, 3H), 2.86 –2.77 (m, 2H), 2.69 (t, *J* = 6.8, 2H).

13C-NMR (125 MHz, CDCl3) δ (ppm) 154.0 (C), 145.9 (C), 141.2 (C), 135.2 (C), 133.9 (C), 133.0 (2xCH), 128.6 (CH), 128.1 (2xCH), 127.9 (CH), 127.8 (CH), 127.7 (CH), 125.8 (CH), 123.4 (C), 119.8 (CH), 116.4 (CH), 110.2 (CH), 56.1 (CH_3), 29.5 (CH_2), 28.1 (CH_2). (The carbon atoms bonded to the boron atom are not observed.

11B-NMR (128 MHz, CDCl3) δ (ppm) 33.73.

HRMS (EI) calculated for $C_{23}H_{20}BNO$ $[M]^+$: 337.1638; found $[M+H]^+$: 337.1642.

Synthesis of 6-phenyl-5,6,7,8-tetrahydro-5-aza-6-borabenzo[*c*]chrysene (3j)

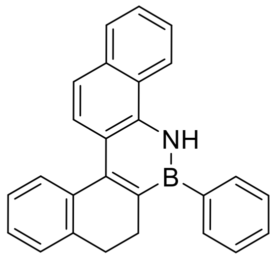

Following the general procedure using compound **1j** (68.0 mg, 0.25 mmol, 1 equiv.) and cold (≈5 ºC) boron trichloride solution (0.5 mL, 0.5 mmol, 2.0 equiv.) and stirring the reaction for 18 hours at room temperature. The B-Cl intermediate formed was treated with phenylmagnesium bromide solution (0.25 mL, 0.25 mmol, 1 equiv.) to give **3j** (55.5 mg, 0.16 mmol, 62%) as a brown oil.

^{1}H-NMR (500 MHz, $CDCl_3$) δ (ppm) 9.02 (bs, 1H), 8.36–8.30 (m, 2H), 7.96 (dd, *J* = 8.0, 1.4 Hz, 1H), 7.84–7.79 (m, 3H), 7.66 (ddd, *J* = 8.4, 6.9, 1.5 Hz, 1H), 7.64–7.58 (m, 2H), 7.57–7.48 (m, 3H), 7.44–7.34 (m, 3H), 2.93–2.88 (m, 2H), 2.79–2.72 (m, 2H).

^{13}C-NMR (125 MHz, $CDCl_3$) δ (ppm) 147.5 (C), 141.1 (C), 136.1 (C), 133.9 (C), 133.1 (2xCH), 132.8 (C), 128.8 (CH), 128.70 (CH), 128.67 (CH), 128.2 (2xCH), 127.71 (CH), 127.66 (CH), 126.5 (CH), 126.1 (CH), 126.0 (CH), 125.7 (CH), 124.4 (C), 120.6 (CH), 119.7 (CH), 118.7 (C), 29.5 (CH_2), 27.8 (CH_2). The carbon atoms bonded to the boron atom are not observed.

^{11}B-NMR (128 MHz, $CDCl_3$) 33.78.

HRMS (APCI) calculated for $C_{26}H_{21}BN$ [M+H]$^+$: 358.1766; found [M+H]$^+$: 358.1767.

Synthesis of 6-phenyl-4,5,6,7-tetrahydro-7-aza-6-boraphenanthro[3,4-*b*]thiophene (3k)

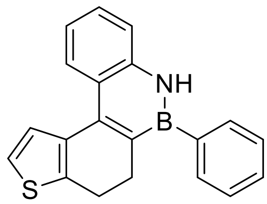

Following the general procedure using compound **1k** (57.0 mg, 0.21 mmol, 1 equiv.) and cold (≈5 ºC) boron trichloride solution (0.42 mL, 0.42 mmol, 2.0 equiv.) and stirring the reaction for 4 hours at room temperature. The B-Cl intermediate formed was treated with phenylmagnesium bromide solution (0.21 mL, 0.21 mmol, 1 equiv.) to give **3k** (42.8 mg, 0,14 mmol, 65%) as a yellow oil.

^{1}H-NMR (500 MHz, $CDCl_3$) 8.33 (d, *J* = 8.2 Hz, 1H), 7.89 (bs, 1H), 7.70 (m, 2H), 7.62 (d, *J* = 5.2 Hz, 1H), 7.51–7.41 (m, 4H), 7.33 (dd, *J* = 8.0, 1.3 Hz, 1H), 7.28–7.21 (m, 2H), 3.00–3.91 (m, 2H), 2.88–2.79 (m, 2H) .

^{13}C-NMR (75 MHz, $CDCl_3$) 143.6 (C), 141.8 (C), 140.0 (C), 134.1 (C), 132.8 (2xCH), 128.5 (CH), 128.0 (2xCH), 127.4 (CH), 127.2 (CH), 127.1 (CH), 122.9 (C), 121.0 (CH), 120.4 (CH), 118.7 (CH), 28.7 (CH_2), 23.4 (CH_2). The carbon atoms bonded to the boron atom are not observed.

^{11}B-NMR (128 MHz, $CDCl_3$) δ (ppm) 34.50.

HRMS (ESI$^+$) calculated for $C_{20}H_{17}BNS$ [M+H]$^+$: 314.1169; found [M+H]$^+$: 314.1169.

Synthesis of 7,16-diphenyl-5,6,7,8,14,15,16,17-octahydro-8,17-diaza-7,16-diborabenzo[*a*] naphtha[1,2-*k*]tetraphene (5)

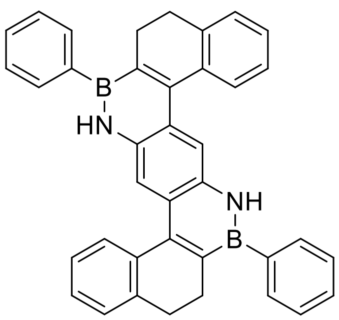

Following the general procedure using compound **4a** (58.88 mg, 0.13 mmol, 1 equiv.) and cold (≈5 ºC) boron trichloride solution (0.52 mL, 0.52 mmol, 4.0 equiv.) and stirring the reaction for 16 hours at −30 ºC. The B-Cl intermediate formed was treated with phenylmagnesium bromide solution (0.26 mL, 0.26 mmol, 2 equiv.) to give **5** (45.3 mg, 0,08 mmol, 70%) as a white solid. M. p.: 119−120 ºC.

^{1}H-NMR (500 MHz, $CDCl_3$) δ (ppm) 8.23 (bs, 2H), 7.98–7.86 (m, 4H), 7.74–7.66 (m, 4H), 7.53–7.32 (m, 12H), 2.90–2.79 (m, 4H), 2.78–2.66 (m, 4H).

^{13}C-NMR (125 MHz, $CDCl_3$) δ (ppm) 145.0 (2xC), 141.0 (2xC), 135.2 (2xC), 133.8 (2xC), 132.9 (4xCH), 128.6 (2xCH), 128.0 (4xCH), 127.8 (4xCH), 127.7 (2xCH), 125.8 (2xCH), 123.1 (2xC), 115.8 (2xCH), 29.2 (2xCH_2), 28.0 (2xCH_2). The carbon atoms bonded to the boron atom are not observed.

^{10}B-NMR (128 MHz, $CDCl_3$) δ (ppm) 31.78.

HRMS (APCI) calculated for $C_{38}H_{31}B_2N_2$ $[M+H]^+$: 537.2688; found $[M+H]^+$: 537.2680.

Synthesis of 2-mesityl-4,7-bis(4-phenylbut-1-yn-1-yl)-2,3-dihydro-1H-benzo[*d*][1,3,2]diaza borole (6)

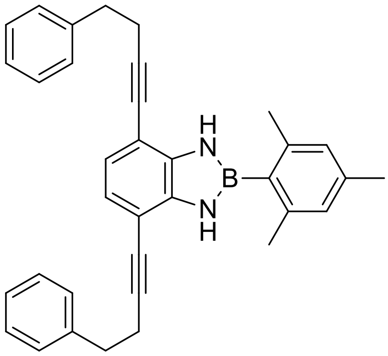

Following the general procedure using compound **4b** (113.2 mg, 0.25 mmol, 1 equiv.) and cold (≈5 ºC) boron trichloride solution (1.0 mL, 1.0 mmol, 4.0 equiv.) and stirring the reaction for 18 hours at 60 ºC. The resulting mixture was concentrated under reduced pressure, redissolved in toluene and treated with phenylmagnesium bromide solution (0.5 mL, 0.5 mmol, 2 equiv.). The remaining Grignard was quenched with a 2-propanol:toluene solution (2:8) and after 10 min the resulting mixture was filtered through a Celite plug. The filtrate was concentrated under reduced pressure and the crude product was purified via silica gel column chromatography (hexane:ethyl acetate 98:2) to give **6** (108.6 mg, 0,175 mmol, 89%) as a brown oil.

^{1}H-NMR (500 MHz, $CDCl_3$) δ (ppm) 7.30–7.25 (m, 4H), 7.24–7.16 (m, 4H), 7.01–6.95 (m, 2H), 6.95 (s, 2H), 6.87 (s, 2H), 6.44 (bs, 2H), 2.94 (t, *J* = 7.2 Hz, 4H), 2.82 (t, *J* = 7.1 Hz, 4H), 2.38 (s, 3H), 2.24 (s, 6H).

^{13}C-NMR (125 MHz, $CDCl_3$) δ (ppm) 141.7 (2xC), 140.7 (2xC), 138.7 (C), 137.6 (2xC), 128.7 (4xCH), 128.6 (4xCH), 127.2 (2xCH), 126.7 (2xCH), 121.4 (2xCH), 106.2 (2xC), 94.3 (2xC), 78.1 (2xC), 35.3 (4xCH_2), 23.4 (CH_3), 22.0 (2xCH_3). The carbon atom bonded to the boron atom is not observed.

^{11}B-NMR (128 MHz, $CDCl_3$) δ (ppm) 30.31.

HRMS: not ionized (ESI) or decomposed (EI).

General procedure for the oxidation of BN-arenes 3

Compound **3** (1 equiv.), 2,4,6-tri-tert-butylpyridine (TBP) (2.1 equiv.) and $[Ph_3C]BF_4$ (2.1 equiv.) were dissolved in DCE (0.1 M) in a Schlenk flask and the reaction mixture was stirred for 24 hours at 75 ºC. Upon cooling, the reaction mixture was passed through a plug of Celite using 20:80 DCM:hexane as eluent. The solvent was removed under reduced pressure and the resulting residue redissolved in hexane and filtered. Then the filtrate was evaporated under reduced pressure and the resulting crude product was further purified via silica gel column chromatography.

Synthesis of 6-phenyl-5,6-dihydro-5-aza-6-borabenzo[*c*]phenanthrene (7c)

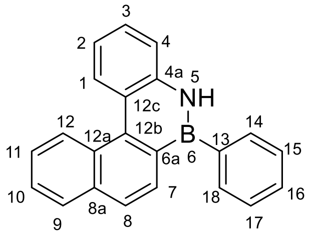

Following the general procedure using compound **3c** (50 mg, 0.16 mmol), TBP (83.1 mg, 0.34 mmol) and $[Ph_3C]BF_4$ (112.2 mg, 0.34 mmol) in DCE (0.16 mL). Purification by flash column chromatography (eluent = hexane:ethyl acetate:toluene 98:2:5) gave **7c** (24.9 mg, 0.082 mmol, 51%) as a white solid. M. p: 114–115 ºC.

^{1}H-NMR (500 MHz, $CDCl_3$) δ (ppm) 8.98 (m, 1H, H-12), 8.74 (d, *J* = 8.2 Hz, 1H, H-1), 8.17 (d, *J* = 8.2 Hz, 1H. H-7), 8.06–8.00 (m, 1H, H-9), 7.89 (bs, 1H, N-H), 7.87 (d, *J* = 8.2 Hz, 1H, H-8), 7.85–7.78 (m, 2H, H-14, H-18), 7.68–7.60 (m, 2H, H-10, H-11), 7.58–7.47 (m, 4H, H-3, H-15, H-16, H-17), 7.42 (d, *J* = 7.5, 1H, H-4), 7.37–7.28 (m, 1H, H-2).

^{13}C-NMR (125 MHz, $CDCl_3$) δ (ppm) 139.7 (C, C-4a), 139.1 (C, C-12b), 137.6 (C, C-13)*, 136.0 (C, C-8a), 133.4 (2xCH, C-14, C-18), 131.1 (CH, C-7), 130.8 (C, C-6a)*, 130.2 (CH, C-1), 129.7 (C, C-12a), 128.7 (CH, C-16), 128.4 (CH, C-9), 128.2 (CH, C-12), 128.1 (2xCH, C-15, C-17), 127.7 (CH, C-3), 126.9 (CH, C-10), 126.4 (CH, C-8), 125.5 (CH, C-11), 123.5 (C, C-12c), 120.9 (CH, C-2), 118.9 (CH, C-4). *Carbon not observed in ^{13}C-NMR, assigned by gHMBC.

^{11}B-NMR (128 MHz, $CDCl_3$) δ (ppm) 36.28.

HRMS (APCI) calculated for $C_{22}H_{17}BN$ $[M+H]^+$: 306.1453; found $[M+H]^+$: 306.1453.

Synthesis of 2-bromo-6-phenyl-5,6-dihydro-5-aza-6-borabenzo[*c*]phenanthrene (7g)

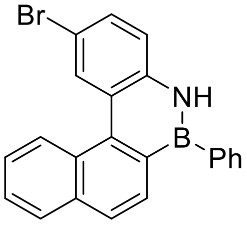

Following the general procedure using compound **3g** (50 mg, 0.13 mmol), TBP (83.1 mg, 0.34 mmol) and $[Ph_3C]BF_4$ (112.2 mg, 0.34 mmol) in DCE (0.13 mL). Purification by flash column chromatography (eluent = hexane:ethyl acetate:toluene 98:2:5) gave **7g** (29.5 mg, 0.077 mmol, 59%) as a white solid. M. p. 130−131 ºC.

^{1}H-NMR (300 MHz, $CDCl_3$) δ (ppm) 8.93–8.88 (m, 1H), 8.86 (d, *J* = 2.2 Hz, 1H), 8.16 (d, *J* = 8.2 Hz, 1H), 8.05–8.00 (m, 1H), 7.89 (d, *J* = 8.1, 1H), 7.86 (bs, 1H), 7.82–7.78 (m, 2H), 7.71–7.65 (m, 2H), 7.58 (dd, *J* = 8.5, 2.2 Hz, 1H), 7.56–7.51 (m, 3H), 7.30 (d, *J* = 8.5 Hz, 1H).

^{13}C-NMR (125 MHz, $CDCl_3$) δ (ppm) 138.7 (C), 137.9 (C), 136.1 (C), 133.5 (2xCH), 132.5 (CH), 131.1 (CH), 130.5 (CH), 129.6 (C), 129.1 (CH), 128.8 (CH), 128.3 (2xCH), 127.7 (CH), 127.3 (2xCH), 126.4 (CH), 125.3 (C), 120.6 (CH), 113.9 (C). The carbon atoms bonded to the boron atom are not observed.

^{11}B-NMR (128 MHz, $CDCl_3$) δ (ppm) 36.30.

HRMS (APCI) calculated for $C_{22}H_{16}BBrN$ $[M+H]^+$: 384.0558; found $[M+H]^+$: 384.0544.

Synthesis of 6-phenyl-5,6-dihydro-5-aza-6-borabenzo[*c*]chrysene (7j)

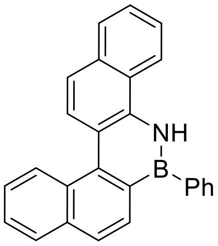

Following the general procedure using compound **3j** (78.9 mg, 0.16 mmol), TBP (83.1 mg, 0.34 mmol) and $[Ph_3C]BF_4$ (112.2 mg, 0.34 mmol) in DCE (0.16 mL). Purification by flash column chromatography (eluent = hexane:ethyl acetate: 98:2) gave **7j** (34.7 mg, 0.10 mmol, 61%) as a white solid. M. p. 135−136 ºC.

^{1}H-NMR (500 MHz, $CDCl_3$) δ (ppm) 9.02–8.93 (m, 2H), 8.77 (d, *J* = 9.0 Hz, 1H), 8.37 (d, *J* = 8.3 Hz, 1H), 8.26 (d, *J* = 8.2, 1H), 8.08–7.99 (m, 2H), 7.95–7.87 (m, 3H), 7.73 (d, *J* = 9.1 Hz, 1H), 7.71–7.62 (m, 4H), 7.61–7.53 (m, 3H).

^{13}C-NMR (125 MHz, $CDCl_3$) 140.2 (C), 136.3 (2xC), 136.4 (C), 133.8 (2xCH), 133.1 (C), 131.1 (CH), 129.9 (C), 129.0 (CH), 128.8 (2xCH), 128.6 (CH), 128.4 (2xCH), 128.3 (CH), 127.2 (CH), 126.8 (CH), 126.5 (CH), 126.3 (CH), 125.6 (CH), 124.6 (C), 120.5 (CH), 120.3 (CH). The carbon atoms bonded to the boron atom are not observed.

^{11}B-NMR (128 MHz, $CDCl_3$) δ (ppm) 36.25.

HRMS (EI) calculated for $C_{26}H_{18}BN$ $[M+H]^+$: 355.1532; found $[M+H]^+$: 355.1535.

Synthesis of 6-phenyl-6,7-dihydro-7-aza-6-boraphenanthro[3,4-*b*]thiophene (7k)

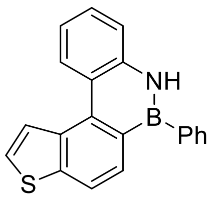

Following the general procedure using compound **3k** (42.8 mg, 0,14 mmol), TBP (72.7 mg, 0.29 mmol) and $[Ph_3C]BF_4$ (95.7 mg, 0.29 mmol) in DCE (0.14 mL). Purification by flash column chromatography (eluent = hexane:ethyl acetate: 97:3) gave **7k** (26.1 mg, 0.084 mmol, 60%) as a colourless oil.

^{1}H-NMR (300 MHz, $CDCl_3$) δ (ppm) 8.80 (d, *J* = 8.0 Hz, 1H), 8.45 (d, *J* =5.6 Hz, 1H), 8.13 (d, *J* = 8.2 Hz, 1H), 8.01 (d2, *J* = 8.2, 0.9 Hz, 1H), 7.85−7.74 (m, 3H), 7.66 (d, *J* = 5.6 Hz, 1H), 7.56−7.45 (m, 4H), 7.40−7.30 (m, 2H)

^{13}C-NMR (125 MHz, $CDCl_3$) δ (ppm) 144.7 (C), 139.5 (C), 133.4 (2xCH), 131.4 (CH), 129.2 (C), 128.8 (CH), 128.5 (CH), 128.4 (C), 128.2 (2xCH), 128.1 (CH), 126.0 (CH), 125.5 (CH). 124.2 (C), 121.5 (CH). 121.1 (CH), 119.2 (CH). The carbon atoms bonded to the boron atom are not observed.

^{10}B-NMR (128 MHz, $CDCl_3$) δ (ppm) 36.78.

HRMS (EI) calculated for $C_{20}H_{14}BNS$ $[M+H]^+$: 311.0940; found $[M+H]^+$: 311.0951.

<u>**Bromination of BN-benzo[*c*]phenanthrene 7c:**</u>

Synthesis of 2,8-dibromo-6-phenyl-5,6-dihydro-5-aza-6-borabenzo[*c*]phenanthrene (7l)

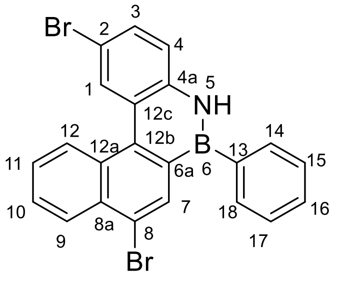

Compound **7c** (121 mg, 0,40 mmol) was loaded in a Schlenk flask under argon. Anhydrous DCM (2.6 mL) was added, and the resulting solution was cooled to 0 ºC. A recently prepared bromine solution (0.6 mL, 0.2 M in DCM, 3 equiv.) was added under argon. After the addition, the reaction mixture was slowly warmed to room temperature. The reaction was monitored by TLC to completion (1.5 h), the mixture was concentrated under reduced pressure. The remaining residue was purified by flash column

chromatography (eluent = hexane:ethyl acetate 98:2) to provide **7l** (121.1 mg, 0.26 mmol, 66%) as a white solid. M. p. 132−133 ºC.

^{1}H-NMR (500 MHz, CDCl$_3$) δ (ppm) 8.83 (d, *J* = 8.9 Hz, 1H, H-12), 8.72 (d, *J* = 2.0 Hz, 1H, H-1), 8.49–8.43 (m, 1H, H-9), 8.40 (s, 1H, H-7), 7.87 (bs, 1H, N-H), 7.82–7.69 (m, 4H, H-10, H-11, H-14, H-18), 7.61–7.50 (m, 4H, H-3, H-15, H-16, H-17), 7.30 (m, 1H, H-4).

^{13}C-NMR (125 MHz, CDCl$_3$) δ (ppm) 138.5 (C, C-4a), 138.2 (C, C-13)*, 137.6 (C, C-12b), 134.6 (CH, C-7), 134.0 (CH, C-8a), 133.3 (2xCH, C-14, C-18), 132.4 (C, C-1), 131.8 (C, C-6a)*, 130.8 (C, C-3), 130.7 (CH, C-12a), 129.1 (CH, C-16), 128.3 (3xCH, C-10, C-15, C-17), 127.9 (CH, C-12), 127.7 (CH, C-9), 126.9 (CH, C-11), 124.5 (C, C-2), 122.3 (C, C-8), 120.5 (CH, C-4), 113.9 (C, C-12c). *Carbon not observed in ^{13}C-NMR, assigned by gHMBC.

^{11}B-NMR (128 MHz, CDCl$_3$) δ (ppm) 36.00.

HRMS (EI) calculated for $C_{22}H_{14}BBr_2N$ [M+H]$^+$: 460.9586; found [M+H]$^+$: 460.9595.

Functionalization of BN-benzo[*c*]phenanthrenes by cross-couplings:

Synthesis of 2,6,8-triphenyl-5,6-dihydro-5-aza-6-borabenzo[*c*]phenanthrene (7m)

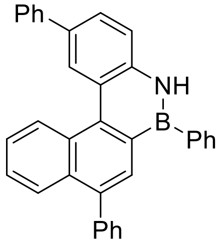

In an oven-dried Biotage microwave vial equipped with a stir bar were added **7l** (30 mg, 0.065 mmol), phenylboronic acid (11.1 mg, 0.09 mmol) and Pd(PPh$_3$)$_4$ (4.0 mg, 0.0033 mmol). Then the vial was sealed with a cap lined with a disposable Teflon septum S15. The corresponding mixture was dissolved in degassed toluene:methanol 3:1 mixture (0.4 mL) under argon. The resulting solution was treated with a suspension of sodium carbonate (162.5 mg) in degassed distilled water (0.75 mL) and the reaction was stirred at 70 ºC for 12 hours. At the end of the reaction, the mixture was concentrated under reduced pressure. The remaining residue was purified by flash column chromatography (eluent = hexane: ethyl acetate: 98:2) to provide **7m** (20.5 mg, 0.045 mmol, 69%) as a white solid. M. p. 101−103 ºC.

^{1}H-NMR (500 MHz, CDCl$_3$) δ (ppm) 9.07 (d, *J* = 8.5 Hz, 1H), 8.98 (s, 1H), 8.14 (s, 1H), 8.07 (d, *J* = 8.3 Hz, 1H), 7.97 (bs, 1H), 7.85–7.80 (m, 2H), 7.77 (dd, *J* = 8.3, 2.0 Hz, 1H), 7.75–7.70 (m, 2H), 7.68–7.62 (m, 1H), 7.61–7.54 (m, 3H), 7.53–7.46 (m, 8H), 7.46–7.41 (m, 1H), 7.40–7.35 (m, 1H).

^{13}C-NMR (125 MHz, CDCl$_3$) δ (ppm) 141.2 (C), 140.9 (C), 139.2 (C), 138.6 (C), 138.2 (C), 134.4 (C), 134.0 (C), 133.4 (2xCH), 131.6 (CH), 130.2 (2xCH), 130.0 (C), 128.9 (2xCH), 128.81 (CH), 128.75 (CH), 128.33 (2xCH), 128.30 (CH), 128.2 (2xCH), 127.2 (CH), 127.04 (2xCH), 127.02 (CH), 126.9 (CH), 126.7 (CH), 126.5 (CH), 125.6 (CH), 123.6 (C), 119.3 (CH). The carbon atoms bonded to the boron atom are not observed.

^{10}B-NMR (128 MHz, CDCl$_3$) δ (ppm) 34.52.

HRMS (APCI) calculated for $C_{34}H_{25}BN$ [M+H]$^+$: 458.2080 found [M+H]$^+$: 458.2073.

Synthesis of 2-(phenylethynyl)-6-phenyl-5,6-dihydro-5-aza-6-borabenzo[*c*]phenanthrene (7n)

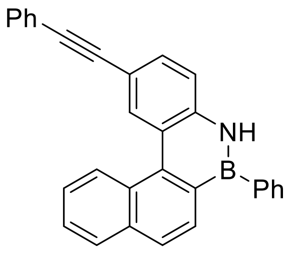

To an oven-dried Biotage microwave vial equipped with a stir bar was added $Pd(PPh_3)_4$ (2.6 mg, 0.0037mmol, 5 mol %), CuI (1 mg, 0.0037 mmol, 5 mol %) and compound **7g** (30 mg, 0.074 mmol 1.0 equiv.). The vial was sealed with a cap lined with a disposable Teflon septum, evacuated under vacuum, and purged with argon five times. Dimethylformamide (0.74 mL) and triethylamine (32 µL) were added, and the resulting suspension was stirred at room temperature for 30 min. Then, phenylacetylene (122.6 mg, 0.089 mmol, 1.2 equiv.) was injected, and the mixture was heated to 80 ºC and stirred at this temperature for 20 h. Purification by flash column chromatography on silica gel (hexane: ethyl acetate 95:5) gave BN-derivate **7n** (26.1 mg, 0.064 mmol, 87%) as white solid. M. p. 125−126 ºC.

^{1}H-NMR (500 MHz, CDCl$_3$) δ (ppm) 8.99 (d, *J* = 7.9 Hz, 1H), 8.92 (s, 1H), 8.17 (d, *J* = 8.2 Hz, 1H), 8.02 (d, *J* = 7.3 Hz, 1H), 7.92 (bs, 1H), 7.89 (d, *J* = 8.0 Hz, 1H), 7.85–7.77 (m, 2H), 7.74–7.63 (m, 3H), 7.62–7.57 (m, 2H), 7.56–7.48 (m, 3H), 7.42–7.29 (m, 4H).

^{13}C-NMR (125 MHz, CDCl$_3$) δ (ppm) 139.7 (C), 138.4 (C), 136.0 (C), 133.6 (CH), 133.4 (2xCH), 131.6 (2xCH), 131.0 (CH), 130.8 (CH), 129.6 (C), 128.9 (CH), 128.5 (CH), 128.4 (2xCH), 128.14 (2xCH), 128.07 (CH), 128.0 (CH), 127.0 (CH), 126.8 (CH), 126.0 (CH), 123.6 (C), 123.5 (C), 119.1 (CH), 115.8 (C), 90.0 (C), 88.4 (C). The C atoms bonded to the boron atom are not observed.

^{11}B-NMR (128 MHz, CDCl$_3$) δ (ppm) 36.25.

HRMS: not ionized (ESI) or decomposed (EI).

Synthesis of 2,8-bis(phenylethynyl)-6-phenyl-5,6-dihydro-5-aza-6-borabenzo[*c*]phenanthrene (7o)

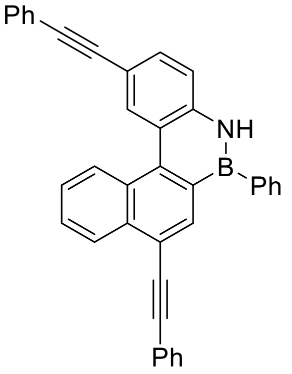

To an oven-dried Biotage microwave vial equipped with a stir bar was added $Pd(PPh_3)_4$ (4.2 mg, 0.006 mmol, 5 mol %), CuI (1.1 mg, 0.006 mmol, 5 mol %) and compound **7l** (60 mg, 0.12 mmol, 1 equiv.). The vial was sealed with a cap lined with a disposable Teflon septum, evacuated under vacuum, and purged with argon five times. DMF (1.20 mL) and NEt_3 (65 µL) were added, and the resulting suspension was stirred at room temperature for 30 min. Then, phenylacetylene (24.4 mg, 0.29 mmol, 2.4 equiv.) was injected, and the mixture was heated to 80 ºC and stirred at this temperature for 20 h. At the end of the reaction, the mixture was concentrated under reduced pressure. Purification by flash column chromatography on silica gel (hexane:EtOAc, 95:5) gave **7o** (60.7 mg, 0.11 mmol, 89%) as a white solid. M.p.: 135−136 ºC.

^{1}H-NMR (300 MHz, CDCl$_3$) δ (ppm) 8.96 (d, *J* = 7.6 Hz, 1H), 8.86 (bs, 1H), 8.69–8.66 (m, 1H), 8.43 (s, 1H), 7.95 (bs, 1H), 7.85–7.75 (m, 4H), 7.70–7.64 (m, 3H), 7.61–7.55 (m, 5H), 7.45–7.33 (m, 7H).

^{13}C-NMR (125 MHz, CDCl$_3$) δ (ppm) 140.0 (C), 139.0 (C), 135.8 (CH), 135.4 (C), 133.9 (CH), 133.5 (2xCH), 131.8 (2xCH), 131.7 (2xCH), 131.4 (CH), 129.5 (C), 129.1 (CH), 128.6 (2xCH), 128.5 (2xCH), 128.4 (3xCH), 128.3 (CH), 128.1 (C), 127.9 (CH), 126.8 (CH), 126.6 (CH), 123.6 (C), 123.5 (C), 123.3 (C), 119.5 (C), 119.3 (CH), 116.1 (CH), 94.9 (C), 90.0 (C), 88.7 (C), 88.2 (C). The carbon atoms bonded to the boron atom are not observed

^{11}B-NMR (128 MHz, CDCl$_3$) δ (ppm) 35.86.

HRMS (ESI$^+$) calculated for $C_{38}H_{25}BN$ $[M+H]^+$: 506.2075; found $[M+H]^+$: 506.2080.

Synthesis of 6-methyl -5,6-dihydro-5-aza-6-borabenzo[b]naphtho[2,1-d]thiophene (9)

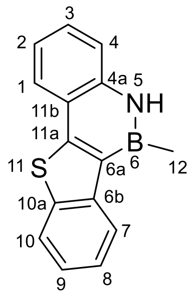

A Schlenk flask was evacuated under vacuum and purged with argon five times. Compound **8** (47.9 mg, 0.2 mmol, 1 equiv.) was dissolved in chloroform (0.8 mL) in the Schlenk flask. Cold (≈5 ºC) boron trichloride 1M solution in hexanes (0.4 mmol, 0.4 mL, 2.0 equiv.) was added dropwise via syringe to the vigorously stirring solution of **8** in chloroform. The reaction was stirred for 4 hours at 50 ºC. The resulting mixture was concentrated under reduced pressure, redissolved in toluene and treated with a methylmagnesium bromide solution (0.2 mL, 0.2 mmol, 1 equiv.) The reaction mixture was stirred for 18 h. The remaining Grignard was quenched with a 2-propanol:toluene solution (2:8) and after 10 min the resulting mixture was filtered through a Celite plug. The filtrate was concentrated under reduced pressure and the crude product was further purified via silica gel column chromatography (hexane:ethyl acetate 95:5) to give **9** (24.2 mg, 0.1 mmol, 50%) as a white solid.

M. p.: 110−111 ºC.

^{1}H-NMR (500 MHz, $CDCl_3$) 8.28 (d, *J* = 8.0 Hz, 1H, H-7), 7.99 (d, *J* = 7.2 Hz, 1H, H-1), 7.97 (d, *J* = 7.3 Hz, 1H, H-10), 7.65 (bs, 1H, N-H), 7.50–7.38 (m, 3H, H-3, H-8, H-9), 7.31 (d, *J* = 8.1 Hz, 1H, H-4), 7.28–7.21 (m, 1H, H-2), 1.23 (s, 3H. H-12).

^{13}C-NMR (125 MHz, $CDCl_3$) δ (ppm) 151.5 (C, C-11a), 143.9 (C, C-6b), 139.7 (C, C-10a), 138.7 (C, C-4a), 134.1 (C, C-6a)*,4a11b4a11b), 128.3 (CH, C-3), 125.8 (CH, C-1), 124.9 (CH, C-7), 124.6 (CH, C-9), 124.5 (CH, C-8), 122.2 (CH, C-2), 121.3 (CH, C-10), 121.2 (C, C-11b), 118.3 (CH, C-4), 3.5 (CH_3, C-12)**. *Carbon not observed in ^{13}C-NMR, assigned by HMBC. ** Carbon not observed in ^{13}C-NMR, assigned by HSQC.

^{11}B-NMR (128 MHz, $CDCl_3$) δ (ppm) 33.50.

HRMS (APCI) calculated for $C_{15}H_{13}BNS$ [M+H]$^+$: 250.0859; found [M+H]$^+$: 250.0857.

X-RAY CRYSTALLOGRAPHIC DATA FOR 7l

Crystallographic data are presented in Tables 1-7. Colourless crystals of **7l** were grown by slow evaporation at room temperature from a solution of the compound in cyclohexane. A single crystal of **7l** was coated in high-vacuum grease and mounted on a glass fibre. X-ray measurements were made using a Bruker D8 VENTURE PhtotonIII area-detector diffractometer with Cu-K_{α} radiation (α = 1.54 Å). Absorption corrections were applied, based on multiple and symmetry-equivalent measurements. The structure was solved by ShelXT structure solution program using Intrinsic Phasing and refined with the XL refinement package using Least Squares minimisation.

All non-hydrogen atoms were assigned anisotropic displacement parameters and refined without positional constraints and all other hydrogen atoms were constrained to ideal geometries and refined with fixed isotropic displacement parameters.

Refinement proceeded smoothly to give the residuals shown in Table 1.

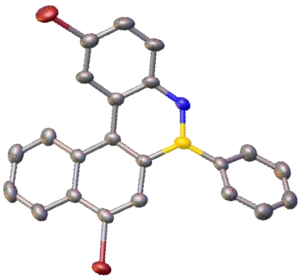

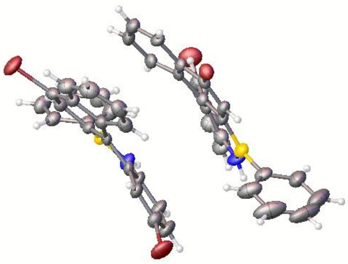

A colorless prism-like specimen of $C_{22}H_{14}BBr_2N$, approximate dimensions 0.200 mm x 0.300 mm x 0.300 mm, was used for the X-ray crystallographic analysis. The X-ray intensity data were measured (λ = 1.54184 Å).

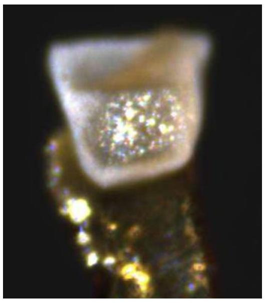

References

1. Sheldrick, G.M. (2015). Acta Cryst A71, 3-8; APEX3 Version 2016.7 (Bruker AXS Inc.) Bruker Instrument Service vV6.2.10
2. SAINT integration software, SAINT V8.38A (Bruker AXS Inc., 2017)
3. SADABS-2016/2 - Bruker AXS area detector scaling and absorption correction (Sheldrick, Bruker AXS Inc.)
4. *SHELXTL program system version 6.1;* XPREP Version 2013/3 (Sheldrick, Bruker AXS Inc.) XS Version 2013/1 (George M. Sheldrick, *Acta Cryst.* (2008). A**64**, 112-122)
5. *International Tables for Crystallography*, Kluwer, Dordrecht, 1992, vol. C.

Table A: Data collection details for 7l.

Axis	dx/mm	2θ/°	ω/°	ϕ/°	χ/°	Width/°	Frames	Time/s	Wavelength/Å	Voltage/kV	Current/mA	Temperature/K
Phi	44.907	110.79	10.53	0.00	22.00	2.00	180	5.00	1.54184	50	1.1	n/a
Phi	44.907	80.79	75.17	0.00	-44.50	2.00	180	5.00	1.54184	50	1.1	n/a
Omega	44.907	110.79	-10.02	0.00	61.50	2.00	60	5.00	1.54184	50	1.1	n/a
Omega	44.907	110.79	-10.02	-168.00	61.50	2.00	60	5.00	1.54184	50	1.1	n/a
Omega	44.907	-45.19	-55.08	40.00	-61.50	2.00	56	5.00	1.54184	50	1.1	n/a
Omega	44.907	-45.19	-55.08	-160.00	-61.50	2.00	56	5.00	1.54184	50	1.1	n/a
Omega	44.907	-30.19	-132.72	180.00	44.50	2.00	54	5.00	1.54184	50	1.1	n/a
Phi	44.907	110.79	103.36	0.00	-22.00	2.00	180	5.00	1.54184	50	1.1	n/a
Omega	44.907	-30.19	-132.72	0.00	44.50	2.00	54	5.00	1.54184	50	1.1	n/a
Omega	44.907	110.79	-10.02	128.00	61.50	2.00	60	5.00	1.54184	50	1.1	n/a
Omega	44.907	110.79	-10.02	-72.00	61.50	2.00	60	5.00	1.54184	50	1.1	n/a
Phi	44.907	110.79	105.17	0.00	-44.50	2.00	180	5.00	1.54184	50	1.1	n/a
Omega	44.907	-30.19	-132.72	270.00	44.50	2.00	54	5.00	1.54184	50	1.1	n/a
Omega	44.907	110.79	-10.02	64.00	61.50	2.00	60	5.00	1.54184	50	1.1	n/a
Omega	44.907	-30.19	-132.72	90.00	44.50	2.00	54	5.00	1.54184	50	1.1	n/a
Omega	44.907	110.79	-10.02	-136.00	61.50	2.00	60	5.00	1.54184	50	1.1	n/a
Omega	44.907	95.79	90.17	180.00	-44.50	2.00	43	5.00	1.54184	50	1.1	n/a
Omega	44.907	110.79	-10.02	-40.00	61.50	2.00	60	5.00	1.54184	50	1.1	n/a
Omega	44.907	110.79	-10.02	32.00	61.50	2.00	60	5.00	1.54184	50	1.1	n/a
Omega	44.907	110.79	-10.02	160.00	61.50	2.00	60	5.00	1.54184	50	1.1	n/a
Omega	44.907	110.79	-10.02	96.00	61.50	2.00	60	5.00	1.54184	50	1.1	n/a
Omega	44.907	110.79	-10.02	-104.00	61.50	2.00	60	5.00	1.54184	50	1.1	n/a
Omega	44.907	-15.19	-125.44	0.00	44.50	2.00	58	5.00	1.54184	50	1.1	n/a

A total of 1809 frames were collected. The total exposure time was 2.51 hours. The frames were integrated with the Bruker SAINT software package using a narrow-frame algorithm. The integration of the data using a triclinic unit cell yielded a total of 45330 reflections to a maximum θ angle of 72.27 (0.81 Å resolution). The final cell constants of a = 8.5993(5) Å, b = 13.7989(8) Å, c = 15.2625(9) Å, α = 90 , β = 90.375(2), γ = 90, volume = 1811.02(18) $Å^3$, are based upon the refinement of the XYZ-centroids of 9498 reflections above 20 σ(I) with 8.637 < 2θ < 144.3. Data were corrected for absorption effects using the Multi-Scan method (SADABS). The ratio of minimum to maximum apparent transmission was 0.352.

Table 1 Crystal data and structure refinement for **7l**

Identification code	**ESO572F1**
Empirical formula	$C_{22}H_{14}BBr_2N$
Formula weight	462.97
Temperature/K	301.0
Crystal system	monoclinic
Space group	$P2_1$
a/Å	8.5993(5)
b/Å	13.7989(8)
c/Å	15.2625(9)
α/°	90
β/°	90.375(2)
γ/°	90
Volume/Å^3	1811.02(18)
Z	4
ρ_{calc}g/cm^3	1.698
μ/mm^{-1}	5.699
F(000)	912.0
Crystal size/mm^3	0.3 × 0.3 × 0.2
Radiation	CuKα (λ = 1.54178)
2Θ range for data collection/°	8.638 to 144.46
Index ranges	-8 ≤ h ≤ 10, -17 ≤ k ≤ 17, -18 ≤ l ≤ 18
Reflections collected	35648
Independent reflections	7039 [R_{int} = 0.0804, R_{sigma} = 0.0564]
Data/restraints/parameters	7039/37/471
Goodness-of-fit on F^2	1.059
Final R indexes [I>=2σ (I)]	R_1 = 0.0505, wR_2 = 0.1252
Final R indexes [all data]	R_1 = 0.0538, wR_2 = 0.1303
Largest diff. peak/hole / e Å^{-3}	0.89/-1.15
Flack parameter	0.11(3)

Table 2 Fractional Atomic Coordinates (×10⁴) and Equivalent Isotropic Displacement Parameters (Å²×10³) for **7l**. U_{eq} is defined as 1/3 of of the trace of the orthogonalised U_{IJ} tensor.

Atom	*x*	*y*	*z*	U(eq)
Br^1	10423.0(8)	3172.7(6)	4389.0(5)	38.1(2)
Br^3	2347.5(14)	6050.1(8)	6347.3(6)	57.3(3)
Br^2	5864.6(16)	8635.7(8)	1301.8(8)	64.9(4)
Br^4	3362.0(17)	1132.0(8)	2089.6(8)	71.5(4)
N^2	2316(8)	5455(5)	2050(4)	38.7(15)
C^9	9244(7)	3970(6)	3616(5)	28.9(14)
C^8	9008(9)	3653(6)	2789(5)	32.9(15)
C^{10}	8521(8)	4829(6)	3935(4)	29.6(14)
C^{16}	7505(8)	5097(6)	2434(5)	31.4(15)
C^{37}	5412(9)	3559(6)	4353(5)	39.1(17)
C^{18}	6321(10)	5305(6)	958(5)	39.5(17)
C^{43}	2304(9)	5891(6)	4513(5)	35.0(16)
N^1	6701(9)	4350(6)	756(4)	45.4(17)
C^{29}	1374(9)	7037(6)	2749(5)	34.9(16)
C^{42}	2881(9)	5474(6)	5249(5)	34.7(16)
C^{30}	1978(12)	7677(7)	2126(6)	46(2)
C^{22}	6671(10)	6727(6)	1833(5)	40.1(18)
C^{35}	3369(8)	4596(5)	3631(4)	28.5(14)
C^{34}	197(10)	7391(6)	3269(5)	39.3(17)
C^{39}	5757(11)	3591(8)	5922(6)	51(2)
C^{40}	4639(11)	4299(7)	5983(6)	47(2)
C^{44}	2554(8)	5467(5)	3677(4)	29.9(15)
C^{17}	6791(9)	5709(6)	1750(5)	34.8(16)
C^{11}	8630(10)	5105(6)	4831(5)	37.0(17)
C^{41}	3888(9)	4675(6)	5234(5)	32.3(15)
C^{36}	4201(8)	4252(5)	4392(5)	30.2(14)
C^{26}	2757(9)	4484(6)	2038(5)	36.1(16)
C^{38}	6175(9)	3248(7)	5093(6)	45.3(19)
C^7	8109(8)	4194(6)	2181(4)	32.2(15)
C^{20}	5271(12)	6842(8)	472(6)	51(2)
C^{28}	3461(9)	3016(6)	2802(5)	36.5(16)
C^{27}	3273(8)	4046(6)	2820(5)	31.8(15)
C^{13}	6775(10)	6359(7)	4587(6)	41.6(19)
C^{12}	7797(10)	5868(6)	5139(5)	41.9(19)
C^{14}	6669(9)	6126(7)	3715(5)	36.2(15)
C^{33}	-348(12)	8346(8)	3181(6)	54(2)
C^1	8218(11)	2756(7)	926(5)	42.1(18)
C^{19}	5477(12)	5857(8)	339(5)	50(2)

Table 2 Fractional Atomic Coordinates (×10^4) and Equivalent Isotropic Displacement Parameters (Å^2×10^3) for **7l**. U_{eq} is defined as 1/3 of of the trace of the orthogonalised U_{IJ} tensor.

Atom	*x*	*y*	*z*	U(eq)
C^{25}	2594(12)	3951(7)	1270(6)	48(2)
C^{31}	1452(14)	8615(8)	2044(6)	59(3)
C^{5}	8390(20)	1021(10)	1063(10)	98(4)
C^{23}	3228(11)	2515(7)	2042(6)	44.0(19)
C^{21}	5937(11)	7265(7)	1194(6)	46(2)
C^{32}	294(15)	8942(8)	2571(7)	62(3)
C^{15}	7590(8)	5380(6)	3352(5)	30.8(15)
C^{6}	7860(17)	1897(8)	1369(7)	68(3)
C^{24}	2810(12)	2965(8)	1257(6)	53(2)
C^{2}	9111(12)	2685(9)	182(7)	59(2)
B^{1}	7677(11)	3773(7)	1268(5)	37.7(19)
B^{2}	2032(10)	5978(7)	2820(5)	33.1(17)
C^{4}	9330(20)	980(12)	331(11)	103(5)
C^{3}	9674(17)	1798(13)	-122(10)	89(4)

Table 3 Anisotropic Displacement Parameters (Å^2×10^3) for **7l**. The Anisotropic displacement factor exponent takes the form: $-2\pi^2[h^2a^{*2}U_{11}+2hka^*b^*U_{12}+\ldots]$.

Atom	U_{11}	U_{22}	U_{33}	U_{23}	U_{13}	U_{12}
Br^{1}	38.4(4)	45.5(5)	30.5(4)	11.4(3)	-0.9(3)	-0.2(3)
Br^{3}	88.4(8)	57.7(6)	25.9(4)	-8.2(4)	1.0(4)	12.4(5)
Br^{2}	92.5(8)	41.4(5)	60.5(6)	16.7(5)	-13.6(6)	0.9(5)
Br^{4}	113.7(10)	40.2(6)	60.4(6)	-16.0(5)	3.8(6)	-0.7(6)
N^{2}	56(4)	39(4)	21(3)	1(3)	1(3)	9(3)
C^{9}	23(3)	35(4)	29(3)	4(3)	1(2)	-3(3)
C^{8}	42(4)	31(3)	25(3)	-1(3)	7(3)	0(3)
C^{10}	33(3)	35(4)	21(3)	3(3)	4(2)	-5(3)
C^{16}	32(3)	40(4)	21(3)	4(3)	2(3)	-4(3)
C^{37}	40(4)	35(4)	42(4)	7(3)	4(3)	-3(3)
C^{18}	43(4)	45(5)	30(4)	10(3)	-1(3)	-3(3)
C^{43}	36(4)	35(4)	34(4)	-5(3)	2(3)	-3(3)
N^{1}	67(5)	46(4)	23(3)	-3(3)	-12(3)	-4(3)
C^{29}	43(4)	38(4)	24(3)	0(3)	-5(3)	-2(3)
C^{42}	39(4)	41(4)	24(3)	1(3)	-2(3)	-12(3)
C^{30}	60(5)	46(5)	33(4)	5(4)	4(4)	3(4)
C^{22}	48(4)	39(4)	33(4)	7(3)	2(3)	-1(3)
C^{35}	27(3)	34(4)	25(3)	0(3)	9(3)	-5(3)
C^{34}	47(4)	42(4)	29(4)	-1(3)	-3(3)	1(3)

Table 3 Anisotropic Displacement Parameters (Å^2×10^3) for **7l**. The Anisotropic displacement factor exponent takes the form: $-2\pi^2[h^2a^{*2}U_{11}+2hka^*b^*U_{12}+\ldots]$.

Atom	U_{11}	U_{22}	U_{33}	U_{23}	U_{13}	U_{12}
C^{39}	59(5)	49(5)	46(5)	18(4)	-15(4)	-1(4)
C^{40}	59(5)	48(5)	33(4)	5(4)	-10(4)	-3(4)
C^{44}	34(4)	34(4)	22(3)	-3(3)	3(3)	-4(3)
C^{17}	35(4)	45(4)	25(3)	5(3)	5(3)	-4(3)
C^{11}	43(4)	45(4)	24(3)	-4(3)	-1(3)	-8(3)
C^{41}	39(4)	33(4)	25(3)	1(3)	2(3)	-6(3)
C^{36}	33(3)	31(4)	27(3)	1(3)	3(3)	-5(3)
C^{26}	43(4)	40(4)	26(3)	-6(3)	10(3)	2(3)
C^{38}	38(4)	40(4)	58(5)	8(4)	0(3)	4(4)
C^{7}	36(4)	39(4)	22(3)	0(3)	5(3)	0(3)
C^{20}	62(6)	52(5)	39(5)	15(4)	-8(4)	9(4)
C^{28}	45(4)	38(4)	27(3)	-6(3)	9(3)	0(3)
C^{27}	32(3)	34(4)	30(3)	-2(3)	12(3)	-2(3)
C^{13}	43(4)	45(5)	36(4)	-6(3)	14(3)	-5(3)
C^{12}	52(5)	44(5)	30(4)	-3(3)	7(3)	-8(4)
C^{14}	37(4)	44(4)	27(3)	4(3)	7(3)	-8(3)
C^{33}	57(5)	58(6)	47(5)	-8(4)	-3(4)	19(4)
C^{1}	57(5)	44(4)	26(3)	-3(3)	-11(3)	-2(4)
C^{19}	66(6)	58(6)	27(4)	11(4)	-6(4)	0(4)
C^{25}	64(6)	52(5)	29(4)	-13(4)	3(4)	5(4)
C^{31}	92(8)	46(5)	38(5)	11(4)	2(5)	1(5)
C^{5}	157(13)	46(6)	90(8)	-7(6)	-59(7)	8(7)
C^{23}	51(5)	40(5)	41(4)	-8(4)	8(4)	2(4)
C^{21}	52(5)	49(5)	38(4)	20(4)	4(4)	-4(4)
C^{32}	97(8)	48(6)	41(5)	4(4)	-12(5)	20(5)
C^{15}	27(3)	36(4)	29(4)	-1(3)	8(3)	-8(3)
C^{6}	112(9)	47(5)	45(5)	1(4)	-20(5)	-10(5)
C^{24}	67(5)	57(6)	36(4)	-19(4)	2(4)	7(4)
C^{2}	61(6)	74(6)	43(5)	-17(5)	2(4)	-4(5)
B^{1}	49(5)	39(5)	25(4)	0(3)	2(3)	-6(4)
B^{2}	36(4)	38(5)	26(4)	-5(3)	4(3)	-3(3)
C^{4}	129(12)	75(8)	105(9)	-52(7)	-60(7)	34(8)
C^{3}	86(9)	112(9)	70(8)	-50(7)	-16(6)	19(8)

Table 4 Bond Lengths for **7l**.

Atom	Atom	Length/Å	Atom	Atom	Length/Å
Br^{1}	C^{9}	1.902(7)	C^{35}	C^{36}	1.441(10)
Br^{3}	C^{42}	1.914(8)	C^{35}	C^{27}	1.453(10)
Br^{2}	C^{21}	1.899(10)	C^{34}	C^{33}	1.405(13)
Br^{4}	C^{23}	1.913(9)	C^{39}	C^{40}	1.373(14)
N^{2}	C^{26}	1.392(11)	C^{39}	C^{38}	1.399(14)
N^{2}	B^{2}	1.403(10)	C^{40}	C^{41}	1.408(11)
C^{9}	C^{8}	1.350(10)	C^{44}	B^{2}	1.550(11)
C^{9}	C^{10}	1.425(11)	C^{11}	C^{12}	1.359(13)
C^{8}	C^{7}	1.416(10)	C^{41}	C^{36}	1.439(10)
C^{10}	C^{11}	1.423(10)	C^{26}	C^{27}	1.407(11)
C^{10}	C^{15}	1.414(10)	C^{26}	C^{25}	1.390(11)
C^{16}	C^{17}	1.475(10)	C^{7}	B^{1}	1.553(11)
C^{16}	C^{7}	1.404(11)	C^{20}	C^{19}	1.386(16)
C^{16}	C^{15}	1.457(10)	C^{20}	C^{21}	1.369(15)
C^{37}	C^{36}	1.415(11)	C^{28}	C^{27}	1.431(11)
C^{37}	C^{38}	1.371(12)	C^{28}	C^{23}	1.363(12)
C^{18}	N^{1}	1.393(12)	C^{13}	C^{12}	1.390(13)
C^{18}	C^{17}	1.388(11)	C^{13}	C^{14}	1.371(11)
C^{18}	C^{19}	1.410(12)	C^{14}	C^{15}	1.413(11)
C^{43}	C^{42}	1.353(11)	C^{33}	C^{32}	1.362(16)
C^{43}	C^{44}	1.421(10)	C^{1}	C^{6}	1.400(15)
N^{1}	B^{1}	1.394(12)	C^{1}	C^{2}	1.377(13)
C^{29}	C^{30}	1.400(12)	C^{1}	B^{1}	1.569(13)
C^{29}	C^{34}	1.380(12)	C^{25}	C^{24}	1.373(15)
C^{29}	B^{2}	1.571(12)	C^{31}	C^{32}	1.362(17)
C^{42}	C^{41}	1.402(12)	C^{5}	C^{6}	1.37(2)
C^{30}	C^{31}	1.376(15)	C^{5}	C^{4}	1.39(3)
C^{22}	C^{17}	1.413(12)	C^{23}	C^{24}	1.396(14)
C^{22}	C^{21}	1.377(12)	C^{2}	C^{3}	1.398(19)
C^{35}	C^{44}	1.394(11)	C^{4}	C^{3}	1.36(3)

Table 5 Bond Angles for **7l**.

Atom	Atom	Atom	Angle/˚	Atom	Atom	Atom	Angle/˚
C^{26}	N^{2}	B^{2}	123.7(7)	N^{2}	C^{26}	C^{27}	119.1(7)
C^{8}	C^{9}	Br^{1}	118.0(6)	C^{25}	C^{26}	N^{2}	119.6(8)
C^{8}	C^{9}	C^{10}	121.7(7)	C^{25}	C^{26}	C^{27}	121.2(8)
C^{10}	C^{9}	Br^{1}	120.1(5)	C^{37}	C^{38}	C^{39}	120.9(8)
C^{9}	C^{8}	C^{7}	121.3(7)	C^{8}	C^{7}	B^{1}	121.1(7)
C^{11}	C^{10}	C^{9}	121.7(7)	C^{16}	C^{7}	C^{8}	119.3(7)
C^{15}	C^{10}	C^{9}	118.6(6)	C^{16}	C^{7}	B^{1}	119.4(7)
C^{15}	C^{10}	C^{11}	119.6(7)	C^{21}	C^{20}	C^{19}	118.8(8)
C^{7}	C^{16}	C^{17}	117.9(7)	C^{23}	C^{28}	C^{27}	120.3(8)
C^{7}	C^{16}	C^{15}	119.1(7)	C^{26}	C^{27}	C^{35}	120.9(7)
C^{15}	C^{16}	C^{17}	123.0(7)	C^{26}	C^{27}	C^{28}	116.4(7)
C^{38}	C^{37}	C^{36}	121.7(8)	C^{28}	C^{27}	C^{35}	122.0(7)
N^{1}	C^{18}	C^{19}	118.9(8)	C^{14}	C^{13}	C^{12}	120.8(8)
C^{17}	C^{18}	N^{1}	120.4(7)	C^{11}	C^{12}	C^{13}	120.0(7)
C^{17}	C^{18}	C^{19}	120.8(8)	C^{13}	C^{14}	C^{15}	121.1(8)
C^{42}	C^{43}	C^{44}	120.9(7)	C^{32}	C^{33}	C^{34}	119.7(9)
C^{18}	N^{1}	B^{1}	123.9(7)	C^{6}	C^{1}	B^{1}	122.0(9)
C^{30}	C^{29}	B^{2}	119.9(7)	C^{2}	C^{1}	C^{6}	117.6(10)
C^{34}	C^{29}	C^{30}	116.4(8)	C^{2}	C^{1}	B^{1}	120.4(9)
C^{34}	C^{29}	B^{2}	123.7(7)	C^{20}	C^{19}	C^{18}	119.8(9)
C^{43}	C^{42}	Br^{3}	117.5(6)	C^{24}	C^{25}	C^{26}	121.6(9)
C^{43}	C^{42}	C^{41}	123.0(7)	C^{32}	C^{31}	C^{30}	119.9(10)
C^{41}	C^{42}	Br^{3}	119.5(5)	C^{6}	C^{5}	C^{4}	120.4(16)
C^{31}	C^{30}	C^{29}	122.2(9)	C^{28}	C^{23}	Br^{4}	117.7(7)
C^{21}	C^{22}	C^{17}	120.4(9)	C^{28}	C^{23}	C^{24}	122.7(9)
C^{44}	C^{35}	C^{36}	119.3(6)	C^{24}	C^{23}	Br^{4}	119.4(7)
C^{44}	C^{35}	C^{27}	117.9(7)	C^{22}	C^{21}	Br^{2}	119.4(8)
C^{36}	C^{35}	C^{27}	122.7(7)	C^{20}	C^{21}	Br^{2}	118.7(7)
C^{29}	C^{34}	C^{33}	121.5(9)	C^{20}	C^{21}	C^{22}	121.8(9)
C^{40}	C^{39}	C^{38}	119.1(8)	C^{31}	C^{32}	C^{33}	120.3(10)
C^{39}	C^{40}	C^{41}	121.7(9)	C^{10}	C^{15}	C^{16}	119.0(7)
C^{43}	C^{44}	B^{2}	121.7(7)	C^{14}	C^{15}	C^{10}	117.5(7)
C^{35}	C^{44}	C^{43}	118.7(7)	C^{14}	C^{15}	C^{16}	123.2(7)
C^{35}	C^{44}	B^{2}	119.5(6)	C^{5}	C^{6}	C^{1}	120.4(13)
C^{18}	C^{17}	C^{16}	120.2(8)	C^{25}	C^{24}	C^{23}	117.5(8)
C^{18}	C^{17}	C^{22}	117.1(7)	C^{1}	C^{2}	C^{3}	122.2(13)
C^{22}	C^{17}	C^{16}	122.4(7)	N^{1}	B^{1}	C^{7}	115.4(8)
C^{12}	C^{11}	C^{10}	120.6(8)	N^{1}	B^{1}	C^{1}	120.2(7)
C^{42}	C^{41}	C^{40}	123.9(7)	C^{7}	B^{1}	C^{1}	124.3(7)

Table 5 Bond Angles for **7l**.

Atom	Atom	Atom	Angle/˚	Atom	Atom	Atom	Angle/˚
C^{42}	C^{41}	C^{36}	116.9(7)	N^{2}	B^{2}	C^{29}	119.1(7)
C^{40}	C^{41}	C^{36}	119.2(8)	N^{2}	B^{2}	C^{44}	115.0(7)
C^{37}	C^{36}	C^{35}	123.4(7)	C^{44}	B^{2}	C^{29}	125.7(7)
C^{37}	C^{36}	C^{41}	117.0(7)	C^{3}	C^{4}	C^{5}	120.5(14)
C^{41}	C^{36}	C^{35}	119.5(7)	C^{4}	C^{3}	C^{2}	118.8(14)

Table 6 Torsion Angles for **7l**.

A	B	C	D	Angle/˚	A	B	C	D	Angle/˚
Br^{1}	C^{9}	C^{8}	C^{7}	178.4(5)	C^{17}	C^{22}	C^{21}	Br^{2}	-177.6(6)
Br^{1}	C^{9}	C^{10}	C^{11}	-1.1(9)	C^{17}	C^{22}	C^{21}	C^{20}	1.4(13)
Br^{1}	C^{9}	C^{10}	C^{15}	-177.0(5)	C^{11}	C^{10}	C^{15}	C^{16}	179.1(7)
Br^{3}	C^{42}	C^{41}	C^{40}	-4.7(11)	C^{11}	C^{10}	C^{15}	C^{14}	-6.8(10)
Br^{3}	C^{42}	C^{41}	C^{36}	177.8(5)	C^{36}	C^{37}	C^{38}	C^{39}	1.4(13)
Br^{4}	C^{23}	C^{24}	C^{25}	174.4(8)	C^{36}	C^{35}	C^{44}	C^{43}	-12.6(10)
N^{2}	C^{26}	C^{27}	C^{35}	-0.9(11)	C^{36}	C^{35}	C^{44}	B^{2}	163.9(6)
N^{2}	C^{26}	C^{27}	C^{28}	169.9(7)	C^{36}	C^{35}	C^{27}	C^{26}	-165.9(7)
N^{2}	C^{26}	C^{25}	C^{24}	-170.9(9)	C^{36}	C^{35}	C^{27}	C^{28}	23.9(10)
C^{9}	C^{8}	C^{7}	C^{16}	2.7(11)	C^{26}	N^{2}	B^{2}	C^{29}	-173.7(7)
C^{9}	C^{8}	C^{7}	B^{1}	-172.7(7)	C^{26}	N^{2}	B^{2}	C^{44}	11.6(11)
C^{9}	C^{10}	C^{11}	C^{12}	-172.9(7)	C^{26}	C^{25}	C^{24}	C^{23}	-1.1(15)
C^{9}	C^{10}	C^{15}	C^{16}	-5.0(10)	C^{38}	C^{37}	C^{36}	C^{35}	-179.9(8)
C^{9}	C^{10}	C^{15}	C^{14}	169.1(6)	C^{38}	C^{37}	C^{36}	C^{41}	4.9(11)
C^{8}	C^{9}	C^{10}	C^{11}	173.0(7)	C^{38}	C^{39}	C^{40}	C^{41}	1.0(14)
C^{8}	C^{9}	C^{10}	C^{15}	-2.8(10)	C^{7}	C^{16}	C^{17}	C^{18}	18.5(10)
C^{8}	C^{7}	B^{1}	N^{1}	175.8(7)	C^{7}	C^{16}	C^{17}	C^{22}	-156.1(8)
C^{8}	C^{7}	B^{1}	C^{1}	-2.0(12)	C^{7}	C^{16}	C^{15}	C^{10}	11.5(10)
C^{10}	C^{9}	C^{8}	C^{7}	4.1(11)	C^{7}	C^{16}	C^{15}	C^{14}	-162.2(7)
C^{10}	C^{11}	C^{12}	C^{13}	2.8(12)	C^{28}	C^{23}	C^{24}	C^{25}	-1.6(15)
C^{16}	C^{7}	B^{1}	N^{1}	0.5(11)	C^{27}	C^{35}	C^{44}	C^{43}	164.3(6)
C^{16}	C^{7}	B^{1}	C^{1}	-177.4(7)	C^{27}	C^{35}	C^{44}	B^{2}	-19.3(10)
C^{18}	N^{1}	B^{1}	C^{7}	10.4(12)	C^{27}	C^{35}	C^{36}	C^{37}	22.9(11)
C^{18}	N^{1}	B^{1}	C^{1}	-171.7(8)	C^{27}	C^{35}	C^{36}	C^{41}	-162.0(7)
C^{43}	C^{42}	C^{41}	C^{40}	172.6(8)	C^{27}	C^{26}	C^{25}	C^{24}	5.2(14)
C^{43}	C^{42}	C^{41}	C^{36}	-4.9(11)	C^{27}	C^{28}	C^{23}	Br^{4}	-175.8(6)
C^{43}	C^{44}	B^{2}	N^{2}	-178.1(7)	C^{27}	C^{28}	C^{23}	C^{24}	0.3(13)
C^{43}	C^{44}	B^{2}	C^{29}	7.7(11)	C^{13}	C^{14}	C^{15}	C^{10}	5.3(11)
N^{1}	C^{18}	C^{17}	C^{16}	-8.4(11)	C^{13}	C^{14}	C^{15}	C^{16}	179.1(7)
N^{1}	C^{18}	C^{17}	C^{22}	166.4(8)	C^{12}	C^{13}	C^{14}	C^{15}	0.3(12)

Table 6 Torsion Angles for **7l**.

A	B	C	D	Angle/°	A	B	C	D	Angle/°
N^1	C^{18}	C^{19}	C^{20}	-171.1(9)	C^{14}	C^{13}	C^{12}	C^{11}	-4.5(13)
C^{29}	C^{30}	C^{31}	C^{32}	-0.9(16)	C^1	C^2	C^3	C^4	0.0(19)
C^{29}	C^{34}	C^{33}	C^{32}	0.0(14)	C^{19}	C^{18}	N^1	B^1	172.4(8)
C^{42}	C^{43}	C^{44}	C^{35}	1.9(11)	C^{19}	C^{18}	C^{17}	C^{16}	172.7(7)
C^{42}	C^{43}	C^{44}	B^2	-174.5(7)	C^{19}	C^{18}	C^{17}	C^{22}	-12.5(12)
C^{42}	C^{41}	C^{36}	C^{37}	169.4(7)	C^{19}	C^{20}	C^{21}	Br^2	172.6(8)
C^{42}	C^{41}	C^{36}	C^{35}	-6.0(10)	C^{19}	C^{20}	C^{21}	C^{22}	-6.4(15)
C^{30}	C^{29}	C^{34}	C^{33}	-0.8(12)	C^{25}	C^{26}	C^{27}	C^{35}	-177.0(8)
C^{30}	C^{29}	B^2	N^2	-43.0(11)	C^{25}	C^{26}	C^{27}	C^{28}	-6.2(11)
C^{30}	C^{29}	B^2	C^{44}	131.1(8)	C^5	C^4	C^3	C^2	2(2)
C^{30}	C^{31}	C^{32}	C^{33}	0.0(17)	C^{23}	C^{28}	C^{27}	C^{35}	174.2(7)
C^{35}	C^{44}	B^2	N^2	5.6(10)	C^{23}	C^{28}	C^{27}	C^{26}	3.5(11)
C^{35}	C^{44}	B^2	C^{29}	-168.6(7)	C^{21}	C^{22}	C^{17}	C^{16}	-177.3(7)
C^{34}	C^{29}	C^{30}	C^{31}	1.2(13)	C^{21}	C^{22}	C^{17}	C^{18}	8.0(12)
C^{34}	C^{29}	B^2	N^2	136.5(8)	C^{21}	C^{20}	C^{19}	C^{18}	1.9(15)
C^{34}	C^{29}	B^2	C^{44}	-49.5(11)	C^{15}	C^{10}	C^{11}	C^{12}	2.9(11)
C^{34}	C^{33}	C^{32}	C^{31}	0.4(16)	C^{15}	C^{16}	C^{17}	C^{18}	-160.6(7)
C^{39}	C^{40}	C^{41}	C^{42}	-172.0(9)	C^{15}	C^{16}	C^{17}	C^{22}	24.8(11)
C^{39}	C^{40}	C^{41}	C^{36}	5.4(13)	C^{15}	C^{16}	C^7	C^8	-10.4(10)
C^{40}	C^{39}	C^{38}	C^{37}	-4.5(14)	C^{15}	C^{16}	C^7	B^1	165.1(7)
C^{40}	C^{41}	C^{36}	C^{37}	-8.2(11)	C^6	C^1	C^2	C^3	-1.0(15)
C^{40}	C^{41}	C^{36}	C^{35}	176.4(7)	C^6	C^1	B^1	N^1	-119.0(10)
C^{44}	C^{43}	C^{42}	Br^3	-175.4(5)	C^6	C^1	B^1	C^7	58.7(12)
C^{44}	C^{43}	C^{42}	C^{41}	7.2(12)	C^6	C^5	C^4	C^3	-4(2)
C^{44}	C^{35}	C^{36}	C^{37}	-160.3(7)	C^2	C^1	C^6	C^5	-0.3(16)
C^{44}	C^{35}	C^{36}	C^{41}	14.7(10)	C^2	C^1	B^1	N^1	62.9(12)
C^{44}	C^{35}	C^{27}	C^{26}	17.4(10)	C^2	C^1	B^1	C^7	-119.3(9)
C^{44}	C^{35}	C^{27}	C^{28}	-152.9(7)	B^1	C^1	C^6	C^5	-178.4(11)
C^{17}	C^{16}	C^7	C^8	170.5(7)	B^1	C^1	C^2	C^3	177.1(10)
C^{17}	C^{16}	C^7	B^1	-14.1(10)	B^2	N^2	C^{26}	C^{27}	-14.2(12)
C^{17}	C^{16}	C^{15}	C^{10}	-169.4(6)	B^2	N^2	C^{26}	C^{25}	162.0(8)
C^{17}	C^{16}	C^{15}	C^{14}	16.9(11)	B^2	C^{29}	C^{30}	C^{31}	-179.3(9)
C^{17}	C^{18}	N^1	B^1	-6.6(13)	B^2	C^{29}	C^{34}	C^{33}	179.8(8)
C^{17}	C^{18}	C^{19}	C^{20}	7.8(14)	C^4	C^5	C^6	C^1	3(2)

Table 7 Hydrogen Atom Coordinates (Å×10^4) and Isotropic Displacement Parameters (Å^2×10^3) for **7l**.

**Atom**	***x***	***y***	***z***	**U(eq)**
H^{2A}	2212.14	5750.37	1556.57	46
H^{8}	9445.11	3068.34	2614.19	39
H^{37}	5698.14	3308.73	3812.28	47
H^{43}	1736.19	6462.49	4554.4	42
H^{1}	6309.57	4102.08	286.77	54
H^{30}	2759.17	7461.7	1755.57	56
H^{22}	7091.84	7033.99	2322.31	48
H^{34}	-246.84	6987.41	3686.66	47
H^{39}	6229.74	3344.1	6423.9	62
H^{40}	4371.85	4534.73	6532.19	56
H^{11}	9278.32	4761.36	5209.21	44
H^{38}	6981.6	2803.07	5043.73	54
H^{20}	4691.24	7209.63	77.03	62
H^{28}	3743.93	2686.56	3309.93	44
H^{13}	6154.48	6850.38	4811.32	50
H^{12}	7910.79	6061.92	5719.91	50
H^{14}	5980.9	6465.16	3355.96	43
H^{33}	-1143.33	8569.92	3537.58	65
H^{19}	5059.75	5560.24	-156.05	60
H^{25}	2332.08	4269.83	752.85	58
H^{31}	1887.83	9024.89	1628.62	70
H^{5}	8107.28	451.76	1347.99	118
H^{32}	-60.33	9576.09	2514.73	75
H^{6}	7262.93	1920.13	1874.42	81
H^{24}	2681.35	2610.48	743.14	64
H^{2}	9346.48	3246.79	-126.76	71
H^{4}	9726.66	387.54	147.99	124
H^{3}	10275.09	1768.59	-626.19	107

X-RAY CRYSTALLOGRAPHIC DATA FOR 9

Crystallographic data are presented in Tables 8-14. Colourless crystals of **9** were grown by slow evaporation at room temperature from a solution of the compound in cyclohexane. A single crystal of **9** was coated in high-vacuum grease and mounted on a glass fibre. X-ray measurements were made using a Bruker D8 VENTURE PhtotonIII area-detector diffractometer with Cu-K_{α} radiation (α = 1.54 Å). Absorption corrections were applied, based on multiple and symmetry-equivalent measurements. The structure was solved by ShelXT structure solution program using Intrinsic Phasing and refined with the XL refinement package using Least Squares minimisation.[6] All non-hydrogen atoms were assigned anisotropic displacement parameters and refined without positional constraints. H1 was also assigned anisotropic displacement parameters and all other hydrogen atoms were constrained to ideal geometries and refined with fixed isotropic displacement parameters. Refinement proceeded smoothly to give the residuals shown in Table 8.

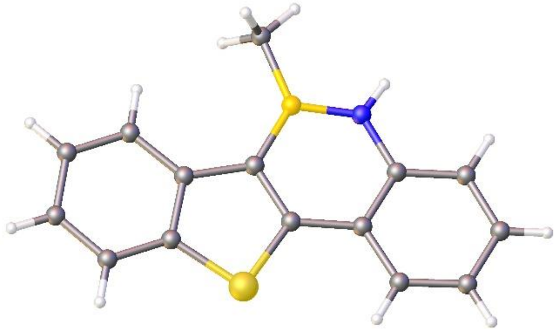

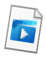

ESP6700F1.mp4

A colorless needle-like specimen of $C_{15}H_{14}BNS$, approximate dimensions 0.010 mm x 0.030 mm x 0.150 mm, was used for the X-ray crystallographic analysis. The X-ray intensity data were measured (λ = 1.54184 Å).

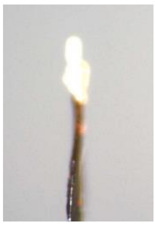

[6] a) Sheldrick, G.M. (2015). Acta Cryst A71, 3-8; APEX3 Version 2016.7 (Bruker AXS Inc.) Bruker Instrument Service vV6.2.10. b) SAINT integration software, SAINT V8.38A (Bruker AXS Inc., 2017). c) SADABS-2016/2 - Bruker AXS area detector scaling and absorption correction (Sheldrick, Bruker AXS Inc.). d) *SHELXTL program system version 6.1;* XPREP Version 2013/3 (Sheldrick, Bruker AXS Inc.) XS Version 2013/1 (George M. Sheldrick, *Acta Cryst.* (2008). A**64**, 112-122). e) *International Tables for Crystallography*, Kluwer, Dordrecht, 1992, vol. C

Data collection details for 9.

Axis	dx/mm	2θ/°	ω/°	ϕ/°	χ/°	Width/°	Frames	Time/s	Wavelength/Å	Voltage/kV	Current/mA	T/K
Phi	39.908	86.73	350.65	0.00	24.00	0.90	400	10.00	1.54184	50	1.1	180
Omega	39.908	86.73	-12.84	80.00	61.50	0.90	118	10.00	1.54184	50	1.1	180
Phi	39.908	86.73	84.80	0.00	-44.50	0.90	400	10.00	1.54184	50	1.1	180
Omega	39.908	86.73	-12.84	-120.00	61.50	0.90	118	10.00	1.54184	50	1.1	180
Omega	39.908	-18.14	-124.02	180.00	44.50	0.90	120	10.00	1.54184	50	1.1	180
Omega	39.908	-56.73	-132.80	270.00	44.50	0.90	87	10.00	1.54184	50	1.1	180
Omega	39.908	-41.73	-132.80	0.00	44.50	0.90	104	10.00	1.54184	50	1.1	180
Omega	39.908	-48.14	-54.69	-54.00	-61.50	0.90	124	10.00	1.54184	50	1.1	180
Omega	39.908	86.73	-12.84	-40.00	61.50	0.90	118	10.00	1.54184	50	1.1	180
Omega	39.908	-41.73	-132.80	90.00	44.50	0.90	104	10.00	1.54184	50	1.1	180
Omega	39.908	86.73	-12.84	160.00	61.50	0.90	118	10.00	1.54184	50	1.1	180
Omega	39.908	-56.73	-132.80	180.00	44.50	0.90	87	10.00	1.54184	50	1.1	180
Omega	39.908	86.73	-12.84	40.00	61.50	0.90	118	10.00	1.54184	50	1.1	180
Omega	39.908	-18.14	-124.02	270.00	44.50	0.90	120	10.00	1.54184	50	1.1	180
Omega	39.908	71.73	70.51	0.00	-44.50	0.90	119	10.00	1.54184	50	1.1	180
Omega	39.908	-41.73	-132.80	180.00	44.50	0.90	104	10.00	1.54184	50	1.1	180
Omega	39.908	71.73	70.51	180.00	-44.50	0.90	119	10.00	1.54184	50	1.1	180
Omega	39.908	86.73	84.80	160.00	-44.50	0.90	99	10.00	1.54184	50	1.1	180
Omega	39.908	71.73	70.51	90.00	-44.50	0.90	119	10.00	1.54184	50	1.1	180
Omega	39.908	-18.14	-124.02	90.00	44.50	0.90	120	10.00	1.54184	50	1.1	180
Phi	39.908	71.73	69.80	0.00	-44.50	0.90	400	10.00	1.54184	50	1.1	180

A total of 3216 frames were collected. The total exposure time was 8.93 hours. The frames were integrated with the Bruker SAINT software package using a narrow-frame algorithm. The integration of the data using an orthorhombic unit cell yielded a total of 52769 reflections to a maximum θ angle of 59.03° (0.90 Å resolution). The final cell constants of a = 5.4361(3) Å, b = 14.3360(8) Å, c = 32.5378(18) Å, volume = 2535.7(2) Å^3, are based upon the refinement of the XYZ-centroids of 9995 reflections above 20 σ(I) with 5.432° < 2θ < 117.3°. Data were corrected for absorption effects using the Multi-Scan method (SADABS). The ratio of minimum to maximum apparent transmission was 0.821.

Table 8 Crystal data and structure refinement for **9**.

Identification code	ESP6700F1
Empirical formula	$C_{15}H_{12}BNS$
Formula weight	249.13
Temperature/K	180.0
Crystal system	orthorhombic
Space group	Pbca
a/Å	5.4361(3)
b/Å	14.3360(8)
c/Å	32.5378(18)
α/°	90
β/°	90
γ/°	90
Volume/Å^3	2535.7(2)
Z	8
ρ_{calc}g/cm^3	1.305
μ/mm^{-1}	2.064
F(000)	1040.0
Crystal size/mm^3	0.15 × 0.03 × 0.01
Radiation	CuKα (λ = 1.54178)
2Θ range for data collection/°	5.432 to 118.142
Index ranges	-6 ≤ h ≤ 6, -15 ≤ k ≤ 15, -36 ≤ l ≤ 36
Reflections collected	46688
Independent reflections	1809 [R_{int} = 0.0532, R_{sigma} = 0.0165]
Data/restraints/parameters	1809/0/168
Goodness-of-fit on F^2	1.130
Final R indexes [I>=2σ (I)]	R_1 = 0.0475, wR_2 = 0.1105
Final R indexes [all data]	R_1 = 0.0682, wR_2 = 0.1362
Largest diff. peak/hole / e Å^{-3}	0.17/-0.26

Table 9 Fractional Atomic Coordinates (×10[4]) and Equivalent Isotropic Displacement Parameters ($Å^2$×10[3]) for **9**. U_{eq} is defined as 1/3 of of the trace of the orthogonalised U_{IJ} tensor.

Atom	*x*	*y*	*z*	U(eq)
S1	7588.3(16)	6149.9(6)	5895.2(3)	77.4(3)
N1	1279(6)	7928(2)	6328.5(9)	77.0(8)
C2	5485(5)	5826(2)	6601.6(9)	64.7(8)
C1	4186(5)	6620(2)	6428.2(9)	64.5(8)
C9	4302(6)	7622(2)	5803.7(9)	70.1(8)
C14	2296(6)	8144(2)	5947.0(10)	70.5(8)
C8	5157(6)	6854(2)	6050.2(9)	67.4(8)
C7	7409(6)	5507(2)	6346.6(9)	69.6(8)
C3	5115(6)	5363(2)	6975.3(10)	74.1(9)
C10	5404(7)	7889(3)	5430.9(11)	86.1(10)
C4	6602(7)	4630(2)	7083.6(11)	80.7(9)
C6	8912(7)	4768(2)	6456.8(11)	81.8(10)
C15	731(7)	7083(3)	7019.6(11)	86.8(10)
C13	1403(8)	8885(3)	5713.9(12)	89.2(11)
C5	8500(7)	4334(2)	6826.3(12)	87.2(10)
C12	2538(9)	9123(3)	5350.7(13)	100.9(13)
C11	4552(9)	8630(3)	5210.5(12)	98.7(12)
B1	2056(7)	7201(3)	6594.3(12)	68.4(9)

Table 10 Anisotropic Displacement Parameters ($Å^2$×10[3]) for **9**. The Anisotropic displacement factor exponent takes the form: $-2\pi^2[h^2a^{*2}U_{11}+2hka^*b^*U_{12}+\ldots]$.

Atom	U_{11}	U_{22}	U_{33}	U_{23}	U_{13}	U_{12}
S1	78.7(6)	79.8(6)	73.7(6)	-10.9(4)	10.7(4)	2.2(4)
N1	65.9(18)	75.1(18)	90(2)	-15.9(16)	-2.1(15)	7.1(15)
C2	59.6(17)	65.3(18)	69.1(18)	-13.4(15)	-3.0(15)	-4.8(15)
C1	61.4(18)	61.8(17)	70.5(18)	-12.6(14)	-3.5(15)	-4.9(15)
C9	72(2)	68.0(19)	70.4(19)	-11.0(15)	-10.9(16)	-7.7(17)
C14	74(2)	62.8(19)	75(2)	-10.2(16)	-14.2(17)	-6.0(16)
C8	67.4(19)	67.0(18)	67.8(18)	-15.1(15)	-2.8(15)	-6.5(15)
C7	68.9(19)	67.7(19)	72.3(19)	-16.9(15)	0.9(16)	-4.8(16)
C3	71(2)	76(2)	75(2)	-7.6(17)	0.8(17)	-3.8(17)
C10	90(2)	91(2)	77(2)	-4.7(19)	-5.6(19)	-8(2)
C4	87(2)	75(2)	81(2)	-0.2(17)	-5.0(19)	-2(2)
C6	78(2)	74(2)	93(2)	-16.6(19)	4.1(19)	10.2(18)
C15	77(2)	90(2)	94(2)	-11.4(19)	10.0(19)	6.8(19)
C13	90(3)	85(2)	93(3)	-7(2)	-22(2)	4(2)
C5	89(3)	74(2)	98(3)	-5(2)	-10(2)	8(2)
C12	116(3)	87(3)	100(3)	11(2)	-38(3)	-6(2)
C11	111(3)	104(3)	81(2)	12(2)	-16(2)	-14(3)
B1	63(2)	63(2)	80(2)	-13.2(18)	-3.8(18)	-5.4(18)

Table 11 Bond Lengths for **9**.

Atom	Atom	Length/Å	Atom	Atom	Length/Å
S1	C8	1.737(3)	C9	C10	1.406(5)
S1	C7	1.737(3)	C14	C13	1.394(5)
N1	C14	1.394(4)	C7	C6	1.385(4)
N1	B1	1.419(5)	C3	C4	1.372(4)
C2	C1	1.453(4)	C10	C11	1.363(5)
C2	C7	1.411(4)	C4	C5	1.395(5)
C2	C3	1.399(4)	C6	C5	1.373(5)
C1	C8	1.380(4)	C15	B1	1.569(5)
C1	B1	1.526(5)	C13	C12	1.376(6)
C9	C14	1.402(4)	C12	C11	1.381(6)
C9	C8	1.440(4)			

Table 12 Bond Angles for **9.**

Atom	Atom	Atom	Angle/°	Atom	Atom	Atom	Angle/°
C7	S1	C8	91.14(15)	C9	C8	S1	121.9(2)
C14	N1	B1	126.0(3)	C2	C7	S1	111.5(2)
C7	C2	C1	112.7(3)	C6	C7	S1	126.3(3)
C3	C2	C1	129.7(3)	C6	C7	C2	122.2(3)
C3	C2	C7	117.6(3)	C4	C3	C2	120.1(3)
C2	C1	B1	131.2(3)	C11	C10	C9	121.4(4)
C8	C1	C2	110.5(3)	C3	C4	C5	121.0(3)
C8	C1	B1	118.2(3)	C5	C6	C7	118.5(3)
C14	C9	C8	118.3(3)	C12	C13	C14	120.0(4)
C14	C9	C10	118.3(3)	C6	C5	C4	120.5(3)
C10	C9	C8	123.4(3)	C13	C12	C11	120.8(4)
N1	C14	C9	119.1(3)	C10	C11	C12	119.7(4)
N1	C14	C13	121.0(3)	N1	B1	C1	114.3(3)
C13	C14	C9	119.8(3)	N1	B1	C15	118.7(3)
C1	C8	S1	114.1(2)	C1	B1	C15	127.0(3)
C1	C8	C9	124.0(3)				

Table 13 Torsion Angles for **9**.

A	B	C	D	Angle/°	A	B	C	D	Angle/°
S1	C7	C6	C5	-179.6(3)	C8	C9	C14	N1	2.9(4)
N1	C14	C13	C12	175.8(3)	C8	C9	C14	C13	-179.2(3)
C2	C1	C8	S1	0.0(3)	C8	C9	C10	C11	-179.1(3)
C2	C1	C8	C9	-178.7(3)	C7	S1	C8	C1	-0.7(2)

Table 13 Torsion Angles for **9**.

A	B	C	D	Angle/°	A	B	C	D	Angle/°
C2	C1	B1	N1	-179.7(3)	C7	S1	C8	C9	178.1(3)
C2	C1	B1	C15	1.5(5)	C7	C2	C1	C8	0.9(4)
C2	C7	C6	C5	0.2(5)	C7	C2	C1	B1	-178.5(3)
C2	C3	C4	C5	0.1(5)	C7	C2	C3	C4	0.2(4)
C1	C2	C7	S1	-1.4(3)	C7	C6	C5	C4	0.2(5)
C1	C2	C7	C6	178.8(3)	C3	C2	C1	C8	179.9(3)
C1	C2	C3	C4	-178.7(3)	C3	C2	C1	B1	0.5(5)
C9	C14	C13	C12	-2.1(5)	C3	C2	C7	S1	179.5(2)
C9	C10	C11	C12	-1.1(6)	C3	C2	C7	C6	-0.4(4)
C14	N1	B1	C1	-0.7(5)	C3	C4	C5	C6	-0.3(5)
C14	N1	B1	C15	178.2(3)	C10	C9	C14	N1	-175.9(3)
C14	C9	C8	S1	178.7(2)	C10	C9	C14	C13	2.0(4)
C14	C9	C8	C1	-2.7(4)	C10	C9	C8	S1	-2.6(4)
C14	C9	C10	C11	-0.4(5)	C10	C9	C8	C1	176.0(3)
C14	C13	C12	C11	0.6(6)	C13	C12	C11	C10	1.0(6)
C8	S1	C7	C2	1.2(2)	B1	N1	C14	C9	-1.2(5)
C8	S1	C7	C6	-179.0(3)	B1	N1	C14	C13	-179.1(3)
C8	C1	B1	N1	0.9(4)	B1	C1	C8	S1	179.5(2)
C8	C1	B1	C15	-177.9(3)	B1	C1	C8	C9	0.8(4)

Table 14 Hydrogen Atom Coordinates (Å×10^4) and Isotropic Displacement Parameters (Å^2×10^3) for **9**.

Atom	*x*	*y*	*z*	U(eq)
H3	3833.05	5557.63	7154.34	89
H10	6771.02	7544.94	5330.36	103
H4	6335.14	4320.04	7337.66	97
H6	10199.81	4566.81	6280.49	98
H15A	1862.63	7257.56	7241.15	130
H15B	225.75	6432	7054.57	130
H15C	-722.59	7487.17	7028.73	130
H13	9.35	9226.92	5805.76	107
H5	9515.3	3827.03	6907.5	105
H12	1928.44	9633.5	5194.56	121
H11	5339	8806.42	4961.66	118
H1	80(60)	8260(20)	6396(10)	82(11)

PHOTOPHYSICAL DATA

Absorption spectra were recorded in a UV-VIS FLS980 Spectrophotometer (Edinburgh Instruments) equipped with a detector (200-1000 nm) that is allowed for absorbance measurements (measurement conditions for all samples: Δλexc = 0.2 nm, step = 1 nm, dwell = 0.2 s). Steady–state fluorescence measurements were carried out by using a FLS980 fluorescence spectrometer with a 450W Xe lamp as a light source and double excitation and emission monochromators. Monochromator at 400 nm was used at the excitation and emission arms. A photomultiplier tube detector cooled by a Peltier system was used for detection. For excitation and emission the measurement conditions were:
For excitation: slits widths Δλexc = 0.4 nm, Δλem = 0.8 nm step = 1 nm, dwell = 0.3 s for **5**, **7c**, **7m**, **7n**; Δλexc = 0.5 nm, Δλem = 0.5 nm step = 1 nm, dwell = 0.5 s for **7g**, **7j**, **9**; Δλexc = 0.5 nm, Δλem = 1 nm step = 1 nm, dwell = 0.3 s for **7k.**
For emission: slits widths of Δλexc = 0.8 nm, Δλem = 0.4 nm step = 1nm, dwell = 0.3 s for **5**, **7c**, **7m**, **7n**; Δλexc = 0.5 nm, Δλem = 0.5 nm step = 1 nm, dwell = 0.5 s for **7g**, **7j**, **9**; Δλexc = 1 nm, Δλem = 0.5 nm step = 1 nm, dwell = 0.3 s for **7k**.

To measure the photoluminescence quantum yield (QY) the FLS980 fluorescence spectrometer is equipped with an integrating sphere. The quantum yield can be represented in the equation below.

$$\eta_1 = \frac{\varepsilon}{\alpha} = \frac{\int L_{emission}}{\int E_{solvent} - \int E_{sample}}$$

ε is the photons emitted by the sample
η is the quantum yield
$L_{emission}$ is the luminescence emission spectrum of the sample, collected using the sphere
E_{sample} is the spectrum of the light used to excite the sample, collected using the sphere
$E_{solvent}$ is the spectrum of the light used for excitation with only the solvent in the sphere, collected using the sphere.

The measurement conditions for all samples were: slits widths Δλexc = 2 nm, Δλem = 0.2 nm step=0.2 nm, dwell = 0.3 s and 3-5 repeats. A rectangular 10 mm cuvette was used for the fluorescence measurements.

UV/Vis (Red) and Fluorescence (Blue) spectra of selected BN-PAHs 5, 7 and 9

Compound 7c

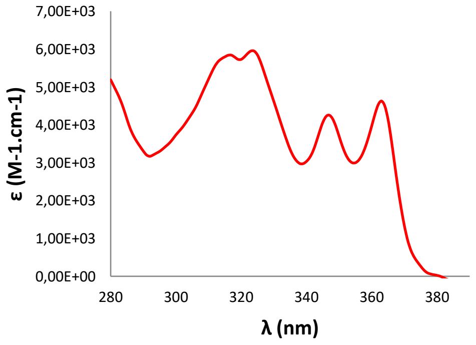

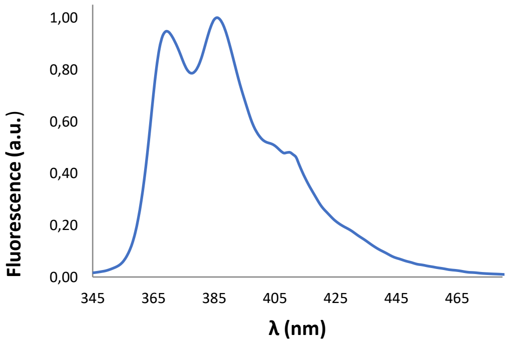

Compound 7g

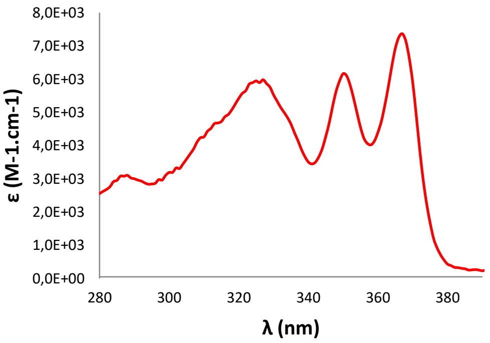

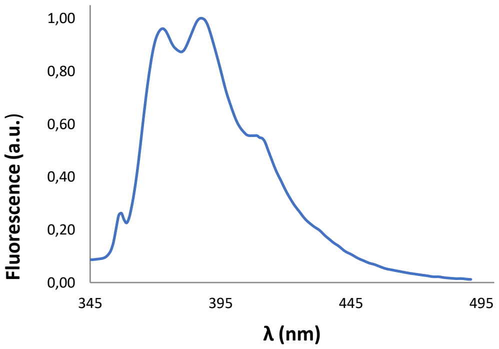

Compound 7m

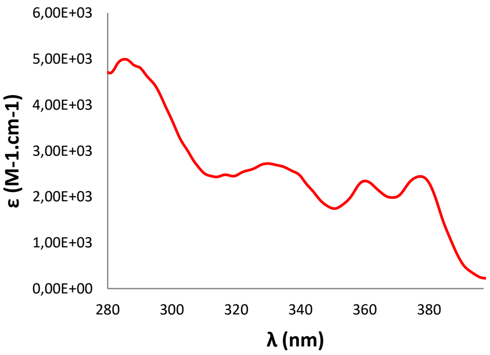

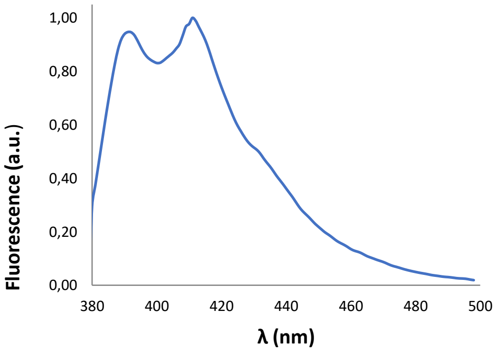

Compound 7n

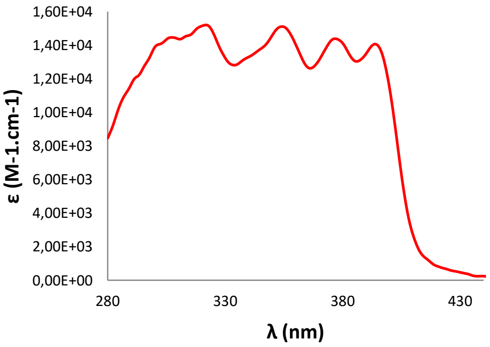

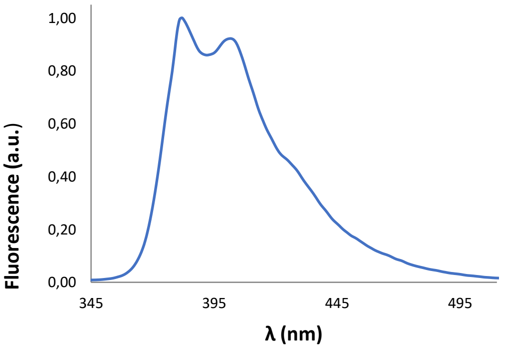

Compound 7j

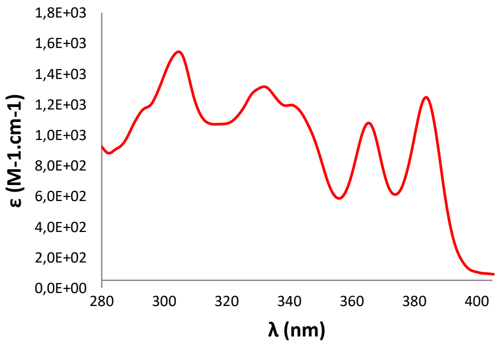

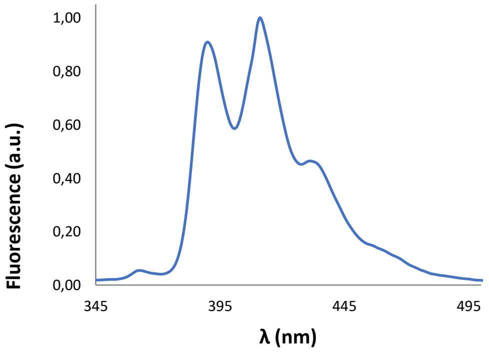

Compound 7k

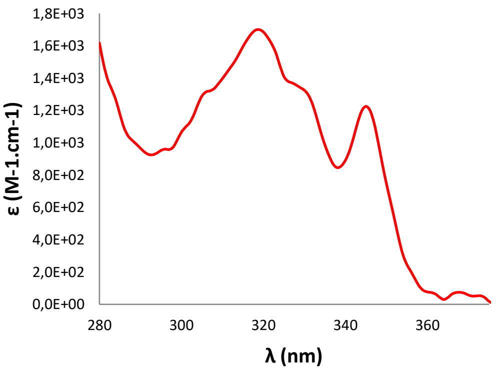

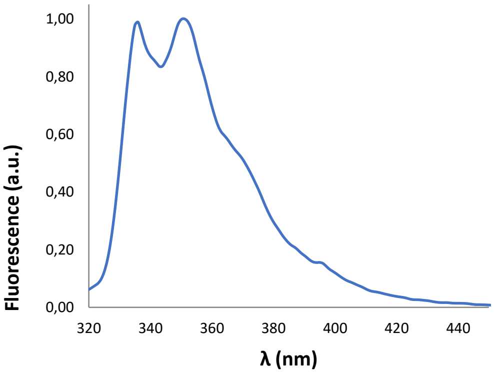

Compound 9

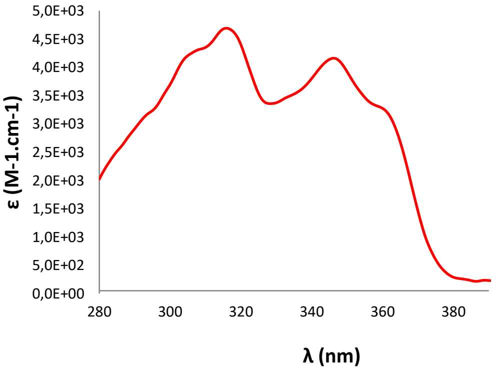

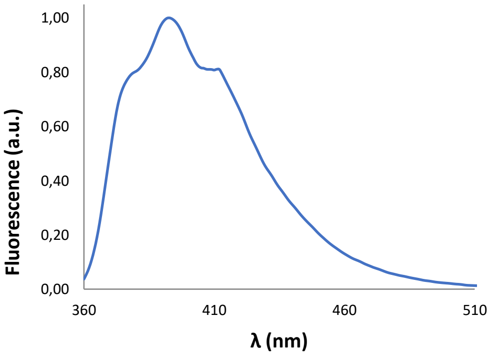

Compound 5

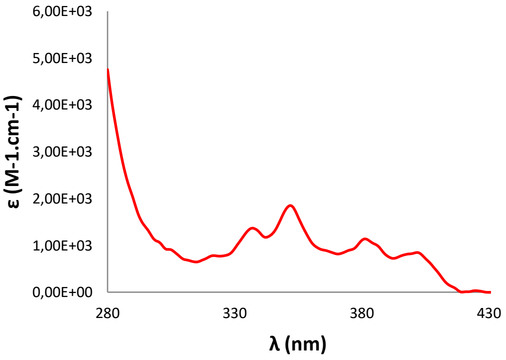

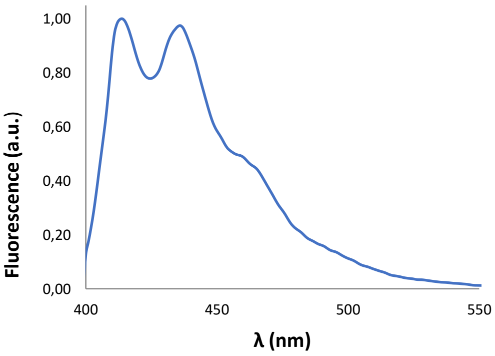

Copies of ^{1}H, ^{13}C, ^{10}B and ^{11}B-NMR spectra for novel compounds and selected gCOSY, TOCSY, NOESY, gHSQC and gHMBC spectra

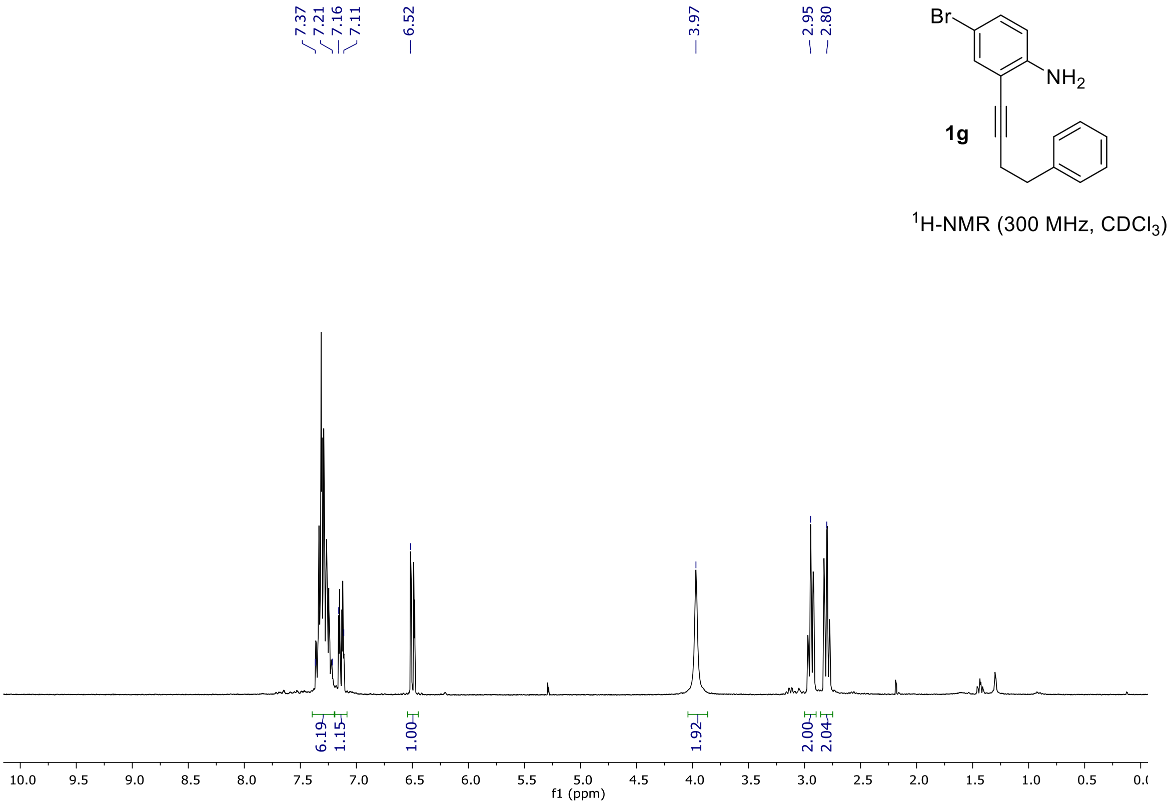
7.37
7.21
7.16
7.11
6.52
3.97
2.95
2.80
Br
NH2
1g
1H-NMR (300 MHz, CDCl3)
6.19
1.15
1.00
1.92
2.00
2.04
f1 (ppm)

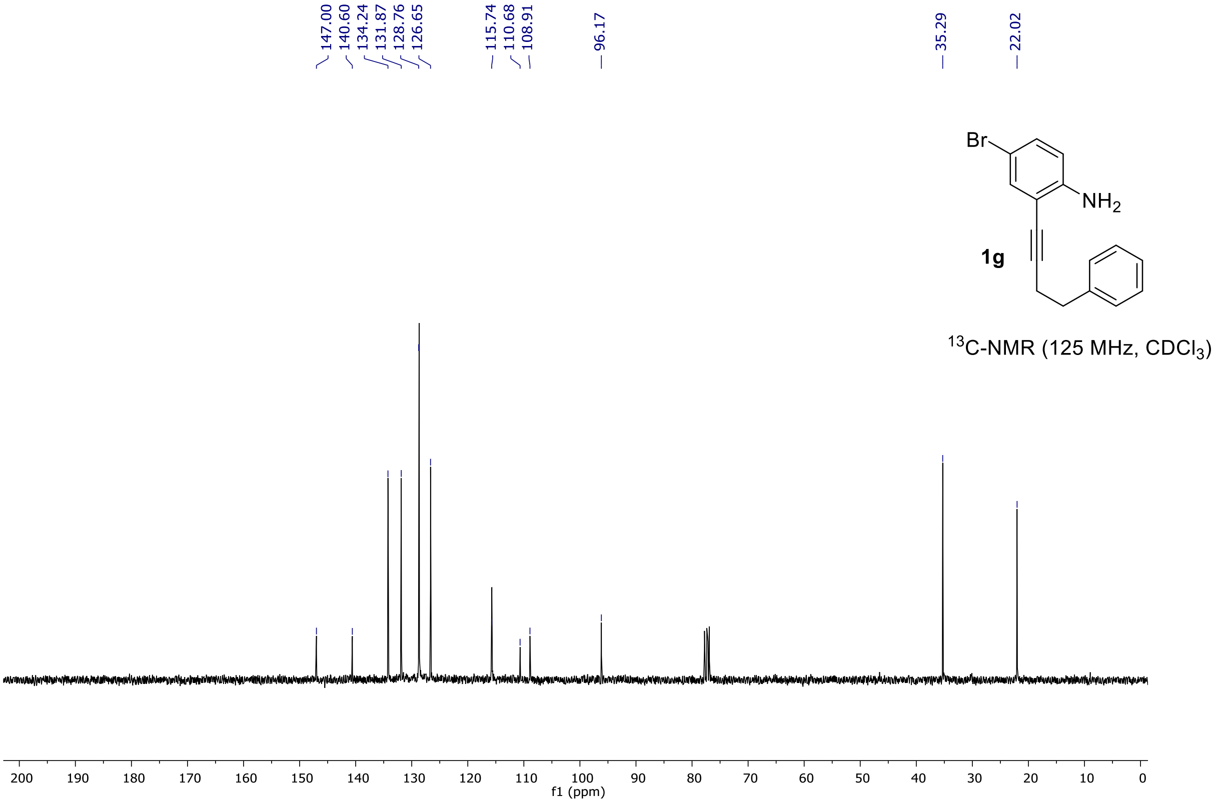
147.00
140.60
134.24
131.87
128.76
126.65
115.74
110.68
108.91
96.17
35.29
22.02
Br
NH2
1g
13C-NMR (125 MHz, CDCl3)
f1 (ppm)

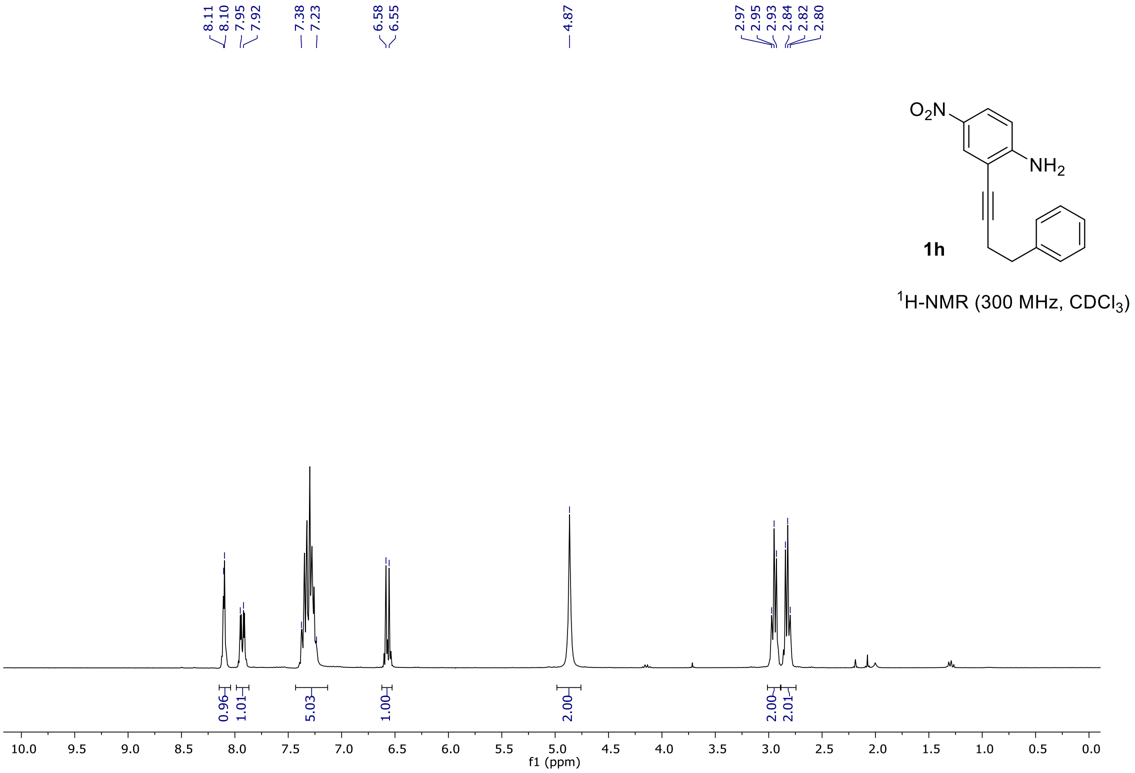
8.11
8.10
7.95
7.92
7.38
7.23
6.58
6.55
4.87
2.97
2.95
2.93
2.84
2.82
2.80
O2N
NH2
1h
1H-NMR (300 MHz, CDCl3)
0.96
1.01
5.03
1.00
2.00
2.00
2.01
10.0 9.5 9.0 8.5 8.0 7.5 7.0 6.5 6.0 5.5 5.0 4.5 4.0 3.5 3.0 2.5 2.0 1.5 1.0 0.5 0.0
f1 (ppm)

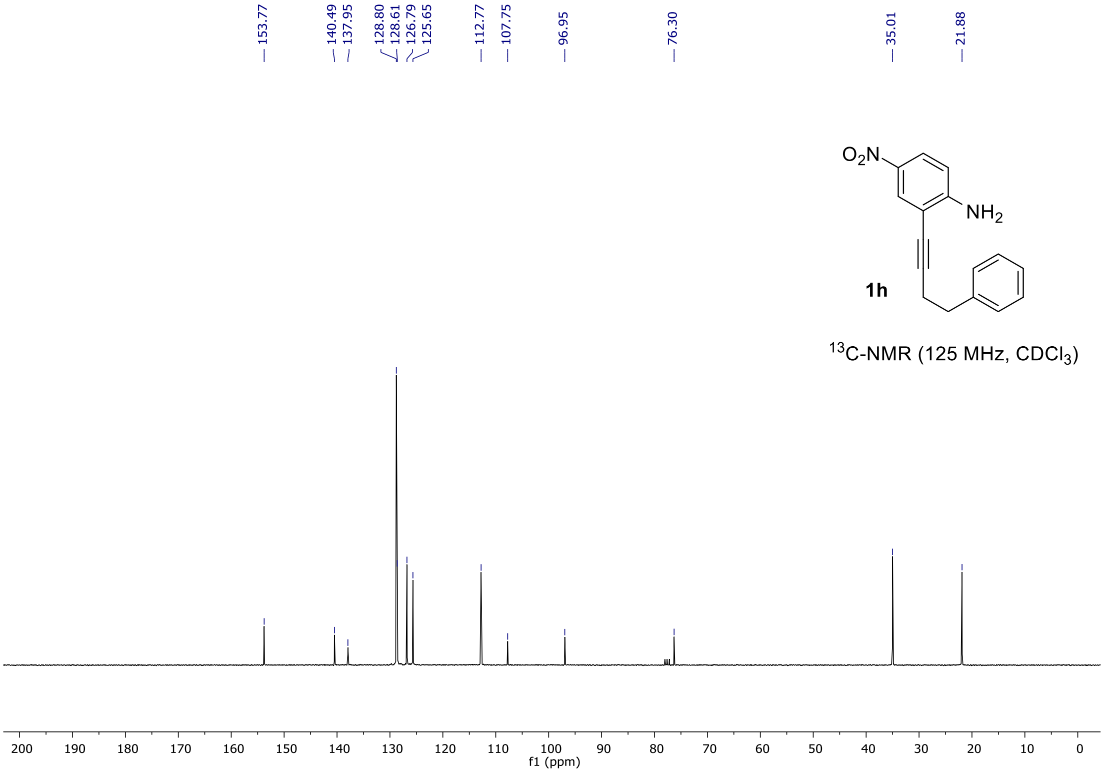
153.77
140.49
137.95
128.80
128.61
126.79
125.65
112.77
107.75
96.95
76.30
35.01
21.88
O2N
NH2
1h
13C-NMR (125 MHz, CDCl3)
200 190 180 170 160 150 140 130 120 110 100 90 80 70 60 50 40 30 20 10 0
f1 (ppm)

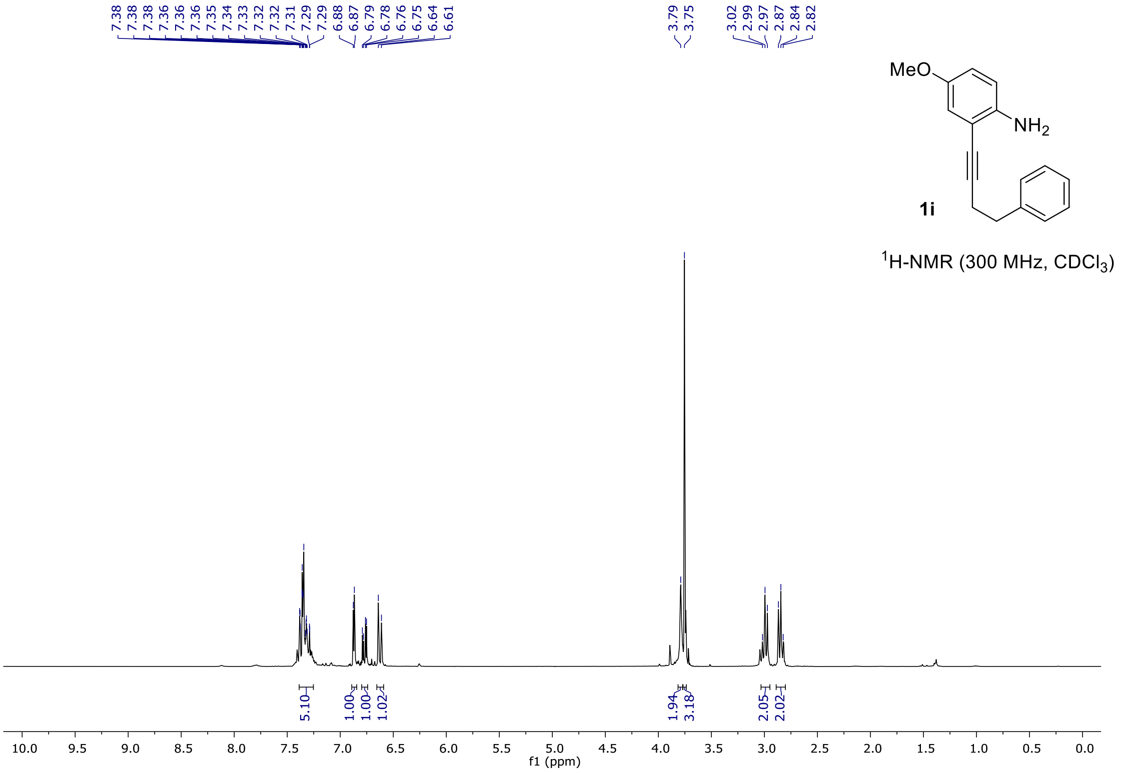
7.38
7.38
7.38
7.36
7.36
7.36
7.35
7.34
7.33
7.32
7.32
7.31
7.29
7.29
6.88
6.87
6.79
6.78
6.76
6.75
6.64
6.61
3.79
3.75
3.02
2.99
2.97
2.87
2.84
2.82
MeO
NH2
1i
1H-NMR (300 MHz, CDCl3)
5.10
1.00
1.00
1.02
1.94
3.18
2.05
2.02
f1 (ppm)

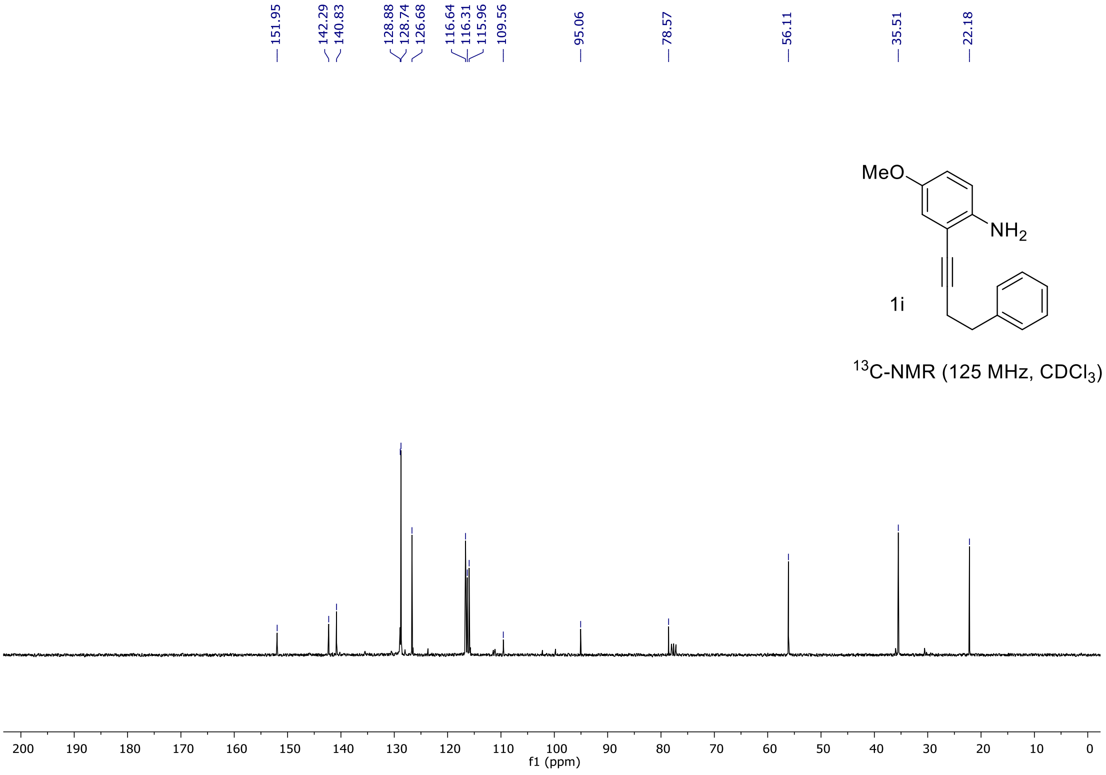
151.95
142.29
140.83
128.88
128.74
126.68
116.64
116.31
115.96
109.56
95.06
78.57
56.11
35.51
22.18
MeO
NH2
1i
13C-NMR (125 MHz, CDCl3)
f1 (ppm)

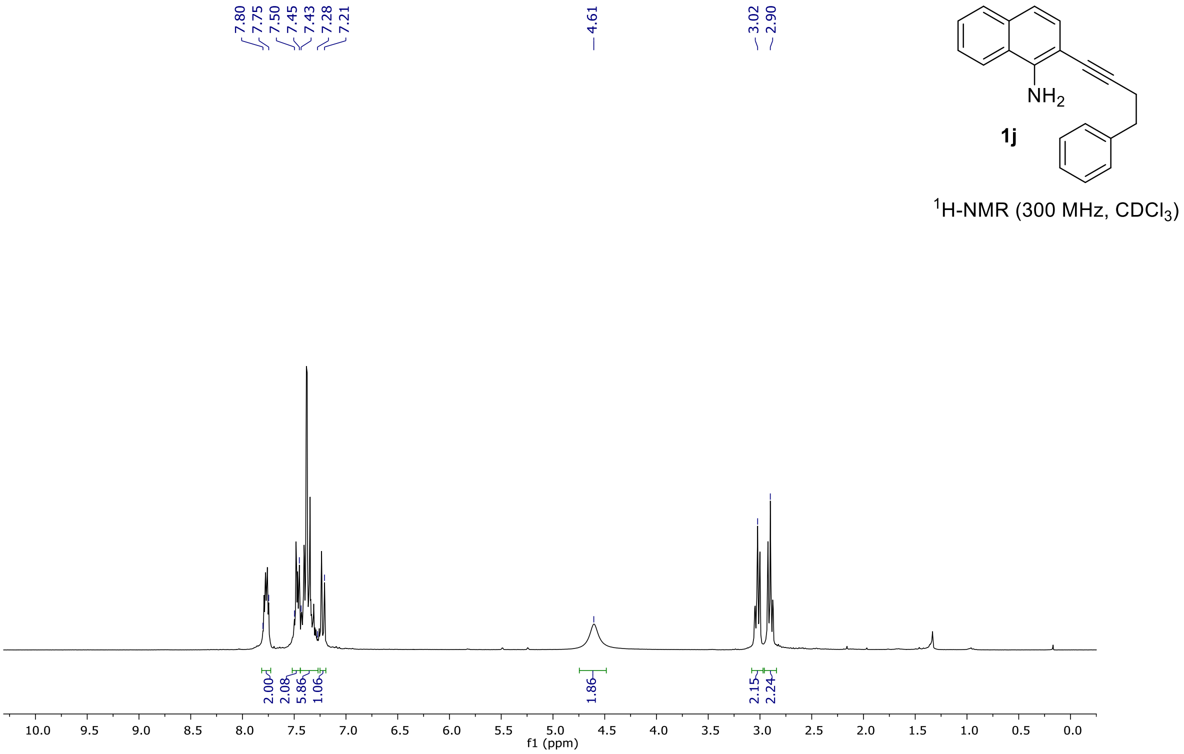

^{1}H-NMR (300 MHz, $CDCl_3$)

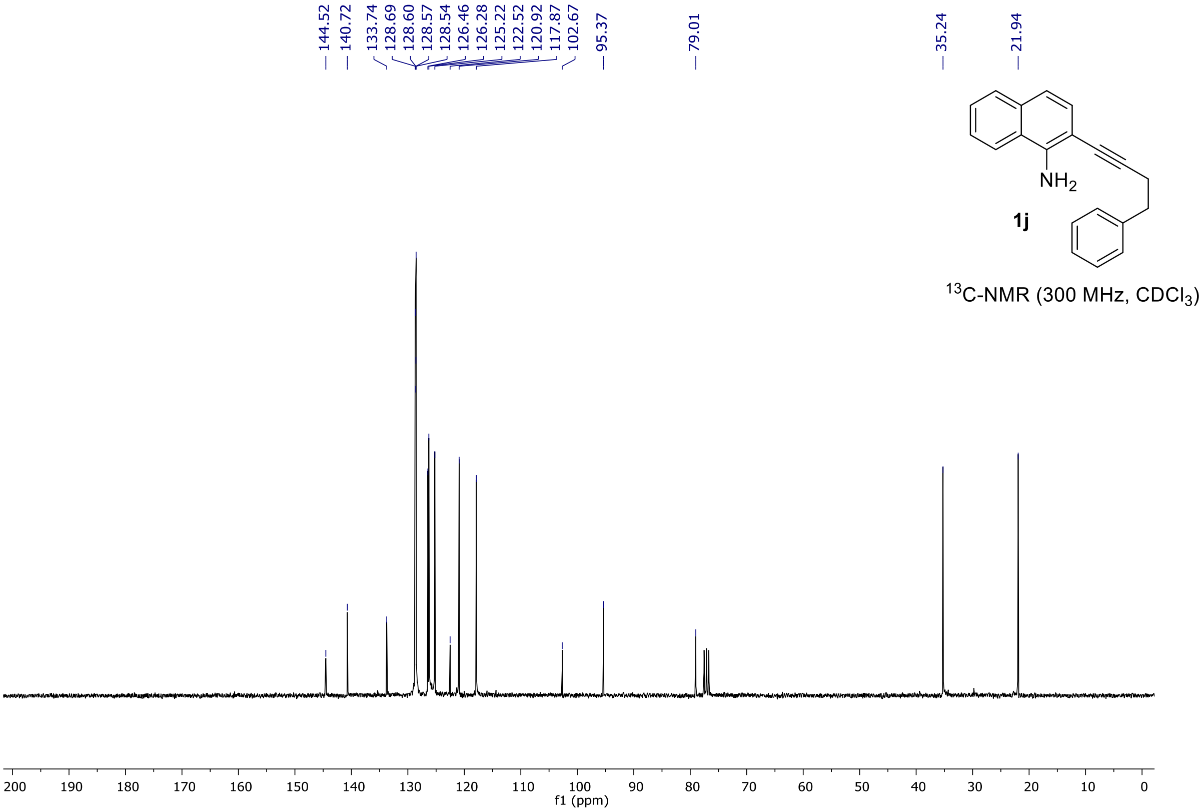

^{13}C-NMR (300 MHz, $CDCl_3$)

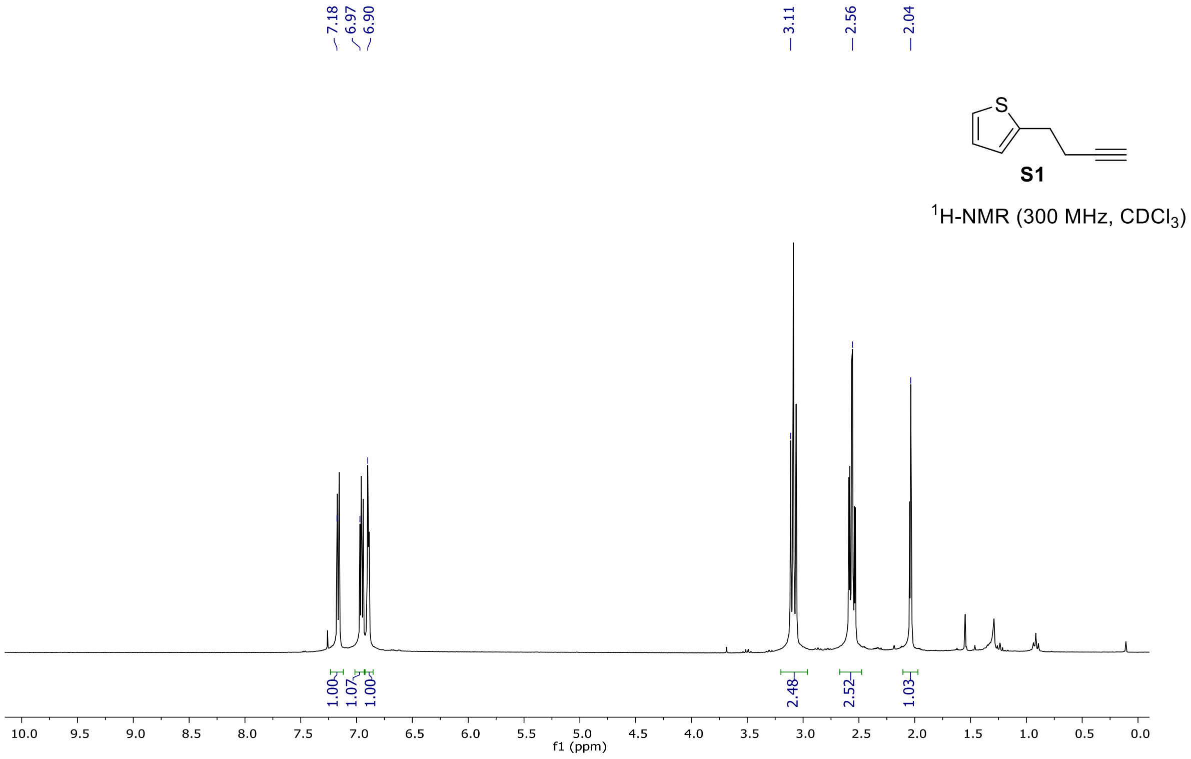
7.18
6.97
6.90
3.11
2.56
2.04
S
S1
1H-NMR (300 MHz, CDCl3)
1.00
1.07
1.00
2.48
2.52
1.03
10.0
9.5
9.0
8.5
8.0
7.5
7.0
6.5
6.0
5.5
5.0
4.5
4.0
3.5
3.0
2.5
2.0
1.5
1.0
0.5
0.0
f1 (ppm)

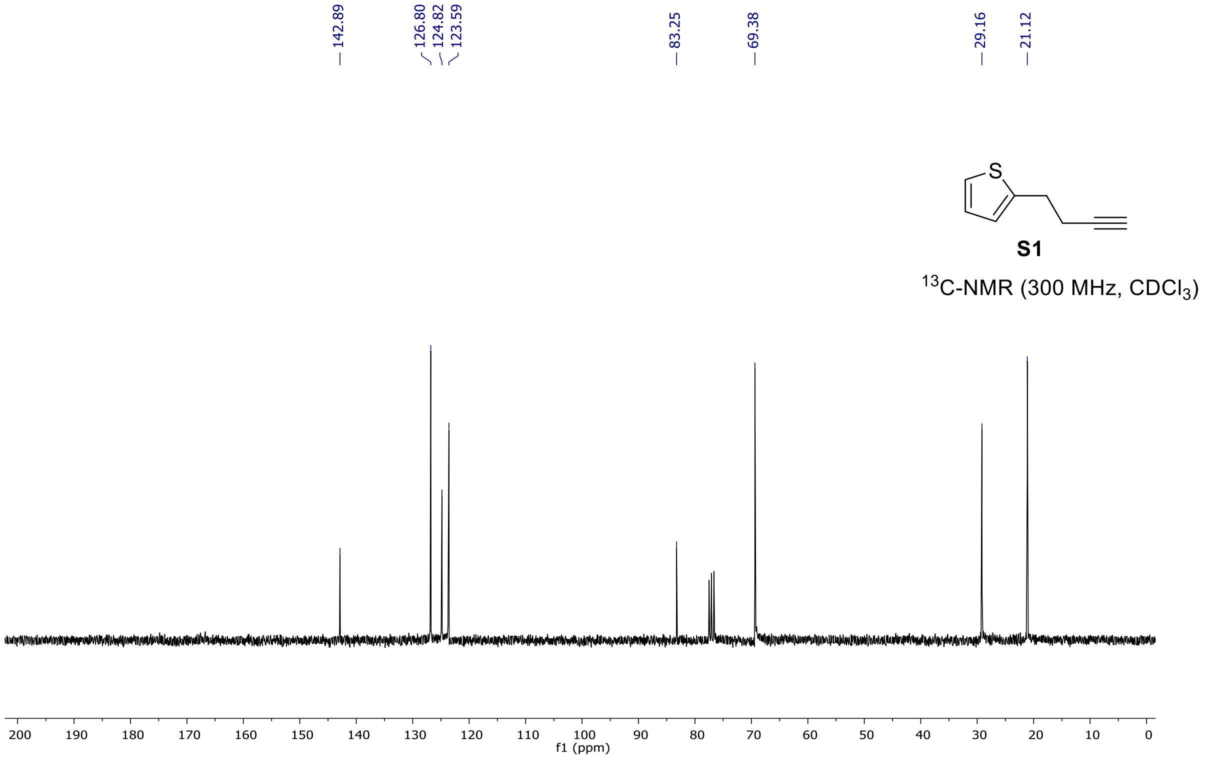
142.89
126.80
124.82
123.59
83.25
69.38
29.16
21.12
S
S1
13C-NMR (300 MHz, CDCl3)
200
190
180
170
160
150
140
130
120
110
100
90
80
70
60
50
40
30
20
10
0
f1 (ppm)

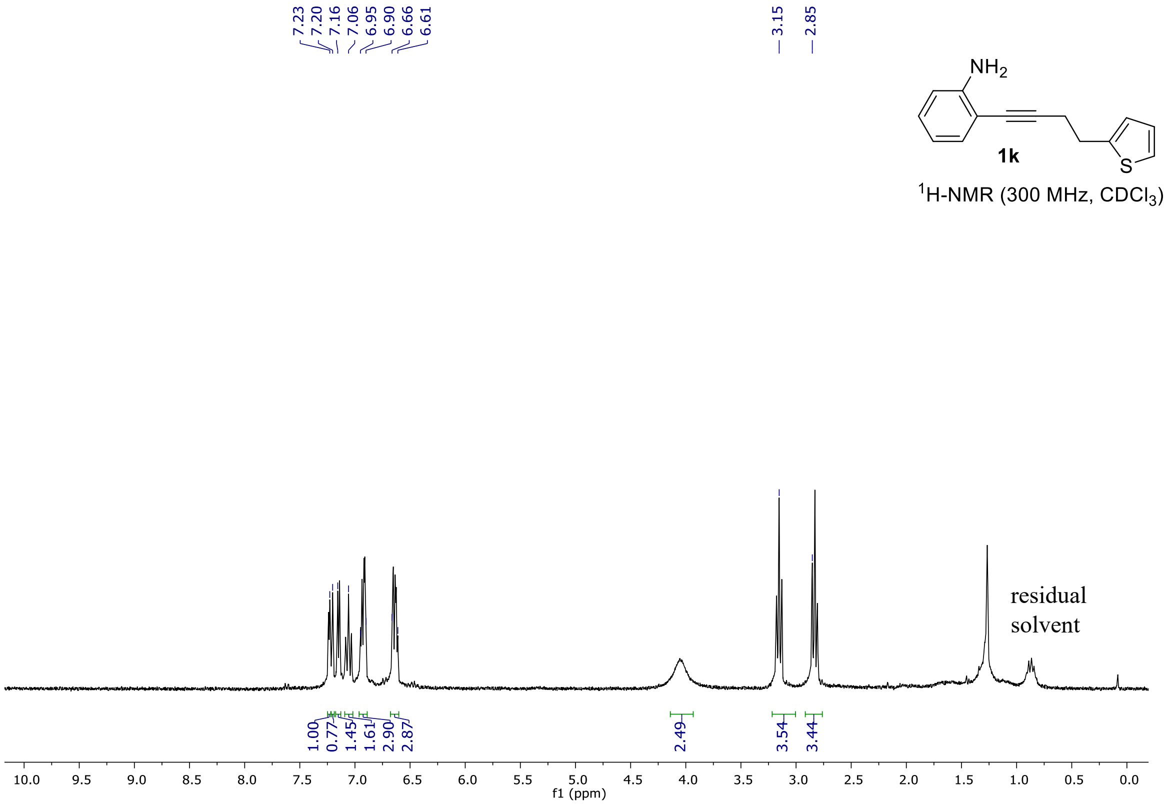

^{1}H-NMR (300 MHz, $CDCl_3$)

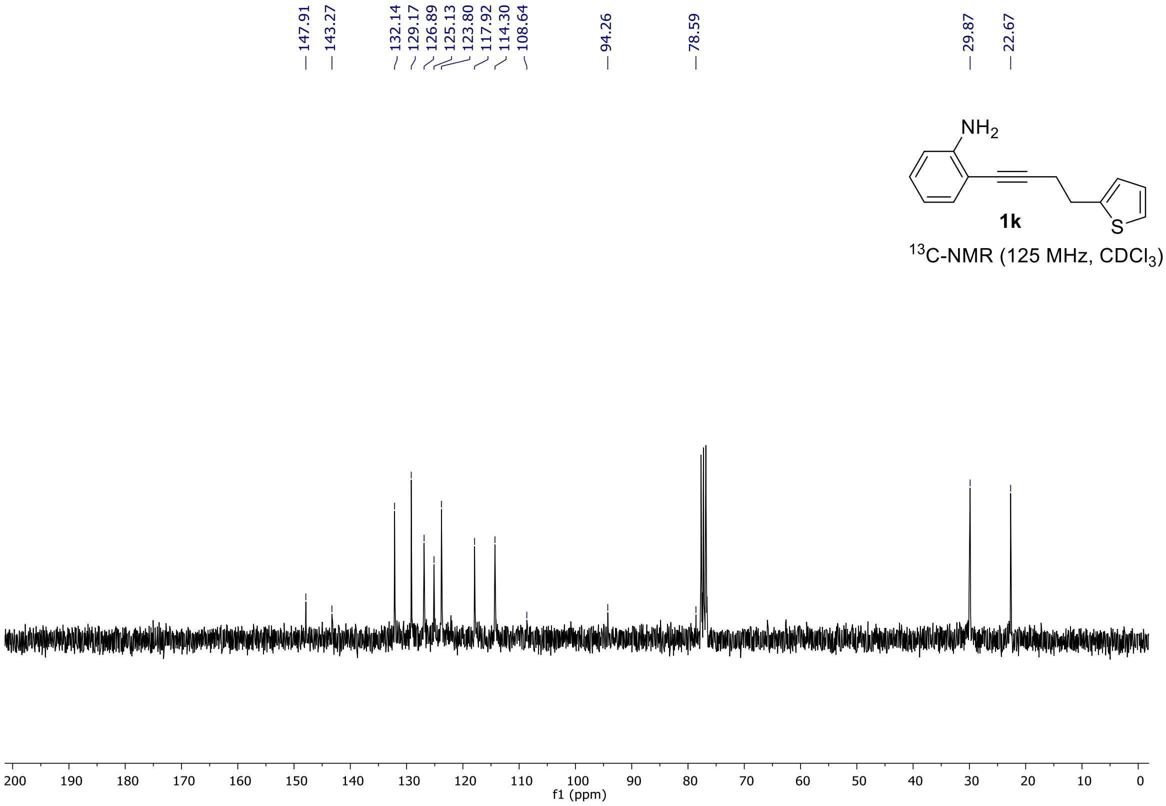

^{13}C-NMR (125 MHz, $CDCl_3$)

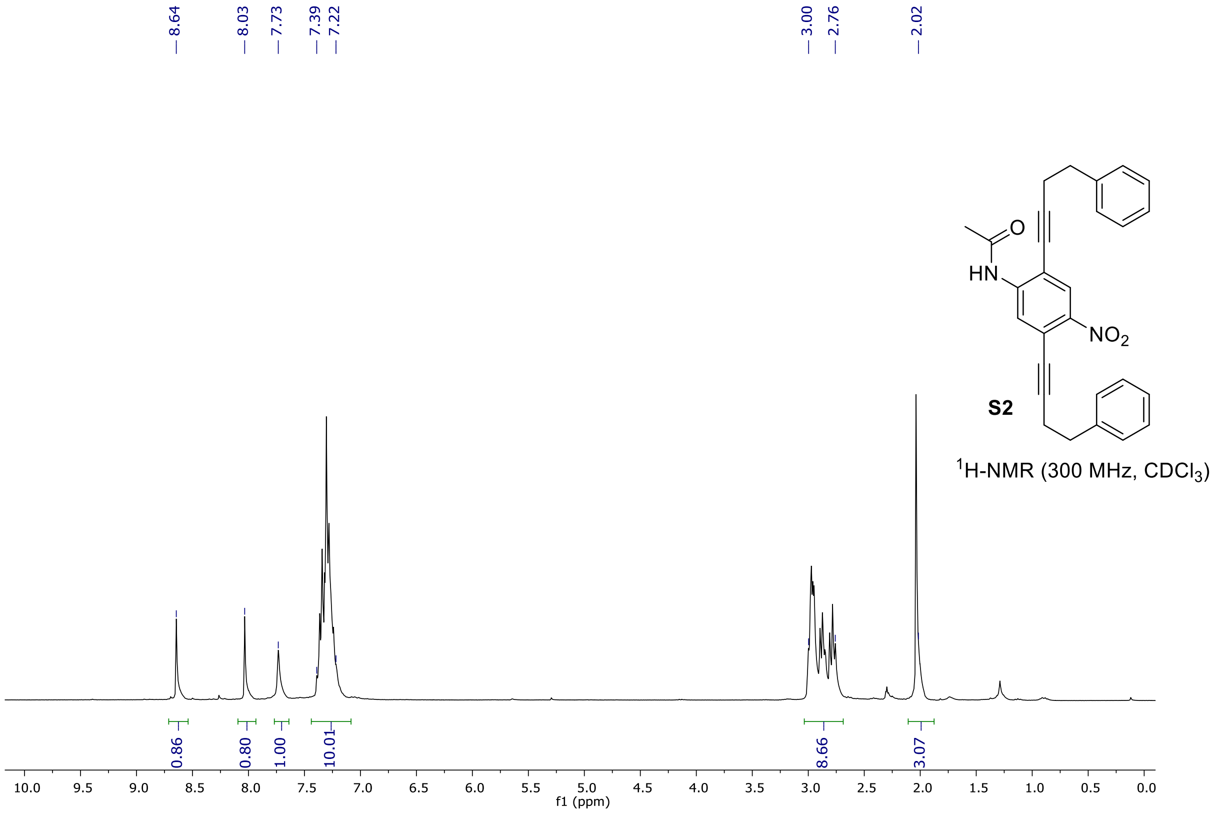
8.64
8.03
7.73
7.39
7.22
3.00
2.76
2.02
O
HN
NO2
S2
1H-NMR (300 MHz, CDCl3)
0.86
0.80
1.00
10.01
8.66
3.07
10.0 9.5 9.0 8.5 8.0 7.5 7.0 6.5 6.0 5.5 5.0 4.5 4.0 3.5 3.0 2.5 2.0 1.5 1.0 0.5 0.0
f1 (ppm)

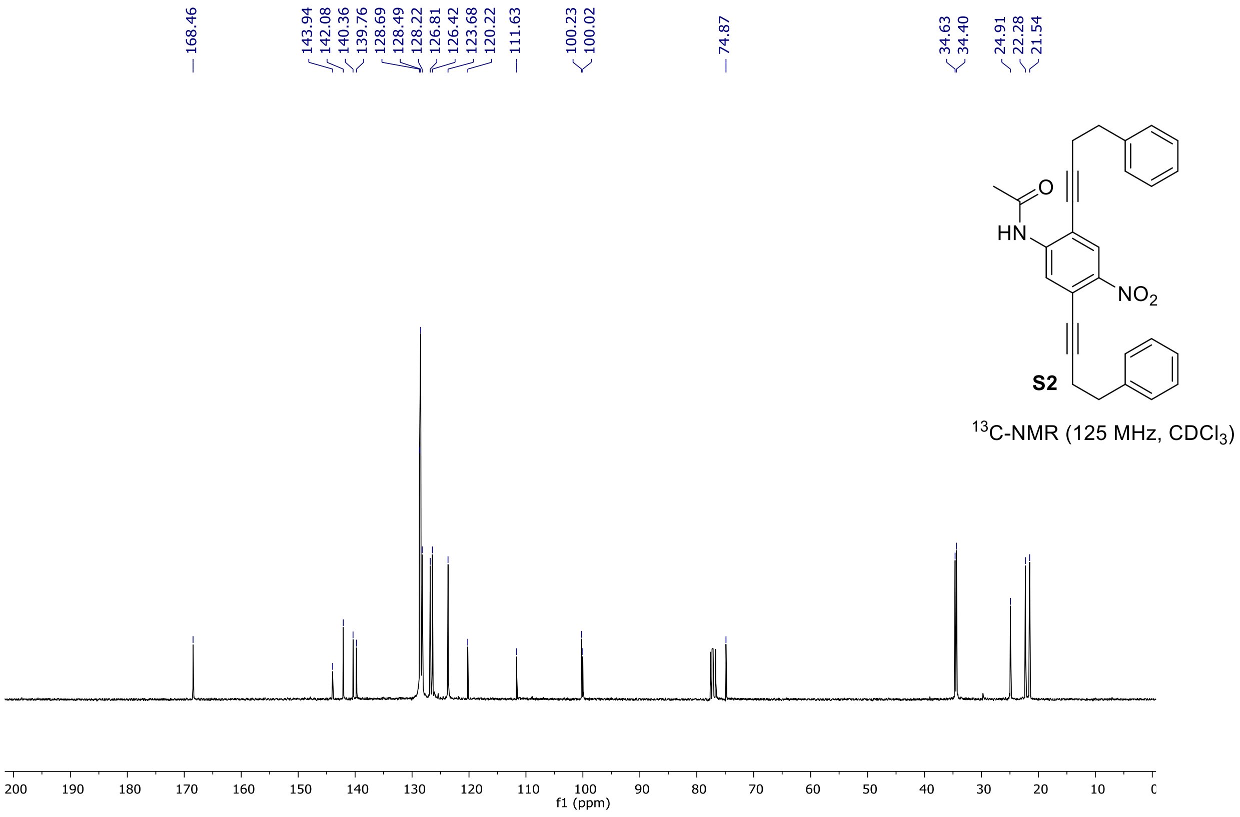
168.46
143.94
142.08
140.36
139.76
128.69
128.49
128.22
126.81
126.42
123.68
120.22
111.63
100.23
100.02
74.87
34.63
34.40
24.91
22.28
21.54
O
HN
NO2
S2
13C-NMR (125 MHz, CDCl3)
200 190 180 170 160 150 140 130 120 110 100 90 80 70 60 50 40 30 20 10
f1 (ppm)

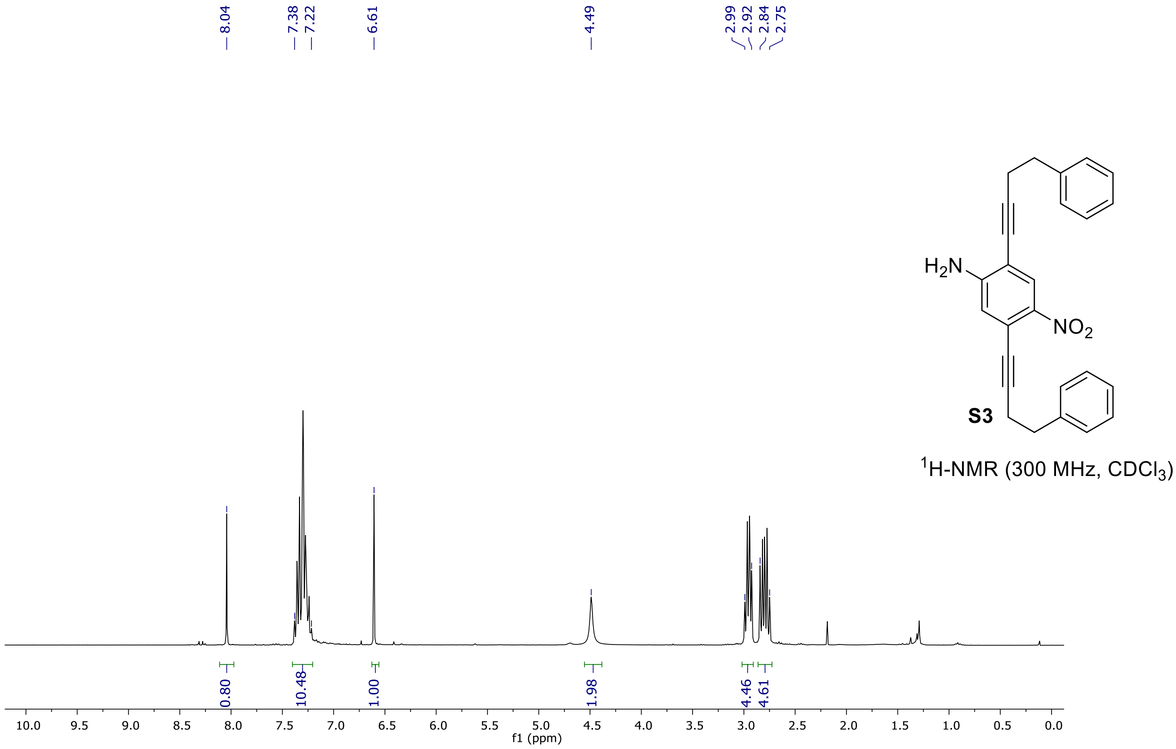

^{1}H-NMR (300 MHz, $CDCl_3$)

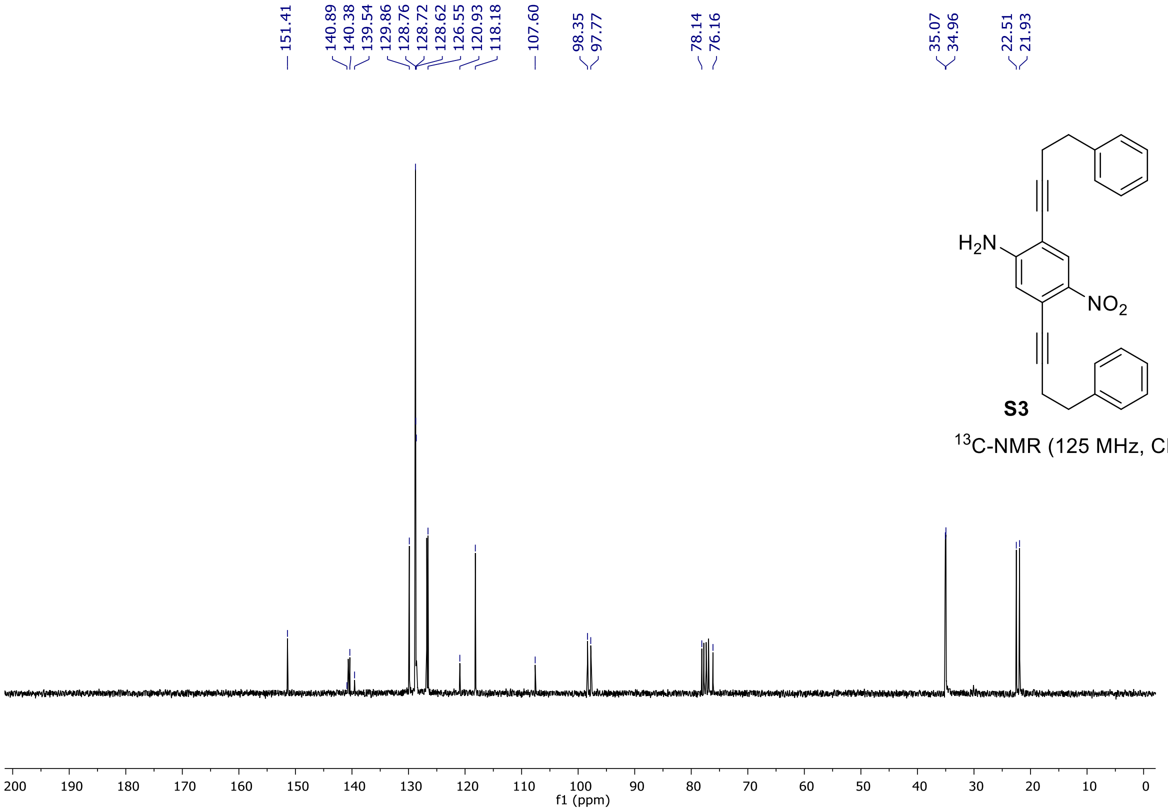

^{13}C-NMR (125 MHz, $CDCl_3$)

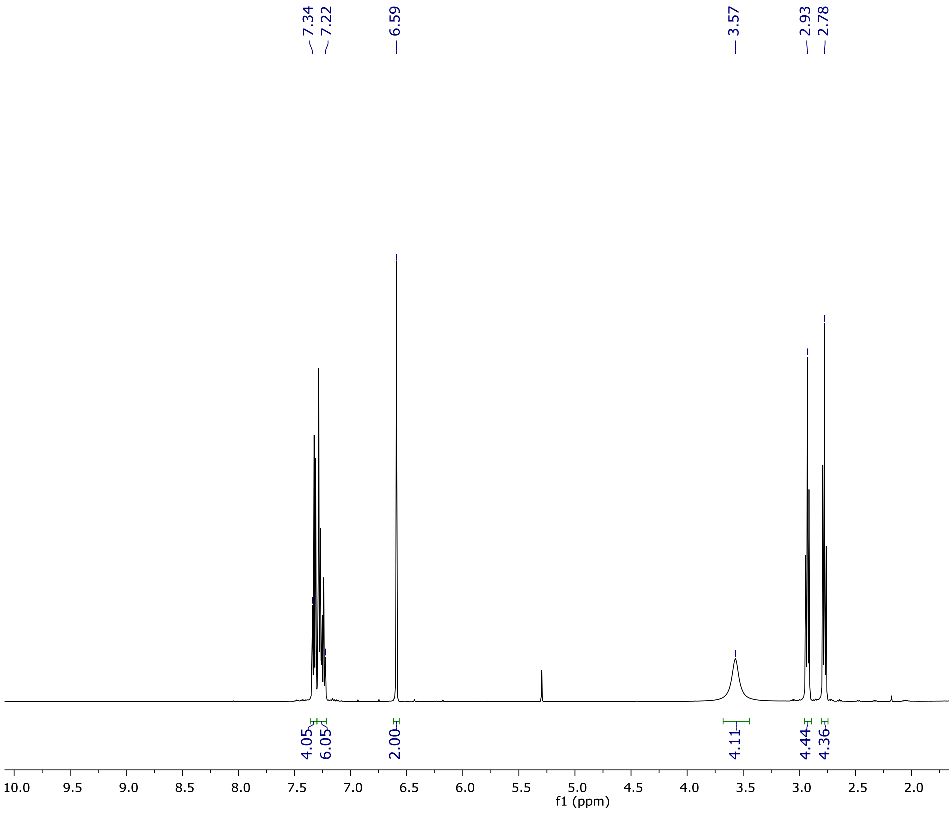

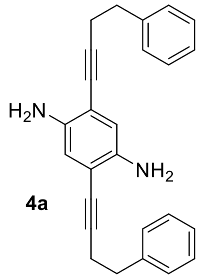

4a

^{1}H-NMR (300 MHz, $CDCl_3$)

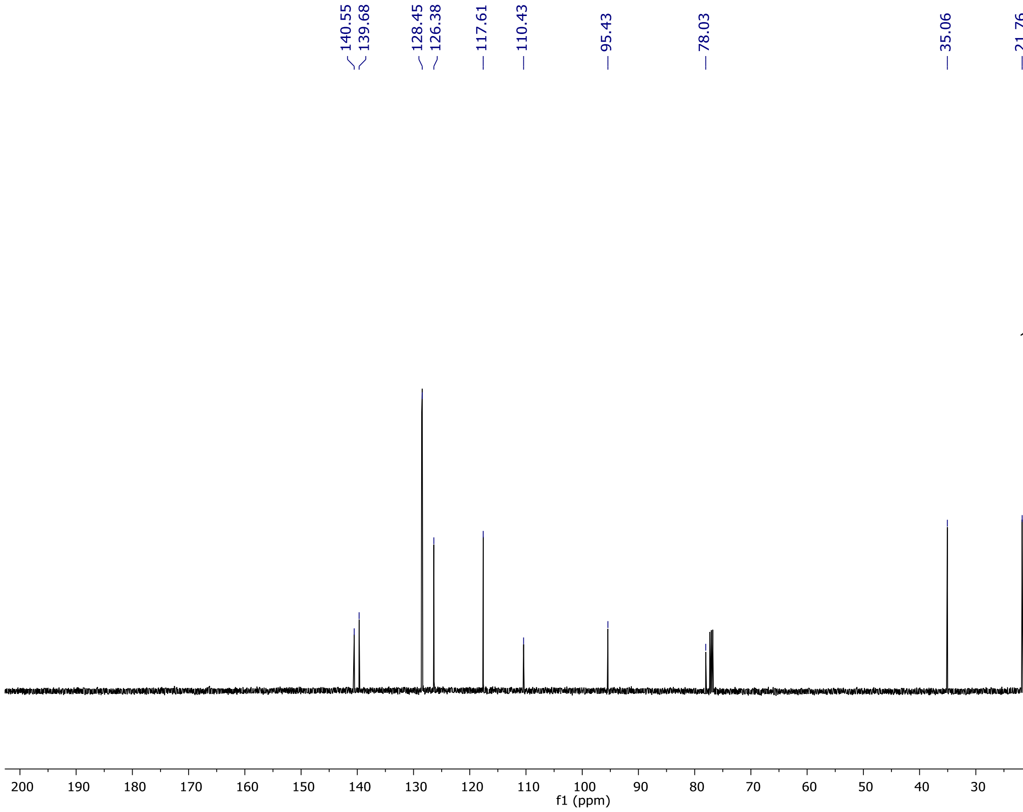

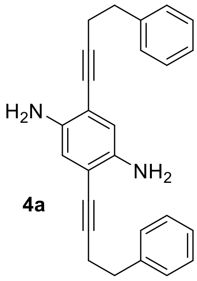

4a

^{13}C-NMR (125 MHz, $CDCl_3$)

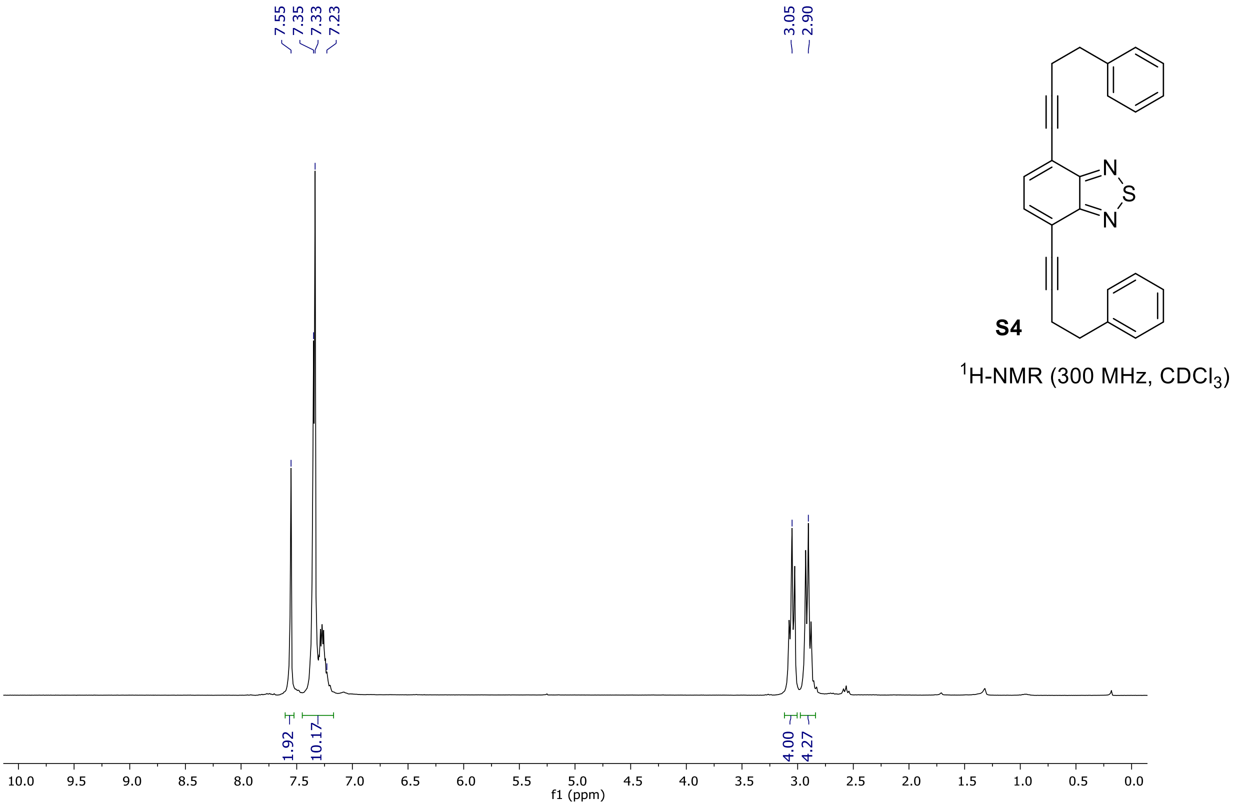
7.55
7.35
7.33
7.23
3.05
2.90
S4
^{1}H-NMR (300 MHz, $CDCl_3$)
1.92
10.17
4.00
4.27
10.0 9.5 9.0 8.5 8.0 7.5 7.0 6.5 6.0 5.5 5.0 4.5 4.0 3.5 3.0 2.5 2.0 1.5 1.0 0.5 0.0
f1 (ppm)

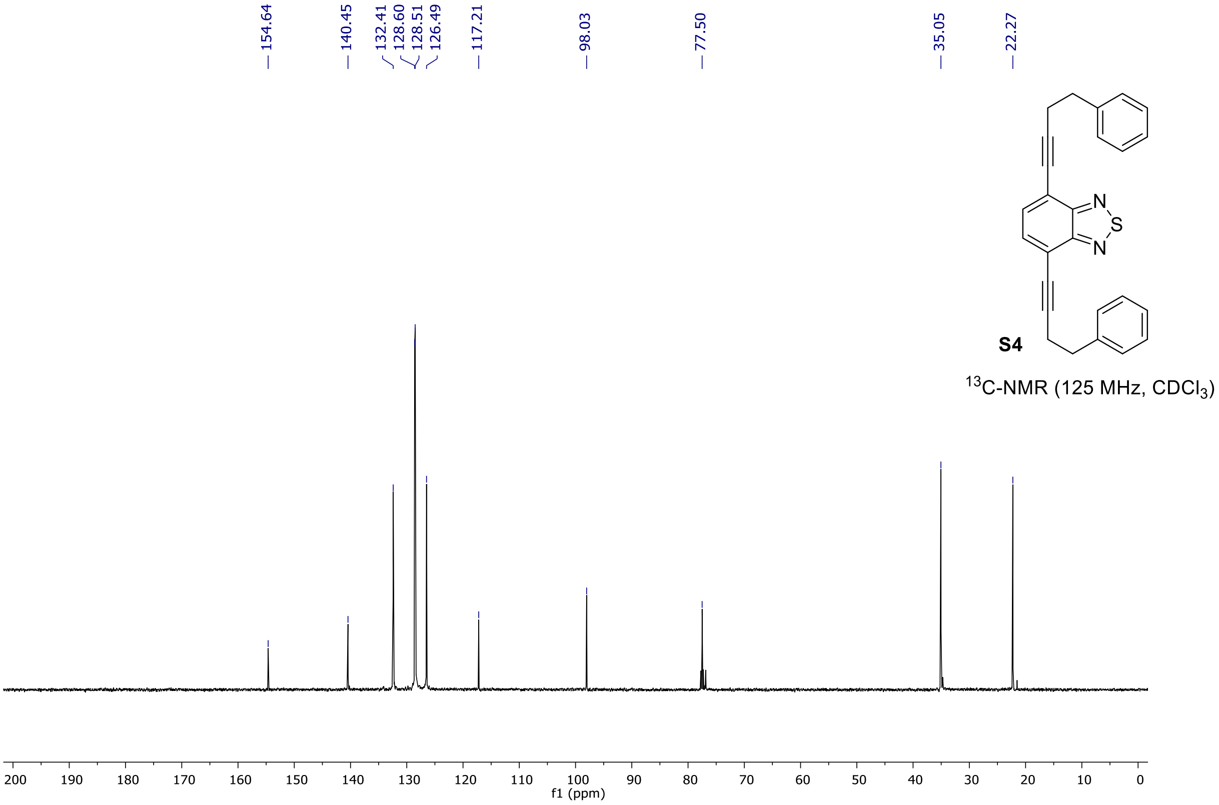
154.64
140.45
132.41
128.60
128.51
126.49
117.21
98.03
77.50
35.05
22.27
S4
^{13}C-NMR (125 MHz, $CDCl_3$)
200 190 180 170 160 150 140 130 120 110 100 90 80 70 60 50 40 30 20 10 0
f1 (ppm)

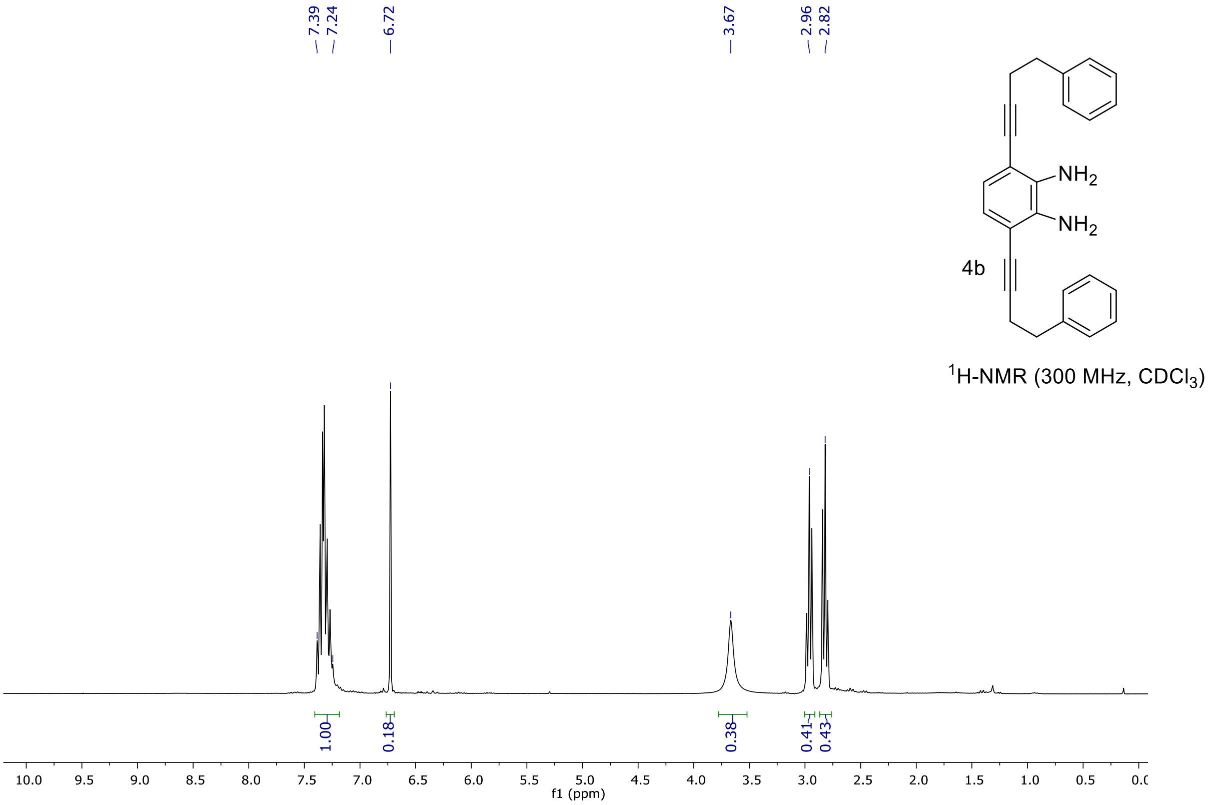
7.39
7.24
6.72
3.67
2.96
2.82
NH2
NH2
4b
1H-NMR (300 MHz, CDCl3)
1.00
0.18
0.38
0.41
0.43
f1 (ppm)

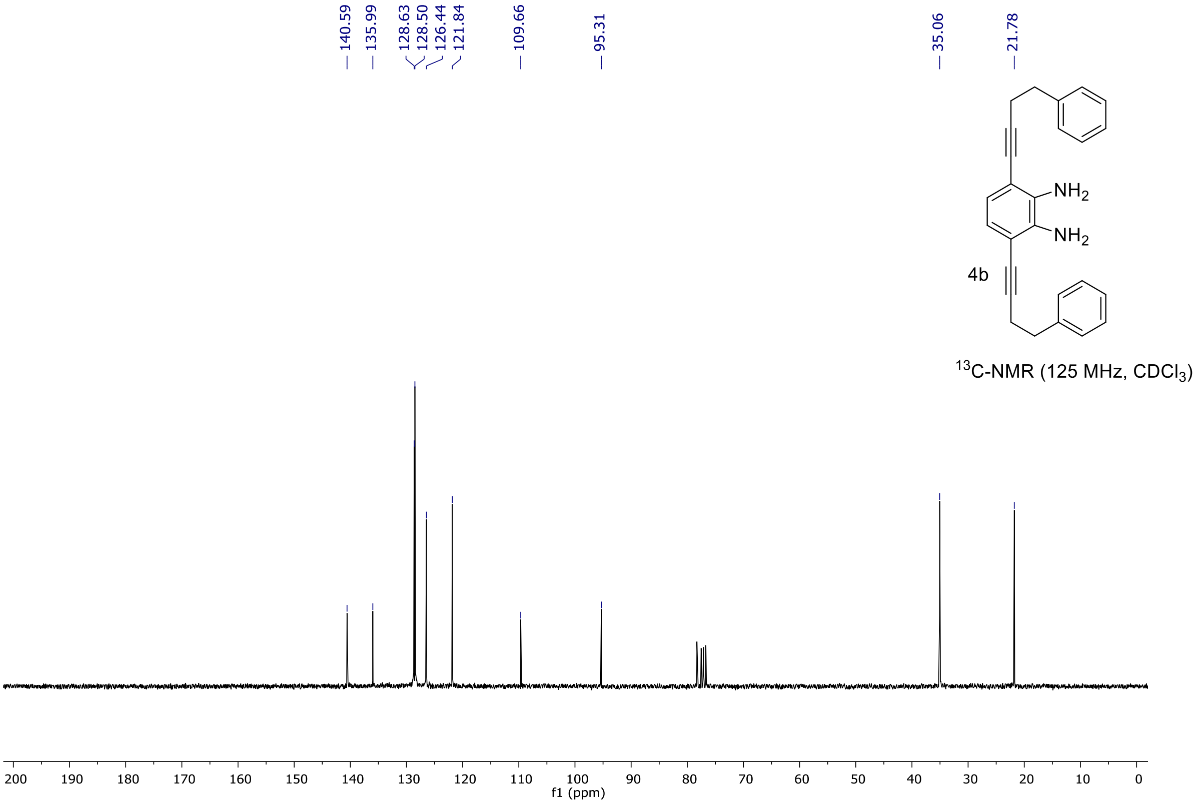
140.59
135.99
128.63
128.50
126.44
121.84
109.66
95.31
35.06
21.78
NH2
NH2
4b
13C-NMR (125 MHz, CDCl3)
f1 (ppm)

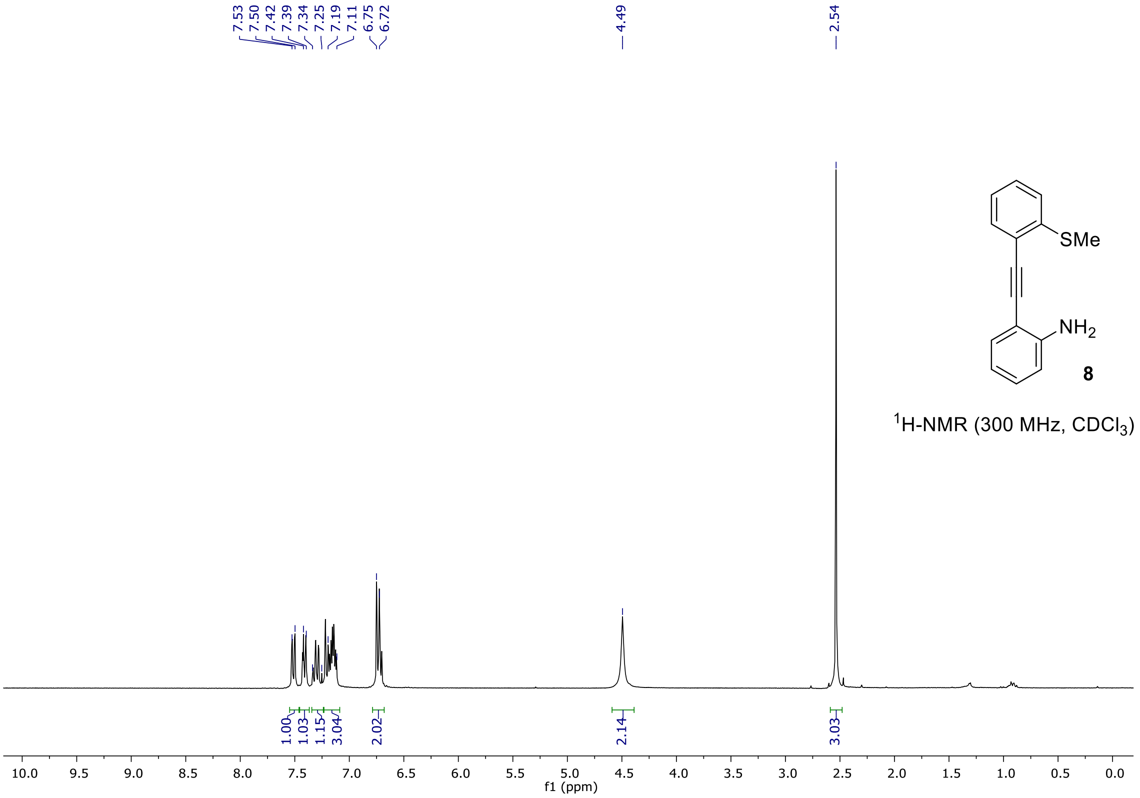
7.53
7.50
7.42
7.39
7.34
7.25
7.19
7.11
6.75
6.72
4.49
2.54
SMe
NH2
8
1H-NMR (300 MHz, CDCl3)
1.00
1.03
1.15
3.04
2.02
2.14
3.03
f1 (ppm)

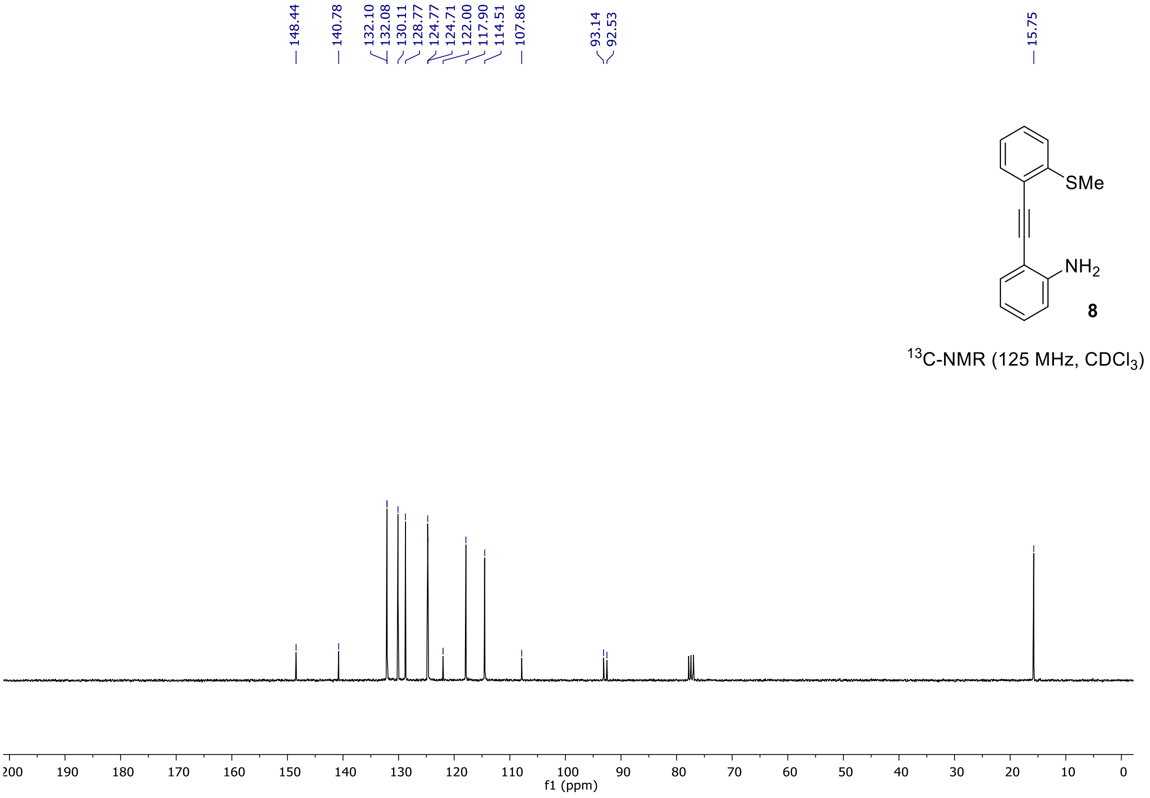
148.44
140.78
132.10
132.08
130.11
128.77
124.77
124.71
122.00
117.90
114.51
107.86
93.14
92.53
15.75
SMe
NH2
8
13C-NMR (125 MHz, CDCl3)
f1 (ppm)

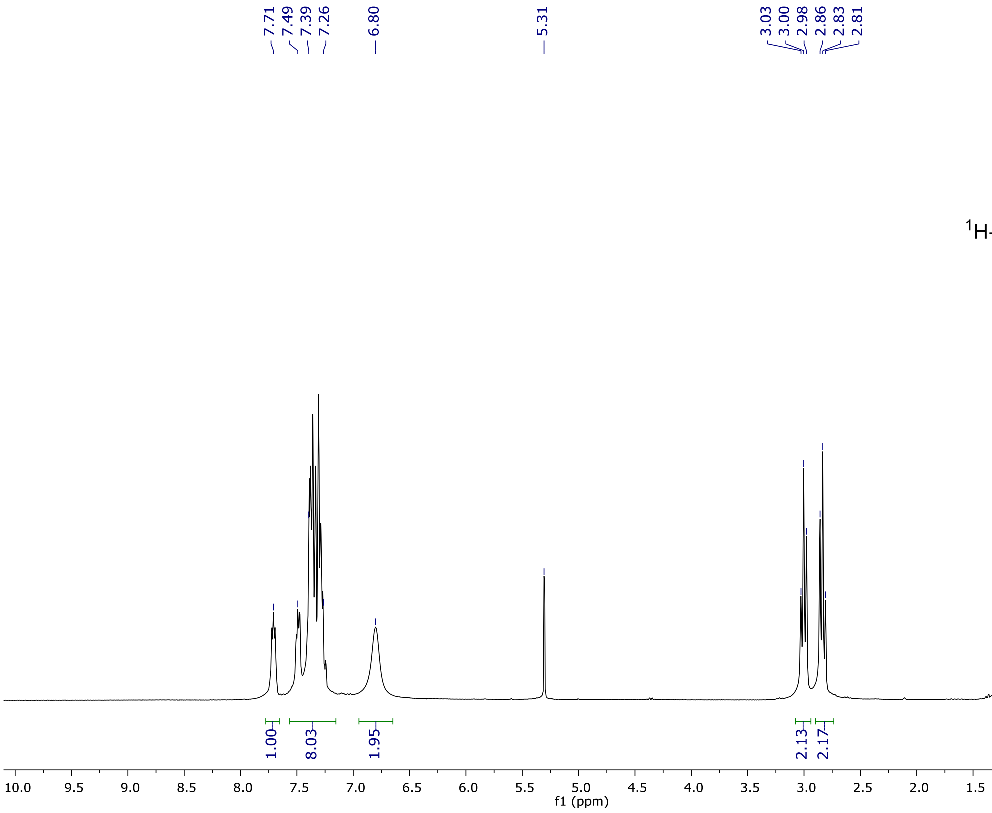

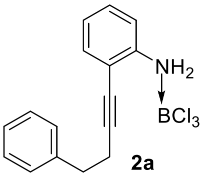

2a

^{1}H-NMR (500 MHz, $CDCl_3$)

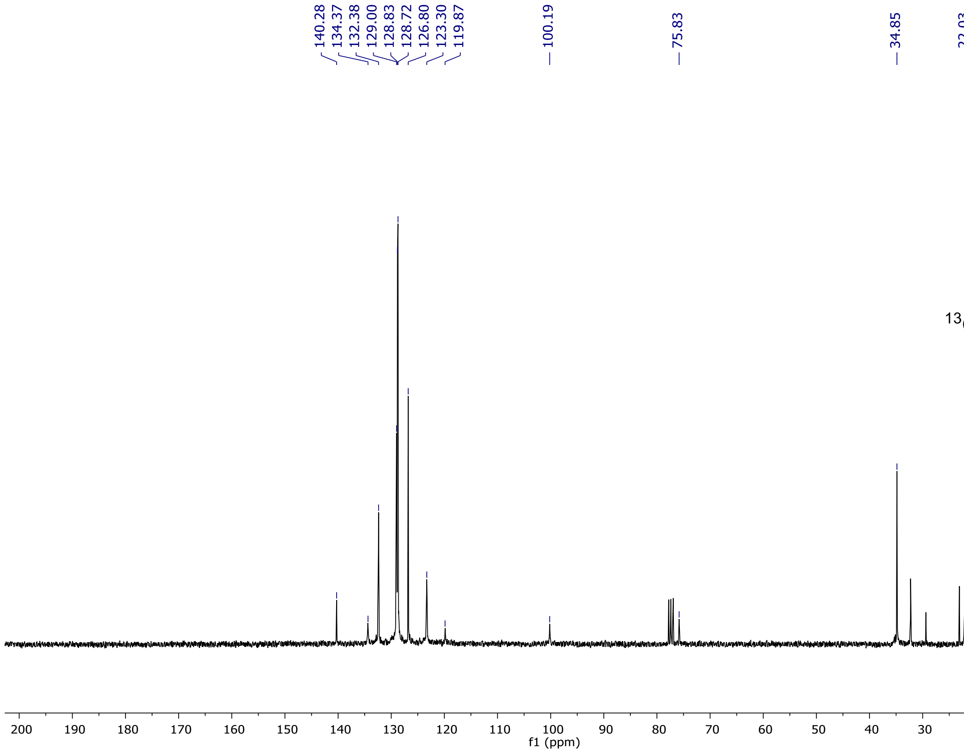

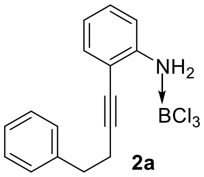

2a

^{13}C-NMR (125 MHz, $CDCl_3$)

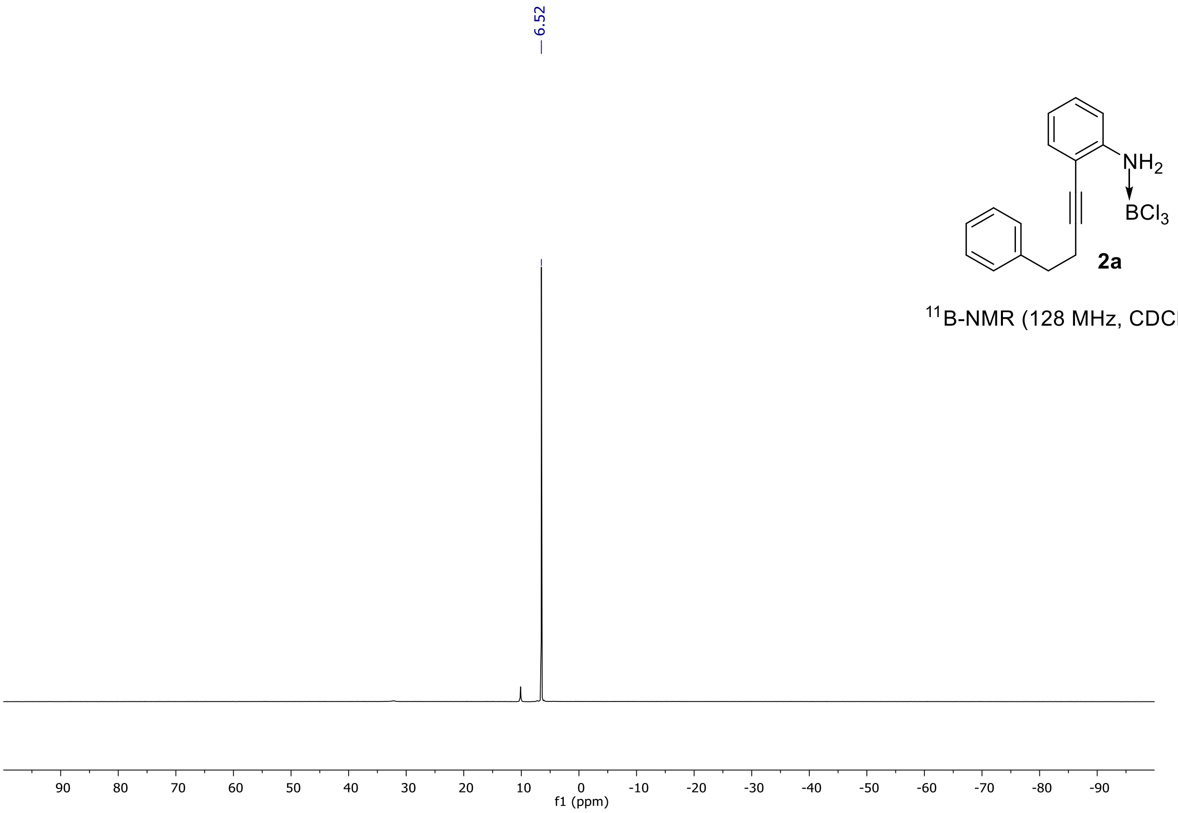

2a

^{11}B-NMR (128 MHz, $CDCl_3$)

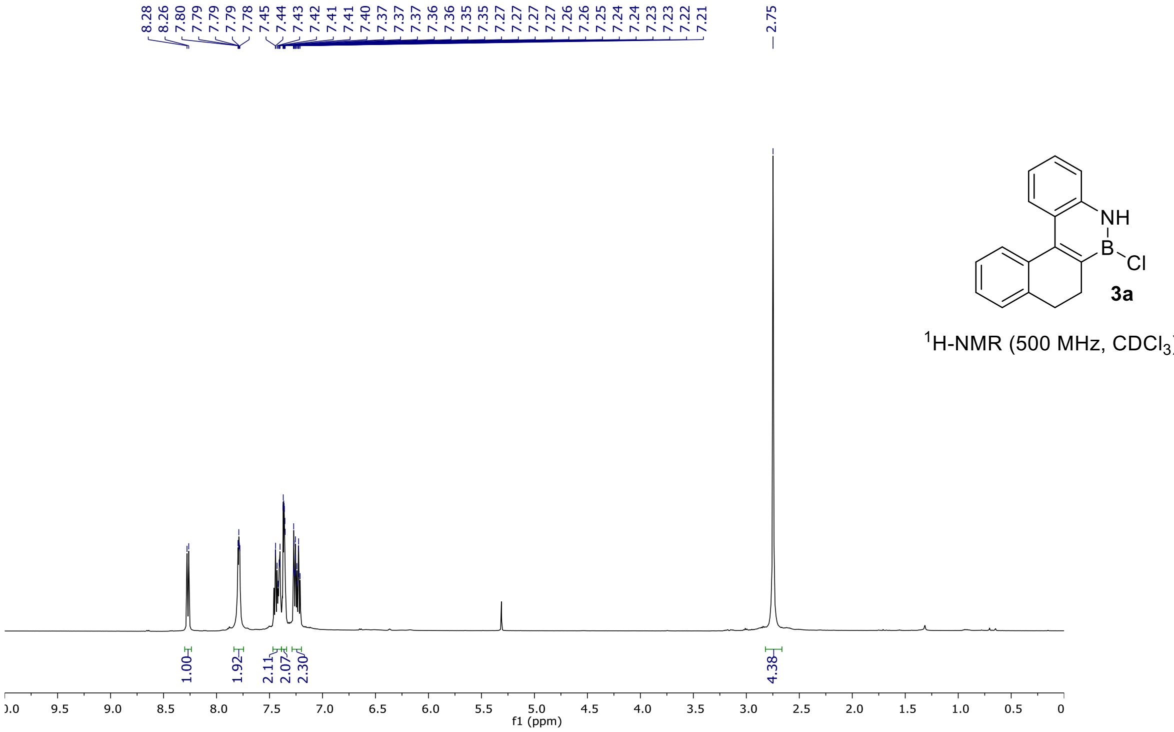

3a

^{1}H-NMR (500 MHz, $CDCl_3$)

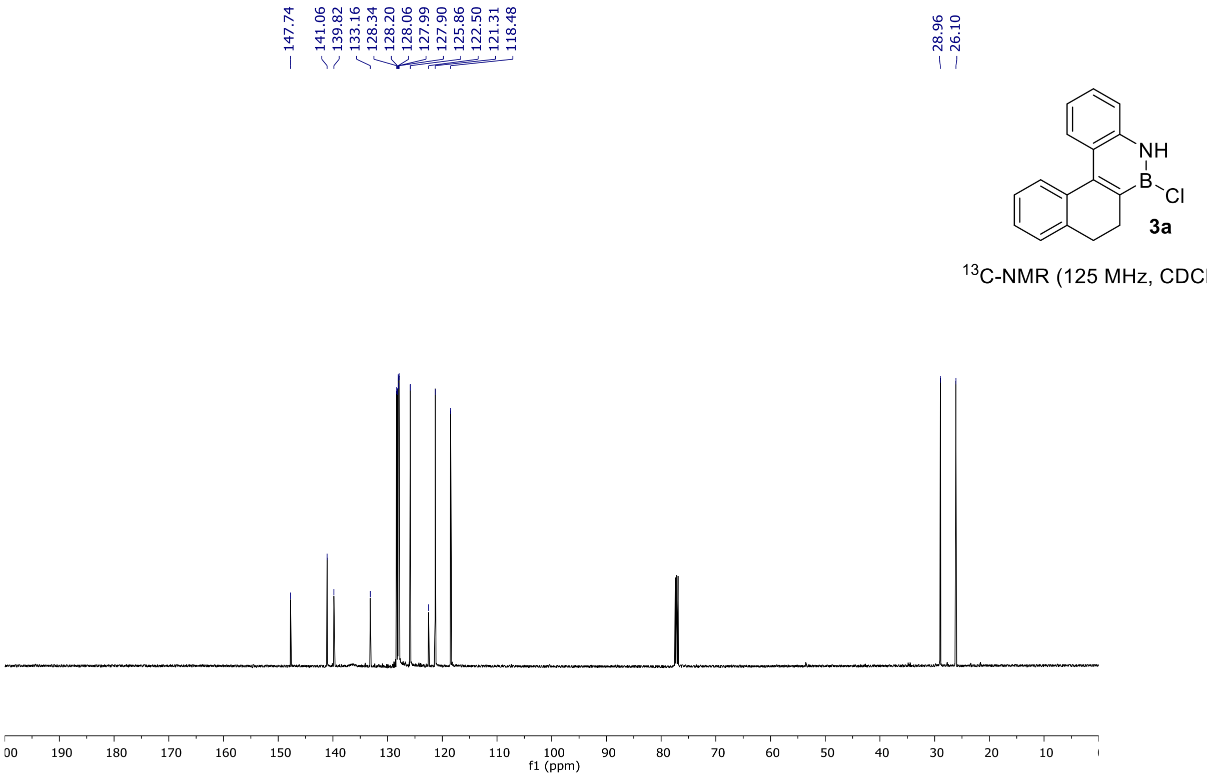

3a

^{13}C-NMR (125 MHz, $CDCl_3$)

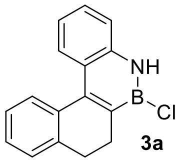

3a

^{11}B-NMR (128 MHz, $CDCl_3$)

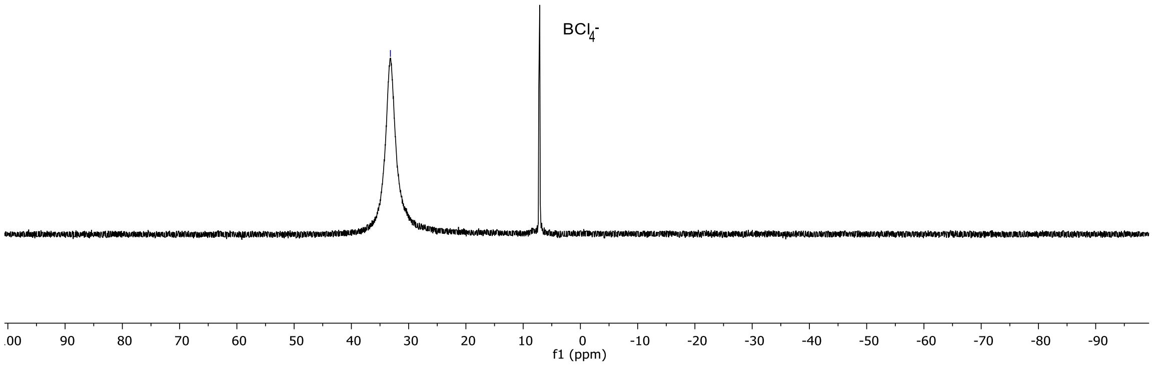

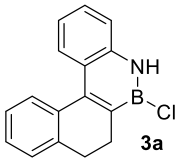

3a

COSY-NMR ($CDCl_3$)

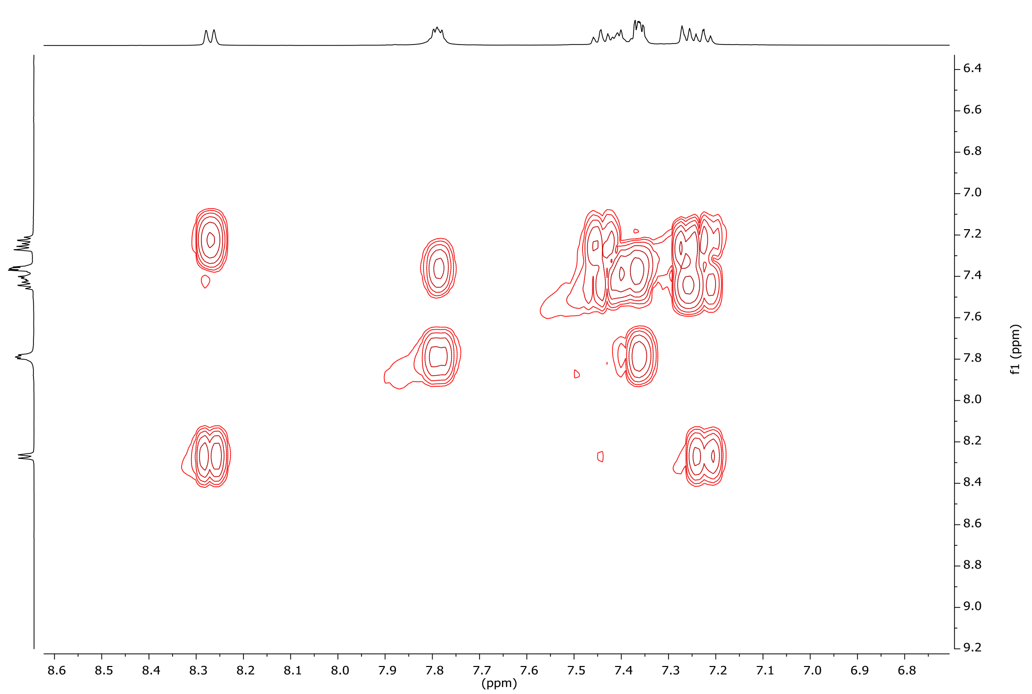

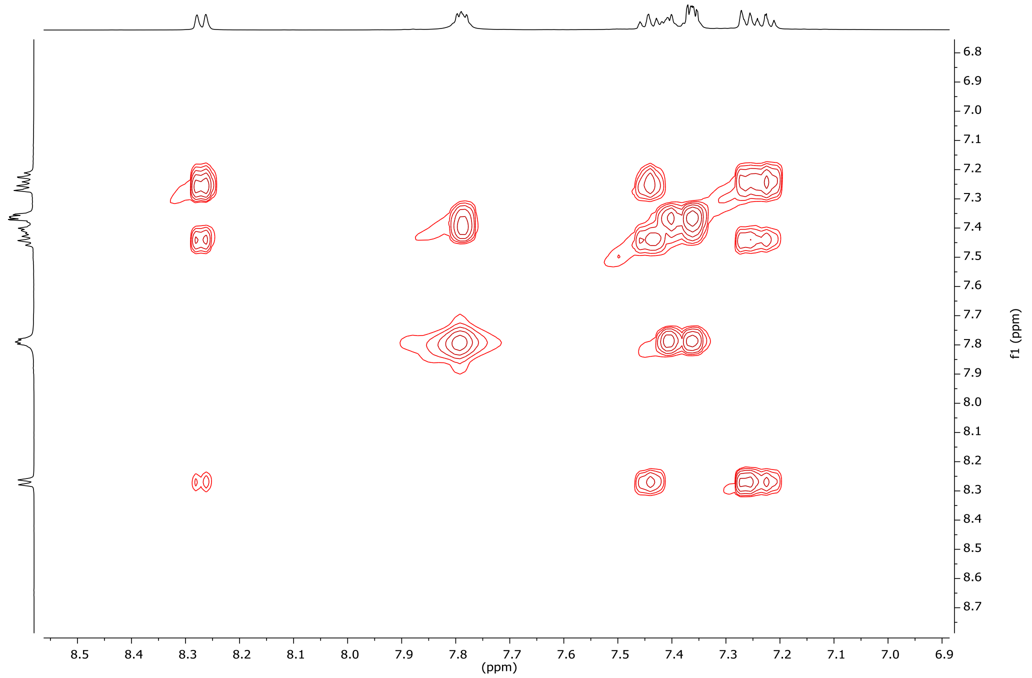

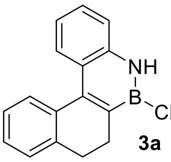

3a

TOCSY-NMR (CDCl$_3$)

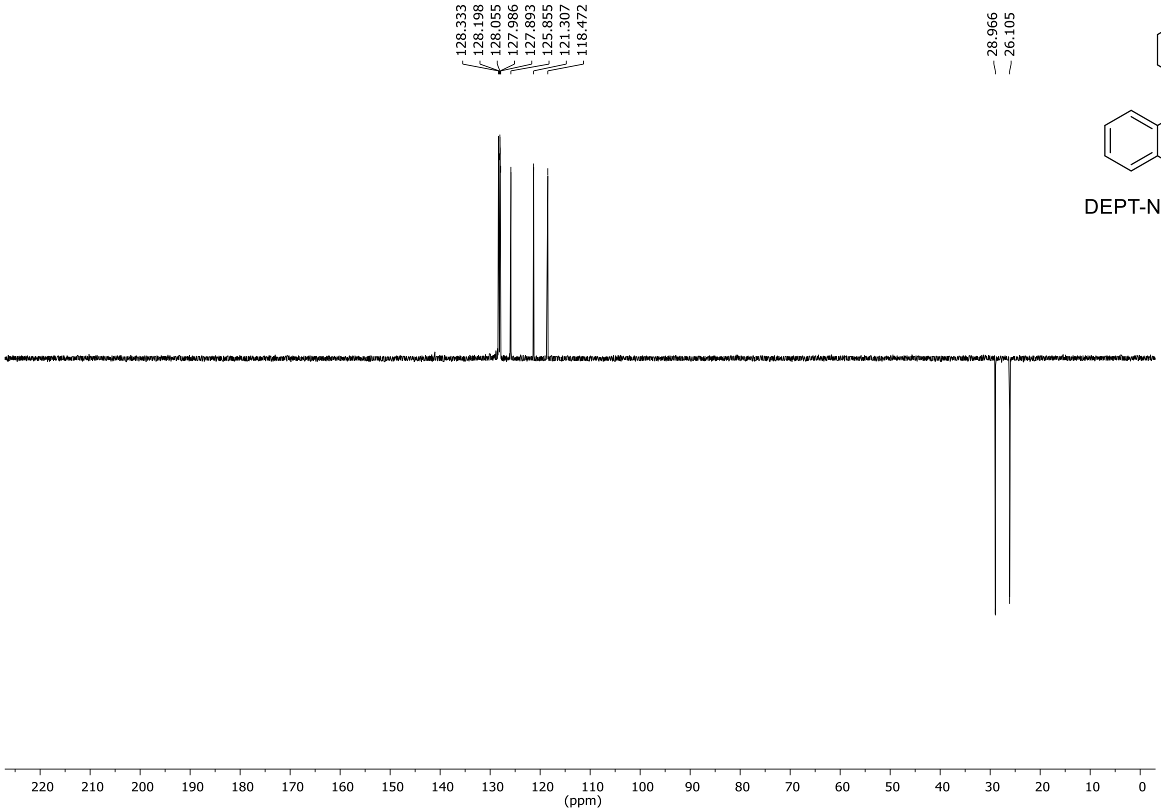

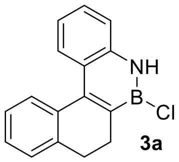

3a

DEPT-NMR (CDCl$_3$)

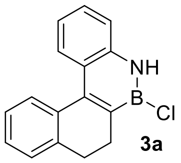

3a

HSQC-NMR $(CDCl_3)$

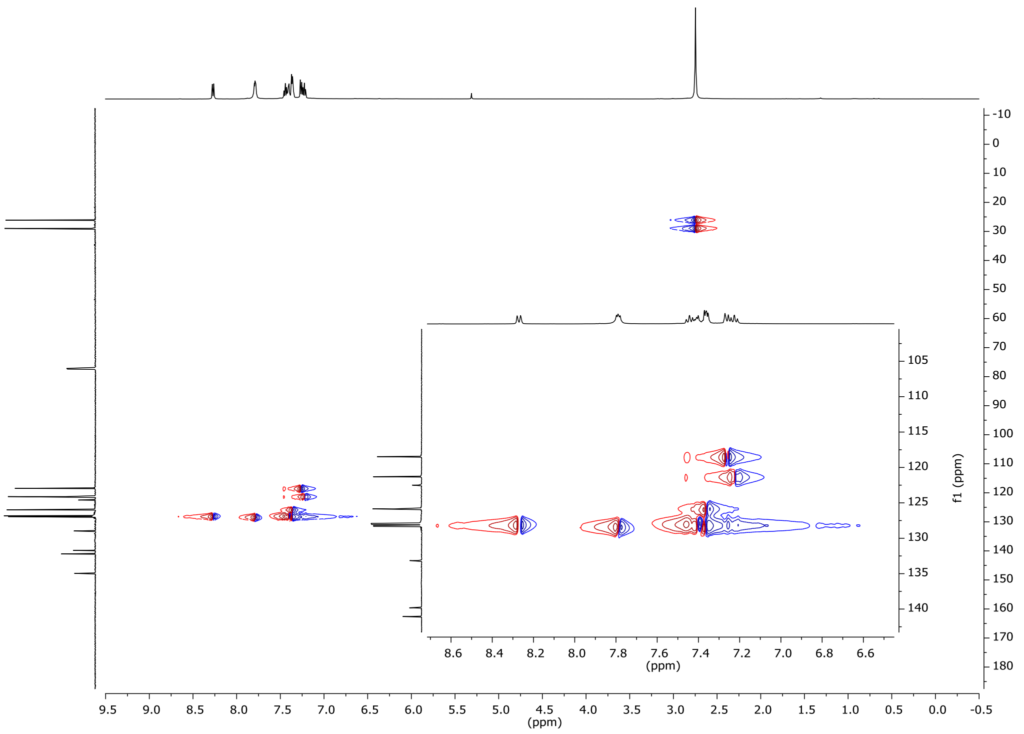

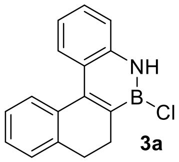

3a

HMBC-NMR $(CDCl_3)$

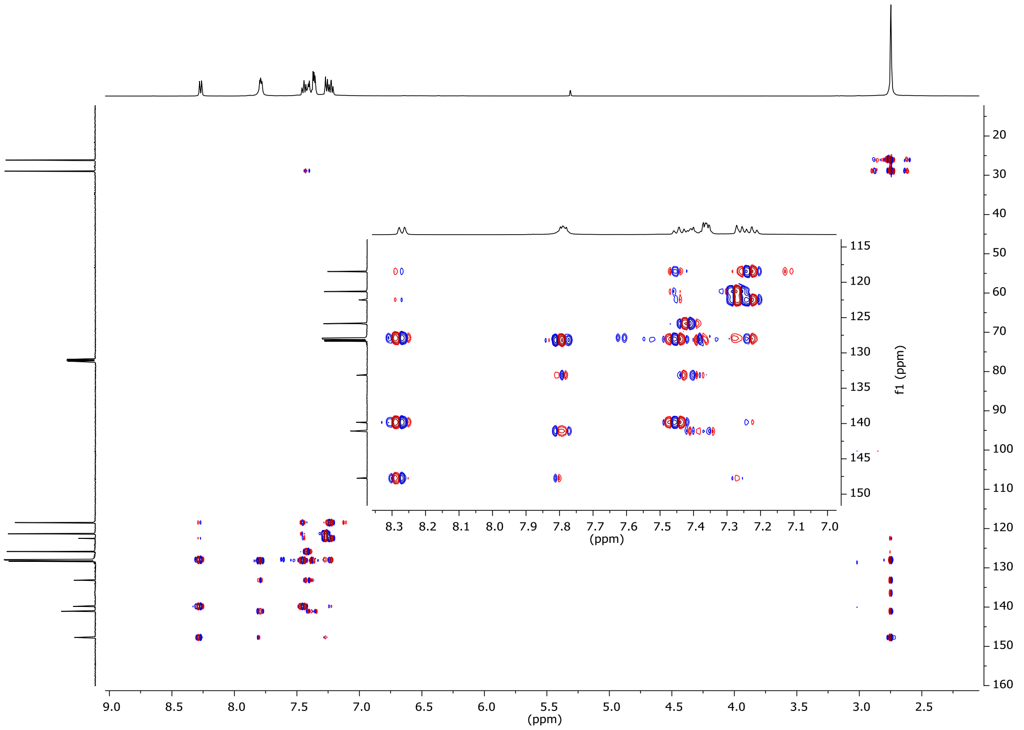

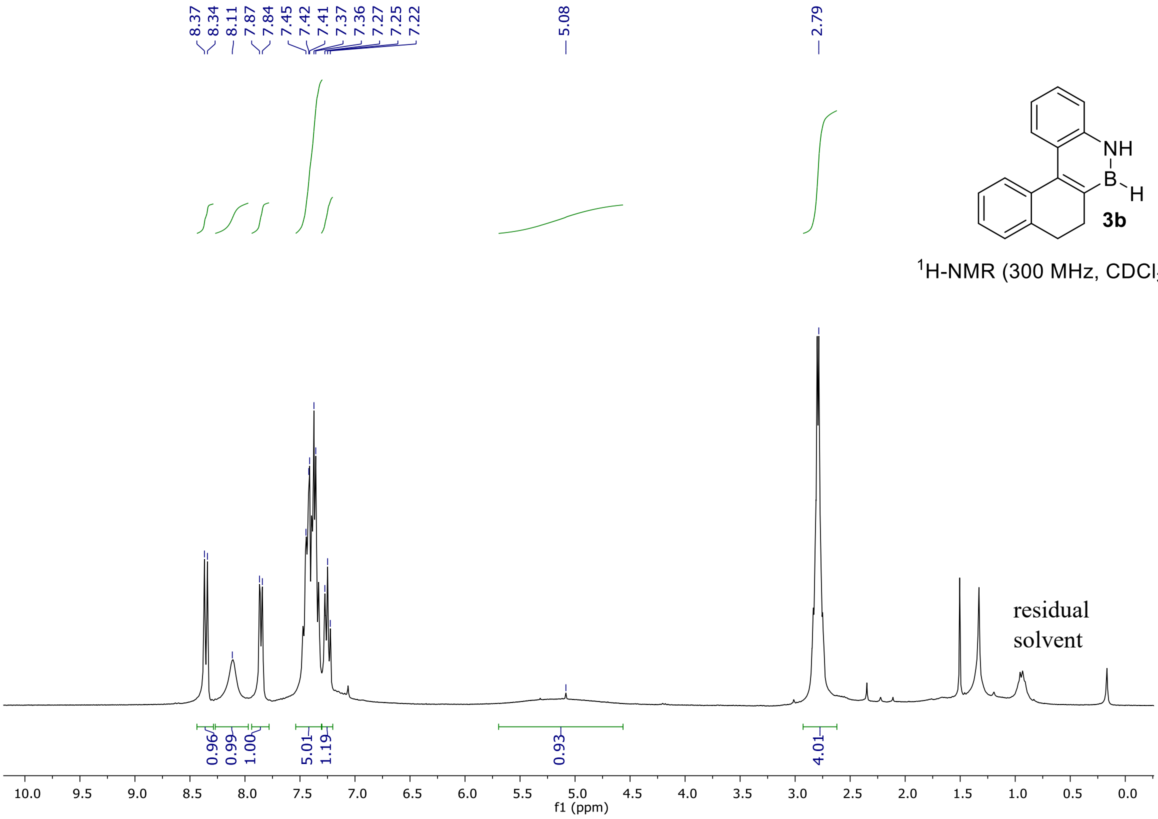

^{1}H-NMR (300 MHz, $CDCl_3$)

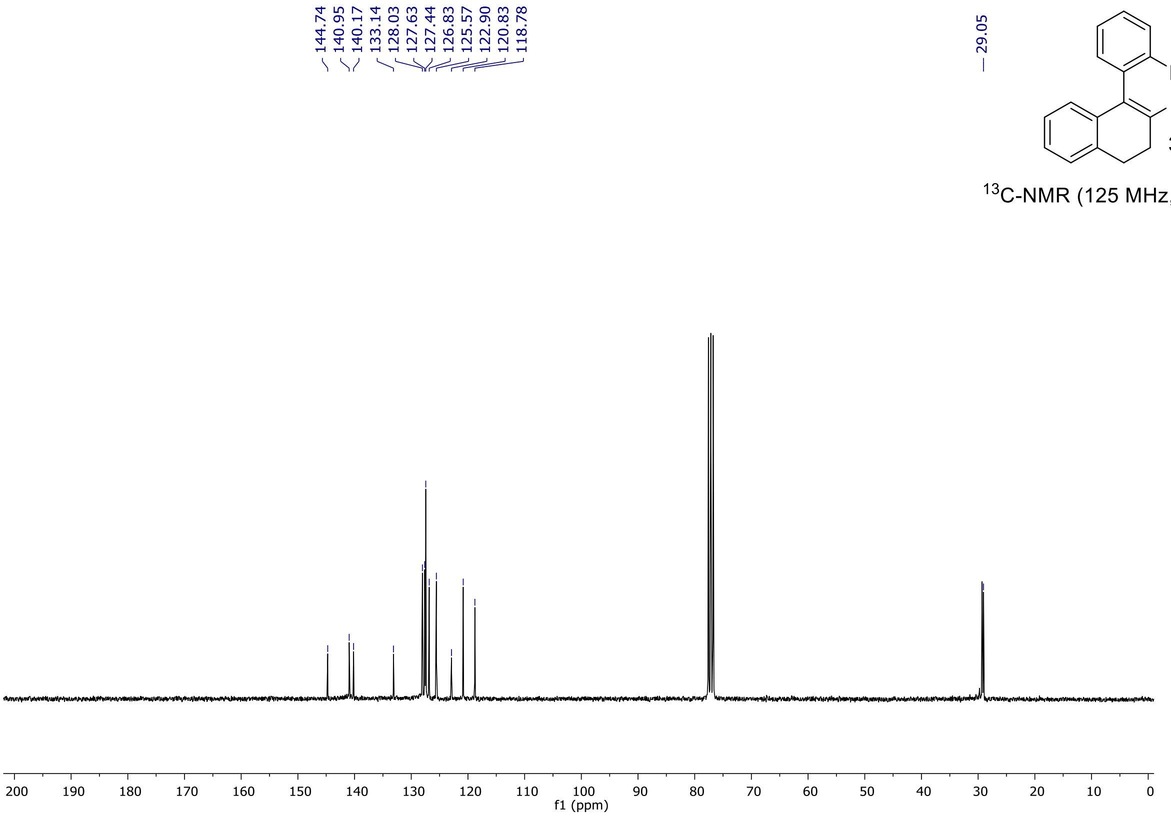

^{13}C-NMR (125 MHz, $CDCl_3$)

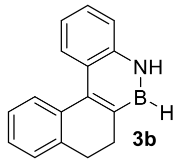

3b

^{11}B-NMR (128 MHz, $CDCl_3$)

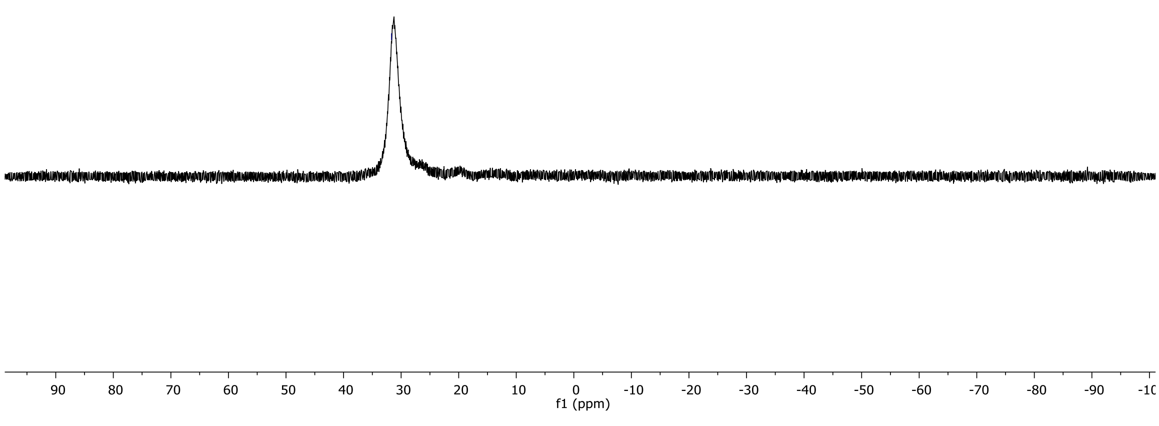

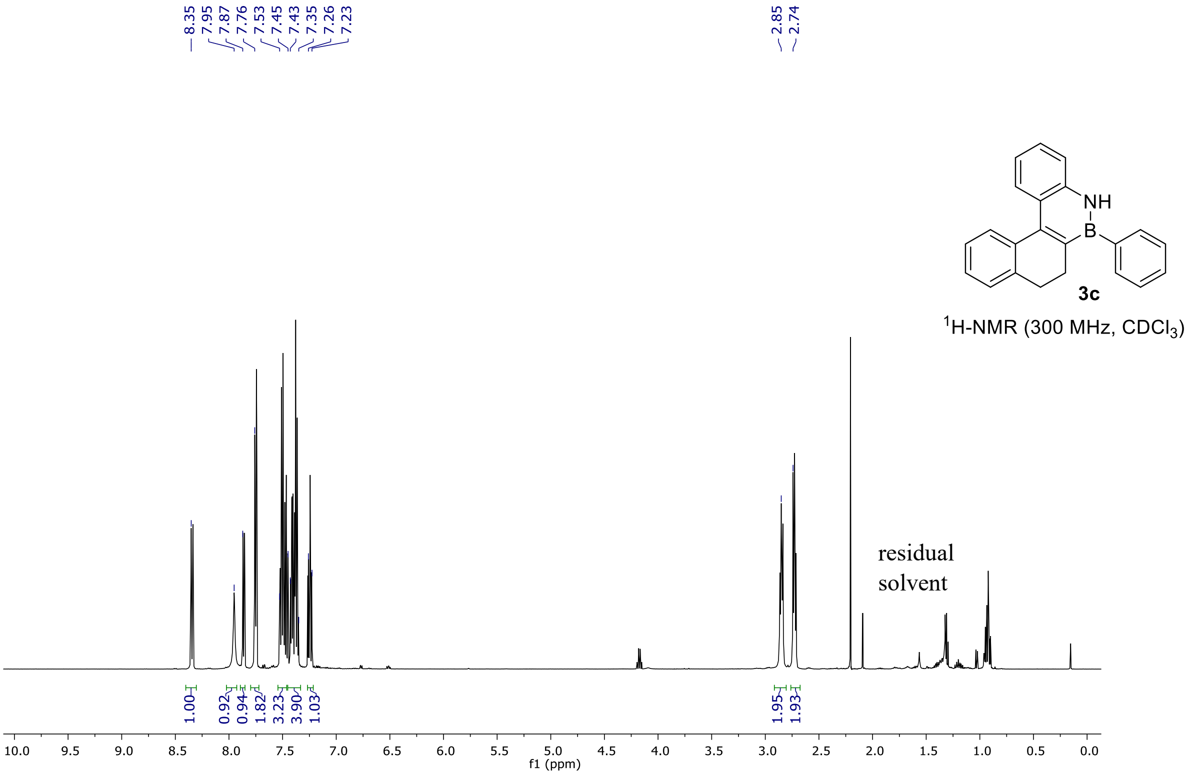
8.35
7.95
7.87
7.76
7.53
7.45
7.43
7.35
7.26
7.23
2.85
2.74
NH
B
3c
¹H-NMR (300 MHz, CDCl₃)
residual
solvent
1.00
0.92
0.94
1.82
3.23
3.90
1.03
1.95
1.93
f1 (ppm)

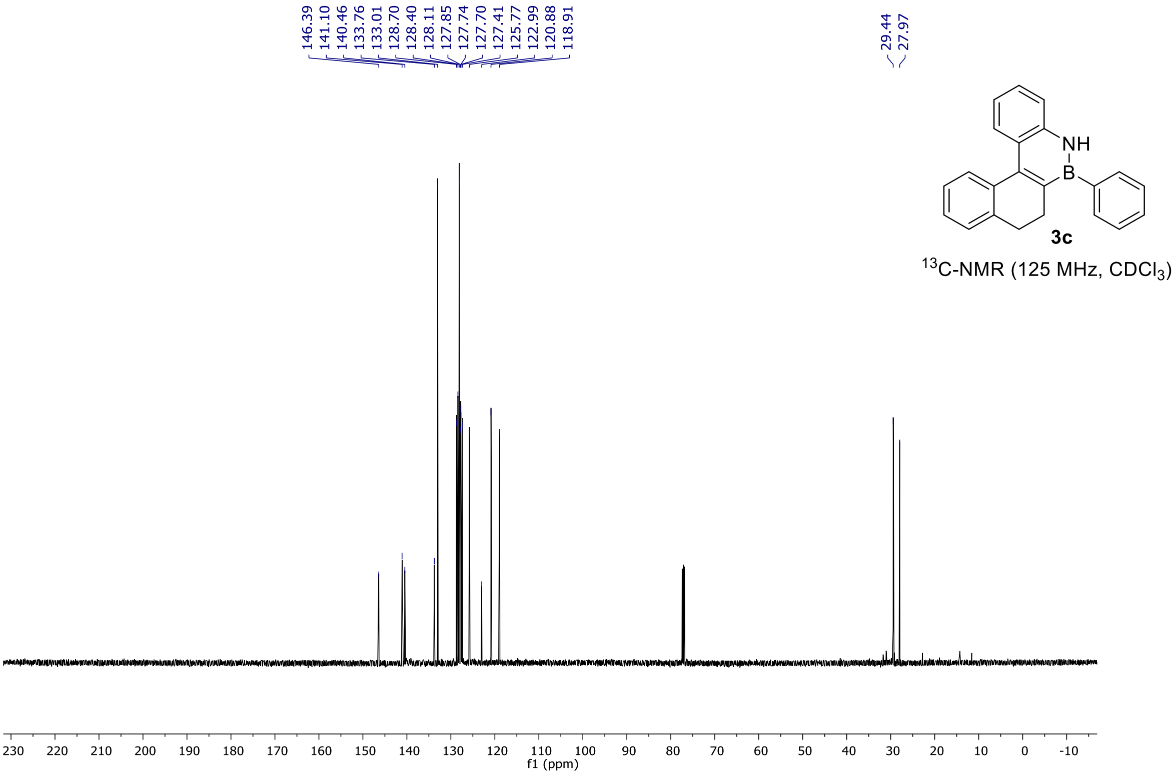
146.39
141.10
140.46
133.76
133.01
128.70
128.40
128.11
127.85
127.74
127.70
127.41
125.77
122.99
120.88
118.91
29.44
27.97
NH
B
3c
¹³C-NMR (125 MHz, CDCl₃)
f1 (ppm)

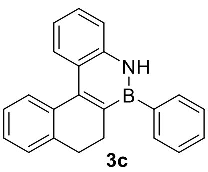

3c

^{11}B-NMR (128 MHz, $CDCl_3$)

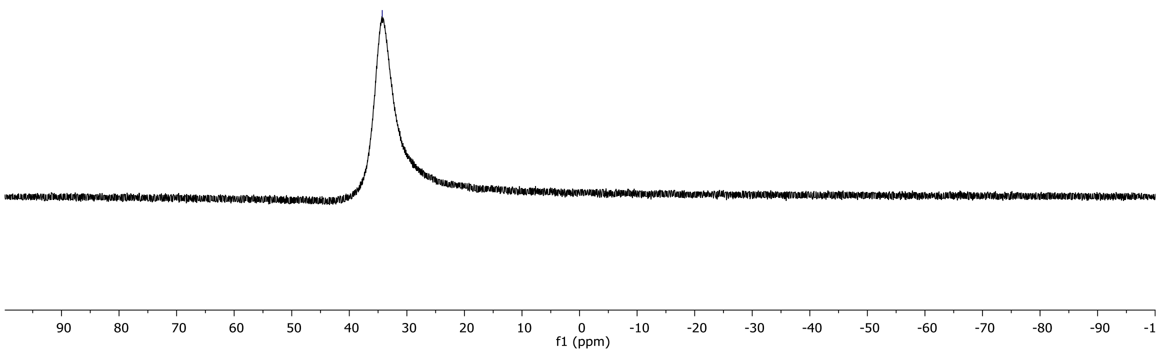

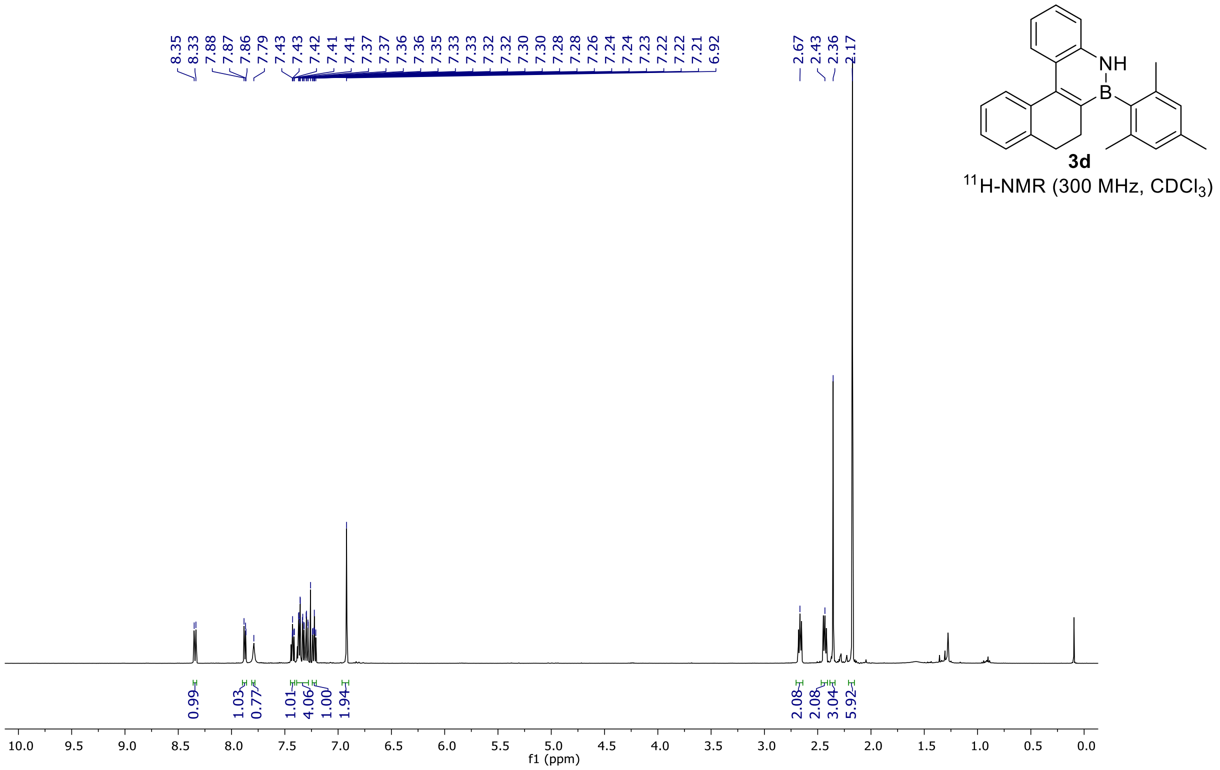

[11]H-NMR (300 MHz, $CDCl_3$)

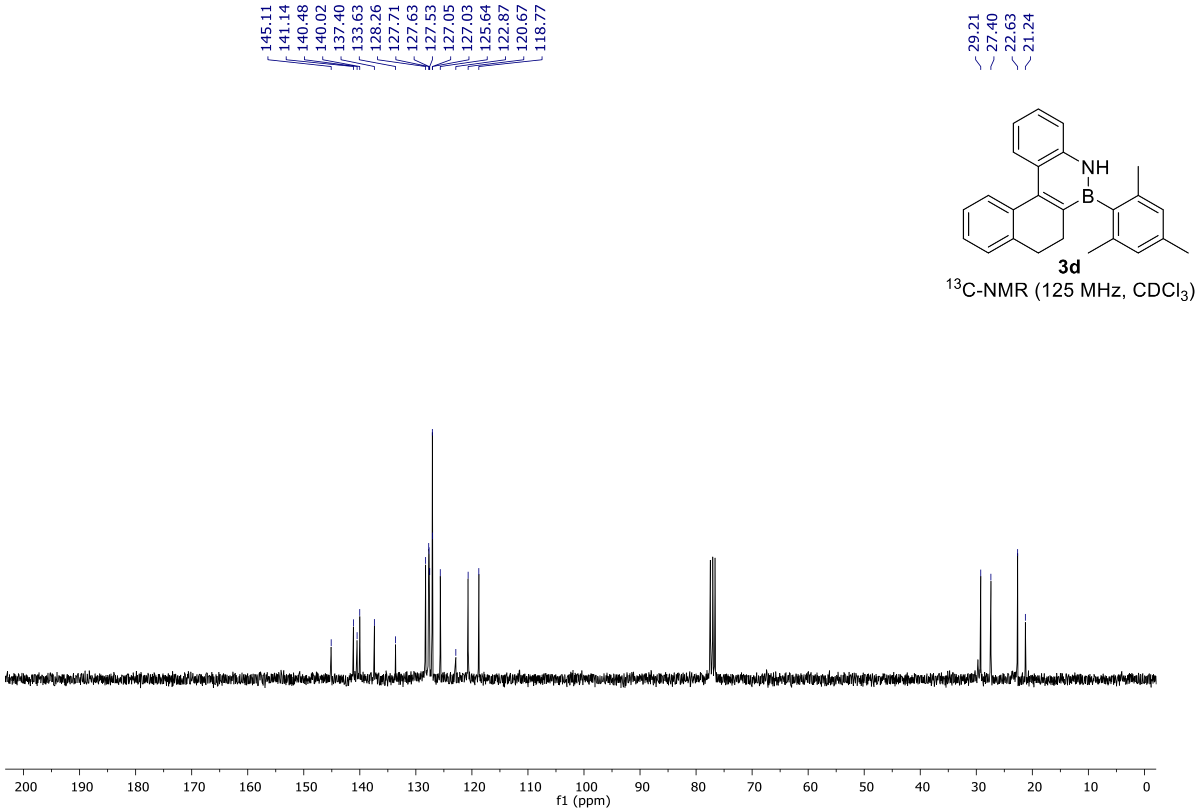

^{13}C-NMR (125 MHz, $CDCl_3$)

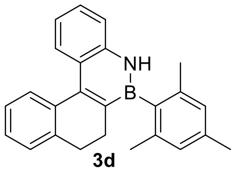

3d

^{11}B-NMR (128 MHz, $CDCl_3$)

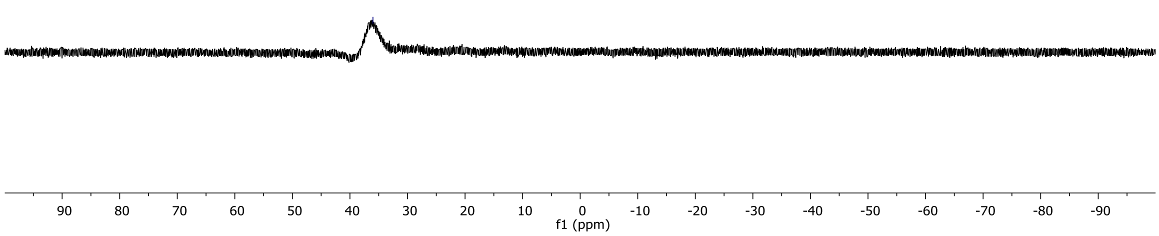

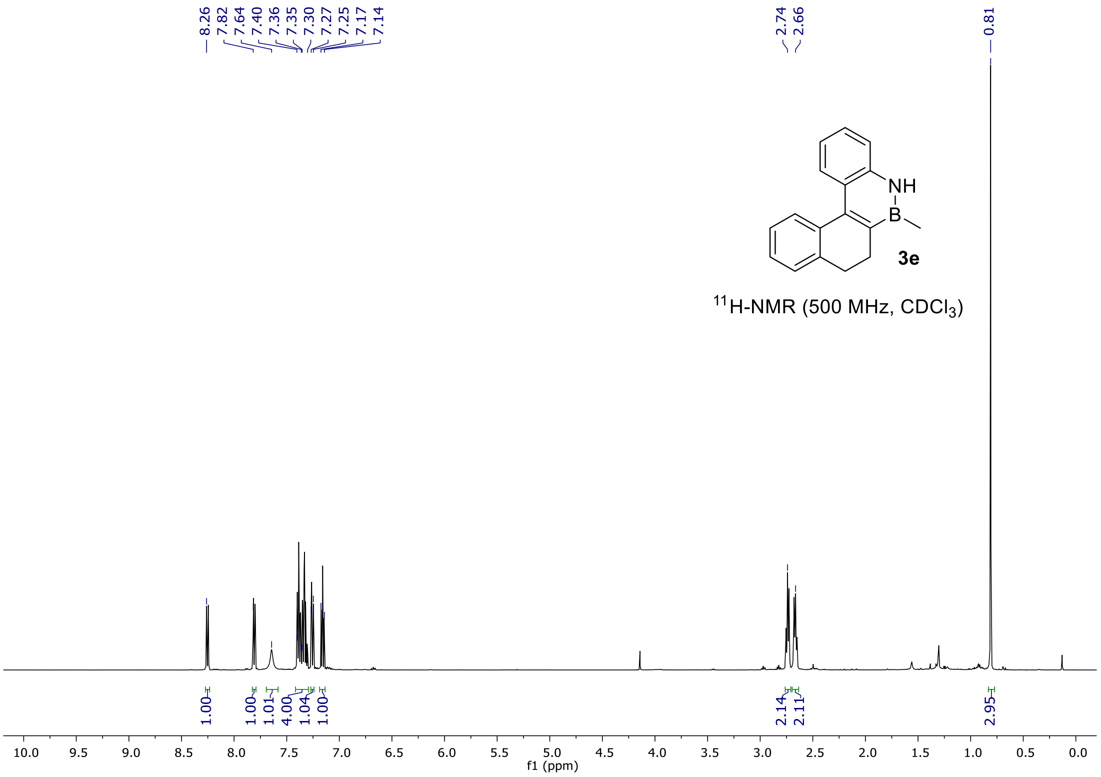
8.26
7.82
7.64
7.40
7.36
7.35
7.30
7.27
7.25
7.17
7.14
2.74
2.66
0.81
NH
B
3e
11H-NMR (500 MHz, CDCl3)
1.00
1.00
1.01
4.00
1.04
1.00
2.14
2.11
2.95
f1 (ppm)

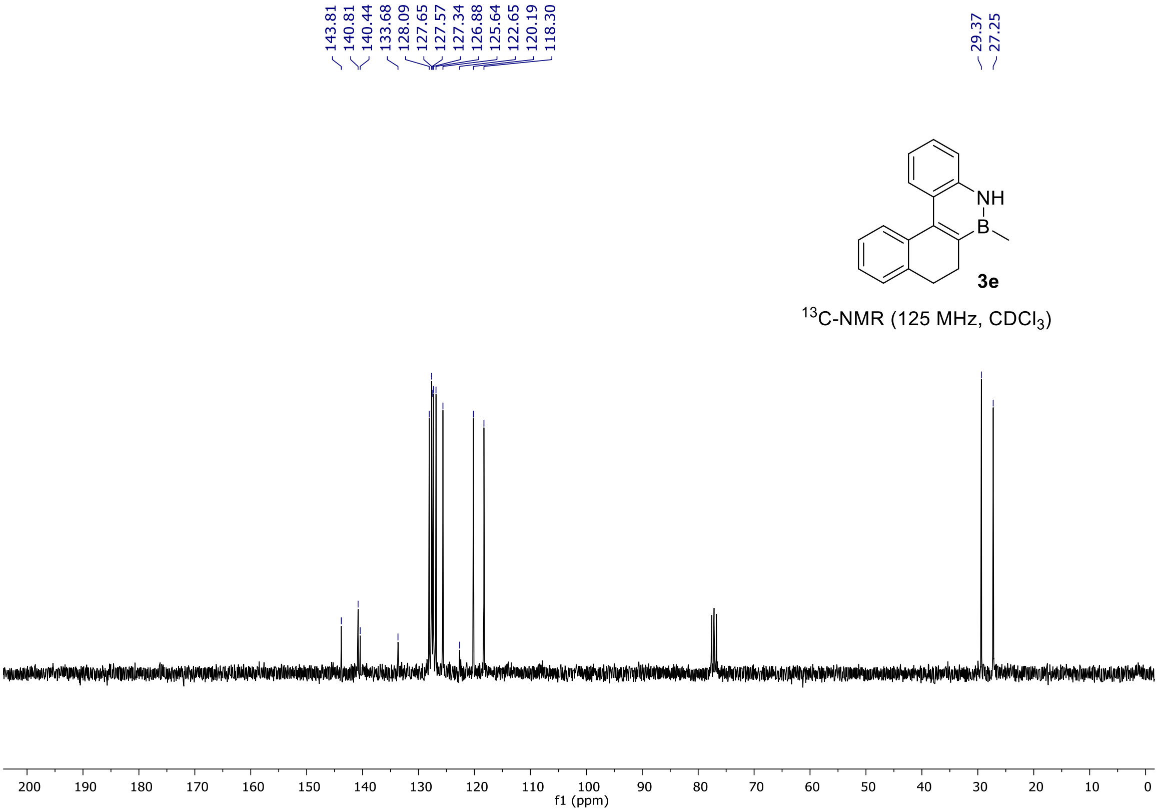
143.81
140.81
140.44
133.68
128.09
127.65
127.57
127.34
126.88
125.64
122.65
120.19
118.30
29.37
27.25
NH
B
3e
13C-NMR (125 MHz, CDCl3)
f1 (ppm)

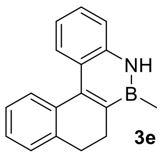

3e

^{11}B-NMR (128 MHz, $CDCl_3$)

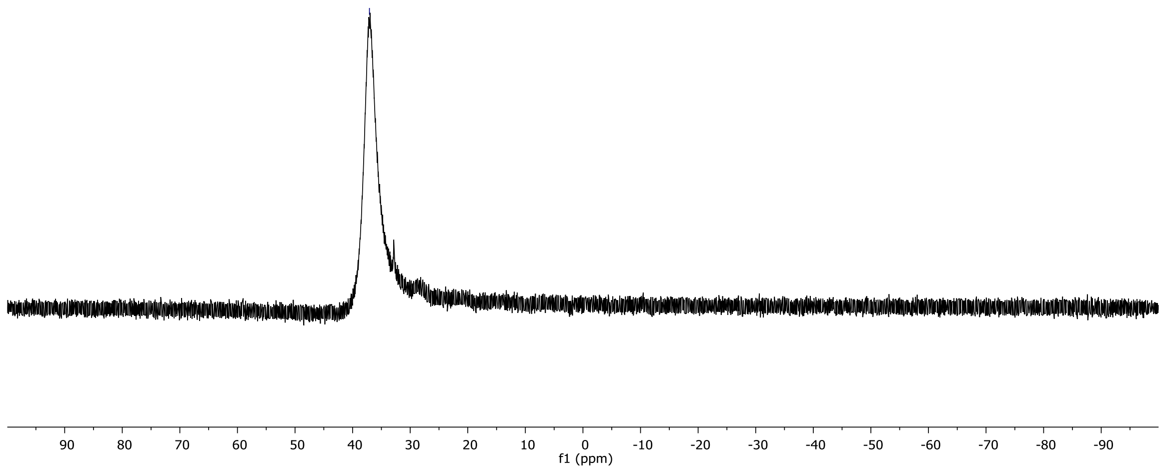

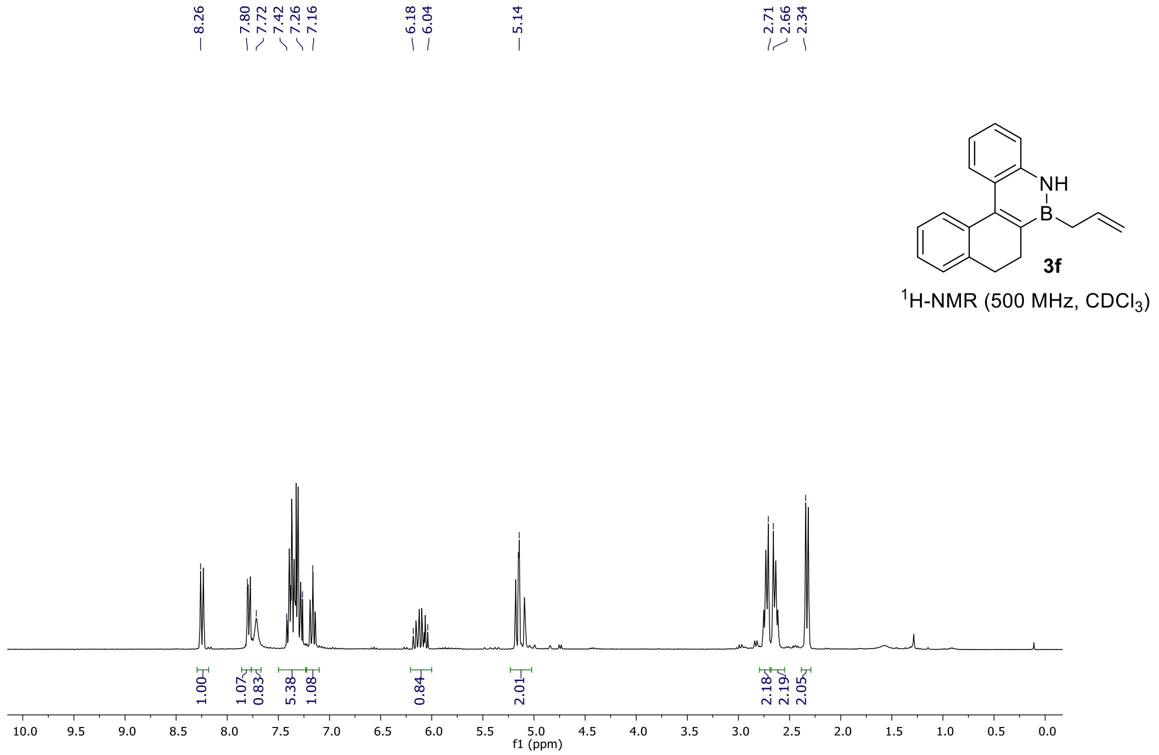

^{1}H-NMR (500 MHz, $CDCl_3$)

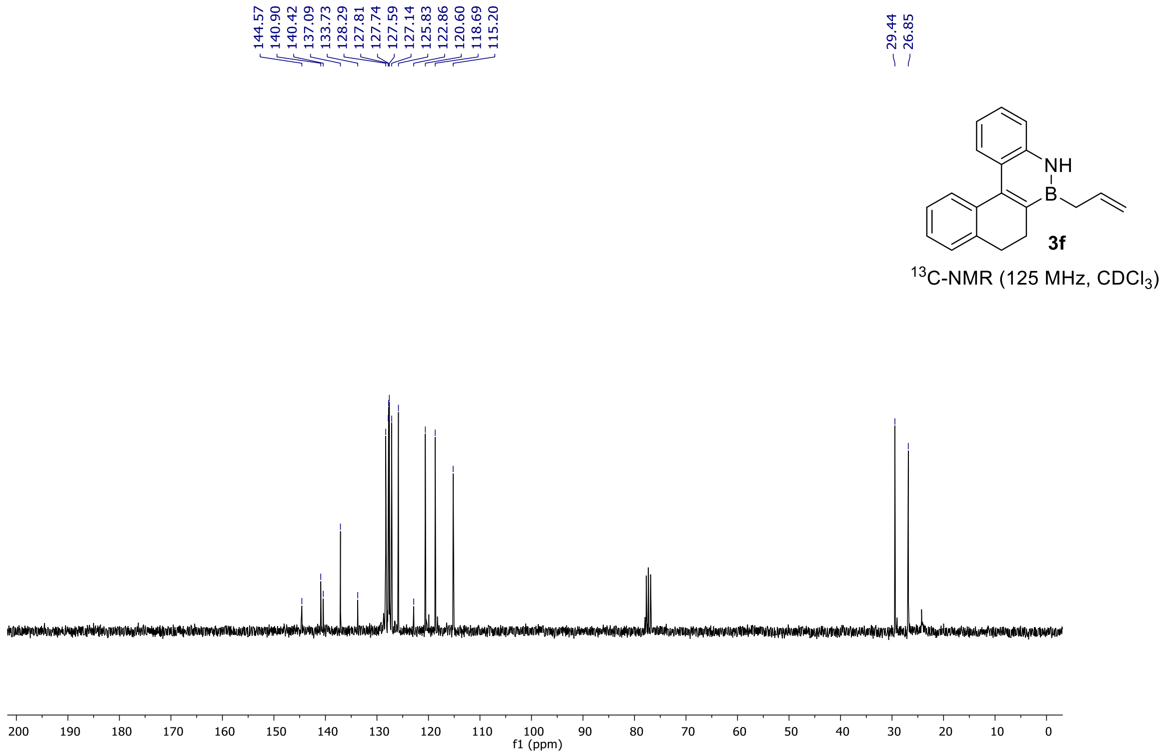

^{13}C-NMR (125 MHz, $CDCl_3$)

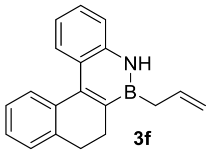

3f

^{11}B-NMR (128 MHz, $CDCl_3$)

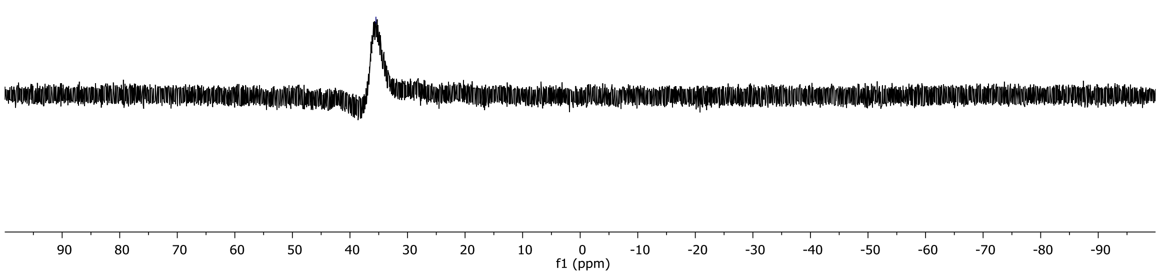

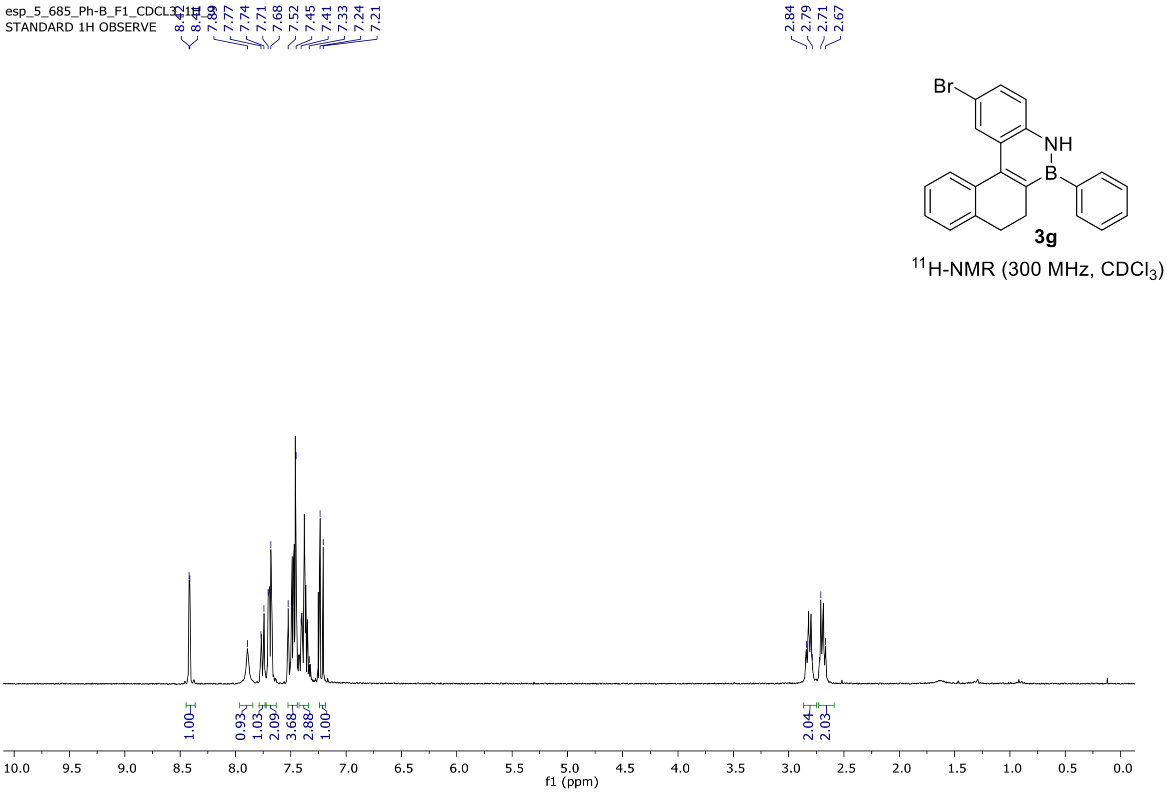
esp_5_685_Ph-B_F1_CDCL3_1H
STANDARD 1H OBSERVE
8.42 8.41 7.89 7.77 7.74 7.71 7.68 7.52 7.45 7.41 7.33 7.24 7.21
2.84 2.79 2.71 2.67
Br
NH
B
3g
11H-NMR (300 MHz, CDCl3)
1.00 0.93 1.03 2.09 3.68 2.88 1.00
2.04 2.03
f1 (ppm)

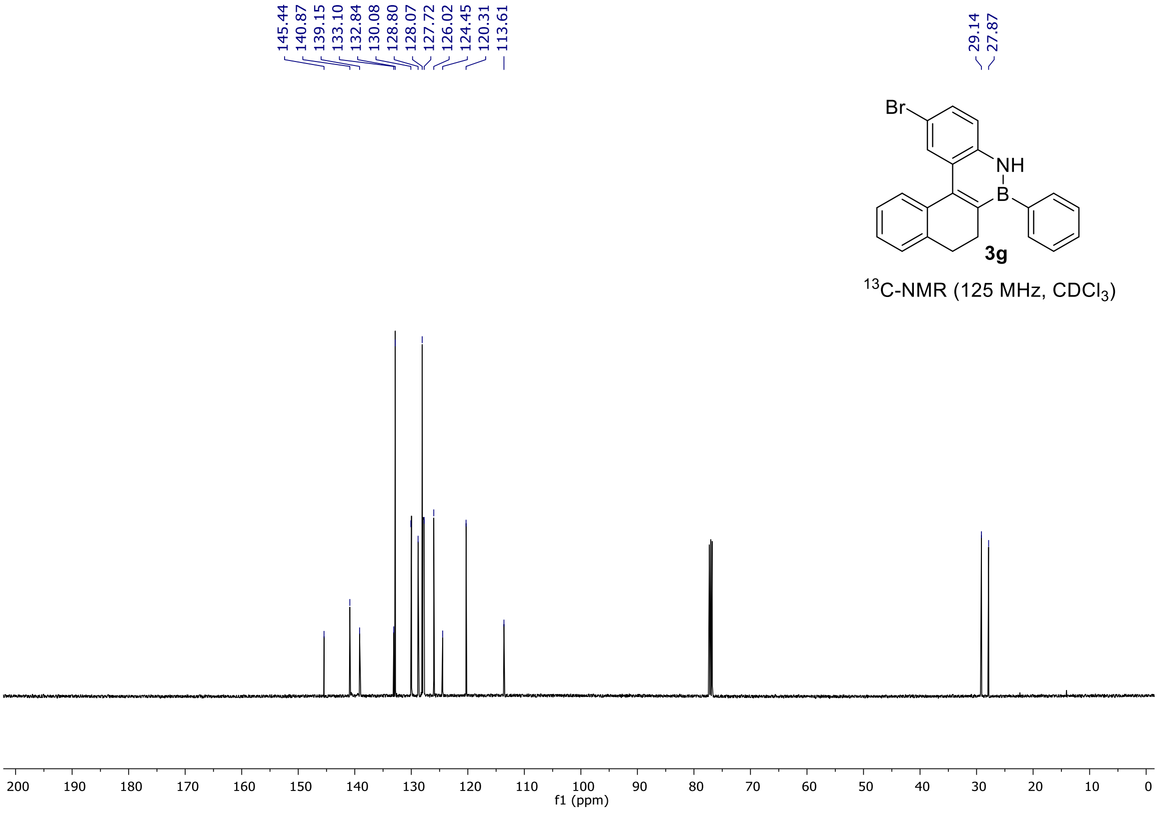
145.44 140.87 139.15 133.10 132.84 130.08 128.80 128.07 127.72 126.02 124.45 120.31 113.61
29.14 27.87
Br
NH
B
3g
13C-NMR (125 MHz, CDCl3)
f1 (ppm)

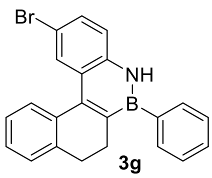

^{11}B-NMR (128 MHz, $CDCl_3$)

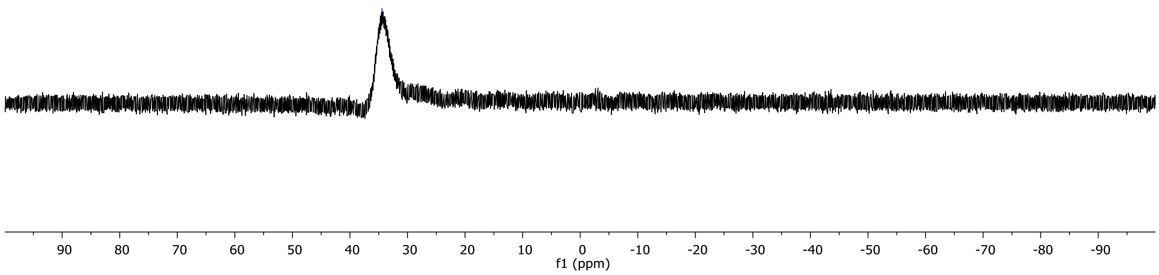

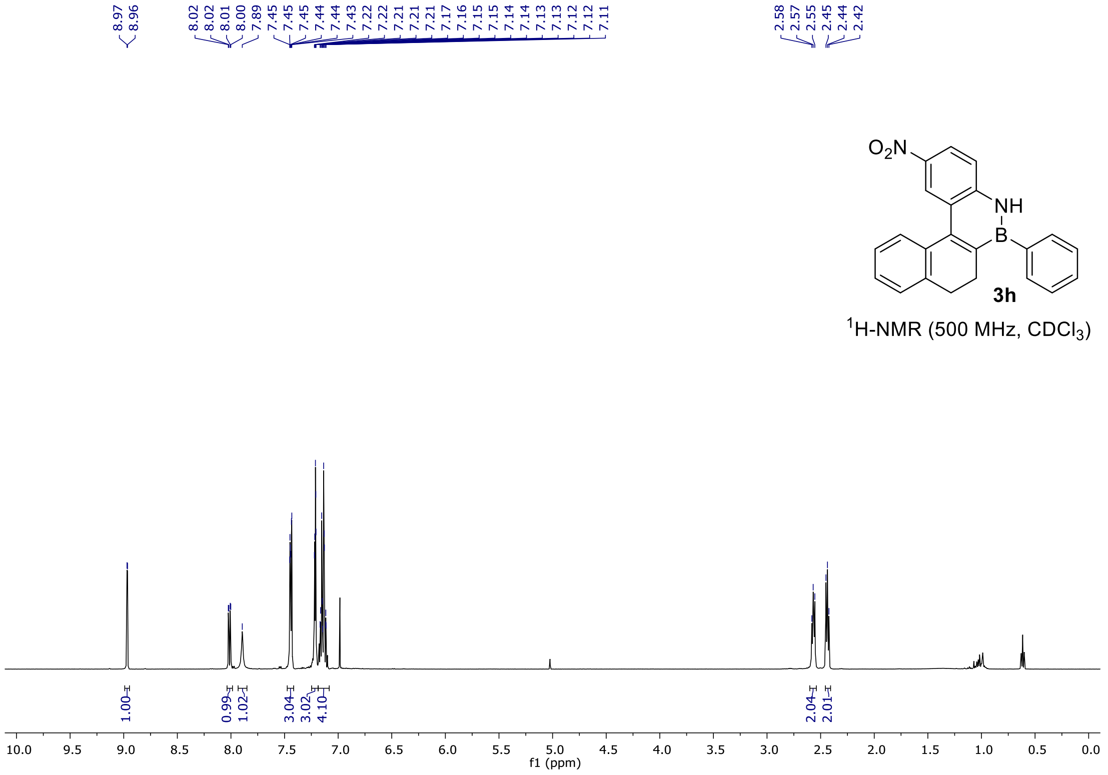

O2N
NH
B
3h
1H-NMR (500 MHz, CDCl3)
f1 (ppm)

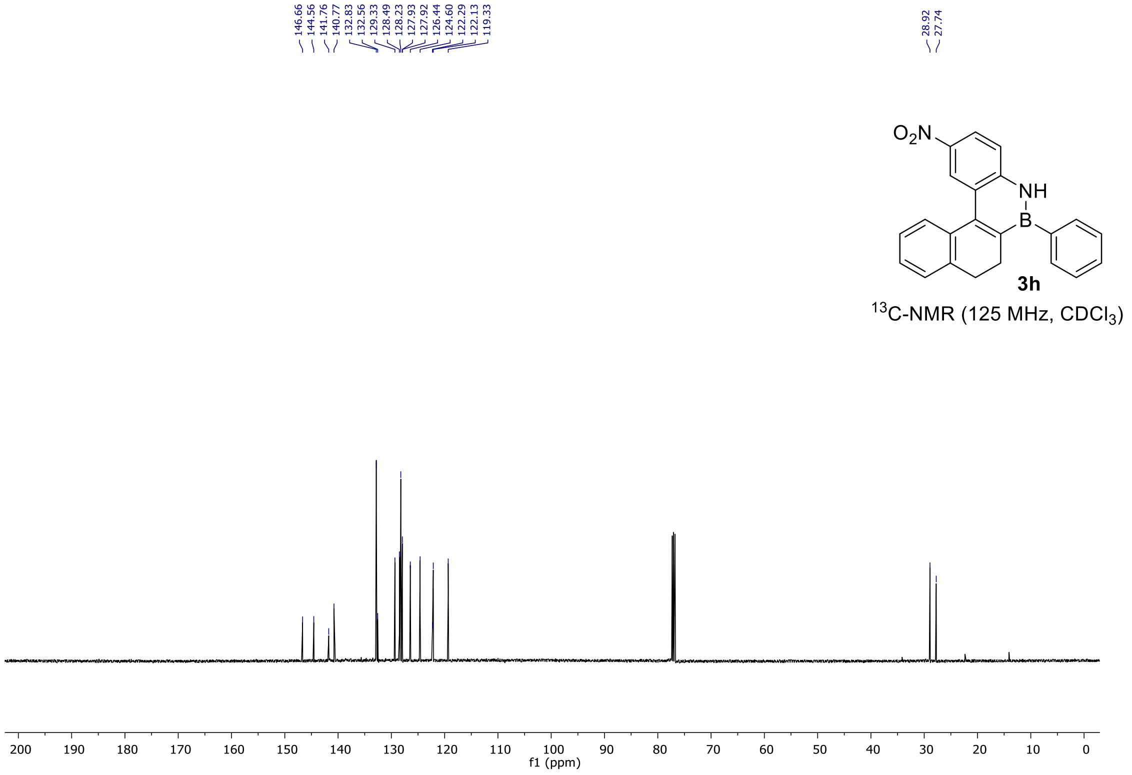

O2N
NH
B
3h
13C-NMR (125 MHz, CDCl3)
f1 (ppm)

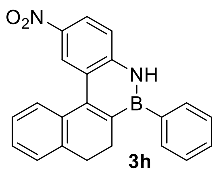

3h

^{11}B-NMR (128 MHz, $CDCl_3$)

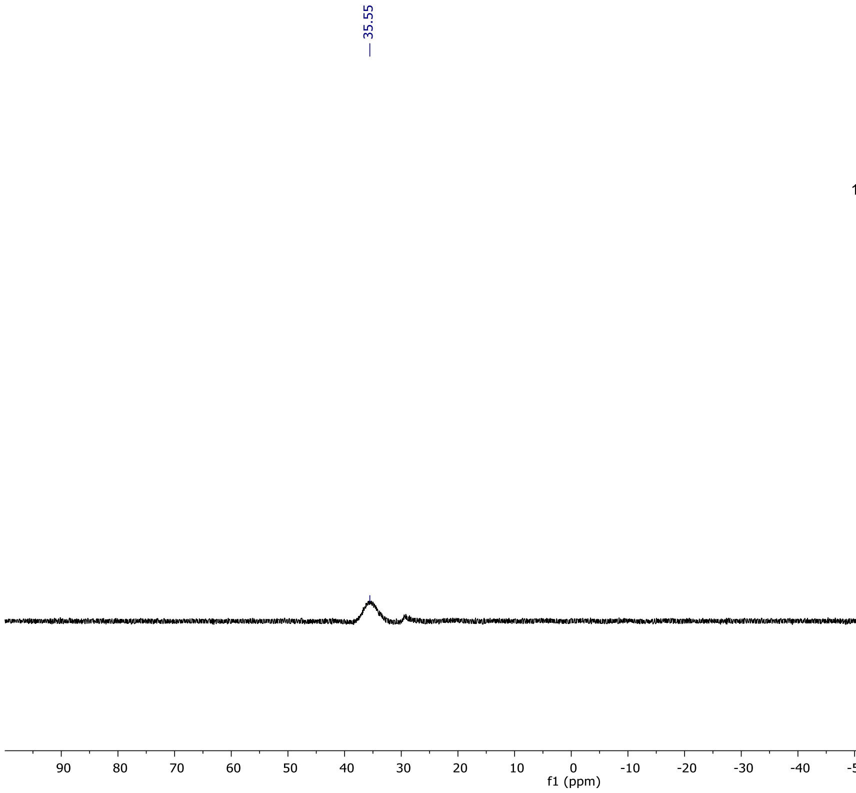

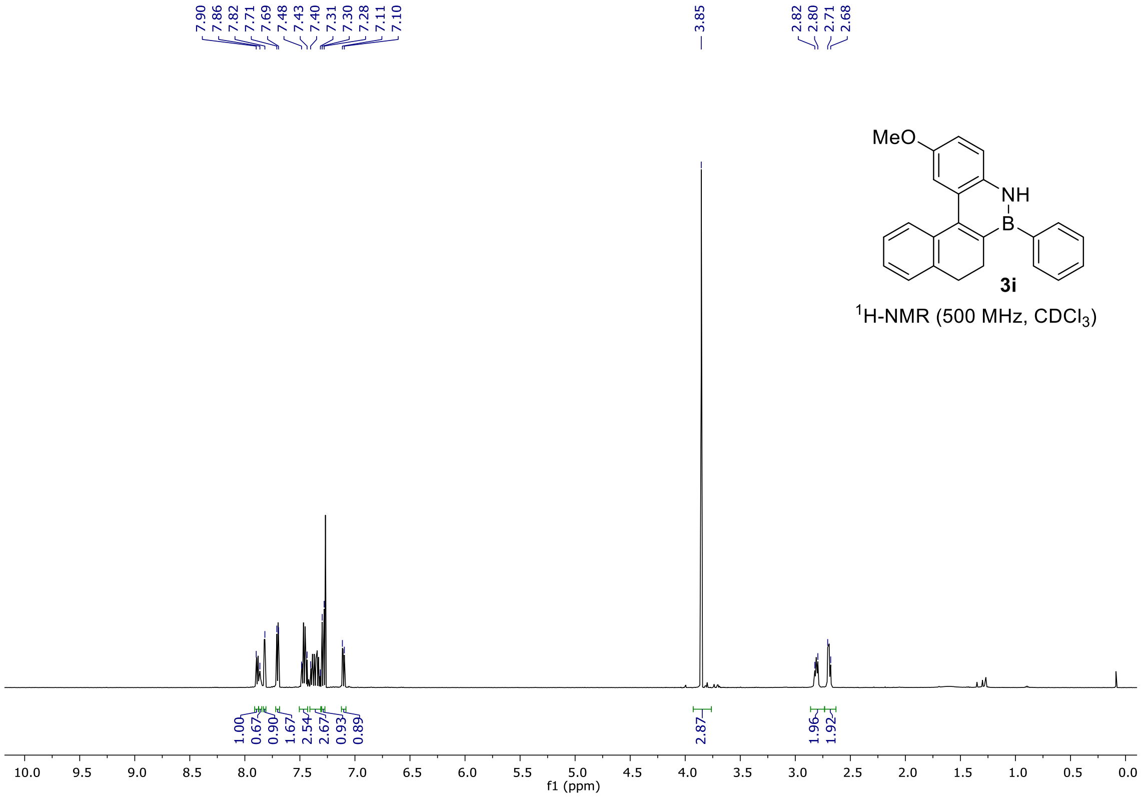
7.90
7.86
7.82
7.71
7.69
7.48
7.43
7.40
7.31
7.30
7.28
7.11
7.10
3.85
2.82
2.80
2.71
2.68
MeO
NH
B
3i
1H-NMR (500 MHz, CDCl3)
1.00
0.67
0.90
1.67
2.54
2.67
0.93
0.89
2.87
1.96
1.92
f1 (ppm)

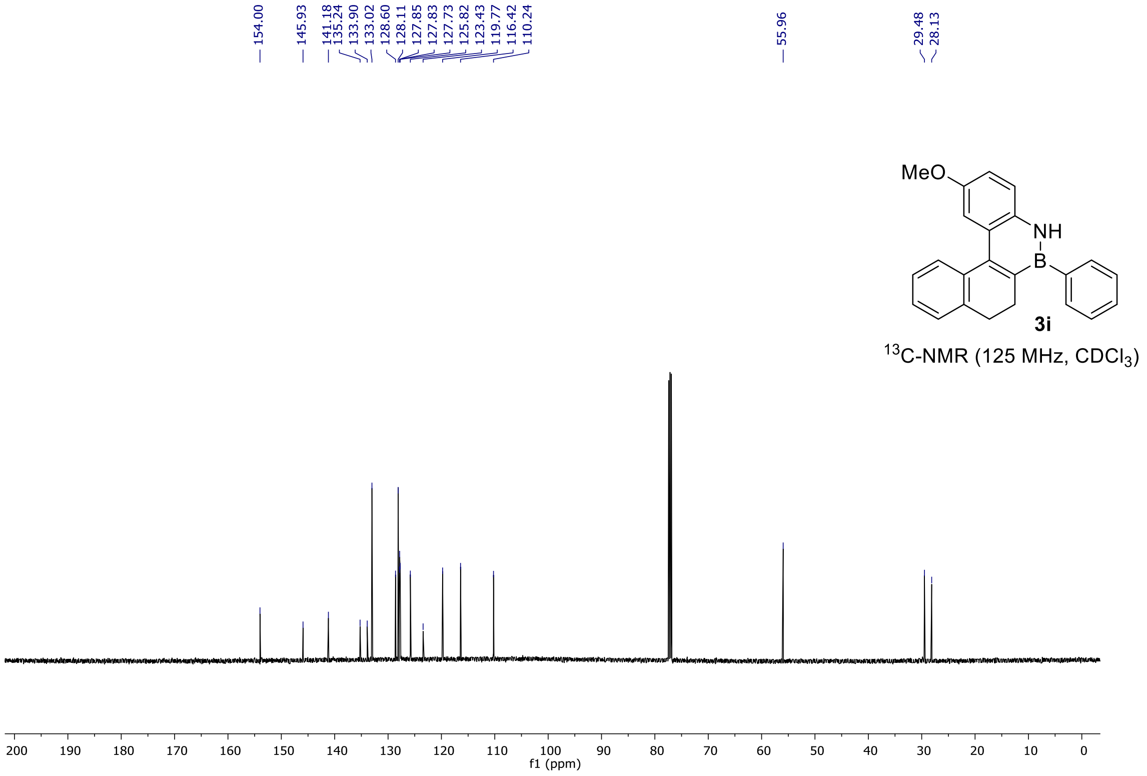
154.00
145.93
141.18
135.24
133.90
133.02
128.60
128.11
127.85
127.83
127.73
125.82
123.43
119.77
116.42
110.24
55.96
29.48
28.13
MeO
NH
B
3i
13C-NMR (125 MHz, CDCl3)
f1 (ppm)

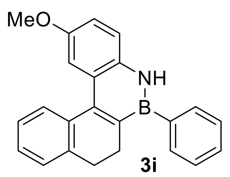

3i

^{11}B-NMR (128 MHz, $CDCl_3$)

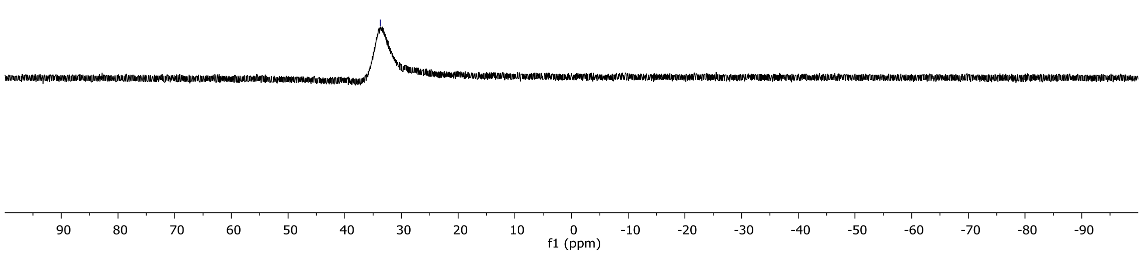

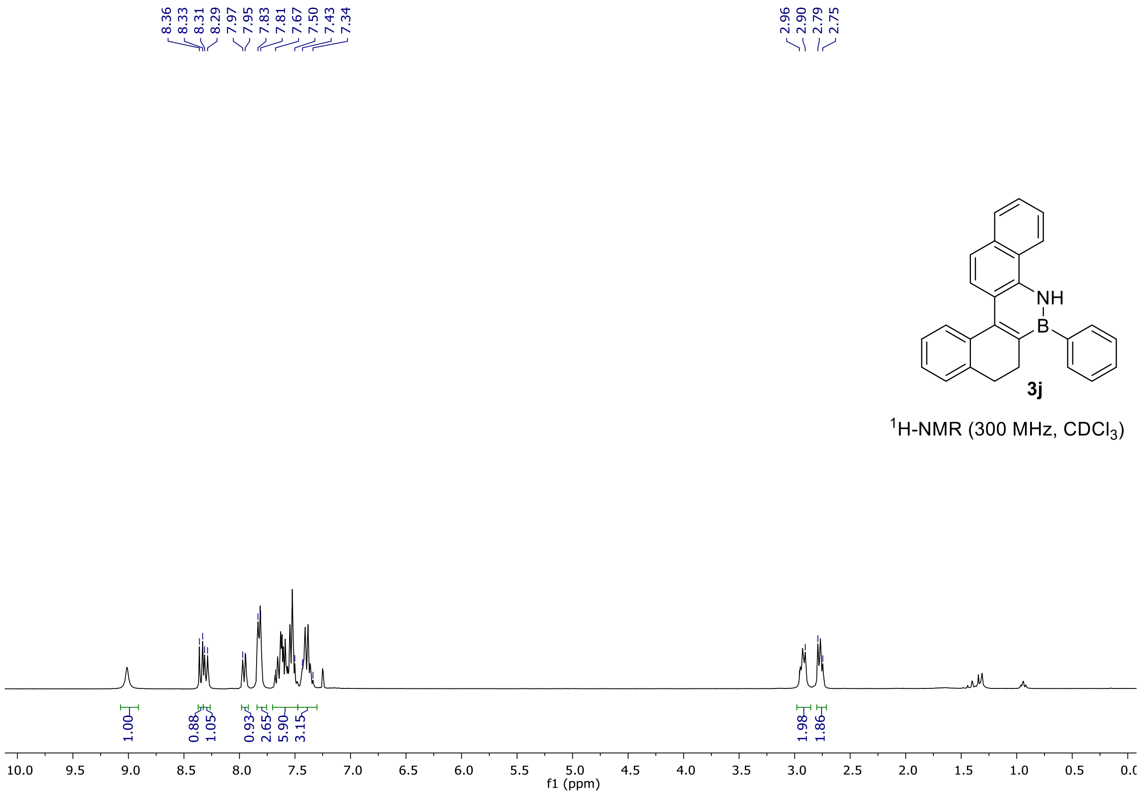
8.36 8.33 8.31 8.29 7.97 7.95 7.83 7.81 7.67 7.50 7.43 7.34
2.96 2.90 2.79 2.75
NH
B
3j
1H-NMR (300 MHz, CDCl3)
1.00 0.88 1.05 0.93 2.65 5.90 3.15 1.98 1.86
f1 (ppm)

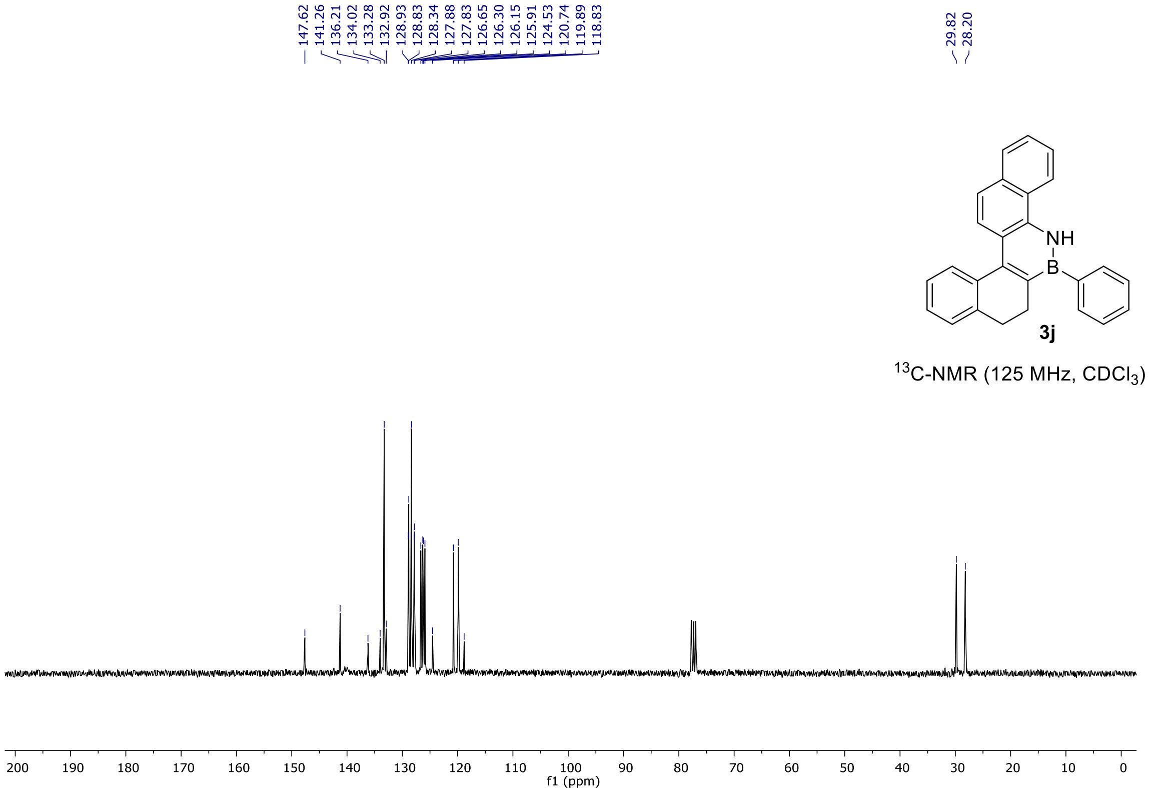
147.62 141.26 136.21 134.02 133.28 132.92 128.93 128.83 128.34 127.88 127.83 126.65 126.30 126.15 125.91 124.53 120.74 119.89 118.83
29.82 28.20
NH
B
3j
13C-NMR (125 MHz, CDCl3)
f1 (ppm)

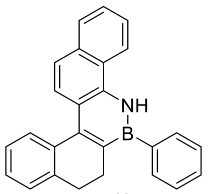

3j

^{11}B-NMR (128 MHz, $CDCl_3$)

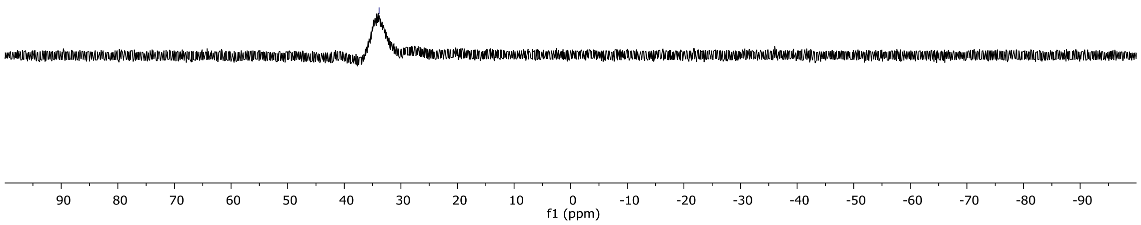

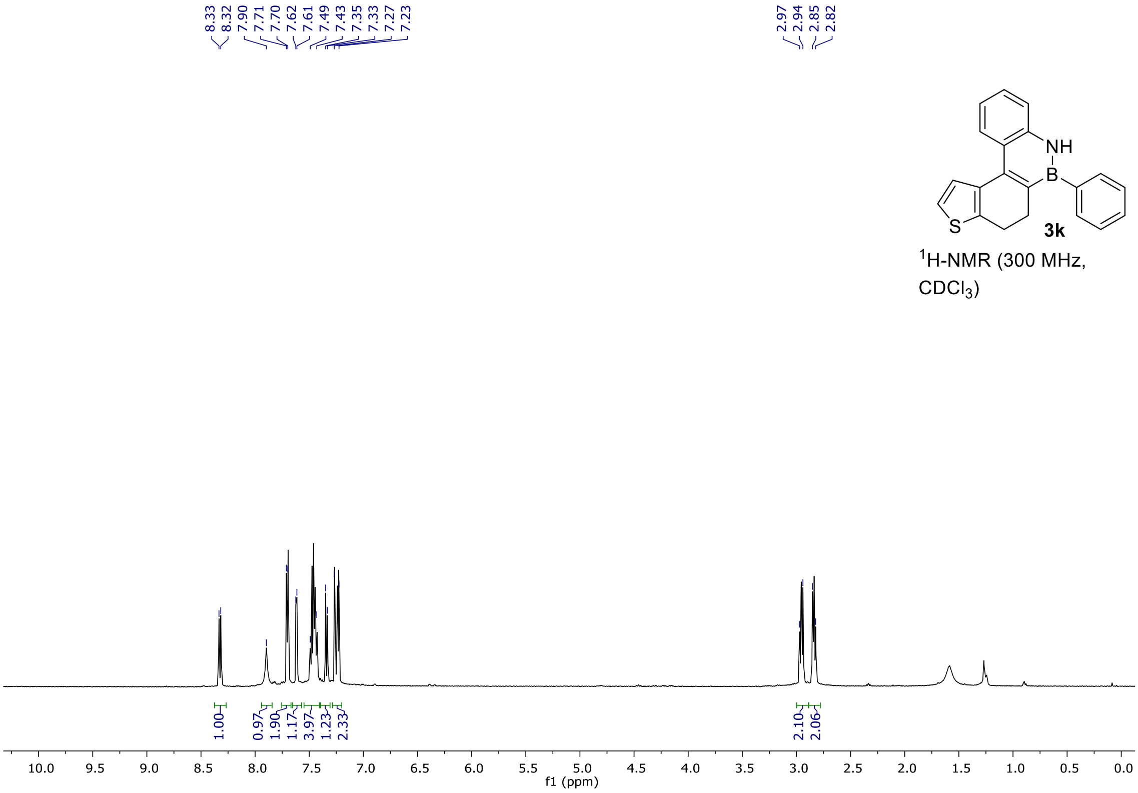

^{1}H-NMR (300 MHz, $CDCl_3$)

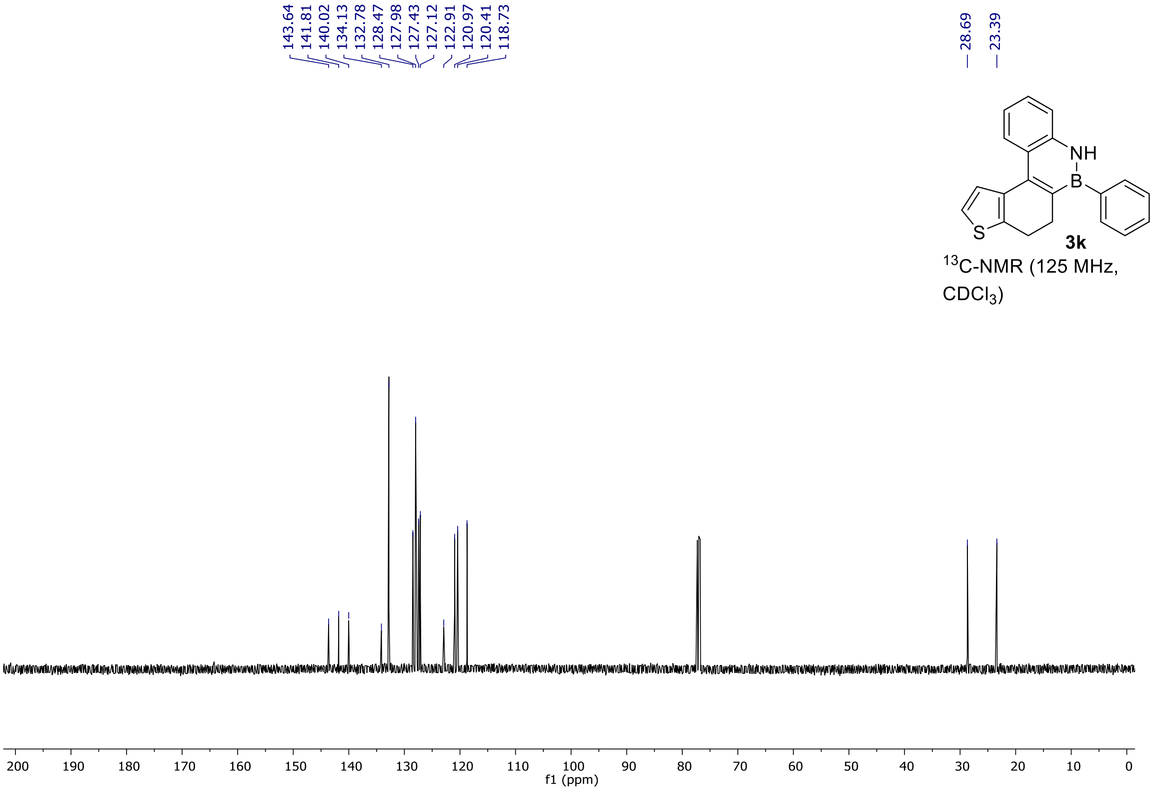

^{13}C-NMR (125 MHz, $CDCl_3$)

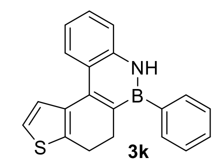

3k

^{11}B-NMR (128 MHz, $CDCl_3$)

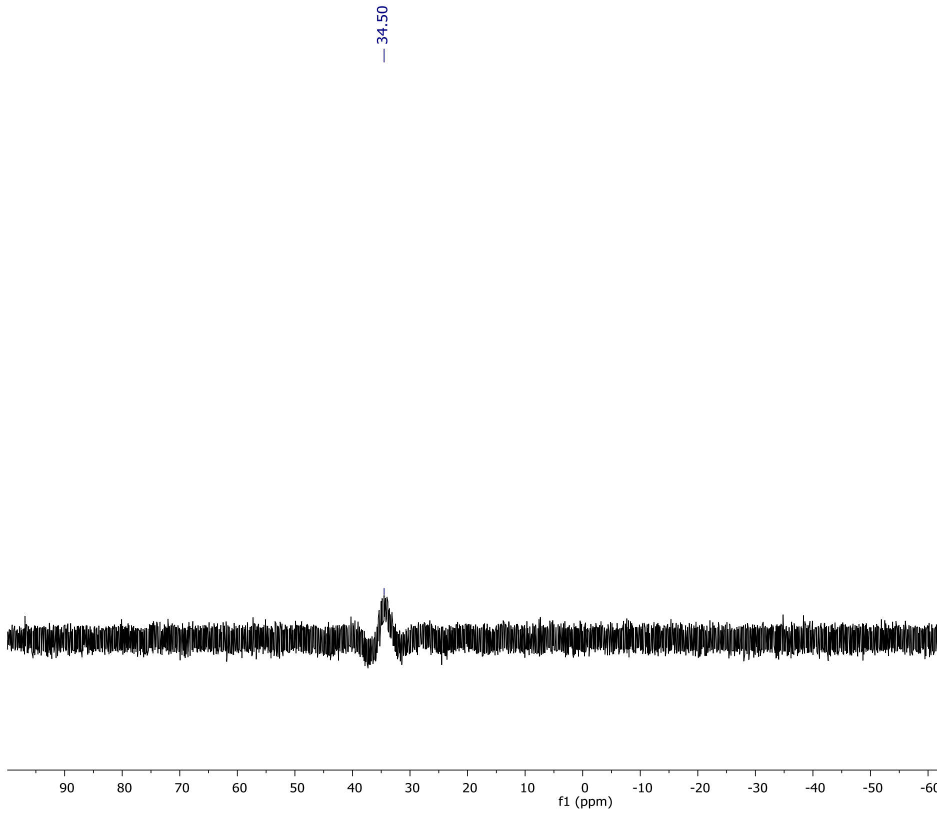

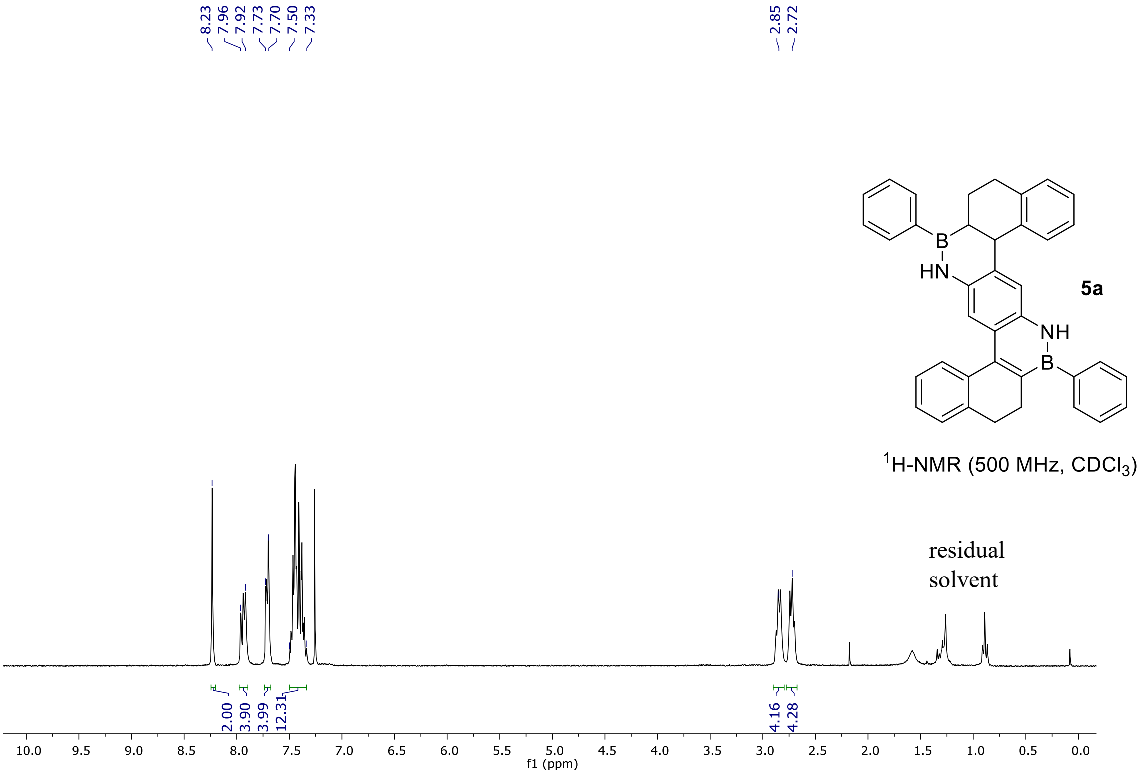
8.23
7.96
7.92
7.73
7.70
7.50
7.33
2.85
2.72
5a
^{1}H-NMR (500 MHz, CDCl$_3$)
residual solvent
2.00
3.90
3.99
12.31
4.16
4.28
f1 (ppm)

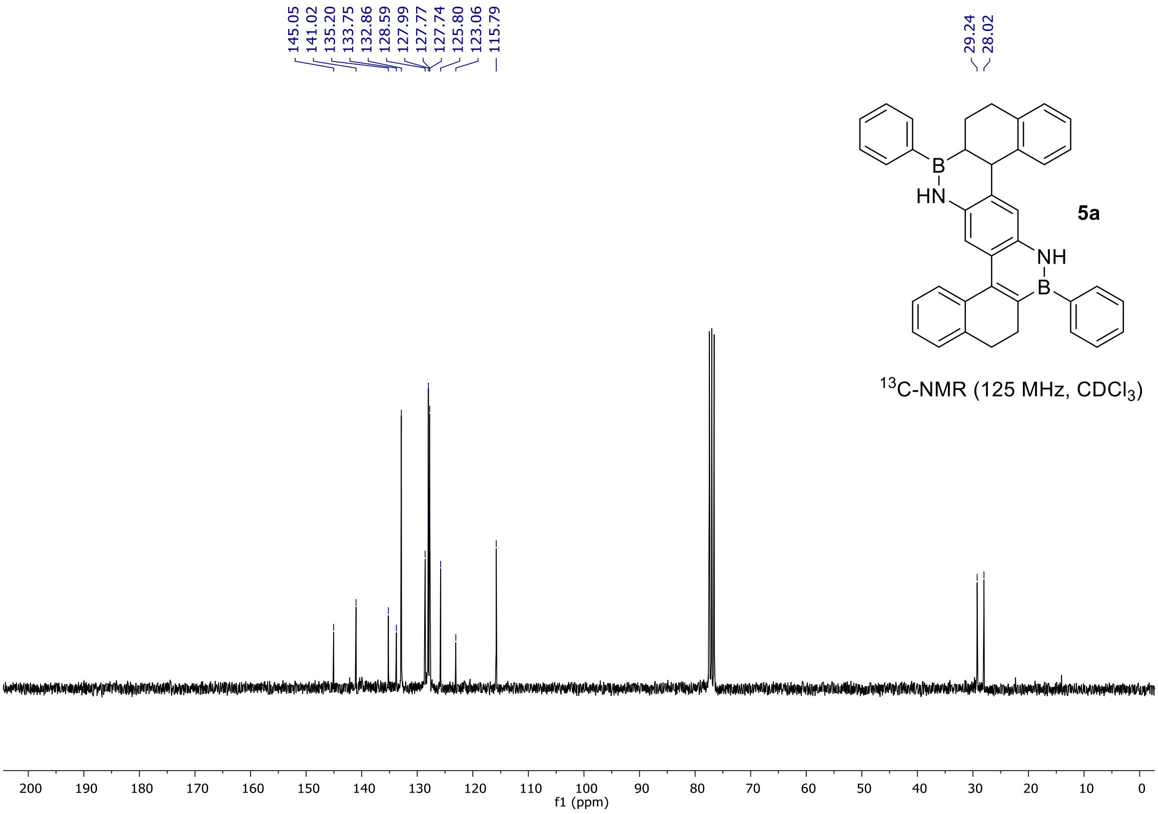
145.05
141.02
135.20
133.75
132.86
128.59
127.99
127.77
127.74
125.80
123.06
115.79
29.24
28.02
5a
^{13}C-NMR (125 MHz, CDCl$_3$)
f1 (ppm)

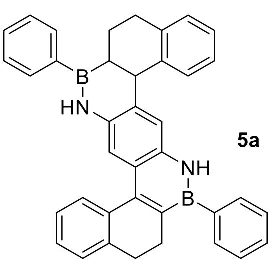

5a

^{10}B-NMR (128 MHz, $CDCl_3$)

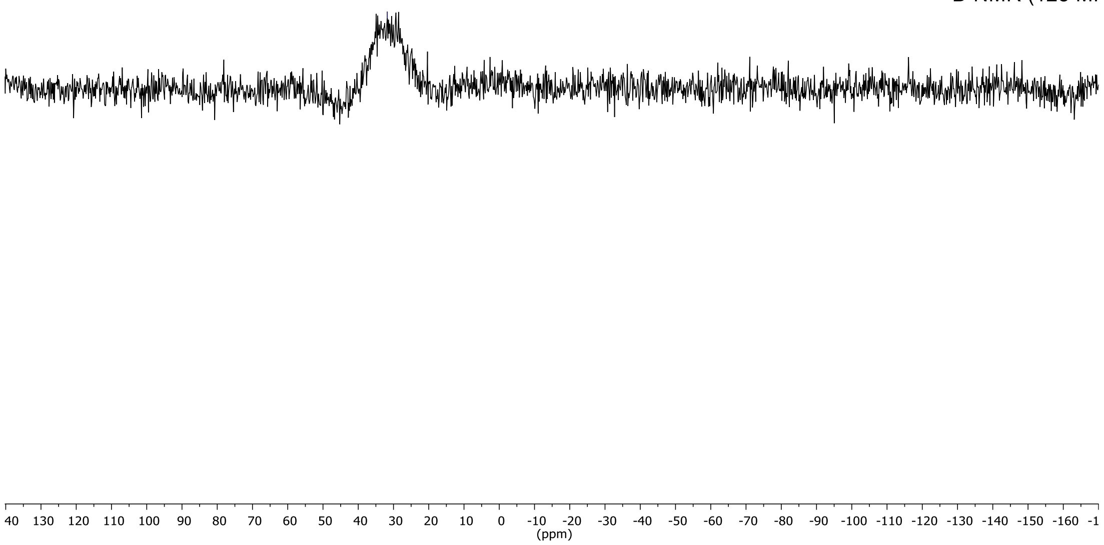

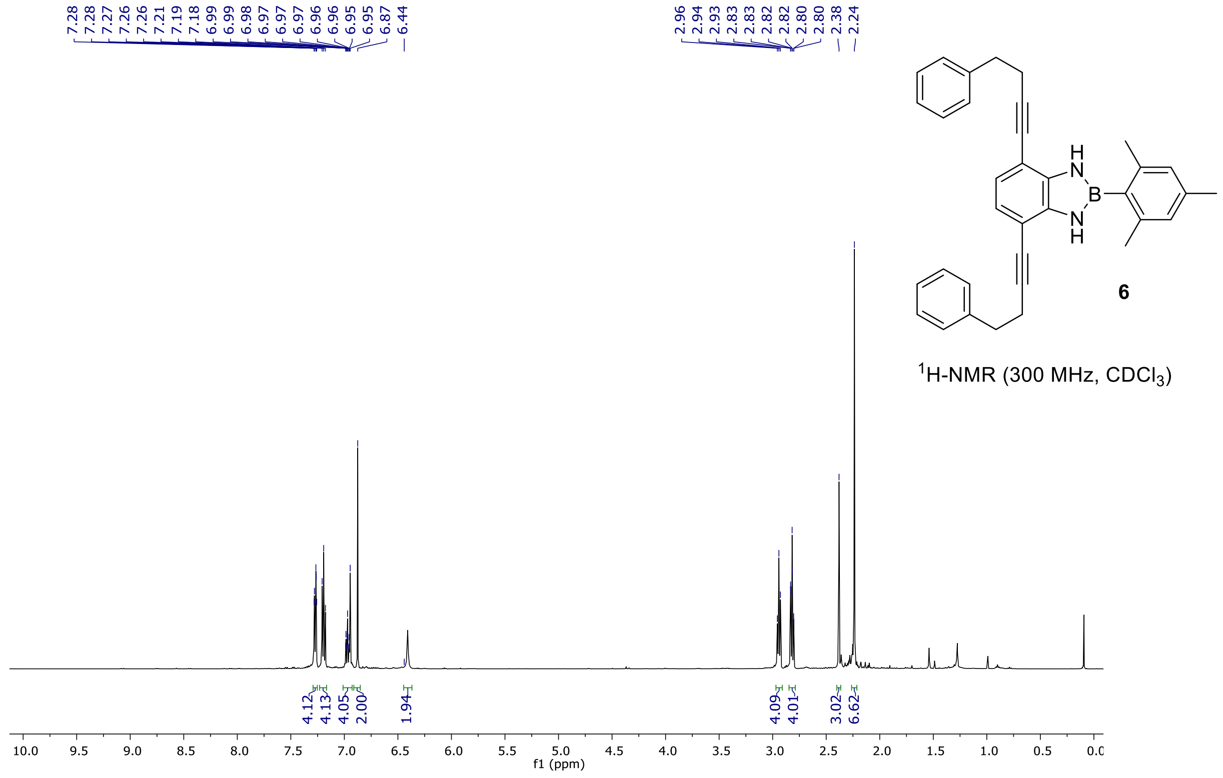
7.28 7.28 7.27 7.26 7.26 7.21 7.19 7.18 6.99 6.99 6.98 6.97 6.97 6.97 6.96 6.96 6.95 6.95 6.87 6.44
2.96 2.94 2.93 2.83 2.83 2.82 2.82 2.80 2.80 2.38 2.24
H
N
B
N
H
6
1H-NMR (300 MHz, CDCl3)
4.12 4.13 4.05 2.00 1.94 4.09 4.01 3.02 6.62
10.0 9.5 9.0 8.5 8.0 7.5 7.0 6.5 6.0 5.5 5.0 4.5 4.0 3.5 3.0 2.5 2.0 1.5 1.0 0.5 0.0
f1 (ppm)

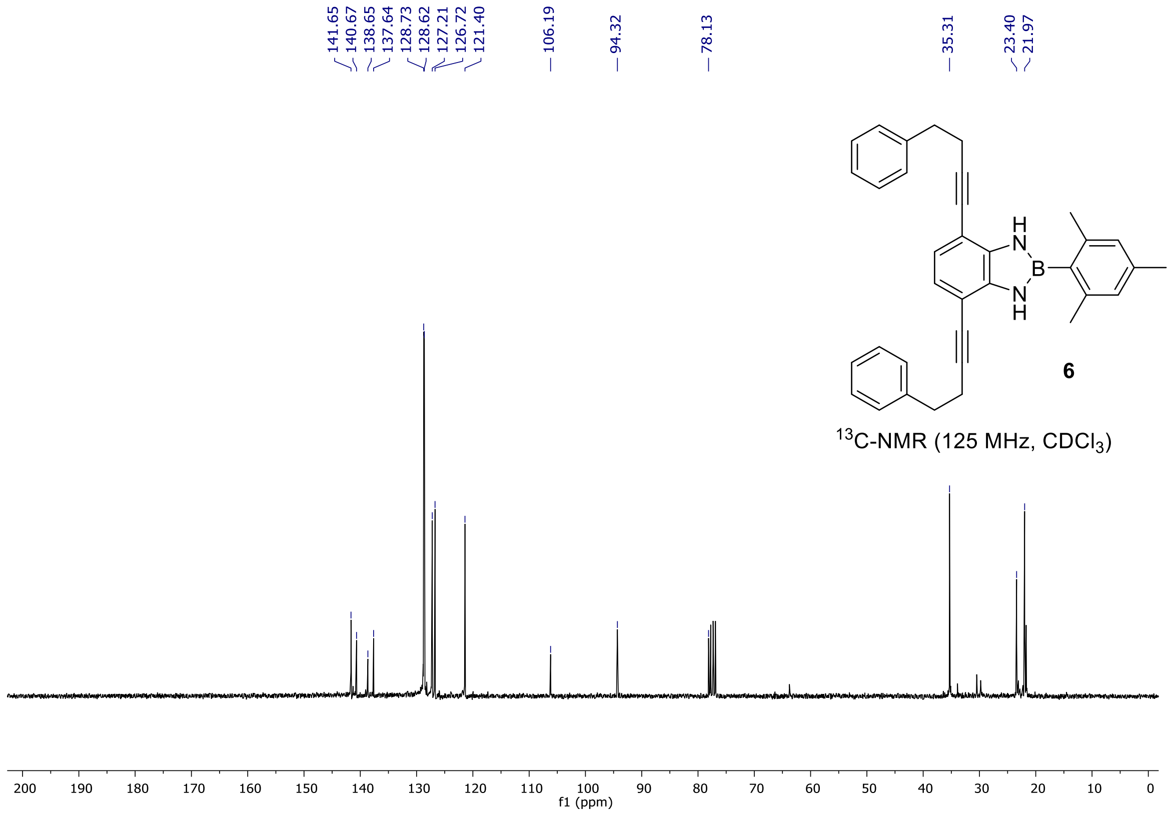
141.65 140.67 138.65 137.64 128.73 128.62 127.21 126.72 121.40
106.19
94.32
78.13
35.31
23.40 21.97
H
N
B
N
H
6
13C-NMR (125 MHz, CDCl3)
200 190 180 170 160 150 140 130 120 110 100 90 80 70 60 50 40 30 20 10 0
f1 (ppm)

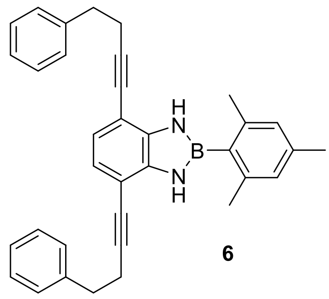

6

^{11}B-NMR (128 MHz, $CDCl_3$)

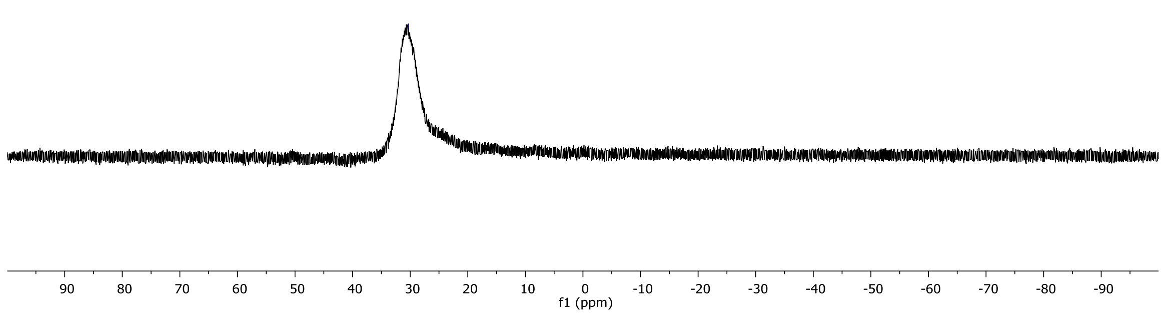

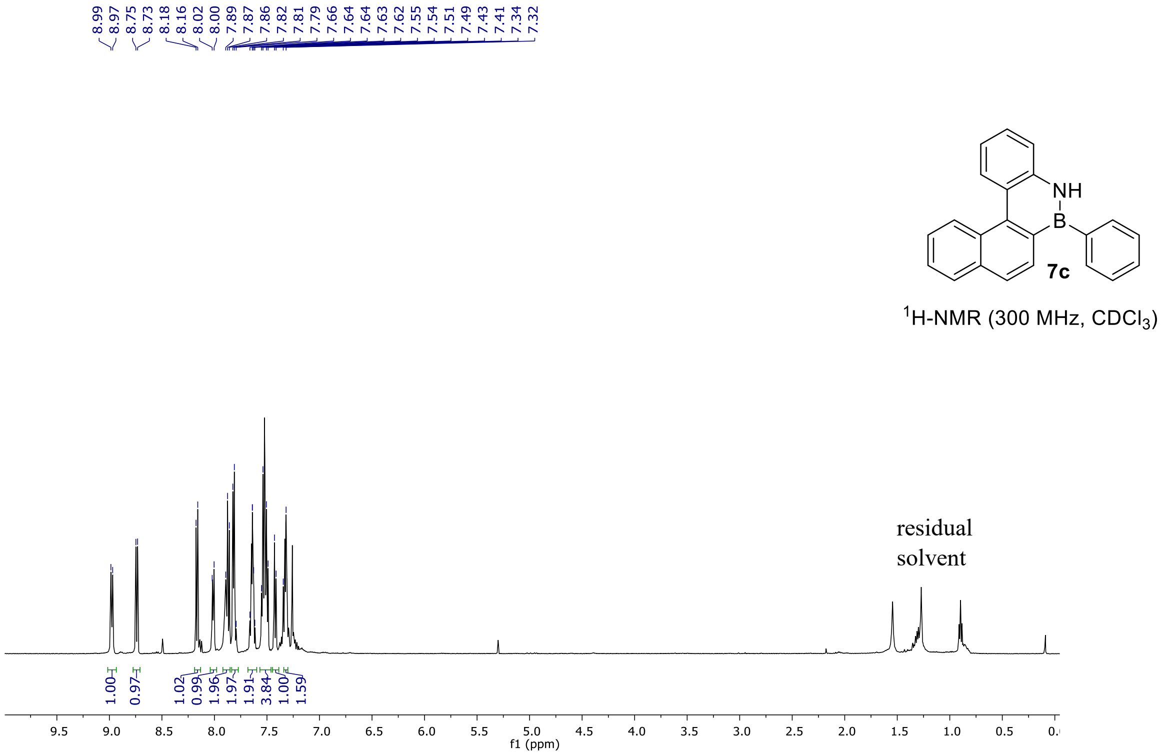
8.99 8.97 8.75 8.73 8.18 8.16 8.02 8.00 7.89 7.87 7.86 7.82 7.81 7.79 7.66 7.64 7.64 7.63 7.62 7.55 7.54 7.51 7.49 7.43 7.41 7.34 7.32
NH
B
7c
^{1}H-NMR (300 MHz, $CDCl_3$)
residual
solvent
1.00 0.97 1.02 0.99 1.96 1.97 1.91 3.84 1.00 1.59
9.5 9.0 8.5 8.0 7.5 7.0 6.5 6.0 5.5 5.0 4.5 4.0 3.5 3.0 2.5 2.0 1.5 1.0 0.5
f1 (ppm)

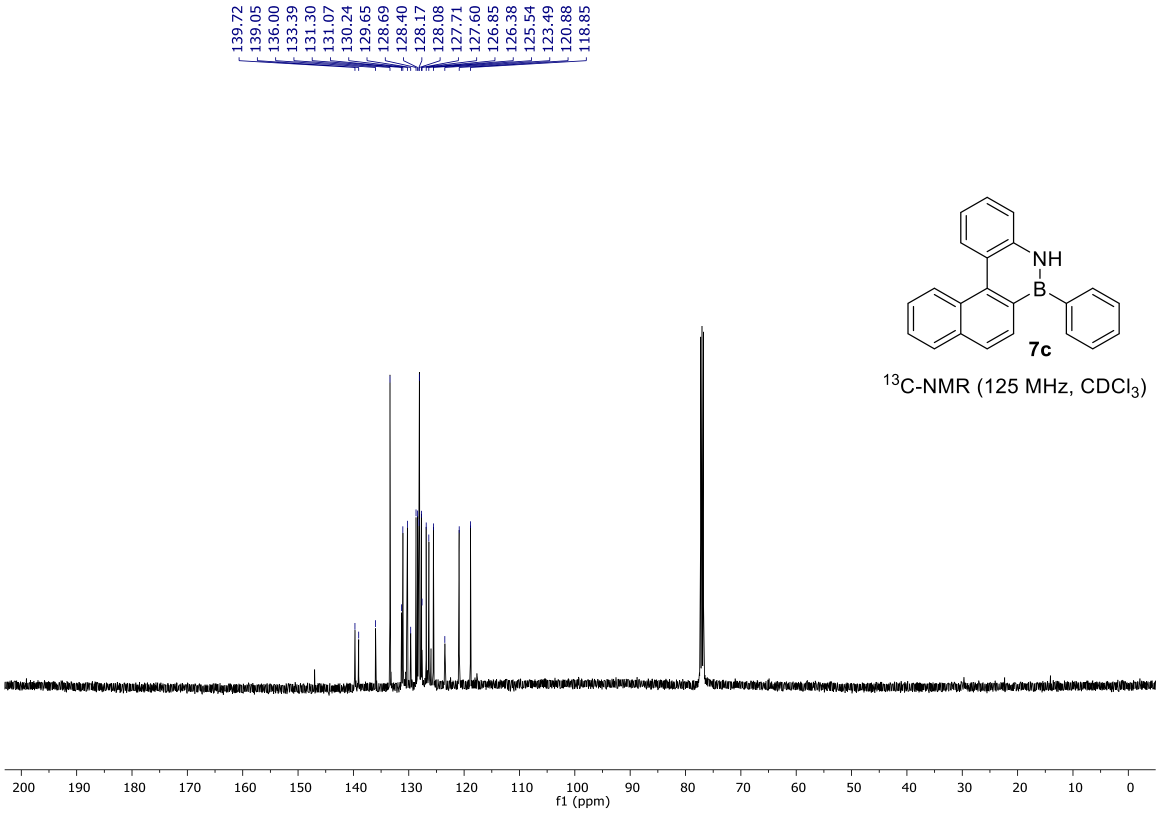
139.72 139.05 136.00 133.39 131.30 131.07 130.24 129.65 128.69 128.40 128.17 128.08 127.71 127.60 126.85 126.38 125.54 123.49 120.88 118.85
NH
B
7c
^{13}C-NMR (125 MHz, $CDCl_3$)
200 190 180 170 160 150 140 130 120 110 100 90 80 70 60 50 40 30 20 10 0
f1 (ppm)

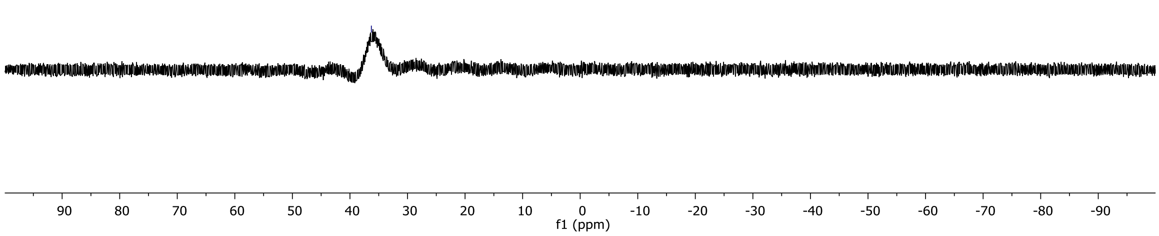

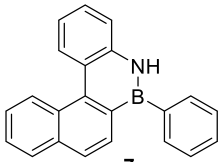

7c

^{11}B-NMR (128 MHz, $CDCl_3$)

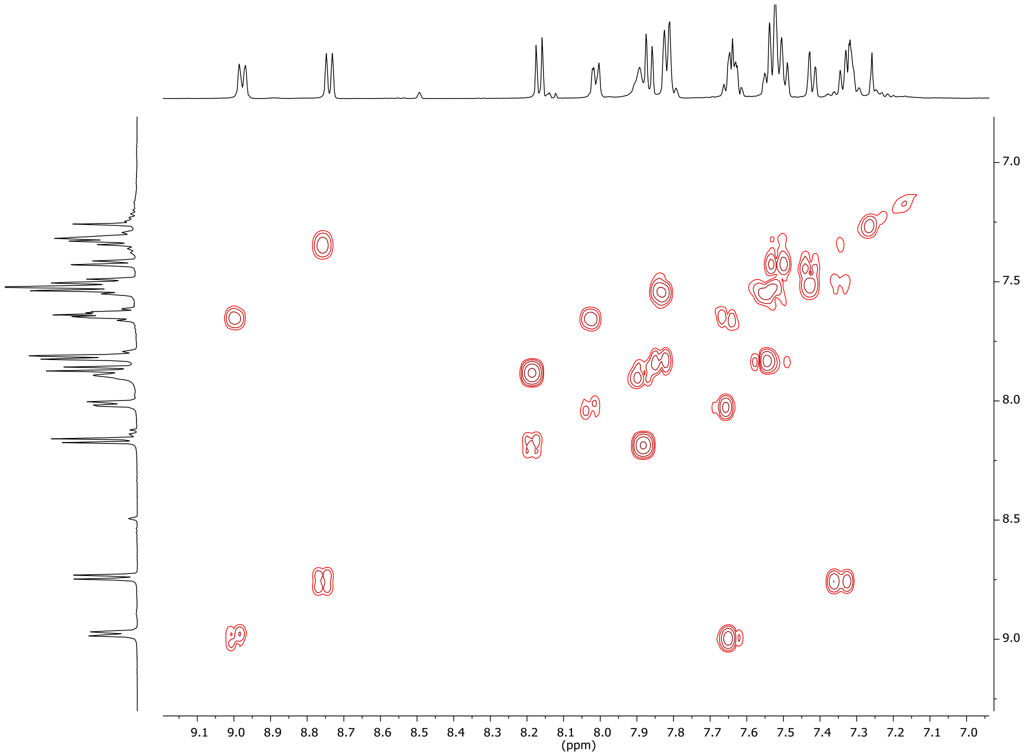

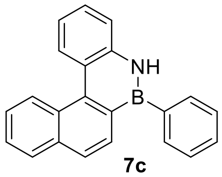

7c

COSY-NMR ($CDCl_3$)

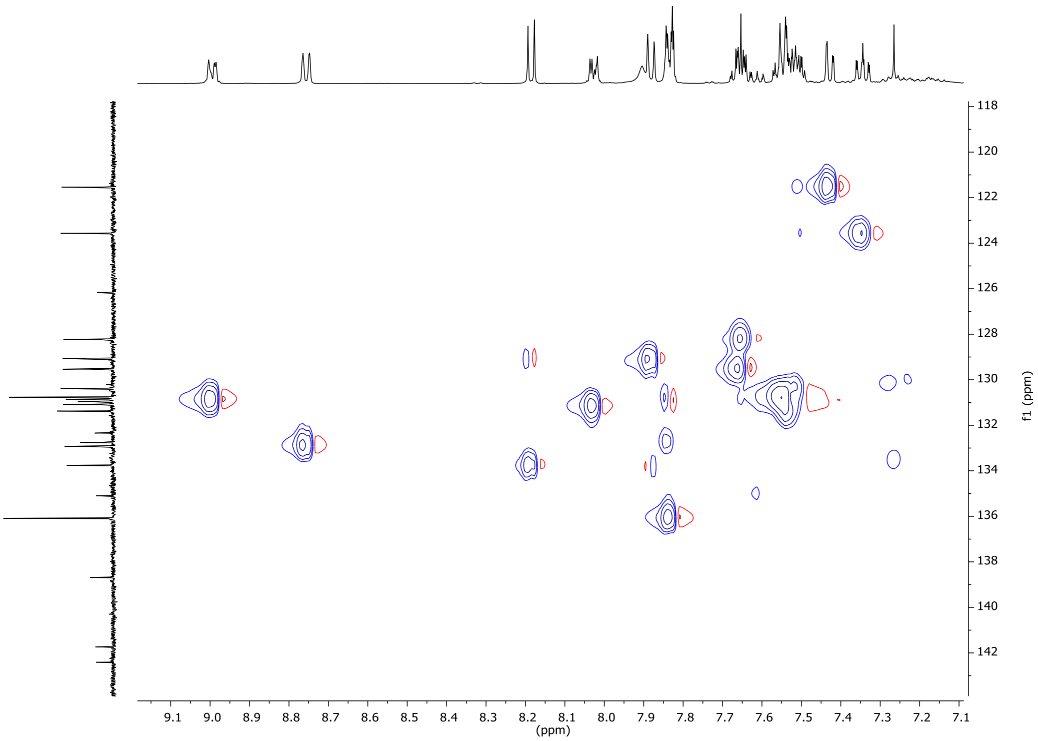

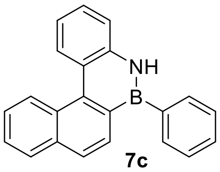

7c

HSQC-NMR ($CDCl_3$)

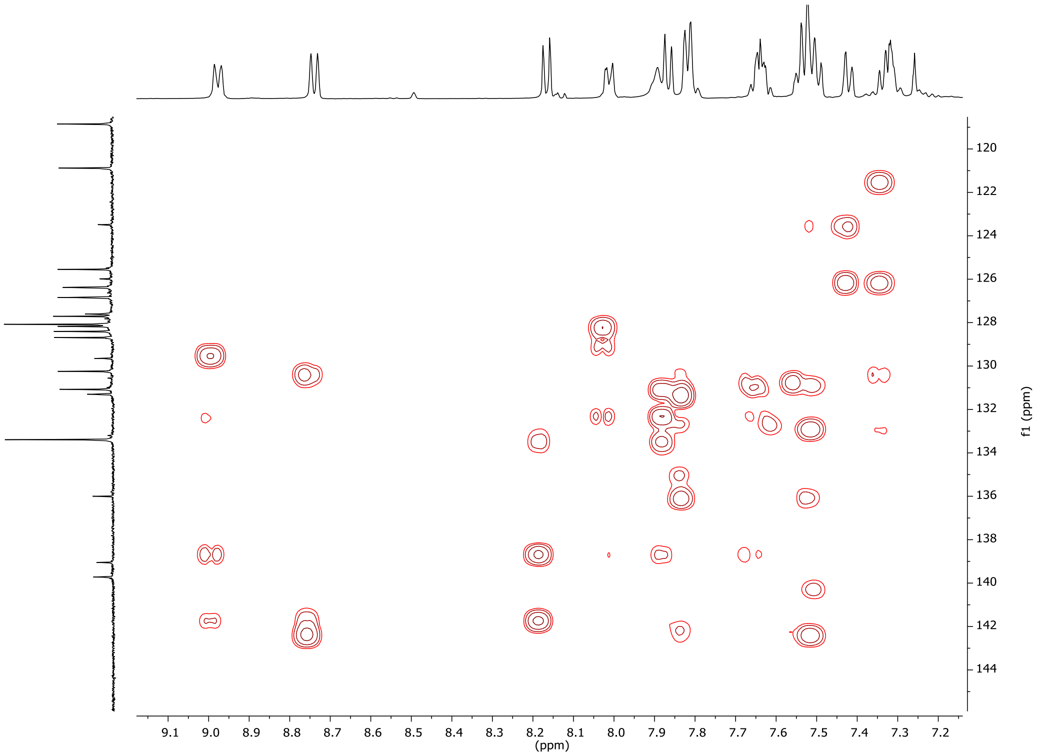

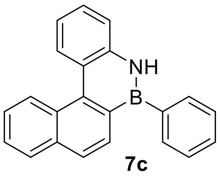

7c

HMBC-NMR ($CDCl_3$)

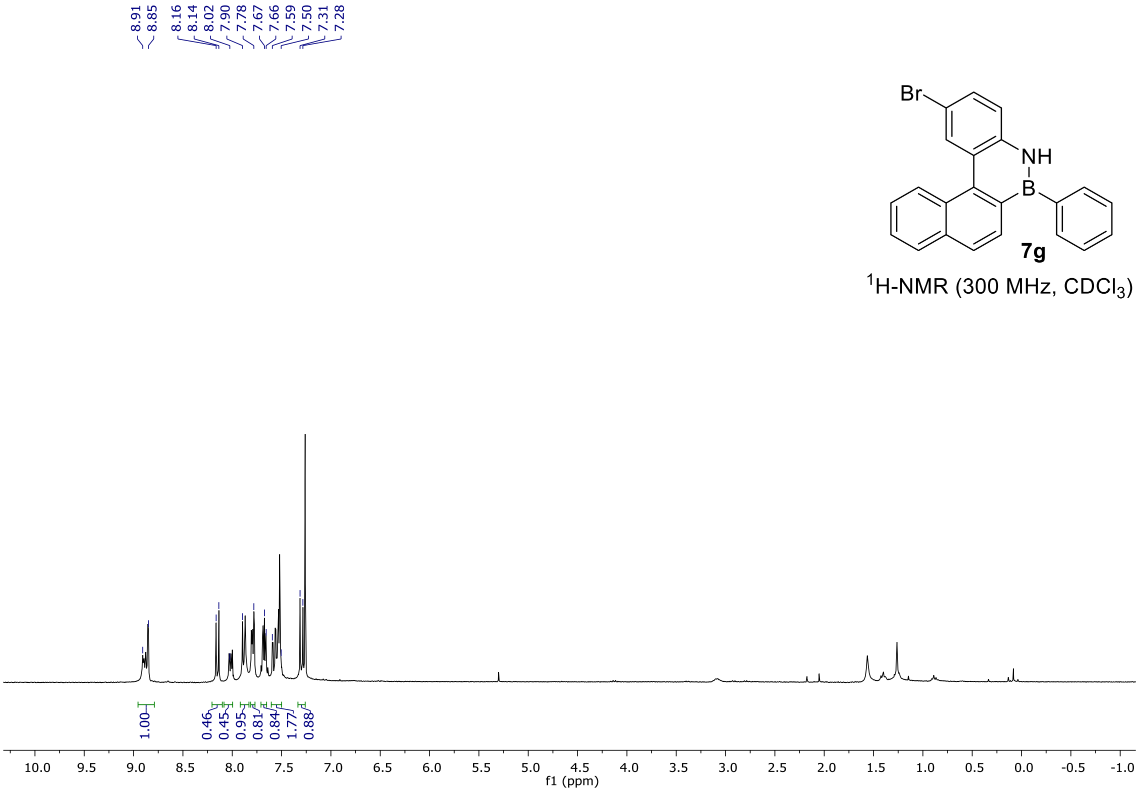

7g

^{1}H-NMR (300 MHz, $CDCl_3$)

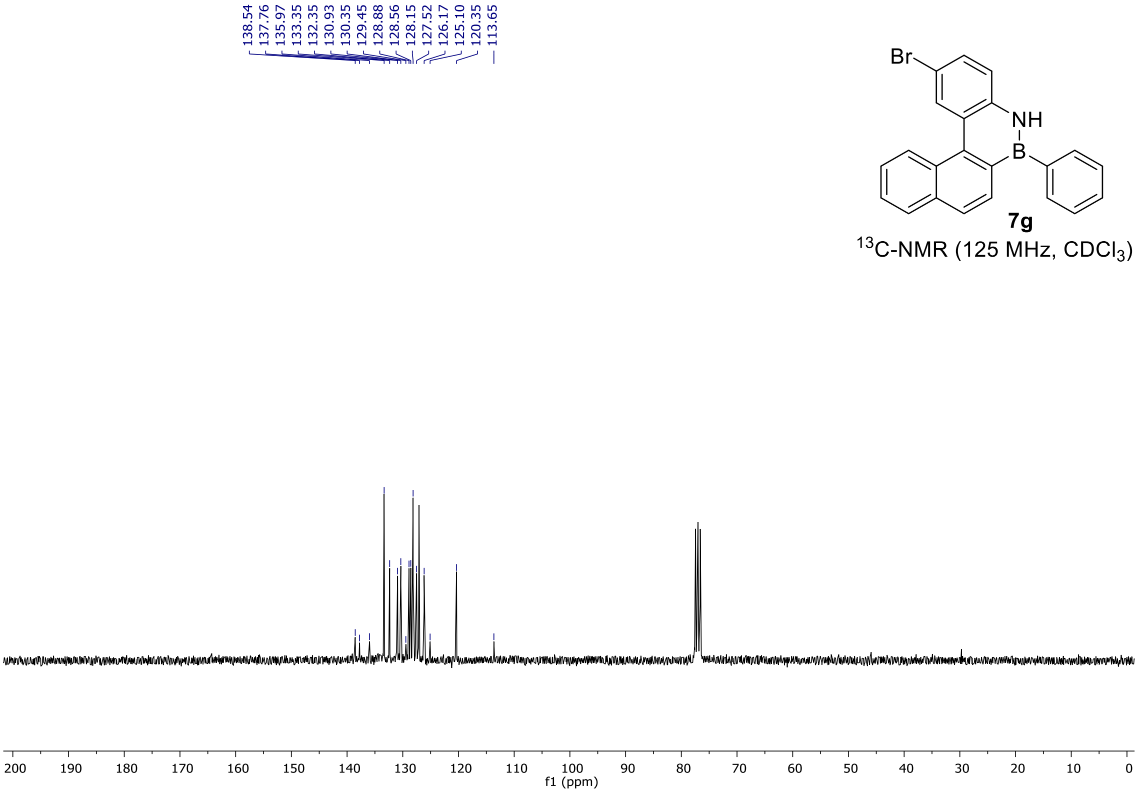

7g

^{13}C-NMR (125 MHz, $CDCl_3$)

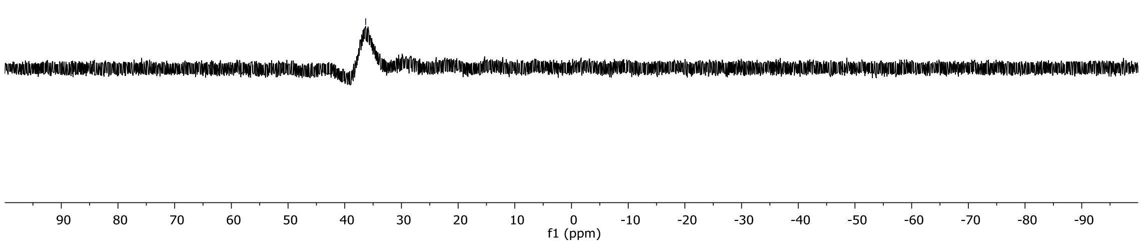

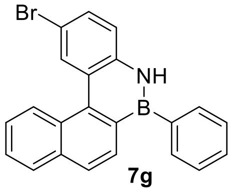

7g

^{11}B-NMR (128 MHz, $CDCl_3$)

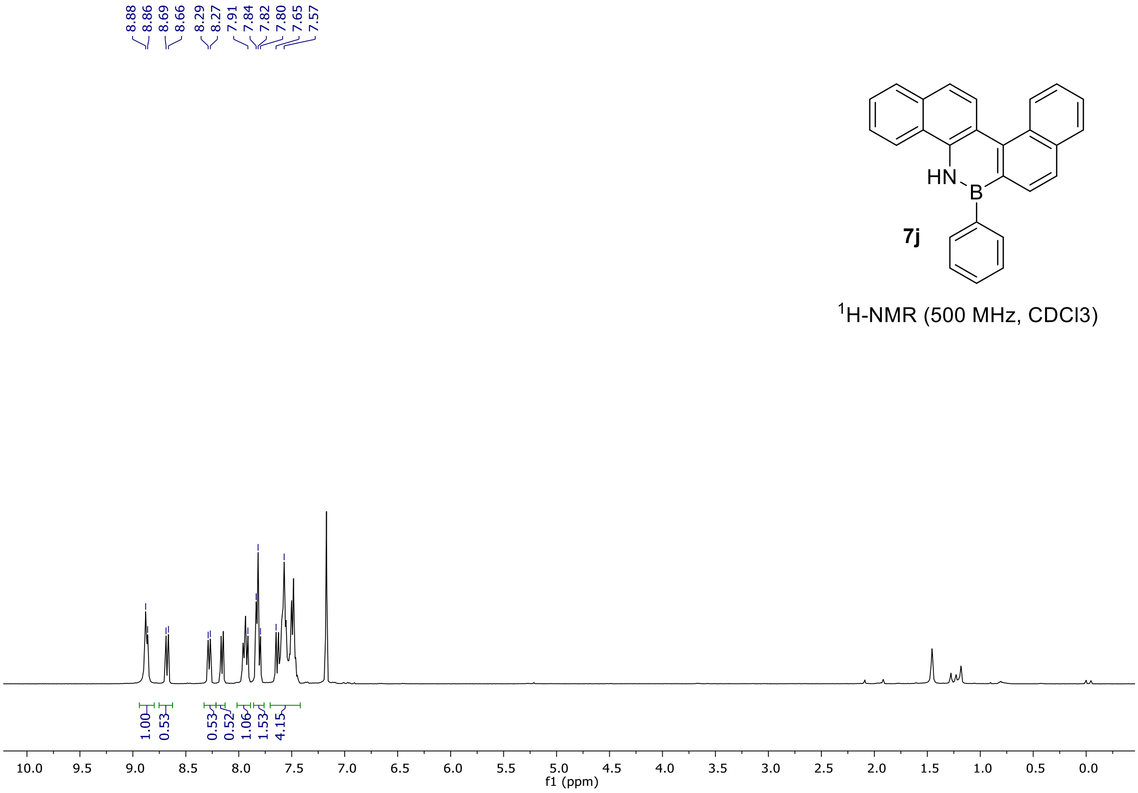

7j

^{1}H-NMR (500 MHz, CDCl3)

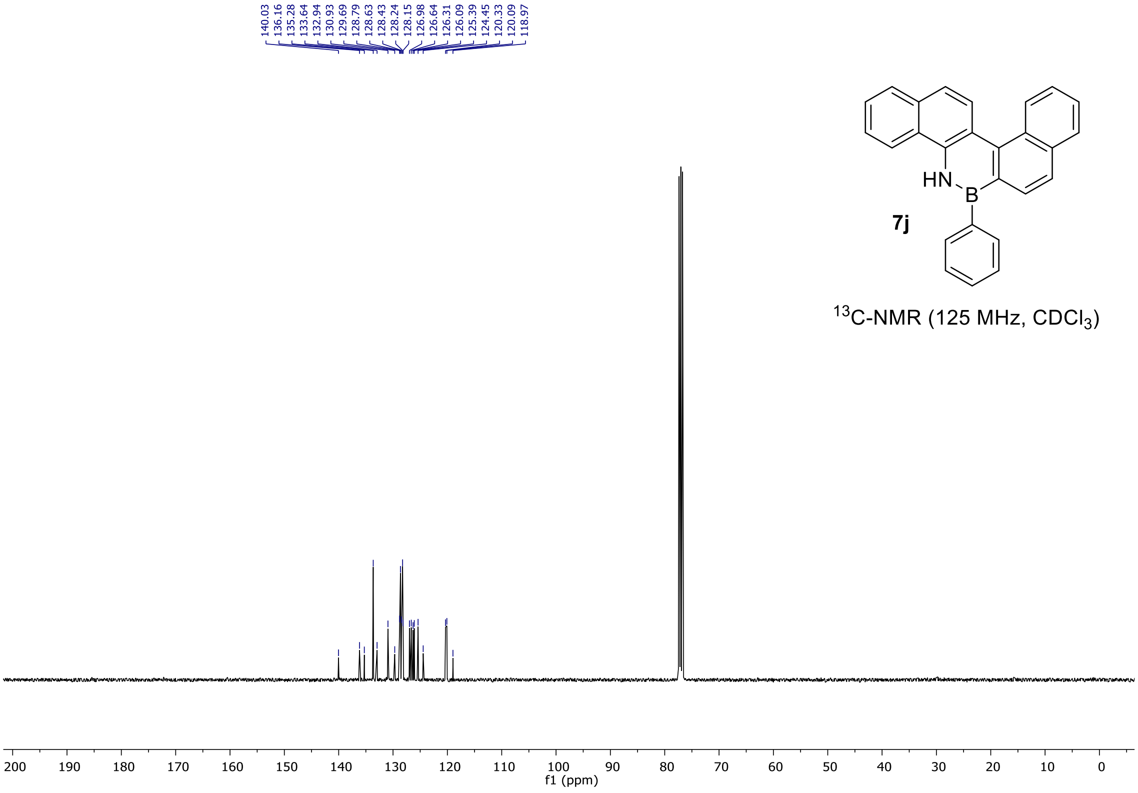

7j

^{13}C-NMR (125 MHz, $CDCl_3$)

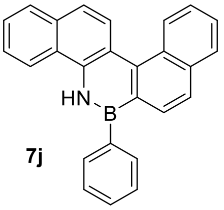

7j

^{11}B-NMR (128 MHz, $CDCl_3$)

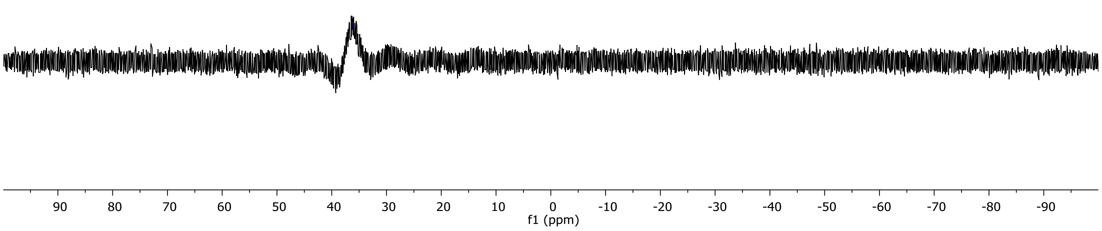

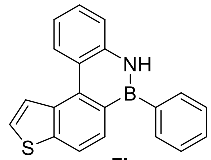

7k

^{1}H-NMR (500 MHz, $CDCl_3$)

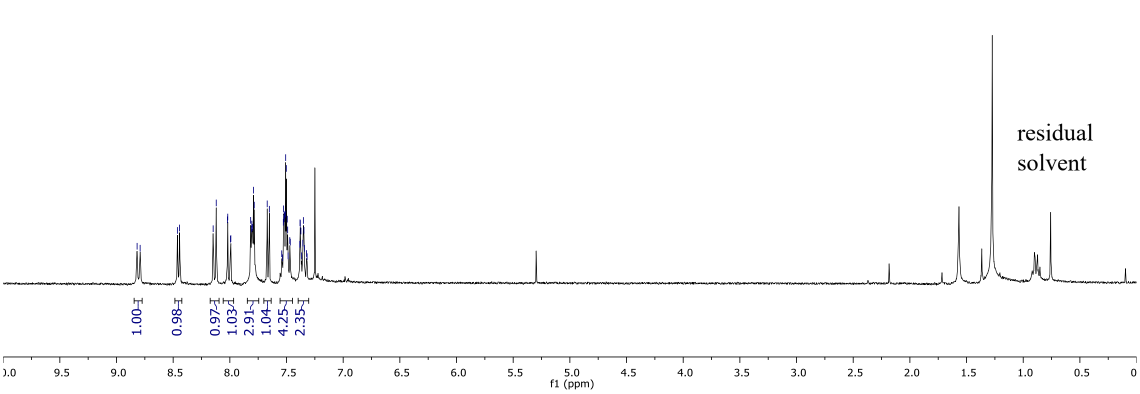

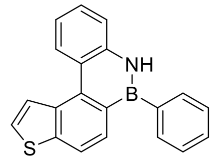

7k

^{13}C-NMR (125 MHz, $CDCl_3$)

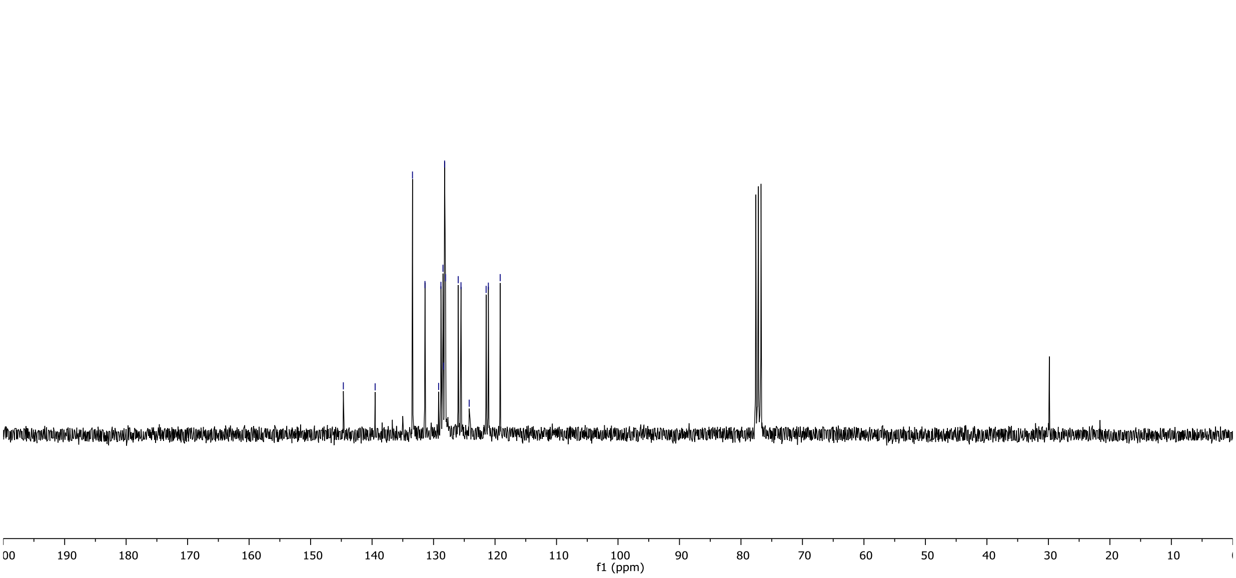

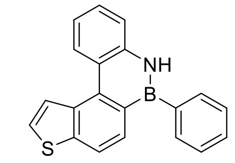

7k

^{11}B-NMR (128 MHz, $CDCl_3$)

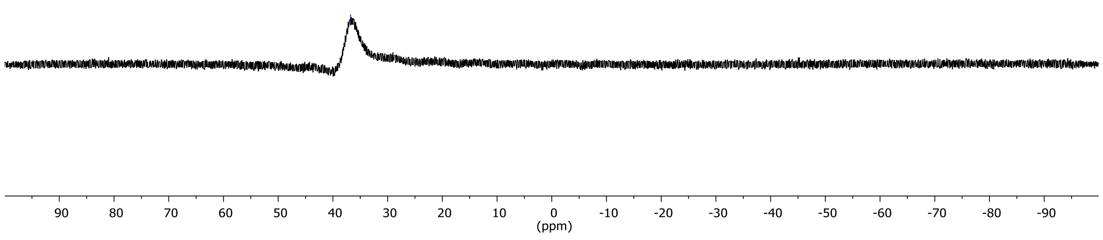

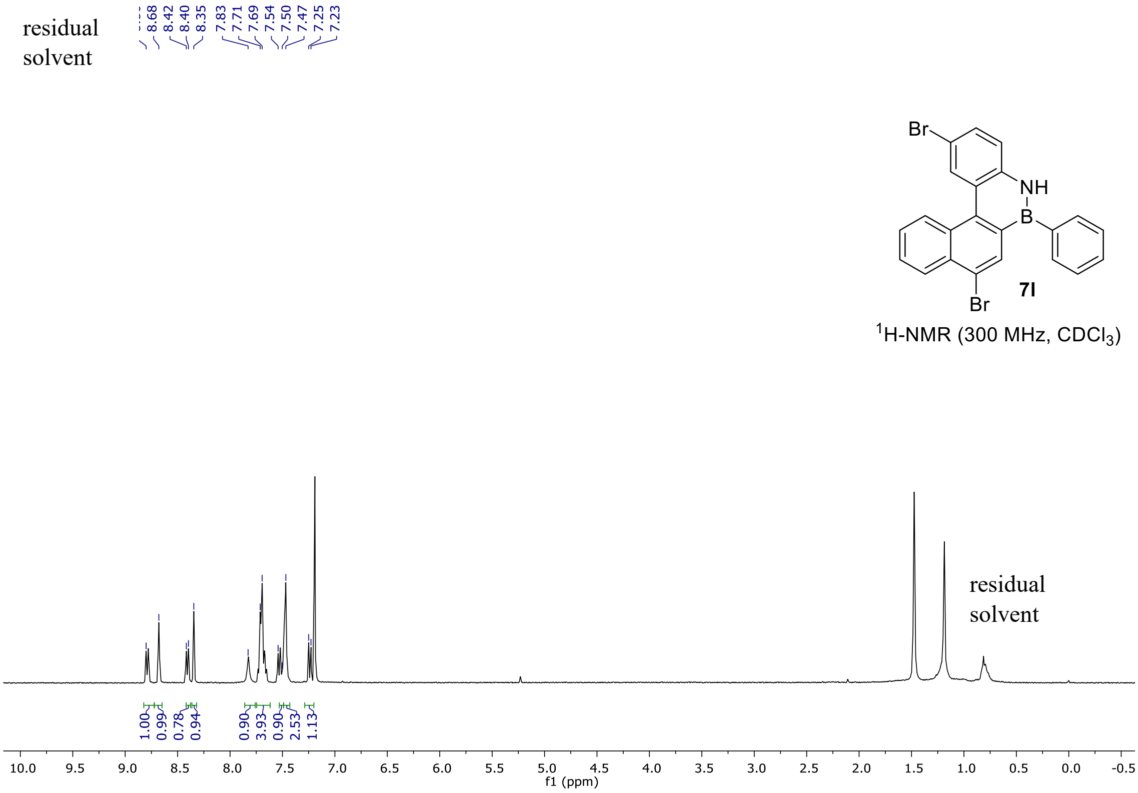

^{1}H-NMR (300 MHz, $CDCl_3$)

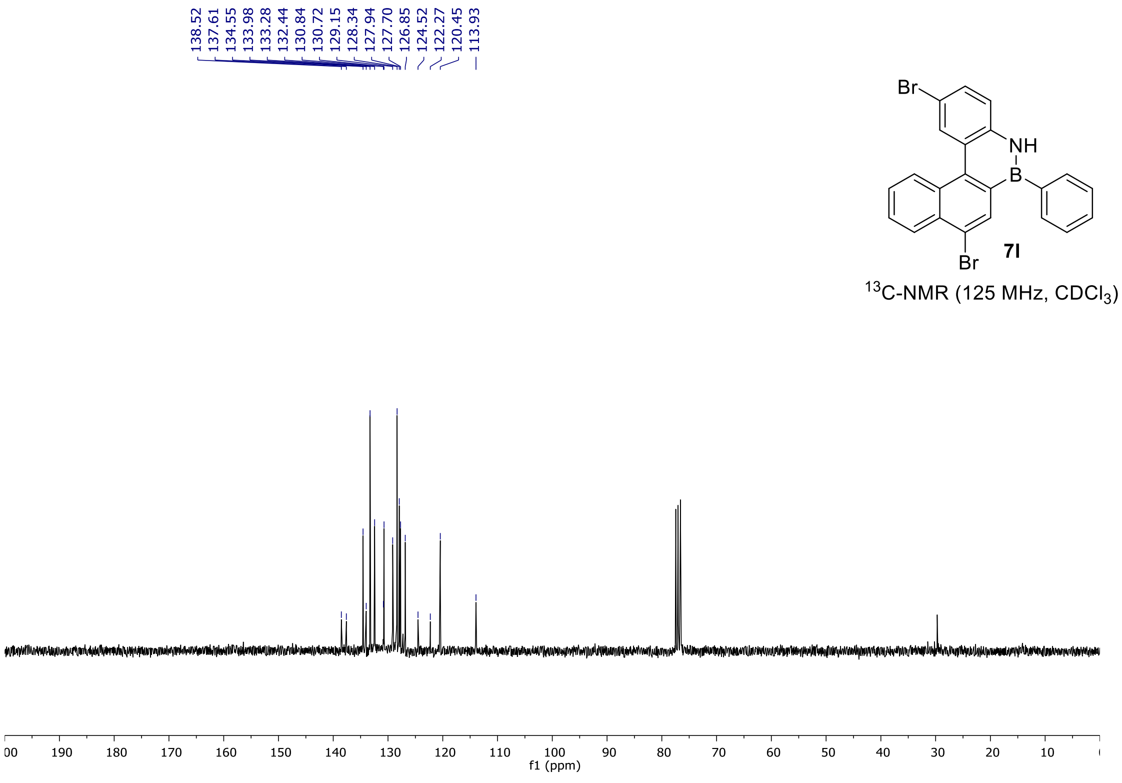

^{13}C-NMR (125 MHz, $CDCl_3$)

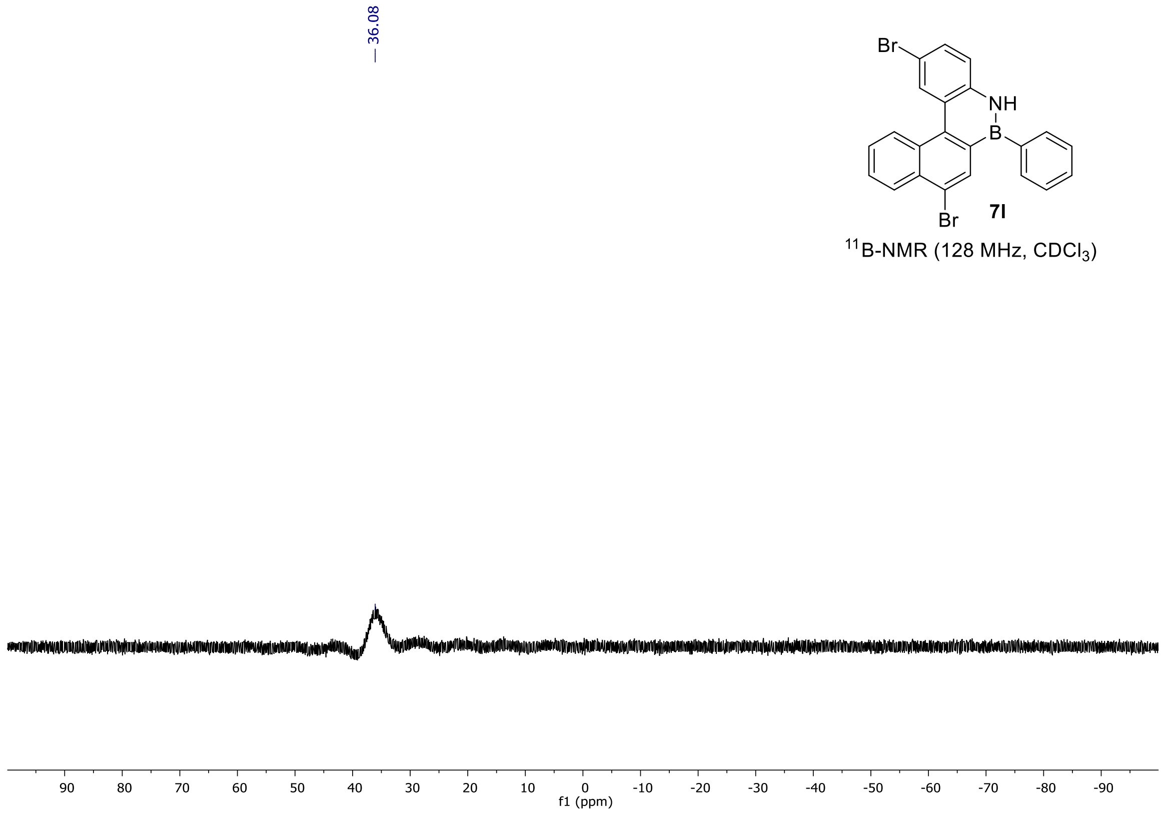

^{11}B-NMR (128 MHz, $CDCl_3$)

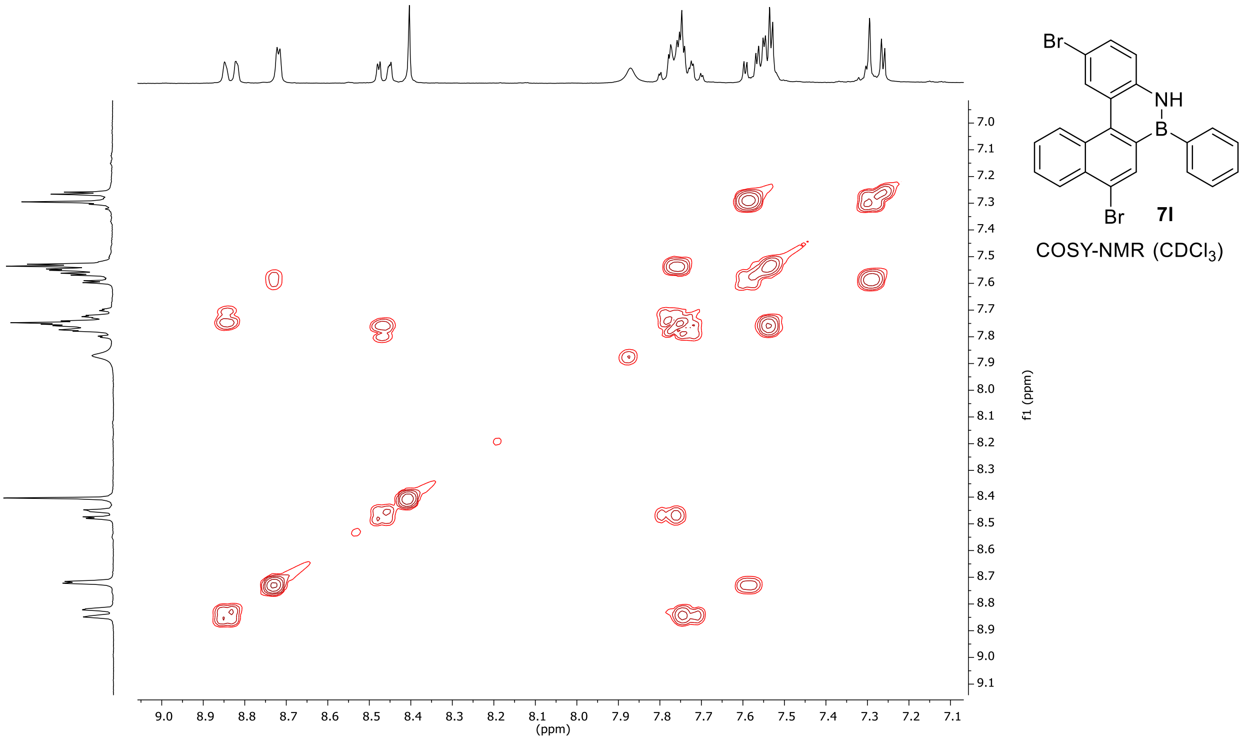

COSY-NMR ($CDCl_3$)

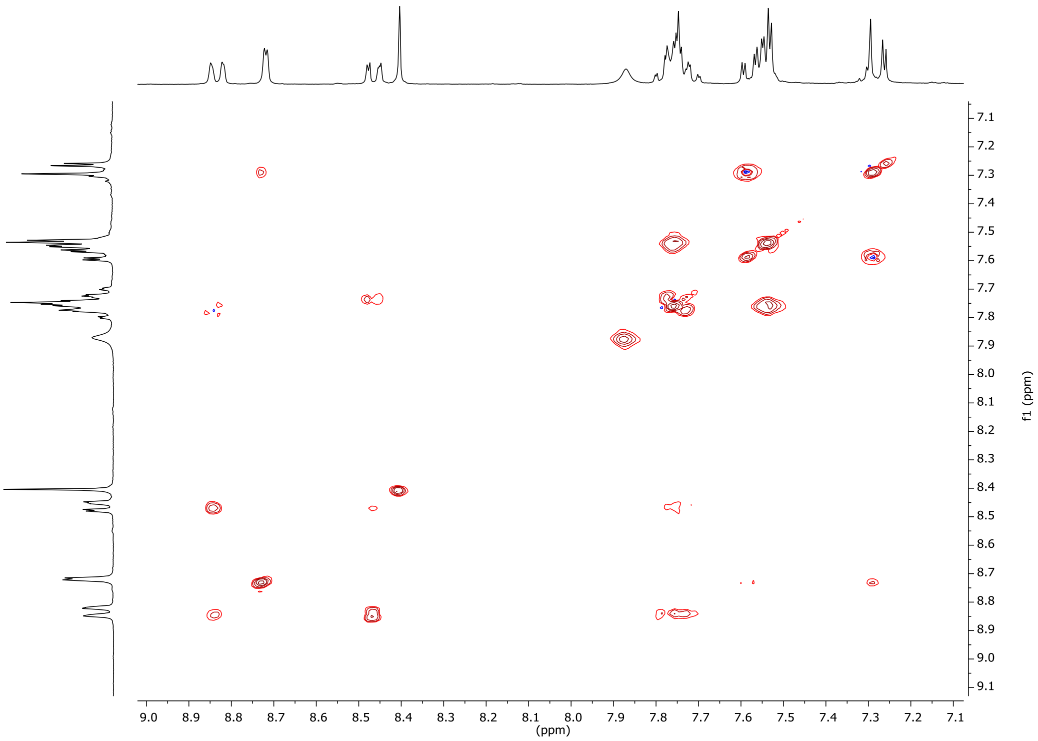

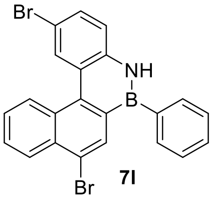

7l

TOCSY-NMR ($CDCl_3$)

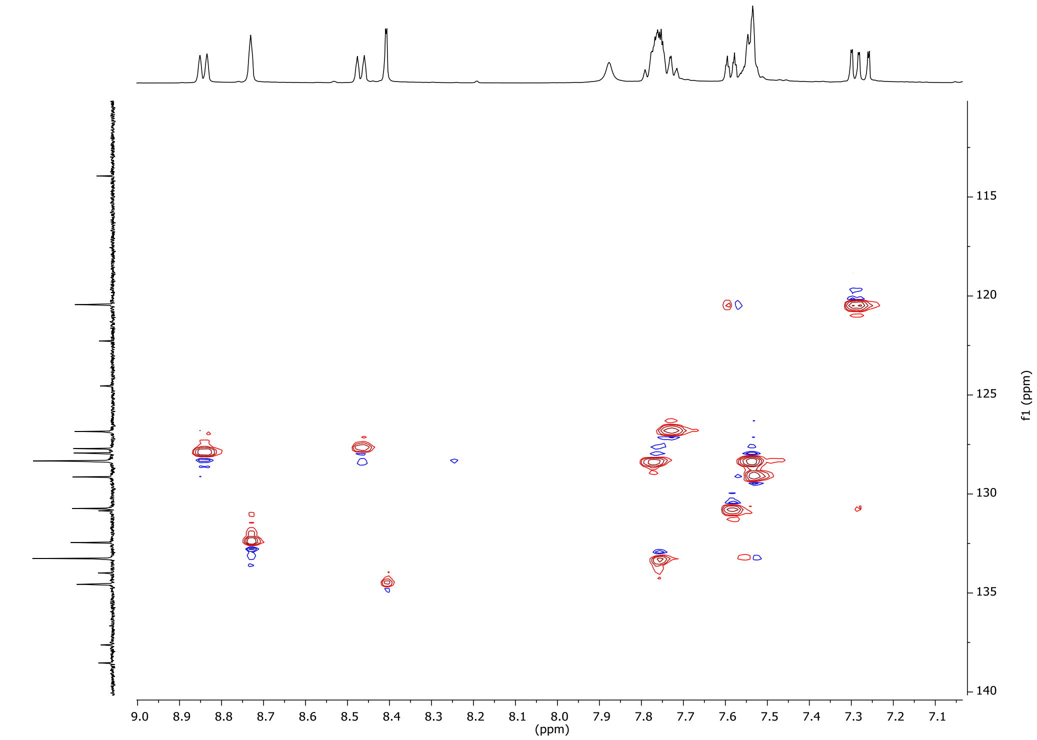

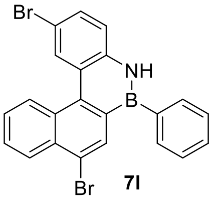

7l

HSQC-NMR ($CDCl_3$)

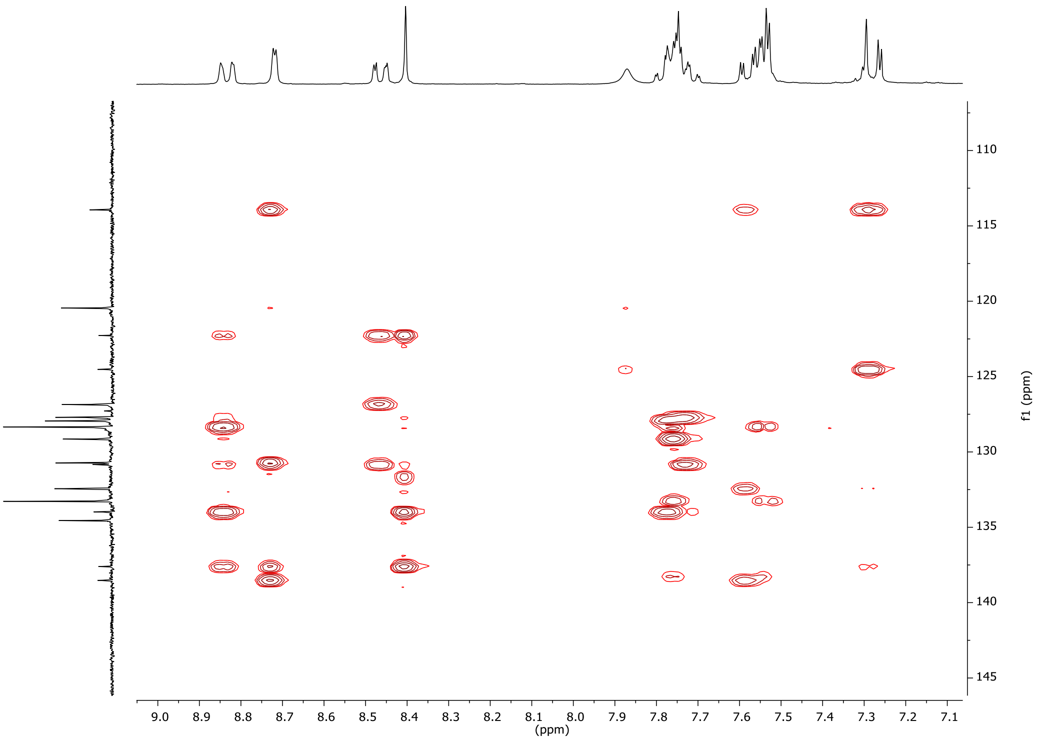

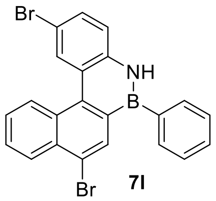

7l

HMBC-NMR ($CDCl_3$)

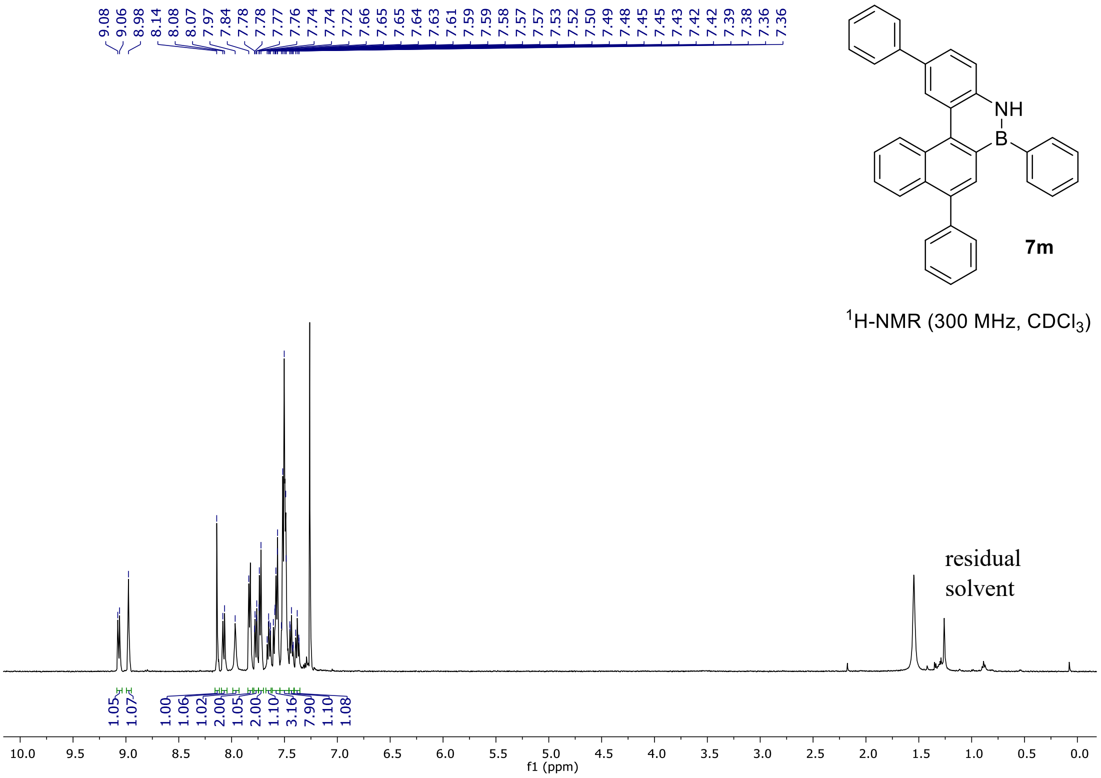

7m

^{1}H-NMR (300 MHz, $CDCl_3$)

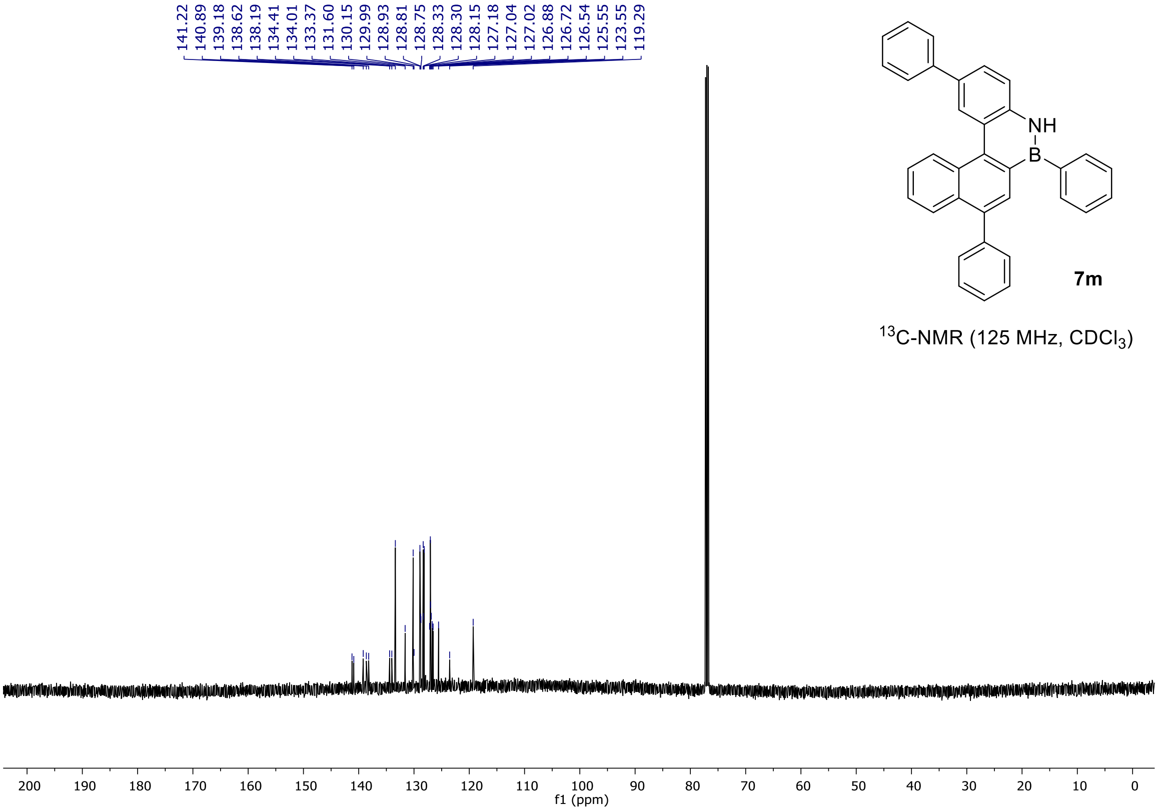

7m

^{13}C-NMR (125 MHz, $CDCl_3$)

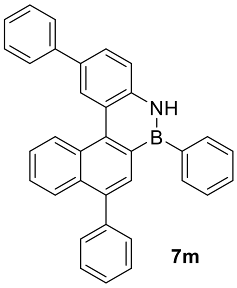

7m

^{10}B-NMR (128 MHz, $CDCl_3$)

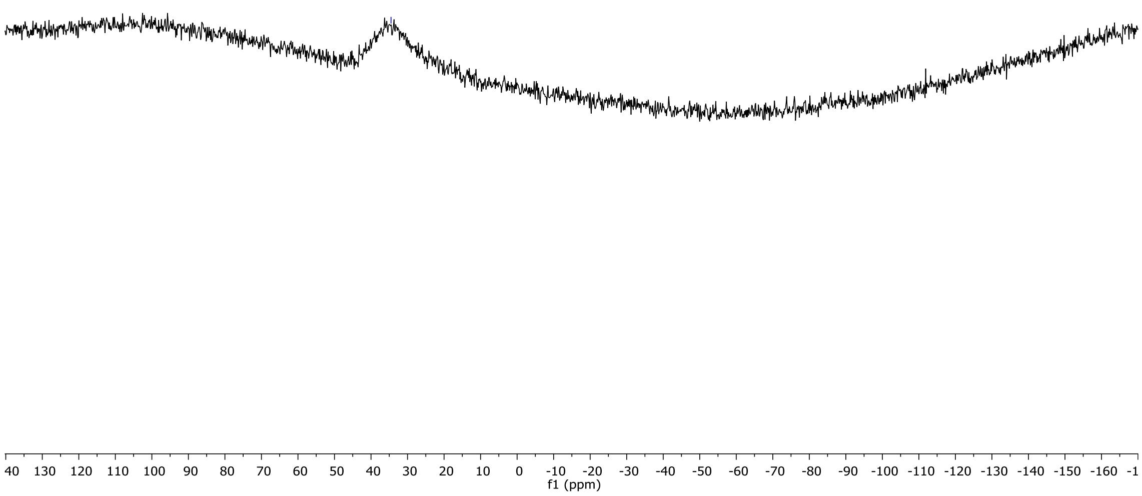

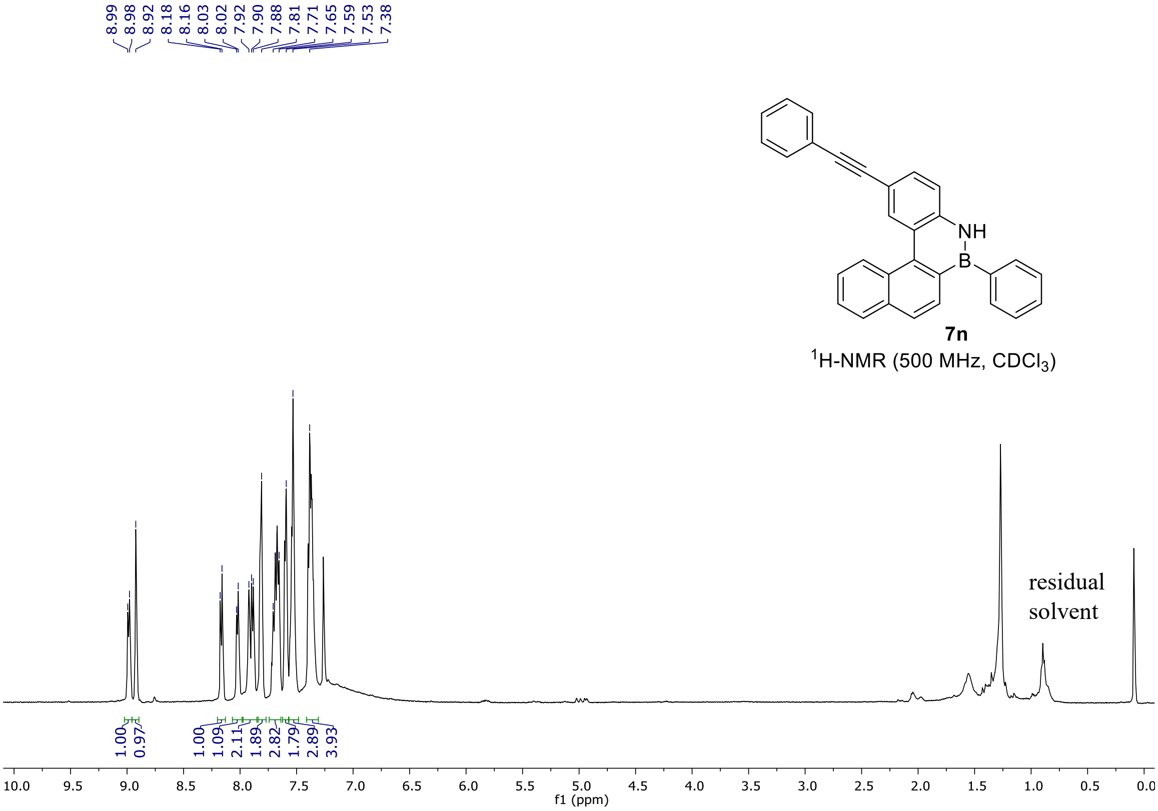
8.99 8.98 8.92 8.18 8.16 8.03 8.02 7.92 7.90 7.88 7.81 7.71 7.65 7.59 7.53 7.38
NH
B
7n
1H-NMR (500 MHz, CDCl3)
residual solvent
1.00 0.97 1.00 1.09 2.11 1.89 2.82 1.79 2.89 3.93
10.0 9.5 9.0 8.5 8.0 7.5 7.0 6.5 6.0 5.5 5.0 4.5 4.0 3.5 3.0 2.5 2.0 1.5 1.0 0.5 0.0
f1 (ppm)

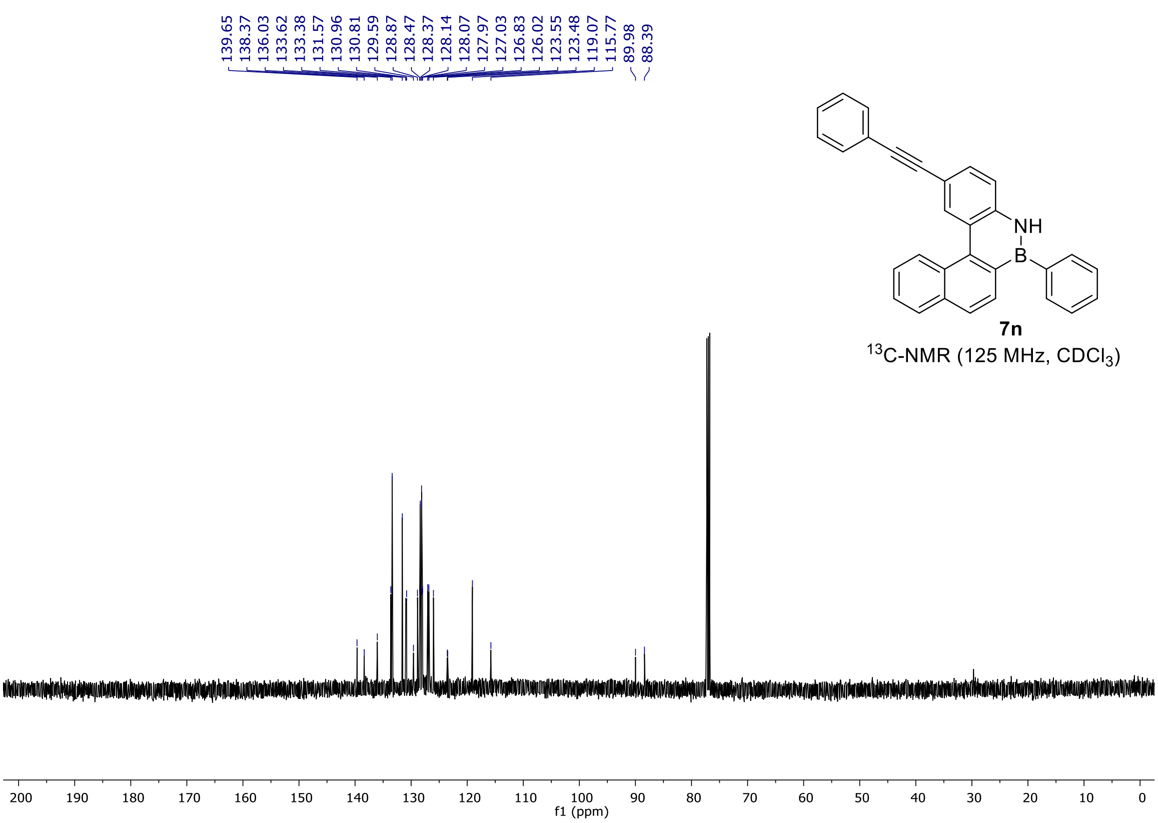
139.65 138.37 136.03 133.62 133.38 131.57 130.96 130.81 129.59 128.87 128.47 128.37 128.14 128.07 127.97 127.03 126.83 126.02 123.55 123.48 119.07 115.77 89.98 88.39
NH
B
7n
13C-NMR (125 MHz, CDCl3)
200 190 180 170 160 150 140 130 120 110 100 90 80 70 60 50 40 30 20 10 0
f1 (ppm)

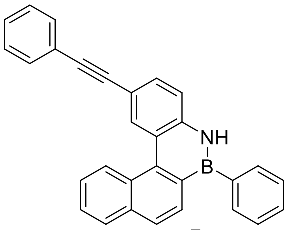

7n

^{11}B-NMR (128 MHz, $CDCl_3$)

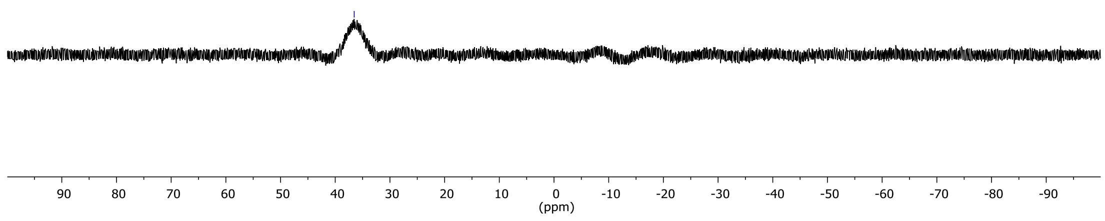

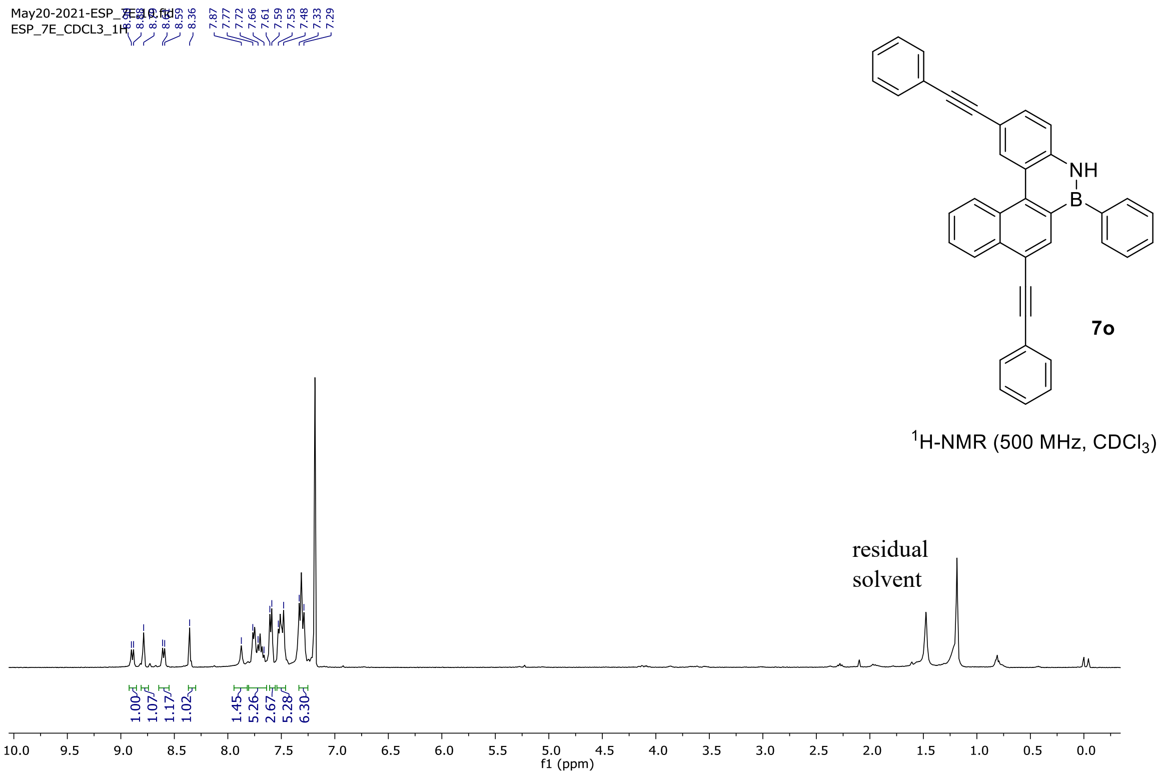

7o

^{1}H-NMR (500 MHz, $CDCl_3$)

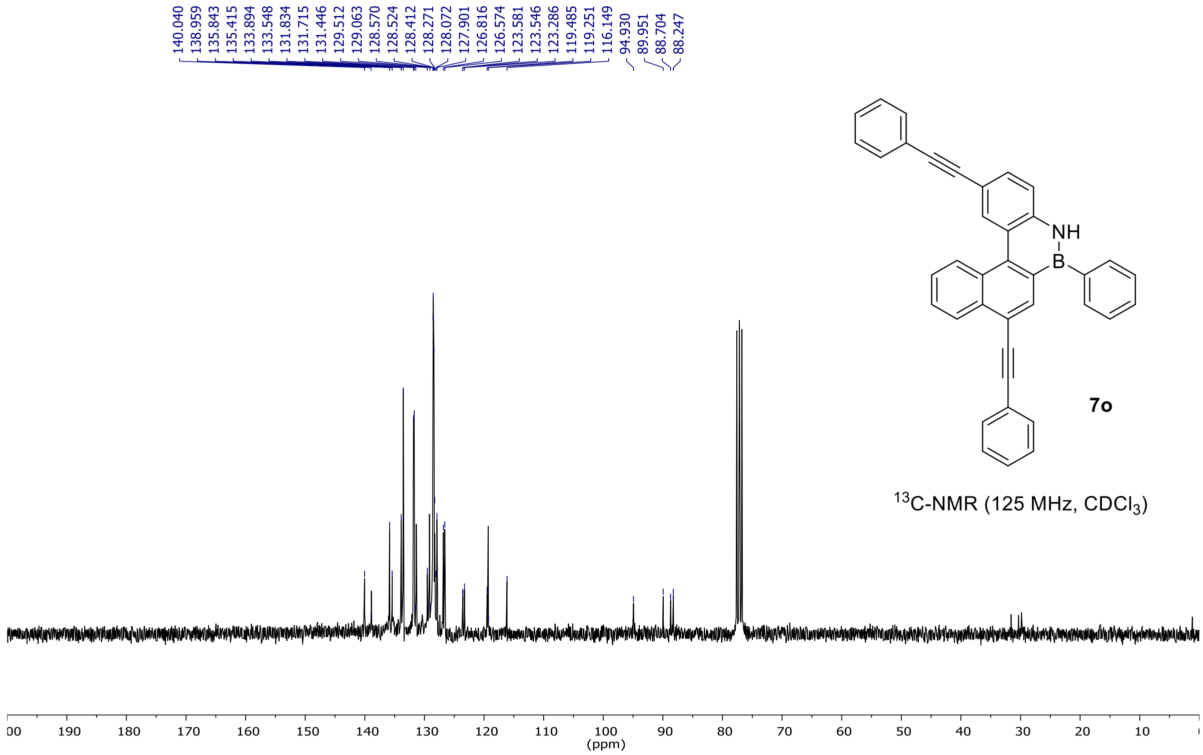

7o

^{13}C-NMR (125 MHz, $CDCl_3$)

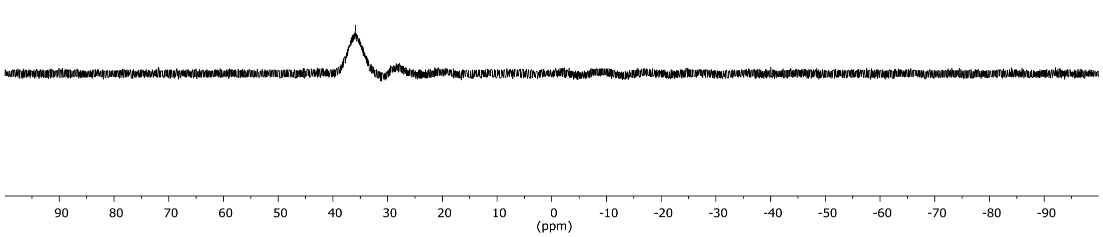

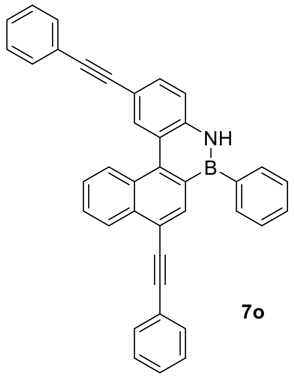

7o

^{11}B-NMR (128 MHz, $CDCl_3$)

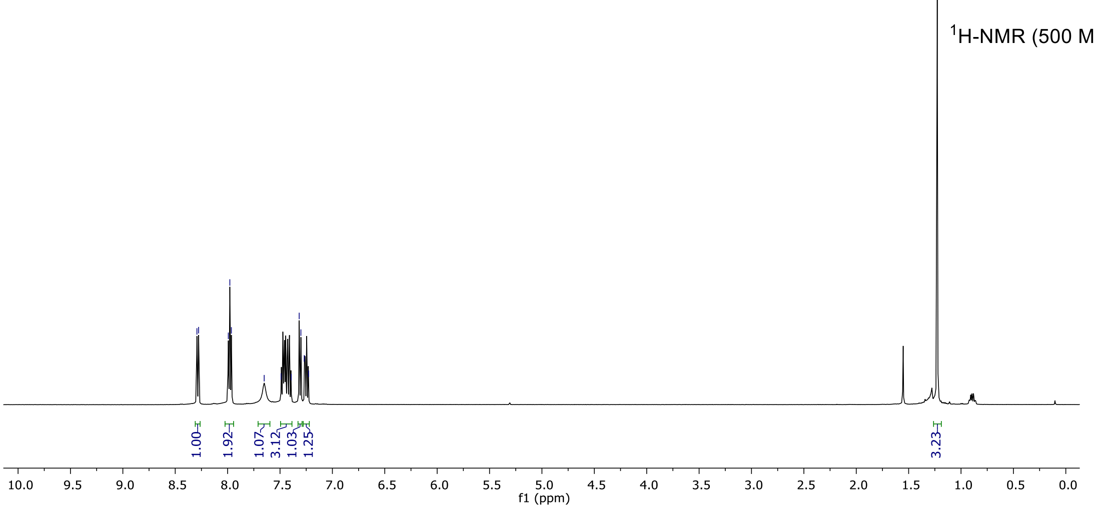

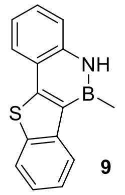

9

^{1}H-NMR (500 MHz, $CDCl_3$)

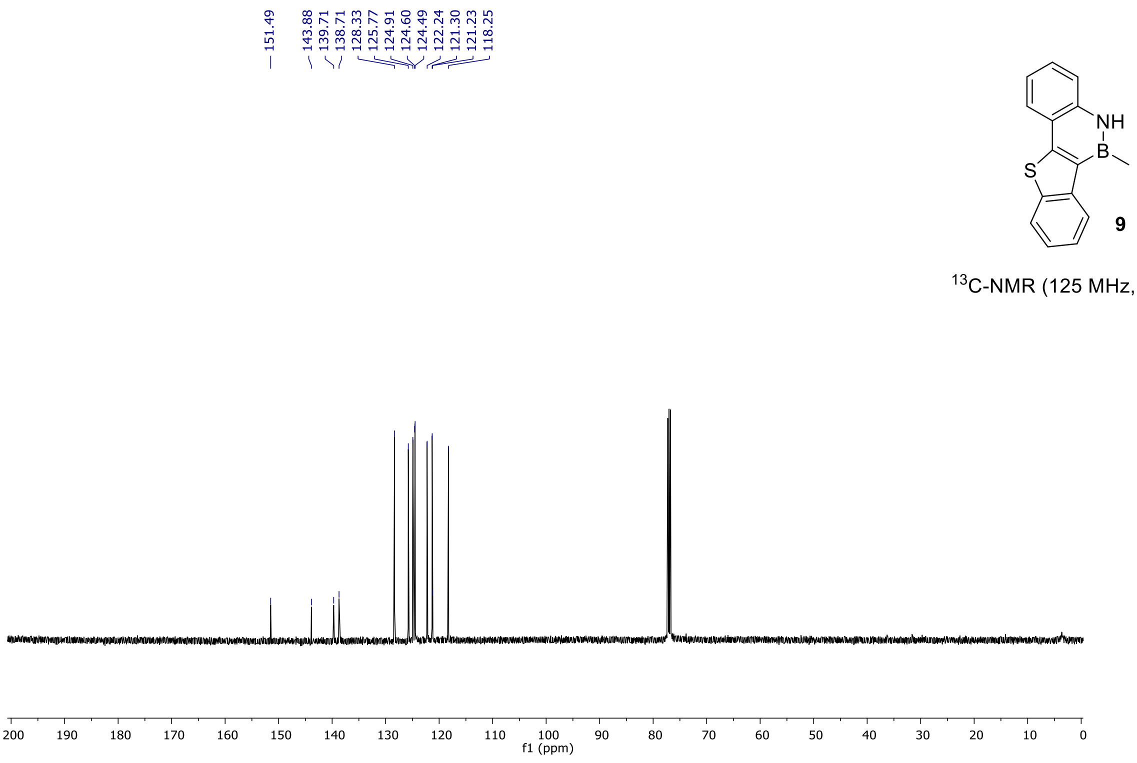

9

^{13}C-NMR (125 MHz, $CDCl_3$)

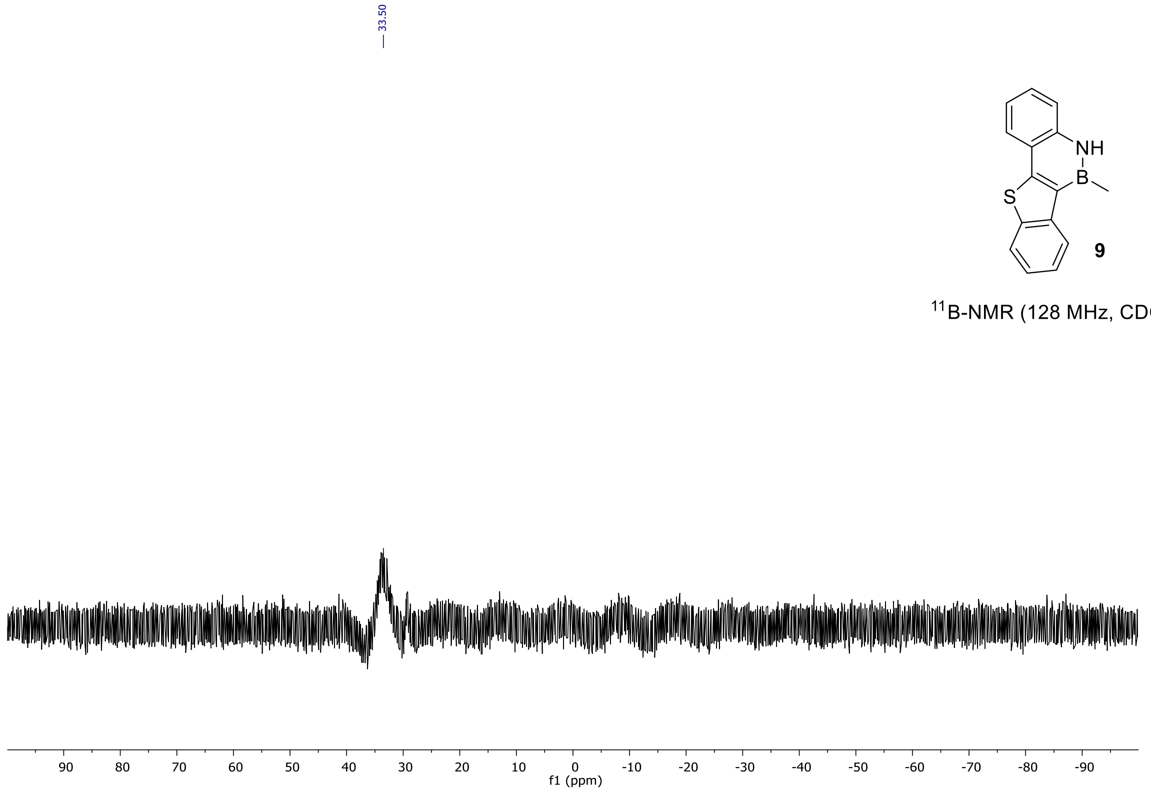

9

^{11}B-NMR (128 MHz, $CDCl_3$)

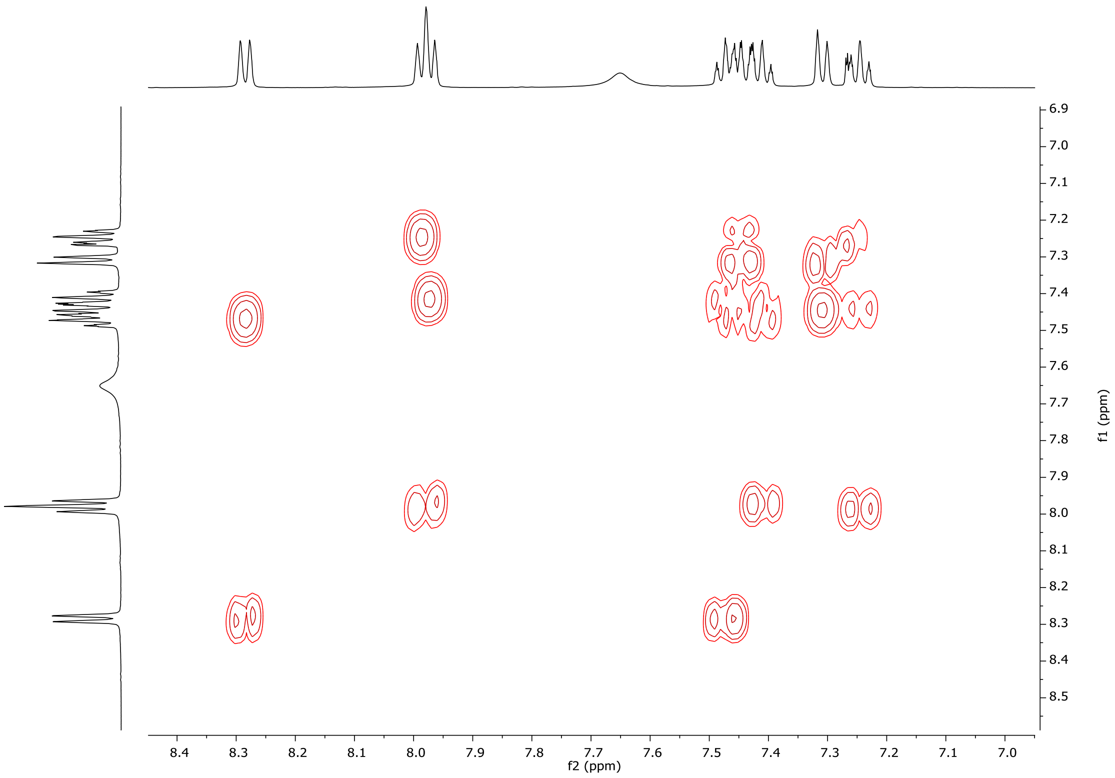

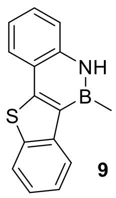

9

COSY-NMR ($CDCl_3$)

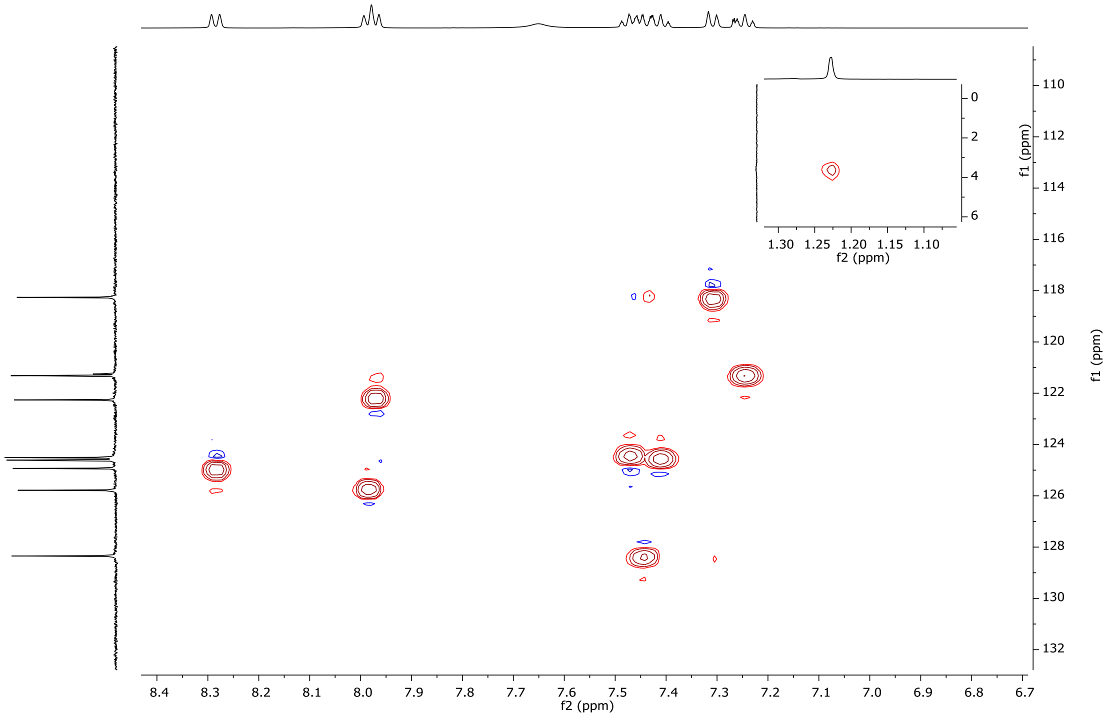

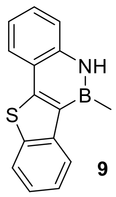

9

HSQC-NMR ($CDCl_3$)

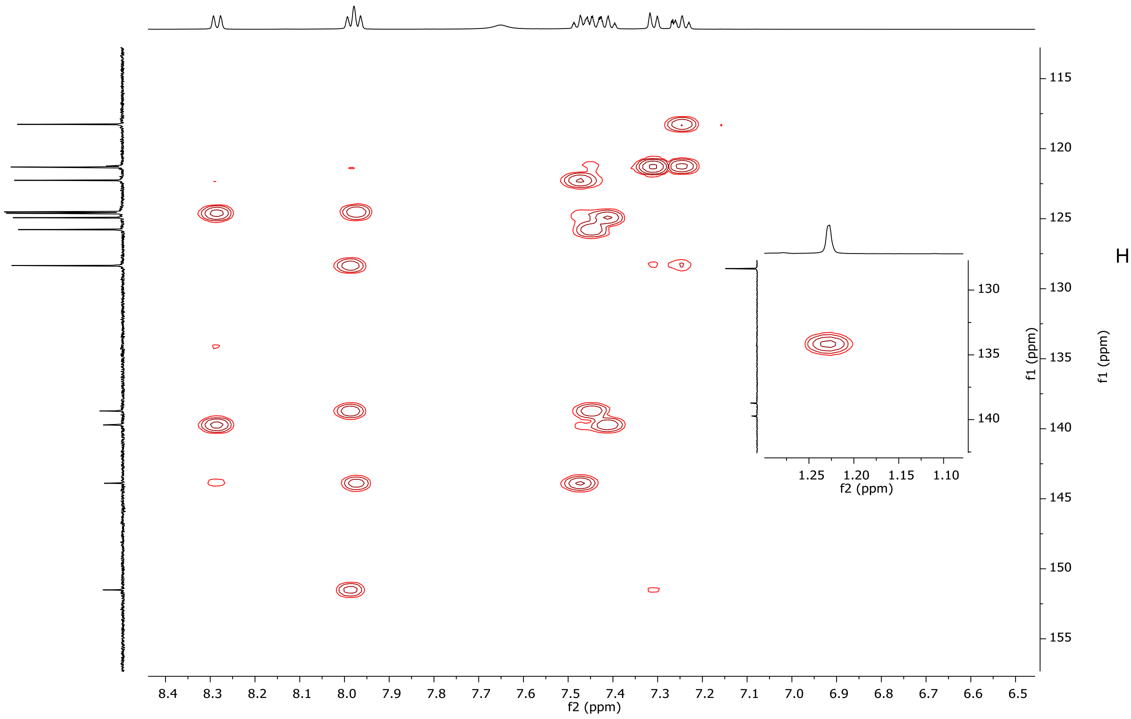

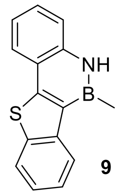

9

HMBC-NMR ($CDCl_3$)
